# Colonization of *Vitis vinifera* L. by the Endophyte *Trichoderma* sp. Strain T154: Biocontrol Activity Against *Phaeoacremonium minimum*

**DOI:** 10.3389/fpls.2020.01170

**Published:** 2020-08-04

**Authors:** Guzmán Carro-Huerga, Stéphane Compant, Markus Gorfer, Rosa E. Cardoza, Monika Schmoll, Santiago Gutiérrez, Pedro A. Casquero

**Affiliations:** ^1^ Research Group of Engineering and Sustainable Agriculture, Natural Resources Institute, Universidad de León, León, Spain; ^2^ Center for Health & Bioresources, AIT Austrian Institute of Technology GmbH, Tulln, Austria; ^3^ Area of Microbiology, University School of Agricultural Engineers, Universidad de León, Ponferrada, Spain

**Keywords:** *Trichoderma* colonization, vine, *Phaeoacremonium minimum*, indigenous strain, mycoparasitism

## Abstract

*Trichoderma* strains used in biological control products usually exhibit high efficiency in the control of plant diseases. However, their behavior under field conditions is difficult to predict. In addition, the potential of indigenous strains has been poorly assayed as well as their possible behavior as endophytes. Hence, niche colonization is a key feature for an effective protection. In this study, we aimed to: (i) explore the possibility of using a new *Trichoderma* strain isolated from vine to control pathogens, (ii) study the *in planta* interaction with the pathogen *Phaeoacremonium minimum* W. Gams, Crous, M.J. Wingf. & L. Mugnai (formerly *Phaeoacremonium aleophilum*), a pioneer fungus involved in Grapevine Trunk Diseases (GTDs) such as esca. For this purpose, fluorescently tagged *Trichoderma* sp. T154 and a *P. minimum* strain were used for scanning electron microscopy and confocal scanning laser microscopy analyses. Data showed that the *Trichoderma* strain is able to colonize plants up to 12 weeks post inoculation and is located in xylem, fibers, as well as in parenchymatic tissues inside the wood. The beneficial fungus reduced colonization of the esca-related pathogen colonizing the same niches. The main observed mechanism involved in biocontrol of *Trichoderma* against the esca pathogen was spore adhesion, niche exclusion and only few typical hypha coiling was found between *Trichoderma* and the pathogen. These results suggest that the *Trichoderma* strain has potential for reducing the colonization of *Phaeoacremonium minimum* and thus, an inoculation of this biological control agent can protect the plant by limiting the development of GTD, and the strain can behave as an endophyte.

## Introduction

During the last 50 years, intensive agriculture has led to a range of problems to human health, environment, flora, and fauna (Geiger et al., 2010). One of the crops requiring considerable amount of pesticides for increasing production is grapevine. *Vitis vinifera* L. (common grapevine) cultivars are among the most widely planted crops in the world and have a high commercial value (Gramaje et al., 2018). A significant amount of costs associated with these cultivars, however, come from intense pest and disease management programs (Cooper et al., 2012). Thus, important efforts are needed to search for alternative control strategies to reduce costs and dependence of chemicals. Currently, Grapevine Trunk Diseases GTDs (Bertsch et al., 2013; Roblin et al., 2019) are among the most important vine destructive diseases. In economic terms, in France, losses due to these diseases are estimated at around one thousand million € per year (Lorch, 2014) in yield production terms. In South Australia, losses were estimated in 1500 kg/ha (Wicks and Davies, 1999), and according to epidemiologic studies, an increasing incidence of GTDs has been found in the region of Castilla and León (Spain), increasing from 1.8% in 2001 to 7% in 2006 (Martín et al., 2007).

Since the ban of sodium arsenite in 2003 for controlling esca or Petri diseases, the use of Biological Control Agents (BCAs) has been described as an interesting, promising, and ecological strategy for controlling these diseases. Many BCAs have shown positive results against GTDs. For instance, bacteria such as a *Bacillus subtilis* isolate (ARC Infruitec-Nietvoorbij) have demonstrated reduction of symptoms (Ferreira et al., 1991; Fourie and Halleen, 2004). Also, indigenous strains of *Streptomyces* have been useful to reduce young grapevine decline caused by *Dactylonectria* sp., *Ilyonectria* sp., *Phaeomoniella chlamydospora*, and *Phaeoacremonium minimum* (Álvarez-Pérez et al., 2017) involved in GTDs.

Regarding fungi, *Trichoderma* strains have been also successfully tested in different conditions. Thus, under *in vitro* conditions, good results have been reported especially against causal agents of the trunk diseases such as *Phomopsis viticola, Eutypa lata, P. chlamydospora, Neofusicoccum australe, Neofusicoccum parvum, Diplodia seriata*, and *Lasidiplodia theobromae* (Kotze et al., 2011). In grapevine nurseries, *Trichoderma* strains have also shown the ability to control infections caused by *P. chlamydospora* and *P. minimum* during the grafting process (Pertot et al., 2016). However, in the field, positive and negative results have been obtained, and the grapevine trunk diseases continue to spread. The use of *Trichoderma* has been proved, also as an efficient protective method against *E. lata* in pruning wounds (Halleen et al., 2010) and can prevent black goo and necroses in the wood below the wound (Di Marco et al., 2004). The use of some *Trichoderma* strains or their metabolites was shown to control grapevine diseases (Mutawila et al., 2016b; Pascale et al., 2017). *Trichoderma* indigenous strains have been able to reduce the incidence of *Rhizoctonia solani* and inducing plant defense-related genes in bean plants (Mayo et al., 2015). However, little information is available on how *Trichoderma* indigenous strains could be protective and colonize endophytically the same niches as the pathogens. Preliminary studies suggest the possibility of using indigenous strains as promising agents for biocontrol in grapevines (Carro-Huerga et al., 2017; Carro-Huerga et al., 2019). Indeed, the use of *Trichoderma* to reduce symptoms and inoculum of pathogen in grapevine plants has been widely described (Di Marco et al., 2004; Fourie and Halleen, 2004; Halleen et al., 2010; Pertot et al., 2016). However, no studies are available regarding the niches, inside the plant, for *Trichoderma* colonization, nor to the kind of interaction established between *Trichoderma* spp. and pathogens belonging to the GTD complex inside grapevine plants.

In this study, we analyzed if an indigenous *Trichoderma* strain could colonize grapevine plants and protect vines against *P. minimum*. For that aim we analyzed: (i) the interaction between a *Trichoderma* sp. T154 isolated from vine and *P. minimum* under *in vitro* and *in planta* conditions, (ii) the niches of colonization of the *Trichoderma* sp. T154 and its behavior with the grapevine plant, and (iii) the persistence and mobility of pathogen and biocontrol agent in penetrating grapevine wood in the tripartite interaction grapevine-*Trichoderma*-*P. minimum*.

## Materials and Methods

### Fungal Strains

*Trichoderma* sp. isolate T154 was isolated from wood of *Vitis vinifera* cv. Tempranillo (unpublished data). This strain was stored at the culture collection of Plant and Pest Diagnostic Laboratory under accession number ULET154 (University of León, Spain).

*Phaeoacremonium minimum* CBS 100398 (formerly *Phaeoacremonium aleophilum* CBS 100398) a GTD related pathogen was used in this study. A *P. minimum*::*gfp7* (formerly *P. aleophilum*::*gfp7*) strain was also used for colonization studies. The latter strain was transformed to express *gfp* and its colonization behavior on vine cuttings was reported by Pierron et al. (2015). Both were initially analyzed under normal light microscope.

### *Trichoderma* Identification

After 3 days of growth on PDA medium, genomic DNA of the *Trichoderma* strain T154 was isolated from 100 mg of mycelia using the Nucleospin Plant II kit (Macherey-Nagel, Düren, Germany) according to the manufacturer’s instructions. Extracts were eluted in 50 μl of sterile water and DNA concentration was estimated using a NanoDrop ND-1000 Spectrophotometer (Thermo Scientific, Wilmington, DE, USA). PCR amplification was performed using 50 ng of template DNA in a final volume of 50 μl containing 10 mM Tris-HCl (pH 8.3), 50 mM KCl, 1.5 mM MgCl_2_, 0.2 mM for each dNTP, 400 nM for each primer, and 1.5 U of DreamTaq DNA polymerase (Thermo Scientific). ITS5-ITS4 were used to amplify nuclear rDNA-ITS regions (White et al., 1990). PCR products were first purified by the NucleoSpinExtract II kit (Machery-Nagel, Düren, Germany) and were then sequenced using primer ITS4 and the kit BigDye Terminator v3.1 Cycle Sequencing Kit (Applied Biosystems) and an automatic capilar sequencer ABI 3130xl (Applied Biosystems) according to the manufacturer’s instructions. For fungal identification, sequences were then compared with NCBI Genbank (National Center for Biotechnology Information, http://www.ncbi.nlm.nih.gov) databases using the BLAST program (http://www.ncbi.nlm.nih.gov/BLAST).

Secondly, for a more accurate analysis of the *Trichoderma* isolate, sequences of six housekeeping genes [*act1* (encoding for the actin); *cal1* (calmodulin); *fas1* (fatty acid synthase alpha subunit); *lcb2* (sphinganine palmitoyl transferase subunit 2); *rpb2* (RNA polymerase 2^nd^ largest subunit); and *tef1* (translation elongation factor 1-alpha)] were retrieved from the genome sequence of that isolate, and were used for a phylogenetic analysis against the same housekeeping sequences retrieved from GenBank genomic sequences of other 12 *Trichoderma* species belonging to four representative clades, i.e. clade Viride, Brevicopactum, Green Spored, and Longibrachiatum (**Supplementary Data S1: Figure S1**). The procedure used to retrieve all these sequences was as follows: First, sequences of these six genes were retrieved by BLASTn software from the genome of *T. harzianum* CBS 226.95, using as queries the sequences of fungal homologous genes, which were found at the GenBank database, against the genome sequence of that strain. Second, the six *T. harzianum* CBS 226.95 housekeeping genes were further used as queries to retrieve the homologous sequences from the genomes of the other 12 *Trichoderma* strains (including *Trichoderma* sp. T154), following the same procedure described above. Third, once the complete genomic sequences of the six housekeeping genes were available from the 13 *Trichoderma* strains used in this study, the intron regions were manually removed and the resulting coding sequences (cds) were used to generate the phylogenetic trees as indicated in the legend to (**Supplementary Data S1: Figure S1; Supplementary Data S3: Appendixes S1–S6**).

Sequences of the six housekeeping genes retrieved from the *Trichoderma* isolate identified in the present work were deposited in the GenBank under the accession numbers: MT701786 for *act1*; MT708552 (*cal1*); MT708557 (*fas1*); MT708564 (*lcb2*); MT708568 (*rpb2*); MT708571 (*tef1*).

### *Trichoderma* Transformation

To transform the *Trichoderma* sp. strain T154, a hygromycin B test was performed to determine its sensitivity to different concentrations of hygromycin B (50, 100, 150, and 200 μg mL^−1^) in PDA medium. It was obtained that 200 μg mL^−1^ was the optimum concentration for performing the experiment, no growth of *Trichoderma* was observed (**Supplementary Data S2: Figure S3**). The transformation of *Trichoderma* sp. T154 with plasmid pBHt2-tdTom (Caasi et al., 2010) was then carried out from a fresh *Trichoderma* sp. T154 spores suspension according to Cardoza et al. (2006). The binary vector pBHt2-tdTom contains Td tomato fluorescent protein under the control of the *tox*A promoter and the hygromycin resistance marker *hph*. Plates containing PPG [mashed-potato-glucose agar (Sousa, 2004)] medium were inoculated with 1 × 10^7^ spores and incubated at 28°C for 3 days. The spores collected from the plate were then used to inoculate 50 ml of CM (5 g malt extract, 5 g yeast extract and 5 g glucose and distilled water up to 1 L) medium and incubated in an orbital shaker at 250 rpm and 28°C for 24 h. Then, 25 mL of that culture were filtered through Nytal^®^ (30 μM pore diameter) (Maissa, Barcelona, Spain) and washed twice with 0.7 M NaCl. After that, the mycelium was re-suspended in 20 ml of NaCl 0.7M containing a mix of lytic enzymes (Lysing enzymes L-1412, Driselase D-8037, Chitinase C-6137, Sigma, USA) at concentrations of 5, 15, and 0.05 mg mL^−1^, respectively. The mycelium was then incubated at 30°C on an orbital shaker at 80 rpm for 20 h. Protoplast formation was analyzed under the microscope at the end of the incubation period to verify the hydrolysis of the mycelium cell-walls. Once the protoplasts were released, they were collected by filtration through Nytal^®^ filters (30 μm pore diameter) and centrifuged for 15 min at 4,000 rpm. The pellet was re-suspended in 0.5 ml STC buffer (10 mM Tris HCl; pH 7.5, 1.2 M sorbitol, and 50 mM CaCl_2_), counted with a Thoma cells, and diluted with STC solution to a concentration of 1 × 10^8^ protoplasts per ml. Finally, the protoplasts were mixed with solution 1 (v:v), to obtain a 5 × 10^7^ protoplast mL^−1^ (solution 1 was prepared by mixing five volumes of STC with 1 volume of PEG [10 mM Tris HCl; pH 7.5, 50 mM CaCl_2_, 30% polyethylene glycol 8000]). One-hundred microliters of this protoplast suspension containing 5 × 10^7^ protoplasts mL^−1^, were then mixed with 10 μg of linearized plasmid, pBHt2-tdTom, resuspended in 100 µl of STC, and 50 µl of PEG. This plasmid was previously linearized with the enzyme HindIII to facilitate the integration of the vector into the fungal genome. The plasmid and the protoplast suspension were then mixed and maintained at room temperature for 15 min, followed by another 15 min at 42°C. Then, 2 ml of PEG were added, and the mixture was incubated at room temperature for another 5 min. Finally, the mixture was diluted with 2 ml of STC and poured as an overlay on regeneration medium plates (27.4% sucrose; 0.1% yeast extract, 0.1% NZ-amine, and 1.2% Bacto-agar). The plates were maintained at room temperature for 5 to 10 min until the medium has solidified, and subsequently incubated at 28°C for 24 h to allow the regeneration of the protoplasts. Finally, a 1% agar overlay containing hygromycin B at a concentration of 200 µg mL^−1^ was added to the plates, and they were left in incubation, at 28°C, until the appearance of the transformants, for 3 to 5 days (**Supplementary Data S2: Figure S3**).

The transformants were analyzed and confirmed by PCR following the TERRA method (PCR Direct polymerase mix. Clontech, Mountain View, CA).

### Pure Culture Interaction

In addition to the plant assays and for evaluating the *in vitro* mycoparasitic interaction between *Trichoderma* sp. T154::*tdTom3* and *P. minimum*::*gfp7*, a confrontation dual assay was carried out as previously described (Kotze et al., 2011) with some modifications. The experiment was performed twice with three biological replicates each.

For analyzing both types of interaction and the mechanisms of biocontrol, confocal laser scanning microscopy **(**CSLM) and SEM were used. Thus, a mycelial plug of *P. minimum*::*gfp7* was placed on a 90 mm diameter Petri dish, and after 14 days (when the pathogen reached an important grade of growth) another plug of *Trichoderma* sp. T154::*tdTom3*, collected from a 7-day-old culture grown on PDA, was placed at 5-cm distance of the pathogen´s plug. The plates were incubated at 25°C for 10 days in the dark (**Supplementary Data S2, Figure S4**).

### Plant Interaction

One-year-old dormant grapevine grafted plants of Tempranillo/110 Richter combination from Vivai Cooperativi Rauscedo (Rauscedo, Italy) were potted up in June 2018. Plants were placed in a phytotronic chamber (photoperiod 16/8, 25°C; 45% humidity) and watered with autoclaved tap water during the experiment. Each plant was considered as a biological replicate and 12 biological replicates per treatment were performed. Budding took four to 6 weeks in 6-L pots containing a sterile mixture of vermiculite and turf (1:1 v/v). These plants (n= 72) were inoculated when at least six leaves were fully developed. In the upper part of the *Vitis vinifera* cv. Tempranillo, a wounding damage at the internode was made using a drilling device with a 3-mm drill head. For each sampling time (6 and 12 weeks) plants were inoculated with hyphae and spores of *Trichoderma* sp. T154::*tdTom3* (n=12), *Trichoderma* sp. T154 (n=12), *P. minimum*::*gfp7* (n=12), or *P. minimum* (n=12) and *Trichoderma* sp. T154::*tdTom3* + *P. minimum*::*gfp7* (n=12). For inoculation, all fungi were grown separately on three PDA plates. A cylindrical plug (3 mm long and 1 mm diameter) of *P. minimum*::*gfp7*, *Trichoderma* sp. T154::*tdTom3*, *P. minimum* or *Trichoderma* sp. T154 strains growing on PDA medium was applied to the wound. In the case of dual inoculation, *Trichoderma* sp. T154::*tdTom3* + *P. minimum*::*gfp7*, both of them were applied in the same hole simultaneously. Only hyphae in the periphery of the growing fungus were collected to avoid selecting fungal material at a different reproductive and metabolic stages at different locations on the same plate. Control plants were inoculated with a plug of uninoculated PDA medium. After inoculation, the wound was covered using a cellophane membrane. Then, plants were maintained in the phytotronic chamber with the same conditions described before and were watered once a week with autoclaved tap water. Plants were harvested after 6 and 12 weeks post-inoculation (**Supplementary Data S2, Figure S5**).

### Plant Sampling and Preparation for Microscopy

At sampling, i.e. at 6 and 12 weeks post-inoculation, secateurs were cleaned with Incidin™ (Ecolab, UK) and used to cut plants up to 15 cm above the inoculation point. All samples with different treatments were then kept in sterile paper bags at 4°C to avoid fungal growth (Mukherjee et al., 2013; Domingues et al., 2016). Samples were then prepared for analyzing the inoculation point by dividing the samples in small parts (technical replicates) (**Supplementary Data S2, Figure S4**).

### Confocal Laser Scanning Microscopy

Observations of fungi and plants were carried out using a confocal microscope (Olympus Fluoview FV1000 with multi-line laser FV5-LAMAR-2 and HeNe(G)laser FV10-LAHEG230-2, Japan). No additional treatment was applied to avoid destruction or reduction of the GFP and tdTom signals. Observations with the confocal microscope were done with objectives of 10×, 20×, and 40× and between 20 and 40 X, Y, Z pictures containing 20 to 60 scans were separately taken at wavelengths of 405, 488, 549 nm in blue/green/orange-red channels, respectively, with the same settings each time. The Imaris 9.3 software (Oxford Instruments) was then used at the confocal microscope to visualize 3D reconstructions. 3D modelling was further applied to the pictures to improve the fungal images. Each CSLM analyze of pure culture interaction consisted of three biological replicates that were analyzed containing three technical replicates. Plant interaction analyses consisted of 12 biological replicates that were analyzed containing four technical replicates.

### Scanning Electron Microscopy (SEM)

Observations of hyphae and spores of pure cultures and vine plant samples were carried out using a scanning electron microscope Hitachi TM3030 device (Hitachi, Germany) to further describe niches of fungal colonization in addition to the confocal microscopy. This study was performed immediately after the analysis with CSLM microscope. For this purpose, pure cultures or plant samples were frozen and fixed with a cooling stage at −25 C. A 15 kV accelerating voltage was used. Each SEM analyze of pure culture interaction consisted of three biological replicates that were analyzed containing five technical replicates. Plant interaction analyze consisted of 12 biological samples with five technical replicates. Each technical replicate was taken at three different magnifications and at least five pictures of every zone were taken where an interaction or a typical fungal structure were visualized. Combined analyses (CSLM and SEM) performed over *Trichoderma-*pathogen-plant interaction consisted of 12 biological samples containing three technical replicates.

## Results

### Identification of *Trichoderma* sp. T154 and *Trichoderma* sp. T154::*tdTom3* by CSLM and SEM Analysis

A preliminary identification of *Trichoderma* sp. T154, based on the analysis of ITS sequences by BLASTn software, indicates that it would correspond to a species close to *T. harzianum*. Thus, this fungal strain belongs to the clade Green Spored according to Jaklitsch and Voglmayr (2015). However, a more detailed phylogenetic analysis, based on the comparison of a concatenated sequence of six housekeeping genes (i.e. *act1, cal1, fas1, lcb2, rpb2*, and *tef1*) with the same sequences of 12 species belonging to the same or to other close *Trichoderma* clades (**Supplementary Data S1: Table S1**), indicated that the strain isolated in the present work, even when it is very close to some species of the Green Spored clade, could not be assigned to a species name (**Supplementary Data S1: Figures S1 and S2**). Pairwise distance values obtained from this analysis further confirmed this conclusion (**Supplementary Data S1: Table S2**). As result, based on these data, the strain isolated in this work was named as *Trichoderma* sp. T154.

This strain exhibited a massive production of spores after 7 days of growth and the typical structures of *Trichoderma* were visualized under normal light microscope. Isolate T154 was further transformed with the *tdTom* gene. As result, six transformants of *Trichoderma* sp. 154 were transferred to selection medium amended with hygromycin and after two selection rounds, a monosporic transformant was chosen. Transformant number 3, named *Trichoderma* sp. T154::*tdTom3*, exhibited the highest fluorescence intensity under a confocal microscope and was selected for further experiments. Conidia and conidiophores of this transformant showed a high red fluorescent signal. The fluorescence was intense and sometimes can be also visualized with punctuated fluorescence along the mycelium.

Similarly, to *Trichoderma* sp. T154, the *tdTom3* transformant also showed the typical *Trichoderma* structures, i.e. conidiophores and phialides (**Supplementary Data S2: Figure S6**).

### CSLM and SEM Analysis of *P. minimum* CBS 100398 and *P. minimum*::*gfp7*

*P. minimum* CBS 100398 was visualized for verifying that the fungus did not present any autofluorescence signal and was only visible under normal light. The *gfp* transformant of this strain showed a strong green fluorescence as expected (**Supplementary Data S2: Figure S7**) and shorts and unbranched conidiophores (**Supplementary Data S2: Figures S7A–C**). Also, typical warts of *P. minimum* on hyphae were observed (**Supplementary Data S2: Figure S8G**). This strain is identified as type II, phialides that are elongate-ampulliform and attenuated at the base tapering toward the apex (**Supplementary Data S2: Figures S8G, I**) (Crous et al., 1996; Mostert et al., 2005).

### CSLM and SEM Analysis of the Interaction Between *Trichoderma* sp. T154**::*tdTom3* and *P. minimum*::*gfp7* and Wild-Type Strains *In Vitro*

The use of the SEM microscope allowed to visualize the typical structures of both fungi in pure culture conditions with spores, mycelium, and phialides of transformed or wild-type strains, both for *Trichoderma* T154 wild-type, *Trichoderma* sp. T154::*tdTom3, P. minimum* wild type or *P. minimum::gfp7* (**Supplementary Data S2: Figure S8**).

**Figure 1:**
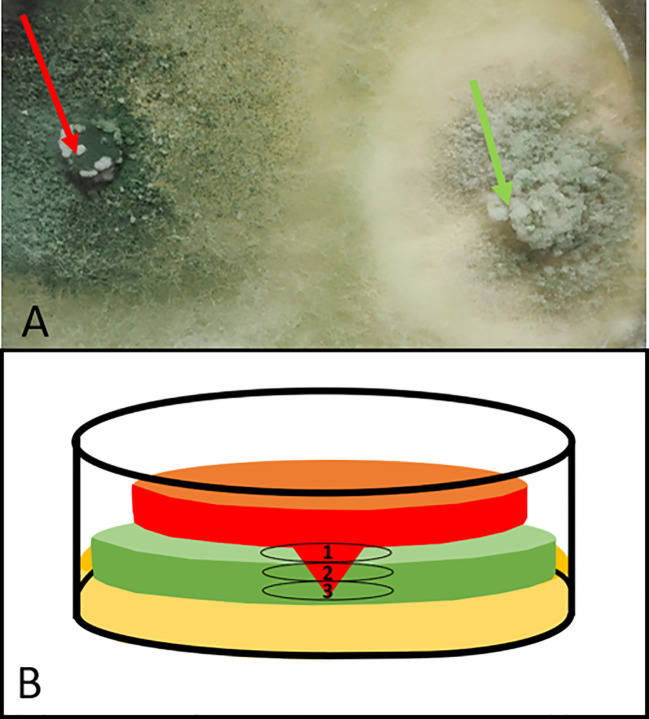
Dual confrontation culture of *Trichoderma* sp. T154::*tdTom3* (left plug) (red arrow) and *P. minimum*::*gfp7* (right plug) (green arrow): **(A)** zoom of a dual culture after 10 days of incubation. Pathogen has been totally colonized. **(B)** Drawing of a transversal section of Petri dish over the interaction between *Trichoderma* sp. T154::*tdTom3* (red) *and P. minimum*::*gfp7* (green) where three different levels can be observed. From the upper part to the bottom. 1, Upper Part. 2, Medium Part. and 3, Bottom Part. Light brown color at the bottom part reflects media culture.

The CSLM microscope was also used for evaluating the interaction between *Trichoderma* sp. T154::*tdTom3* and *P. minimum*::*gfp7.* During dual confrontation assay, *Trichoderma* sp. T154::*tdTom3* was able to colonize the whole Petri dish and exhibited a high ability to overgrow the pathogen (**Figure 1A**). In order to evaluate the overgrowth of *Trichoderma* that was completely covering the pathogen, each sample was evaluated three times over the same point using the tape touch method (Harris, 2000), and three different parts were observed (**Figure 1**): **1**) the upper part where *Trichoderma* was totally overgrowing the pathogen; **2**) a medium part in which there was a strong interaction between both fungi; and **3**) finally, the bottom part, where the pathogen was still resisting the attack of *Trichoderma* (**Figure 1**).

**Figure 2:**
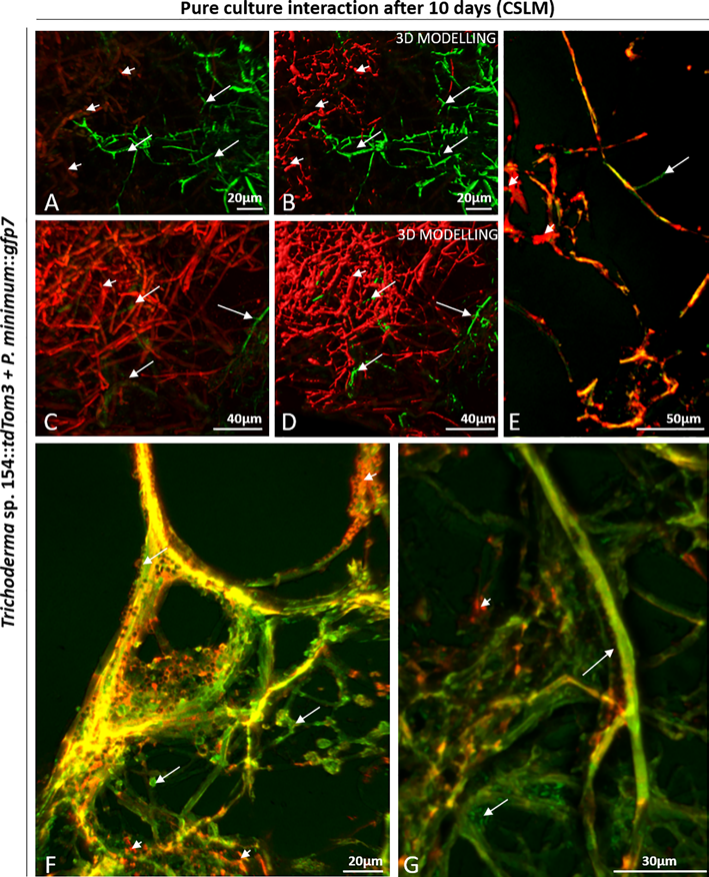
CSLM observation of the interaction zone in a dual culture of *Trichoderma* sp. T154::*tdTom3* (red) (arrowheads) and *P. minimum*::*gfp7* (green) (arrows). **(A**–**D)** Upper part, confrontation zone between *Trichoderma* sp. T154::*tdTom3 and P. minimum*::*gfp7*. **(E**, **F)** Medium part, *Trichoderma* sp. T154::*tdTom3* is controlling *P. minimum*::*gfp7*, and a clear interaction between both fungi is observed. **(G)** Bottom part of the sample, zone where *P. minimum*::*gfp7* is established. Representative pictures of biological replicates (3) are presented in this figure.

Each part was analyzed separately (**Figure 1B**). In the upper part, both fungi were confronted. Thus, a massive and strong presence of both fungi was detected under the microscope (green and red) (**Figures 2A, B**). Another situation was found in the upper part, where *P. minimum*::*gfp7* was trying to grow but *Trichoderma* sp. T154::*tdTom3* did not allow it due to a high amount of mycelium of the beneficial fungus (**Figures 2C, D**). In the medium part, the mycelia of both fungi were intimately linked (yellow color due to combination of green and red fluorescence) (**Figures 2E, F**). During this interaction, *Trichoderma* spores were detected over the *P. minimum*::*gfp7* and also mycelium of *Trichoderma* sp. T154::*tdTom3* was growing close to the mycelium of *P. minimum*::*gfp7* (**Figure 2F**). And finally, in the bottom part, green fluorescence color was predominant, indicating that most hyphae belong to *P. minimum::gfp7* (**Figure 2G**).

**Figure 3:**
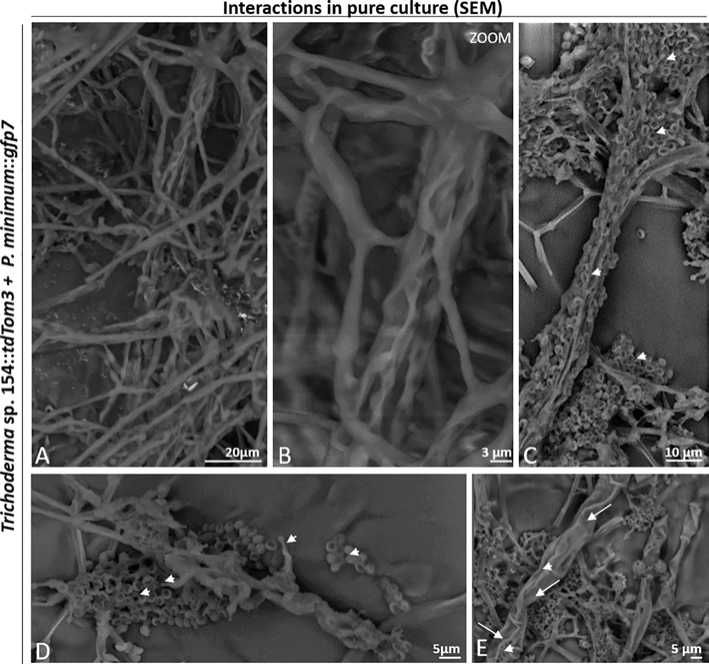
Interaction between *Trichoderma* sp. T154::*tdTom3* (arrowhead) and *P. minimum*::*gfp7* (arrows) in pure culture. **(A)** General overview of the interaction of both fungi. **(B)** Interaction of different hyphae of *Trichoderma* sp. T154::*tdTom3* over a hyphae of *P. minimum*::*gfp7* showing mycoparasitism. **(C)** Spores of *Trichoderma* sp. T154::*tdTom3* colonizing *P. minimum*::*gfp7*. **(D)** Mycelium of both fungi in contact. Spores and structures of *Trichoderma* can be distinguished. **(E)** Different hyphae of *Trichoderma* sp. T154::*tdTom3* colonizing *P. minimum*::*gfp7.* Representative pictures of biological replicates (3) are presented in this figure.

SEM analysis was further performed to visualize the interaction between both fungi in all interaction zones. The co-cultivation resulted in a mix of both mycelia (**Figure 3A**). Both fungi started to interact by growing in parallel (**Figures 3B, C**). *Trichoderma* spores were also observed, adhered to the pathogen´s hyphae (**Figures 3C, D**). Also, conidiophores of *Trichoderma* were detected, which were producing spores that were stuck to the mycelium of *P. minimum*::*gfp7* and where a hyphal coiling was observed (**Figures 3D, E**).

### Evaluation of Plant Inner Tissues After 6 Weeks Post *In Planta* Inoculation Using CSLM Microscope

**Figure 4:**
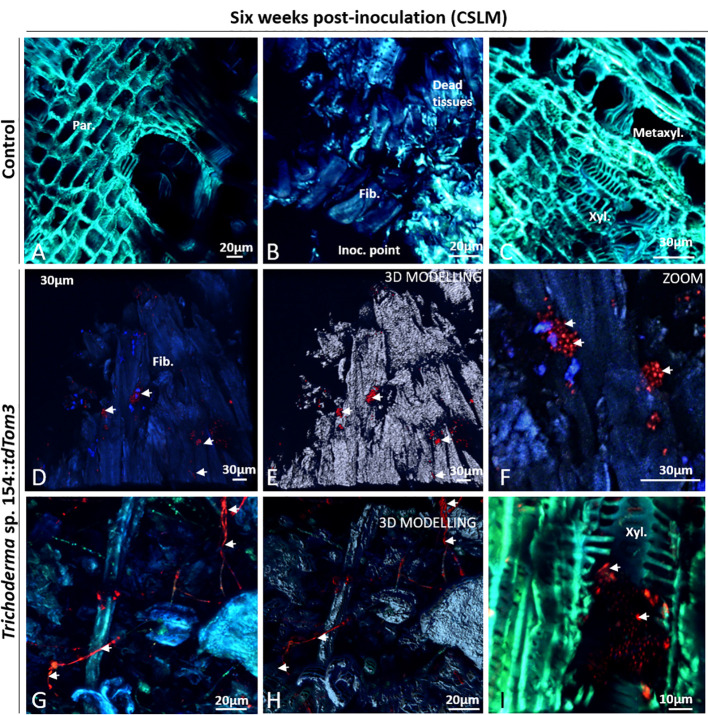
CSLM Control plants and plants inoculated with *Trichoderma* sp. T154*::tdTom3* (arrowhead) after 6 weeks. **(A)** Parenchyma zone without any sign of fluorescence. **(B)** Inoculation point showing different tissues free of fungi. **(C)** Xylem zone and occlusion close to the injured zone without any sign of fluorescent fungi. **(D**, **E)** Inoculation point colonized by spores and its 3D modelling identifying clearly all spores. **(F)** Zoom of the previous picture for showing a big group of *Trichoderma* sp. T154*::tdTom3* alive spores dispersed randomly inside tissues. **(G**, **H)** Inside dead tissues is possible to identify *Trichoderma* sp. T154*::tdTom3 mycelium*. **(I)** Xylem section showing spores of *Trichoderma* sp. T154*::TdTom3* inside it. Fib.: fibers, Inoc. point: inoculation point, Metaxyl.: metaxylem, xyl.:xylem. Representative pictures of biological replicates (12) are presented in this figure.

Plant samples were first evaluated after 6 weeks post inoculation. Mock controls were evaluated to confirm the lack of green or red fluorescence in any of the plant tissues after adjusting the settings to only detect GFP and red fluorescence from the fungi and to ensure that plants had not any type of structural alteration. First, regions close to the injury showed no presence of any red and green fluorescence in parenchymatic tissues (**Figure 4A**). Furthermore, no sign of transformed strains was found in dead tissues, where injury was performed with a PDA plug (**Figure 4B**). Other tissues close to the injury were also evaluated for confirming that mock controls did not show the presence of any kind of alteration and any green or red fluorescence (**Figure 4C**).

Colonization by *Trichoderma* sp. T154::*tdTom3* inoculated in plant vines was evaluated. Close to the injury, plant fibers were colonized by this fungus with small groups of spores (**Figures 4D, E**), but spores were also observed through different locations around the injury zone (**Figure 4F**). Fungal hyphae were also observed in this area (**Figures 4G, H**). However, *Trichoderma* sp. T154::*tdTom3* was not found far from the injury zone, and most of its presence was restricted to spores (**Figures 4D, I**) and few mycelial structures (**Figures 4G, H**).

**Figure 5:**
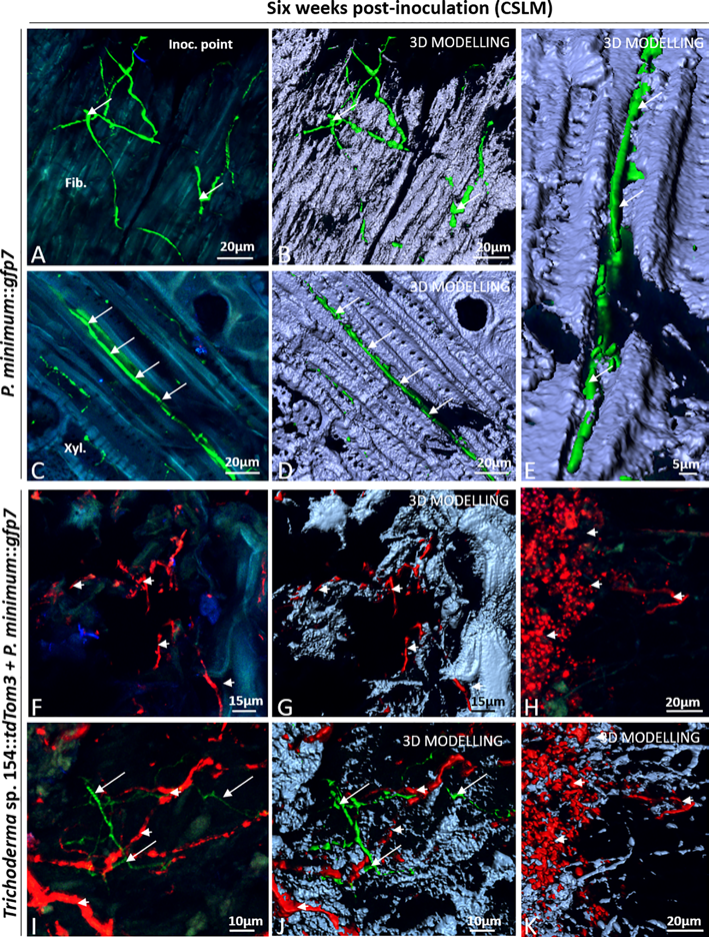
CSLM in plants inoculated with *P. minimum*::*gfp7* (arrows) and plants inoculated with both fungi, *Trichoderma* sp. T154**::*tdTom3* (arrowhead) and *P. minimum*::*gfp7* (arrows) after 6 weeks. **(A**, **B)** Mycelium of *P. minimum*::*gfp7* colonizing fiber tissues. **(C**–**E)** Hyphae of *P. minimum*::*gfp7* between xylem and phloem vessels following transversal section of tissues. **(F**, **G)** Dominance of *Trichoderma* sp. T154::*tdTom3* mycelium in dead tissues. **(H**–**K)** Presencce of spores and some hyphae of *Trichoderma* sp. T154**::*tdTom3* can be identified. **(I**, **J)** Interaction into plant between *Trichoderma* sp. T154**::*tdTom3 and P. minimum*::*gfp7* in different locations. Fib.: fibers, Metaxyl.: metaxylem, Phl: phloem, Xyl.:xylem. Representative pictures of biological replicates (12) are presented in this figure.

*P. minimum*::*gfp7* was mainly found, 6 weeks post inoculation, close to the inoculation point (**Figure 5A**) and full of hyphae were found around and inside the wood fibers or the parenchyma in this area (**Figure 5B**). This fungus was also present in alive tissues, but only in the xylem vessels (**Figures 5C–E**). Interestingly, no symptoms of esca or grapevine trunk diseases were detected.

Finally, the interaction between *P. minimum*::*gfp7* and *Trichoderma* sp. T154::*tdTom3* was evaluated *in planta*. Most images show the presence only of *Trichoderma* sp. T154::*tdTom3* (**Figures 5F–K**). In the injury zone, dead tissues were mostly colonized by hyphae of *Trichoderma* sp. T154::*tdTom3* (**Figure 5F**). In addition, 3D modelling demonstrated a medium degree of fungal colonization of the injury zone (**Figure 5G**). Also, fungal spores were found in this tissue (**Figures 5H, K**) as well as dead and alive fungal material exhibiting a very few blue fluorescence that was revealed by 3D modelling (**Figure 5K**) (corresponding to fungal cell-walls of either *Trichoderma*, *Phaeoacremonium* or natural endophytes). In dual inoculation, no fluorescence of *P. minimum*::*gfp7* was found in the injury zone (**Figures 5F–H, K**), and only in a few cases the mycelium of *P. minimum*::*gfp7* was observed in areas where *Trichoderma* sp. T154::*tdTom3* was present (**Figures 5I, J**).

### Evaluation of Plant Inner Tissues After 6 Weeks Post *In Planta* Inoculation by Comparing CSLM and SEM Microscope

CSLM and SEM were used over same tissues for evaluating fungal-plant interaction. First, using a CSLM, transformed fungi were identified using this technique because of the color emitted by fluorescent green and red proteins, and secondly, a SEM microscopy was performed to define the type of interaction.

**Figure 6:**
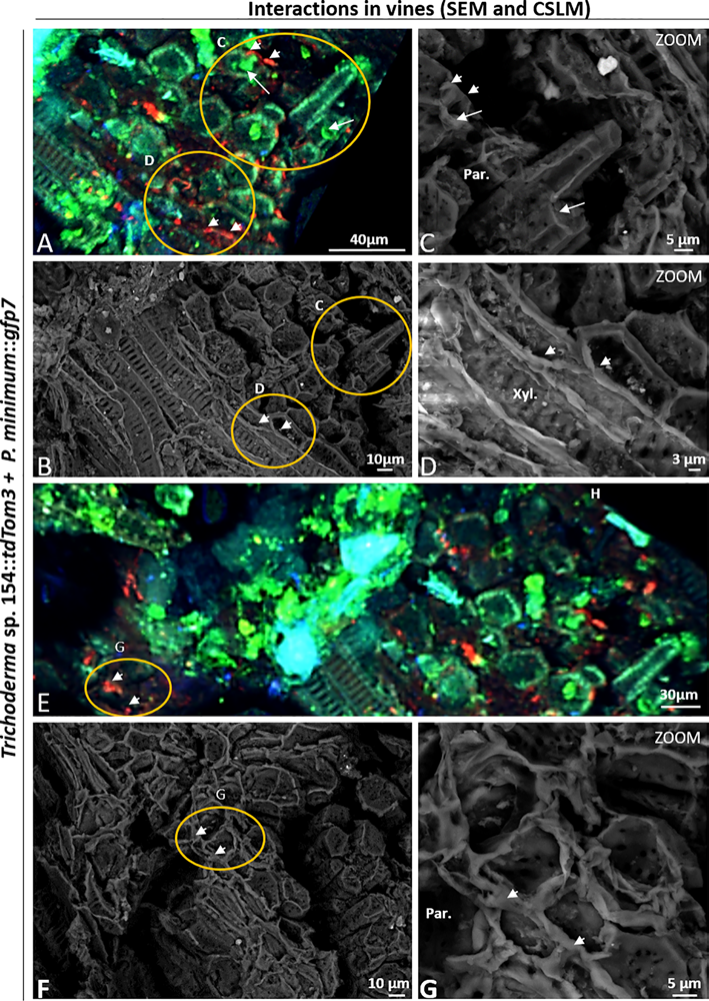
CSLM and SEM in plants inoculated with *P. minimum*::*gfp7* (arrows) and *Trichoderma* sp. T154**::*tdTom3* (arrowhead) *and P. minimum*::*gfp7* (arrows) after 6 weeks. **(A**, **B)** Detail of vine plant tissues that were analyzed by CSLM in the injury. **(C)** *P. minimum*::*gfp7* and *Trichoderma* sp. T154**::*tdTom3* colonizing parenchyma tissues in different locations. **(D)** *Trichoderma* sp. T154**::*tdTom3* over xylem vessels. **(E**, **F)** Another detail of vine plant tissues that were evaluated using CSLM in the injury**. (G)** *Trichoderma* sp. T154::*tdTom3* colonizing parenchymatic tissues. Par.: parenchyma, Xyl.: Xylem. Representative pictures of biological replicates (12) are presented in this figure.

CSLM microscope images have shown that many different tissues were colonized by the two fungi but they were never found in the same position (**Figures 6A–E**).

In both cases, the green and red fluorescences were located in different places without showing clear interactions between the fungi (**Figure 6A**). In **Figure 6C**, both fungi were found colonizing parenchymatic tissues but in different locations without showing any kind of interaction. Hyphae of *Trichoderma* sp. T154::*tdTom3* were found in the xylem vessels but growing in parallel to them, showing the behavior of a beneficial endophyte without causing any damage to the plant (**Figure 6D**). Using CSLM microscope we observed that other tissues were colonized (**Figure 6E**). A high performance of colonization in some parts was visualized for *Trichoderma* sp. T154::*tdTom3* and a strong but rare colonization of *P. minimum*::*gfp7* was observed (**Figure 6E**). No hyphal coiling was proved but *Trichoderma* was found as colonizing fibers possibly with another fungus (**Figures 6F, G**) and *P. minimum*::*gfp7* was detected in other places (**Figure 6E**).

### Evaluation of Plant Inner Tissues After 12 Weeks Post *In Planta* Inoculation Using CSLM Microscope

**Figure 7:**
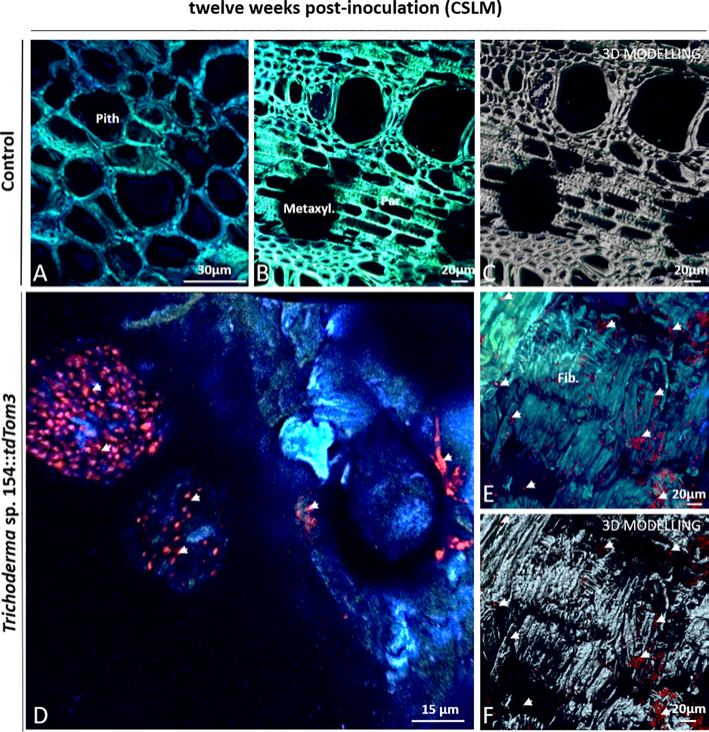
CSLM Control plants and plants inoculated with *Trichoderma* sp. T154**::*tdTom3* (arrowhead) after 12 weeks. **(A)** Pith zone without any sign of fluorescence. **(B**, **C)** Xylem zone and occlusion close to injured zone without any sign of fluorescent fungi. **(D)** Magnification of the inoculation point. *Trichoderma* sp. T154**::*tdTom3* spores can be seen with some hyphae. **(E, F)** Tissue colonized with plenty of *Trichoderma* sp. T154**::*tdTom3* spores distributed in fiber elements. Fib.: fibers, Metaxyl.: metaxylem, Par: parenchyma. Representative pictures of biological replicates (12) are presented in this figure.

After 12 weeks post inoculation, no differences were visualized in any structure of vine plants in comparison to mock inoculation (**Figures 7A–C**).

In the case of *Trichoderma*, after 12 weeks, large groups of spores and very few hyphae of *Trichoderma* sp. T154::*tdTom3* were found over the inoculation point (**Figure 7D**). In comparison to 6 weeks post inoculation, mycelium was indeed poorly found and spores were mainly visualized. In the wood fibers, xylem or parenchyma, close to the injury, a great number of *Trichoderma* sp. T154::*tdTom3* spores were also observed (**Figures 7E, F**). Thus, a poor colonization by the *Trichoderma* strain was observed after 12 weeks post inoculation.

**Figure 8:**
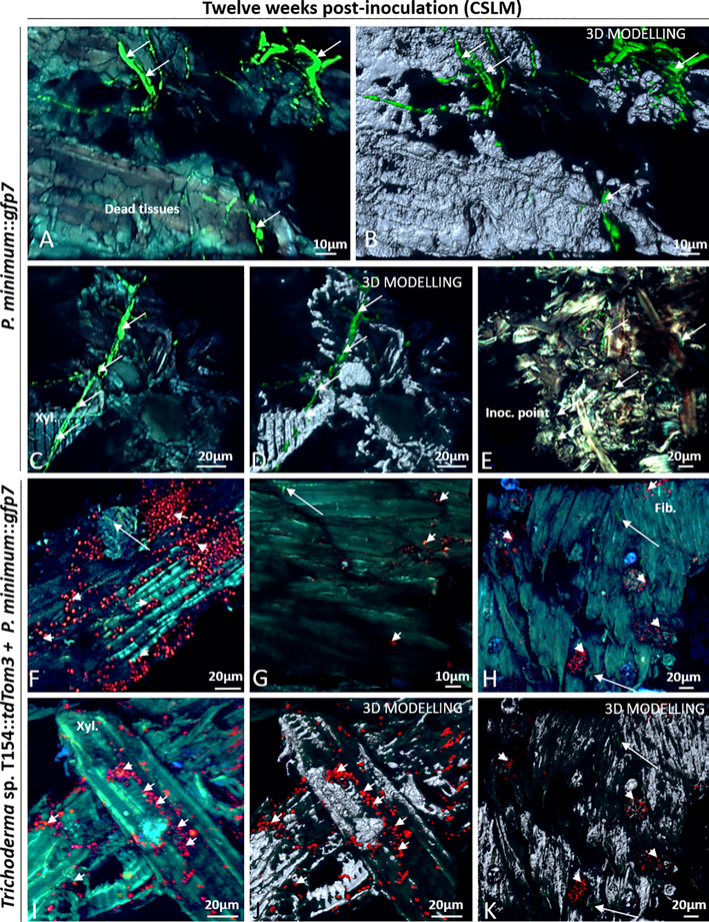
CSLM over plants inoculated with *P. minimum*::*gfp7* (arrows) and plants inoculated with both fungi, *Trichoderma* sp. T154**::*tdTom3* (arrowhead) *and P. minimum*::*gfp7* (arrows) after 12 weeks. **(A**, **B)** Fiber tissues colonized by hyphae of *P. minimum*::*gfp7.* **(C**, **D)** Xylem colonized by *P. minimum*::*gfp7* and expanding to other tissues. **(E)** Inoculation point colonized partially by *P. minimum*::*gfp7*. **(F)** Presence of spores of *Trichoderma* sp. T154**::*tdTom3* colonizing fibers and some hyphae of *P. minimum*::*gfp7*. **(G**–**I)** Few colonization of hyphae of *P. minimum*::*gfp7* and spores of *Trichoderma* sp. T154**::*tdTom3*. **(J**, **K)** Spores of *Trichoderma* sp. T154**::*tdTom3* over xylem vessels. Fib.: fibers, Inoc. point: inoculation point, Xyl.:xylem. Representative pictures of biological replicates (12) are presented in this figure.

In the case of single inoculation of *P. minimum*::*gfp7*, mycelia were visualized mainly colonizing the xylem, wood fibers or parenchyma in dead tissues close to the injury (**Figures 8A–E**).

In dual inoculation, *Trichoderma* spores were found in most images (**Figures 8 F–I**). A very few hyphae of *P. minimum*::*gfp7* was observed over different locations but they were found as colonizing the same tissues as *Trichoderma* sp. T154::*tdTom3* (**Figures 8G, H, J, K**). *Trichoderma* sp. T154::*tdTom3* was found mainly at xylem tissues (**Figures 8I, J**). No *in planta* interaction was found after 12 weeks between *Trichoderma* and the pathogen, so that, no SEM was performed for this reason.

## Discussion

Previous studies have evaluated *Trichoderma* strains isolated from grapevine plants against *P. minimum* under *in vitro* conditions (Kotze et al., 2011; Carro-Huerga et al., 2017). Also, the influence of *Trichoderma* species inoculated on grapevine cultivars has been previously described for protecting vine against GTD´s (Mutawila et al., 2011a). The optimum period for inoculating *Trichoderma* is 6 h after pruning in cultivars Chenin Blanc and Cabernet Sauvignon in field conditions (Mutawila et al., 2016a). Moreover, different experiments about colonization have described a good wound colonization after applying *Trichoderma* strains (John et al., 2005; John et al., 2008) Mutawila et al. (2011b) described grapevine woody tissue colonization by *Trichoderma harzianum* using strains carrying reporter genes, one expressing *gfp* (STE-U 6517) and the other *DsRed* (STE-U 6518). Mutawila et al. (2011c) described grapevine woody tissue colonization by *Trichoderma harzianum* using strains carrying reporter genes, one expressing *gfp* (STE-U 6517) and the other *DsRed* (STE-U 6518) (Mutawila et al., 2011b). For pathogens such as *P. chlamydospora* and *E. lata*, over one-year-old canes were then used for evaluating the efficacy of the *Trichoderma* strain, and the authors concluded that *Trichoderma harzianum* reduced the growth of the inoculated pathogens, being re-isolated more frequently from the xylem than from the pith and reaching at maximum depths of 30 mm in dual-inoculated shoots. Albeit (GFP)-labeled *Trichoderma harzianum* strains were used, no *Trichoderma* strains were visualized in grapevines nor their behavior inside tissues, rather they were used for re-isolation from plant tissues. Only the pathogens were visualized *in planta*.

There was no evidence using biomarkers, such as GFP or tdTom, about the three-way interaction *Trichoderma-*plant-pathogen, which corresponds to an approach that would simulate the natural interactions occurring in agro-ecosystems (Lu et al., 2004; Vinale et al., 2008).

In our study, we deciphered the location of a *Trichoderma* indigenous strain inside the plant and its behavior as an endophyte in grapevine wood; also, its interaction with the pathogen *P. minimum*, related to esca and Petri diseases, was tested in *in vitro* assay and in grapevine plants by CSLM and SEM.

Some strains of *Trichoderma* spp. are endophytes (Jaklitsch et al., 2006; Gazis et al., 2011) and several strains are recognized as promising biological control agents against GTDs (González and Tello, 2011). For instance, strains of this genus have been tested during the last 15 years against Botryosphaeria dieback, Esca complex and Eutypa dieback with promising positive results (Mondello et al., 2018). *Trichoderma* genus is the second most abundant of the endophytic mycobiota in woody tissues (Bruez et al., 2014). The use of grapevine endophytes shows a significant protection against other grapevine diseases (Ferrigo et al., 2017). Thus, it can be an important source for searching for an efficient biological control strain. However, the location of these fungi inside the plant has been poorly studied, and we indeed did not know before this study where the *Trichoderma* strains could be present in vines as well as on how it can interact with some vine pathogens as *P. minimum*.

In this study, we evaluated the exact location of one strain of *Trichoderma* that was isolated from grapevine cv. Tempranillo and its behavior upon penetration into the same variety and into a 1-year-old injured tissue. Thus, transformants of *Trichoderma* sp. T154 with *tdTom* gene were selected, which allowed to visualize red fluorescence under fluorescence microscopy. The strain was identified as colonizing parenchyma, fibers, and xylem vessels inside wood mainly after 6 weeks post inoculation. Fluorescence of *P. minimum* (GFP) allowed to distinguish both fungi in co-inoculation. The *P. minimum* strain was located mainly in xylem vessels as an endophyte during times of experiments.

To ensure that interactions are the same between wild-type strains and transformed fungi, *in vitro* assays on plates were performed (see **Supplementary Material**). Previously to analyzing any kind of interaction in grapevine plants, plates with *Trichoderma* sp. T154, *Trichoderma* sp. T154::*tdTom3*, *P. minimum*, *P. minimum*::*gfp7*, and interaction of both transformed fungi were visualized by CSLM and in SEM, which let to identify conidiophores, conidia, and hyphae. These fungi were also visualized under normal light (data not shown) to show that no macroscopic and phenotypic differences were found between the transformants and their wild-type strains.

*Trichoderma* sp. strain T154 was able to overgrow the pathogen. The most usual observation was adhesion of spores to the pathogen hyphae. Coiling of hyphae was also observed, albeit rarely. In some cases coiling interaction was indeed identified in agreement with previous descriptions (Lu et al., 2004), but only in a few cases.

The main mycoparasitism mechanisms exhibited by *Trichoderma* in this work were spore adhesion and parallel growth adhesion. These results are in agreement with previous reports, analyzing a *T. harzianum* strain against different wood decay fungi (Murmanis et al., 1988). Alignment of *Trichoderma* with the host hyphae and spore adhesion were identified as the main mechanisms of action. In this case, no typical coiling and hooks were visualized. Also a *T. harzianum* strain from the product Biotricho [Agro-Organics (PTY) Ltd., RSA] showed hyphal adhesion as a main mechanism of action against *P. minimum* as determined with a microscopic analysis. Furthermore, strains belonging to other *Trichoderma* species showed this kind of interaction against other pathogens such as *Diplodia seriata* or *Neofusicoccum parvum* (Kotze et al., 2011). Also, parallel growth was observed for the *T. harzianum* strains AG1, AG2, and AG3 (Agrimm Technologies Ltd, New Zealand) (John et al., 2004).

In our study the niches of colonization were analyzed. After 6 weeks, *P. minimum*::*gfp7* was only found close to the fibers next to the injury and mainly very close to the inoculation point, which is in agreement with previously described results (Fleurat-lessard et al., 2014; Pierron et al., 2015). *P. minimum::gfp7* was able to colonize xylem. In comparison to Pierron et al. (2015), a different scion and kind of tissue (one-year-old tissue) was assayed. No fungus was found outside of the inoculation points. In most of the sites tested, a strong fungal presence was shown mainly in all dead tissues in the injury and xylem vessels. Thus, a different adaptation to the cultivar could be a reason for the observed differences.

After 6 weeks post inoculation, *Trichoderma* sp. T154::*tdTom3* exhibited a good development in plant, which resulted in the finding of a high proportion of hyphae and spores. Plant fibers were colonized, with different groups of spores and hyphae inside this type of tissue. However, after 12 weeks, the strain of *Trichoderma* was found at a very low rate only in fibers. Most of *Trichoderma* hyphae started to colonize the injury but after 12 weeks most of them were not fluorescent, which would indicate a poor endophytic ability of this strain inside the grapevine plant or a lack of development due to not enough nutrients, pH, moisture. Thus, further studies are needed for evaluating the capability of growing under these conditions to optimize applications.

In this work, the environmental conditions in the growth chamber were 24°C and 45% of relative humidity, which were similar to those described previously (Pierron et al., 2015). Good results were obtained at a combination of high relative humidity and high temperature (24°C × 95°C and 100%RH and 28°C and 95%RH) with BCAs (Hannusch and Boland, 1996), being a very important factor for a successful colonization. This refers that different climatic events such as raining could improve *Trichoderma* colonization.

Colonization of grapevine plants by *Trichoderma* sp. T154::*tdTom3* and *P. minimum*::*gfp7* (after 6 and 12 weeks) revealed less quantity of *P. minimum* in most of the sites investigated. After the evaluation of 6 weeks post inoculation was done, most of *P. minimum* mycelium had disappeared and only spores have been able to survive in comparison to *Trichoderma* sp. T154::*tdTom3*. Spores of *Trichoderma* sp. T154 were found in xylem vessels and fibers. Inoculation was done inside the plant, so no bark colonization was analyzed. No movement of fungal mycelium and spores through the plant was detected. This finding suggests that both fungi did not show a spread of colonization during this short period of time. But, according to the reduction of pathogen inoculum, if any of these fungi arrives and colonizes the injury, it will not allow the other one to grow, hence prevention is the most important thing for avoiding GTD´s in vines. In conclusion, applying a biocontrol agent after pruning or planting could protect plant for the most critical period when the plant is very vulnerable to be infected.

Both fungi colonize parenchymatic cells and were located around plant cells enabling normal development of the plant. Xylem vessels and parenchymatic tissues were other types of tissues colonized by the *Trichoderma* strain. In addition, no interaction between the two fungi was found in grapevine plants where fungi where able to establish different niches of colonization inside the plant. However, the pathogen colonization was reduced. This reinforces the idea that the first fungus that arrives and is established outcompetes the other one. Mechanisms are likely related to secretion of antifungal substances by the *Trichoderma* strain, induction of the plant systemic resistance, and niche exclusion. This is in agreement with previous reports, indicating that competitive exclusion would be a key factor for persisting in the wood (Mutawila et al., 2011a).

## Conclusion

The results indicate that an indigenous *Trichoderma* strain can reduce *P. minimum* plant colonization during their endophytic colonization and can also exclude the pathogen from plant niches. Both fungi colonize different plant tissues, such as xylem vessels and parenchymatic cells. Albeit hyphal coiling from *Trichoderma* around pathogen is a well-known mechanism of action described for *Trichoderma* spp., our study shows that rather spore adhesion and niche exclusion constitute the main mechanism of action for biocontrol of the pathogen as analyzed by microscopic studies. Indigenous *Trichoderma* spp. have potential for reducing the colonization of *P. minimum*, a pioneer agent causing GTD. Thus, an inoculation of one of these BCAs can protect the vine plant by limiting the development of the disease.

## Data Availability Statement

The raw data supporting the conclusions of this article will be made available by the authors, without undue reservation.

## Author Contributions

GC-H, SC, MG, RC, SG, and PC designed the experiments. GC-H, RC, SG, and MG made the fungal transformation. GC-H and SC made the observation. GC-H, SC, MG, RC, MS, SG, and PC performed the data interpretation and manuscript preparation. All authors contributed to the article and approved the submitted version.

## Funding

The grant awarded to GC-H (FPU15/04681) comes from the Ministry of Education, Culture, and Sport (Spain). We thank Pago de Carraovejas winery for the project “GLOBALVITI IDI-20120746” “Solución global para mejorar la producción vitivinícola frente al cambio climático basada en robótica, tecnología IT y en estrategias biotecnológicas y de manejo del viñedo” (Global solution for enhancing viticulture production against: climatic change based on: robotics, IT technology, biotechnological strategies, and vineyard management) that was granted by the Centro para el Desarrollo Tecnológico Industrial –CDTI-. SC and MG received funding *via* DaFNE Project Nr. 101384 from the Austrian Federal Ministry for Sustainability and Tourism (BMNT).

## Conflict of Interest

The authors declare that the research was conducted in the absence of any commercial or financial relationships that could be construed as a potential conflict of interest.
