# Supplementary material for: Colonization of Vitis vinifera L. by the Endophyte Trichoderma sp. Strain T154: Biocontrol Activity Against Phaeoacremonium minimum
Source: Front Plant Sci. 2020 Aug 4;11:1170. doi: 10.3389/fpls.2020.01170 (PMC7417607; doi:10.3389/fpls.2020.01170)
Supplement: Supplementary file 1 [file DataSheet_1.pdf]

Niches of colonization of *Vitis vinifera* L. by an endophyte *Trichoderma* sp. T154 strain and its biocontrol activity against *Phaeoacremonium minimum*.

G. Carro-Huerga[1], S. Compant [2], M. Gorfer [2], R.E. Cardoza Silva[1,3], M. Schmoll[2], S. Gutiérrez[1,3], P.A. Casquero[1*]

[1] Research Group of Engineering and Sustainable Agriculture, Natural Resources Institute, Universidad de León, 24071 Portugal Avenue 41, León, Spain.

[2] AIT Austrian Institute of Technology GmbH, Center for Health & Environment, Bioresources Unit, 3430 Tulln, Austria.

[3] Area of Microbiology, University School of Agricultural Engineers, Universidad de León, Ponferrada Campus, 24401 Astorga Avenue s/n, Ponferrada, Spain.

[*] **Correspondence:** Pedro Antonio Casquero: pacasl@unileon.es

---

Data S1

This file contains:

Figures S1-S2

Tables S1-S2

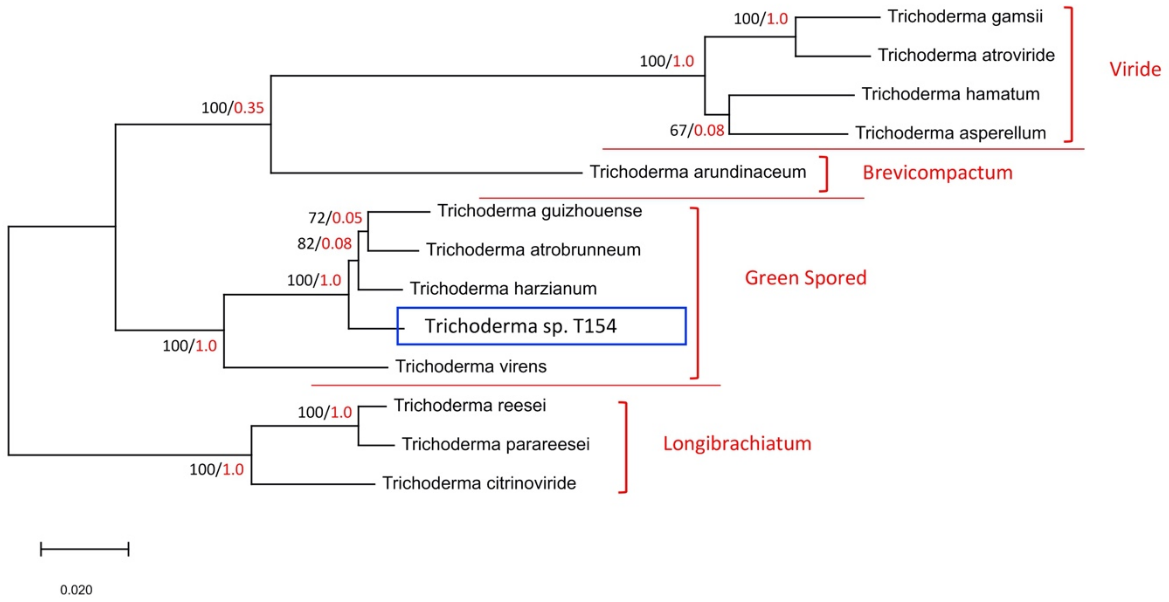

Figure S1. *Trichoderma* species phylogenetic tree. The tree was inferred from alignments of coding nucleotide sequences of *act1, cal1, fas1, lcb2, rpb2, and tef1* housekeeping genes. Trees for these six genes were inferred by two methods, and the results were combined: 1) nucleotide sequences from each gene were aligned separately, the alignments were concatenated, and the resulting concatenated alignment was subjected to maximum likelihood analysis using IQ-tree software 1.6.7 (Nguyen et al., 2014). Branch support was determined by bootstrap analysis after 1000 pseudoreplicates. 2) nucleotide sequences from each housekeeping gene were aligned separately and subjected to maximum likelihood analysis separately. The resulting six trees were used to generate a consensus tree using RAxML software (Stamatakis 2014). Branch support was determined by internode certainly (IC) analysis as implemented in RAxML (Salichos et al., 2014). The tree in the figure was inferred using method 1 but shows branch support values derived from both methods: bootstrap values are shown before the forward slash (i.e. /), and the IC values are shown in red color after the forward slash. At the right size of the tree, there are indicated in red color the names of the *Trichoderma* clades where the different species included in the present study are located. The strain isolated in the present work is squared with a blue line.

Nguyen, L.-T., Schmidt, H.A., von Haeseler, A., Minh, B.Q. (2014) IQ-TREE: A fast and effective stochastic algorithm for estimating maximum likelihood phylogenies. Mol Biol Evol 32:268–274. doi: 10.1093/molbev/msu300

Salichos L, Stamatakis A, Rokas A (2014) Novel information theorybased measures for quantifying incongruence among phylogenetic trees. Mol Biol Evol 31(5):1261–1271. doi: 10.1093/molbev/msu061

Stamatakis, A. (2014). RAxML version 8: a tool for phylogenetic analysis and post-analysis of large phylogenies. Bioinformatics 30: 1312-1313. doi: 10.1093/bioinformatics/btu033.

Figure S2. Single-gene trees inferred from the coding sequences of the six housekeeping genes used in the present analysis. Sequences were aligned using Muscle as implemented in MEGA X (Kumar et al., 2018). Trees were inferred by the maximum likelihood method using ultrafast bootstrap (Minh et al., 2013) as implemented by IQ-Tree (Nguyen et al., 2014). Numbers near branches are bootstrap values based on 1000 pseudoreplicates. All trees are rooted on the species belonging to the clade Longibrachiatum (i.e. *T. reesei, T. parareesei, T. citrinoviride*). Note that these trees were used to generate the consensus tree used for **Figure S1**.

act1

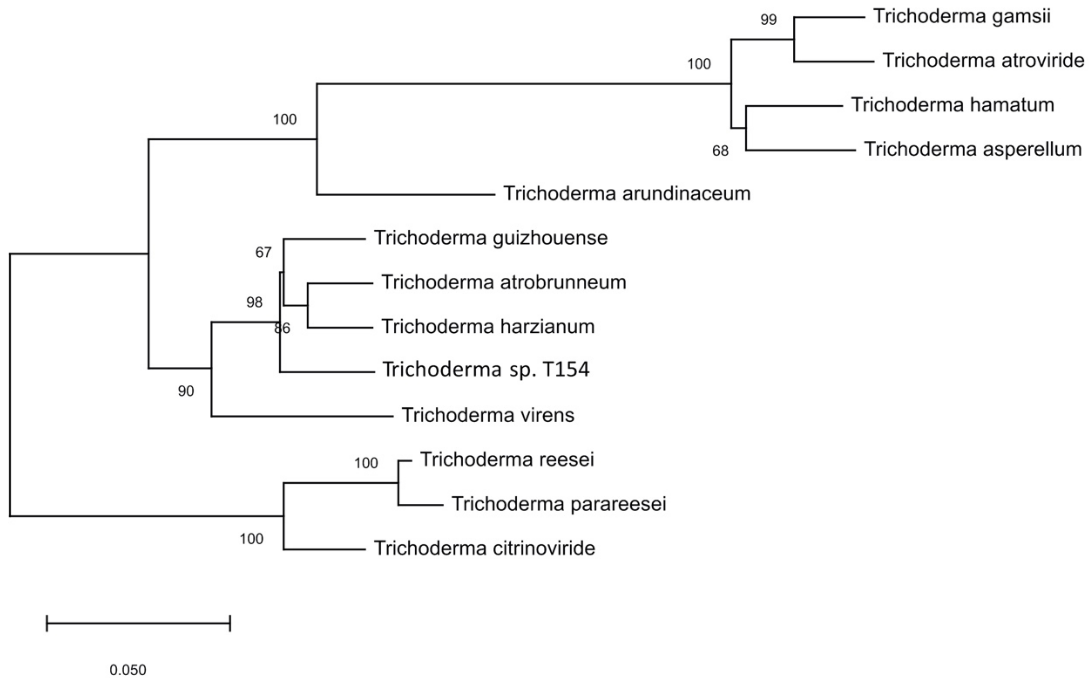

Figure S2 (continued)

Figure S2 (continued)

cal1

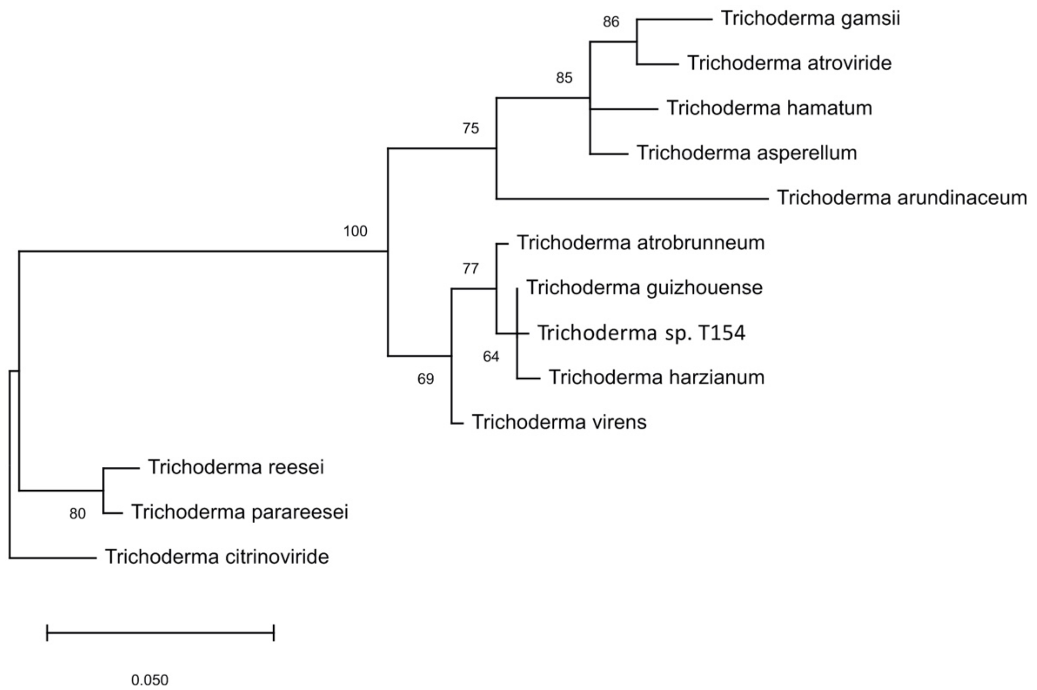

fas1

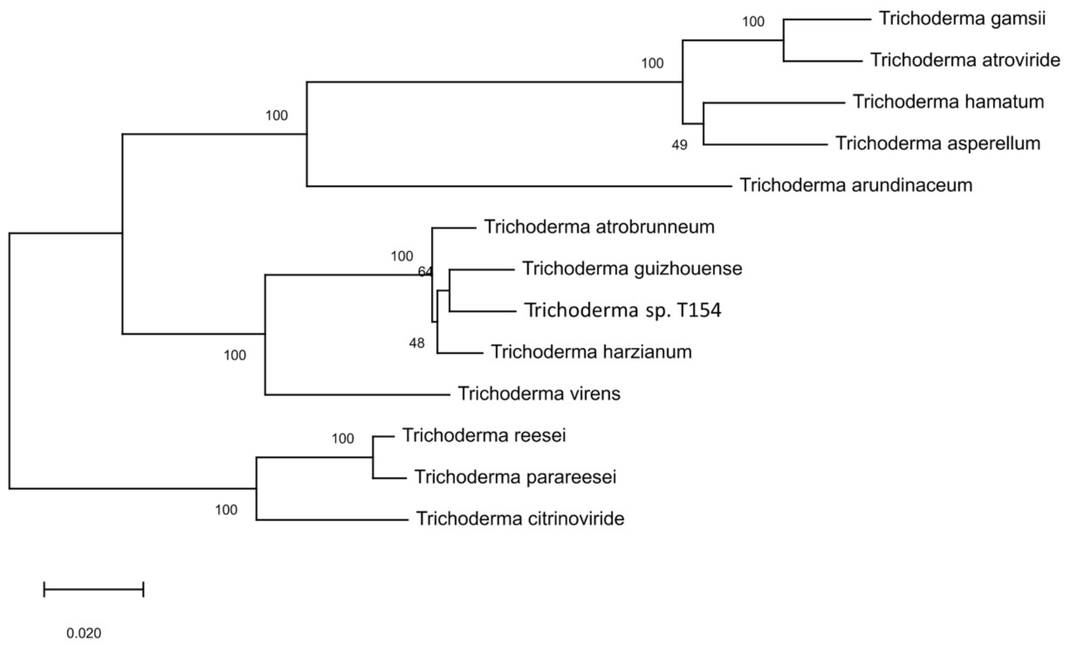

Figure S2 (continued)

lcb2

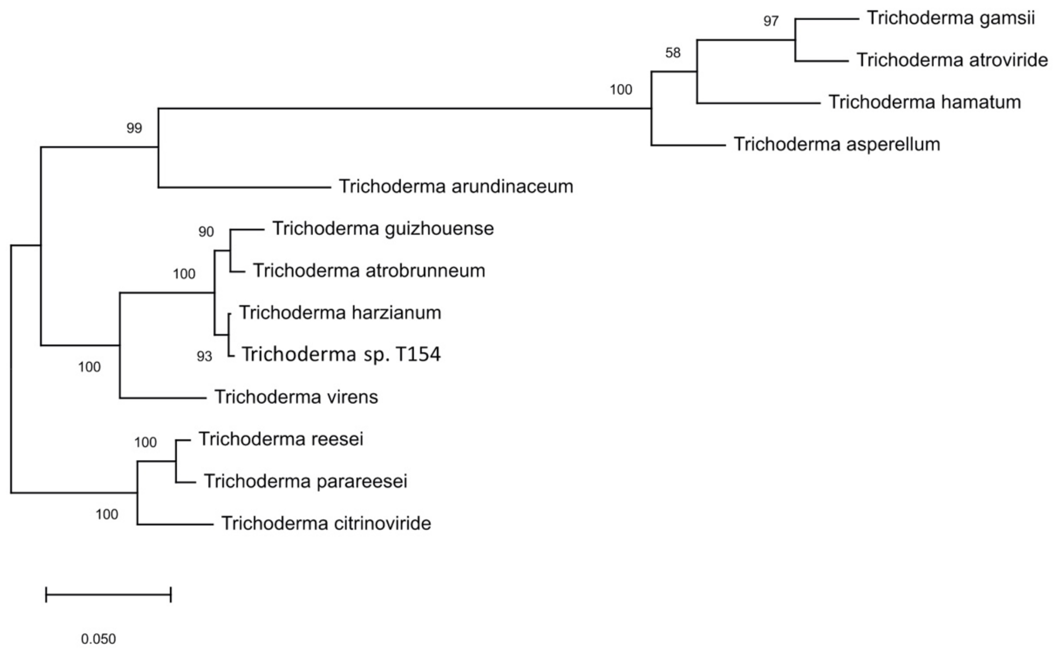

rpb2

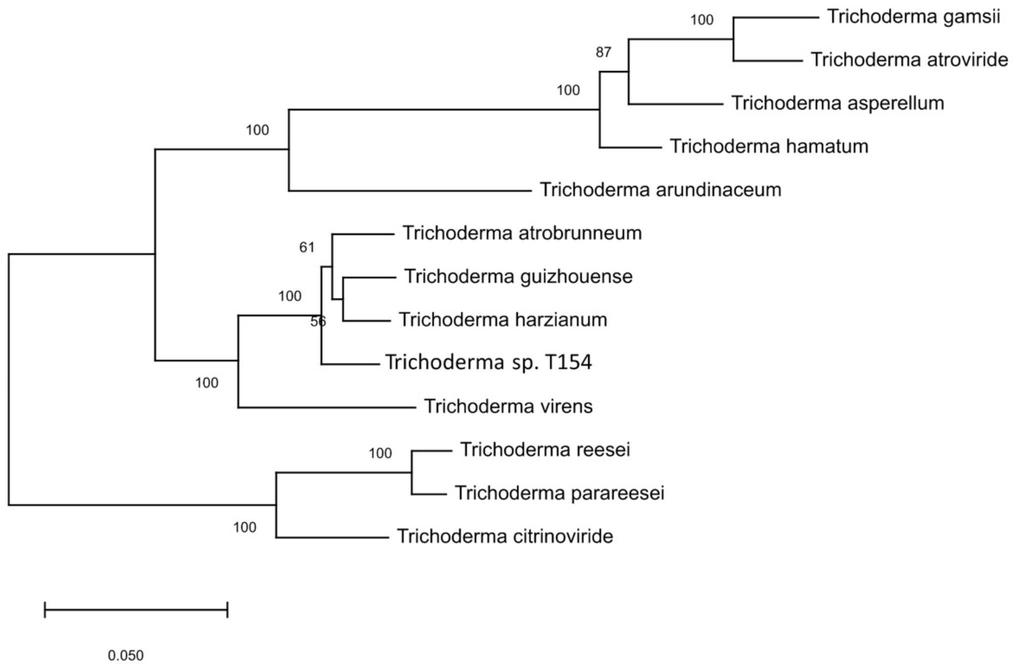

Figure S2 (continued)

tef1

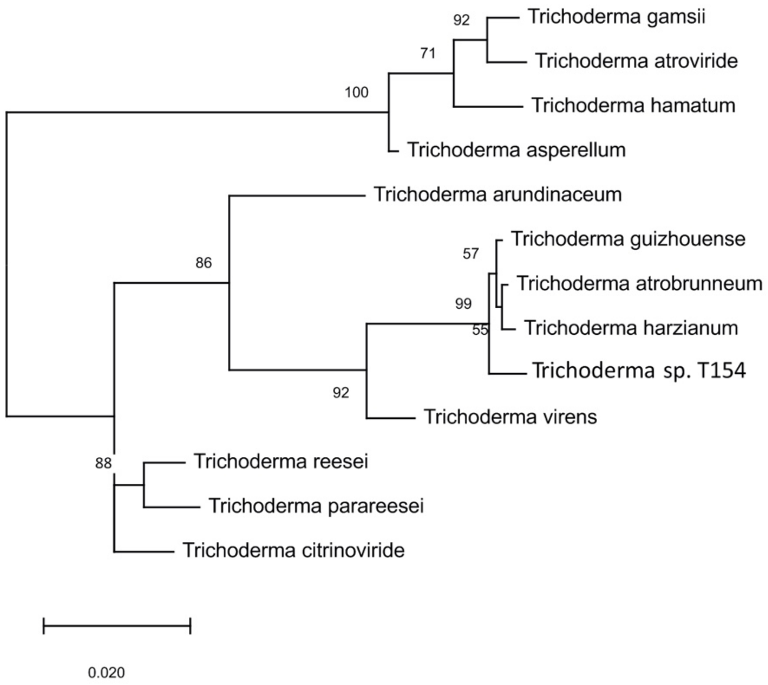

References cited in Figure S2

Kumar, S., Stecher, G., Li, M., Knyaz, C., Tamura, K (2018). MEGA X: Molecular evolutionary genetics analysis across computing platforms. Mol Biol Evol 35: 1547-1549. doi: 10.1093/molbev/msy096.

Minh BQ, Nguyen MA, von Haeseler A (2013) Ultrafast approximation for phylogenetic bootstrap. Mol Biol Evol 30: 1188-1195.

Nguyen, L.-T., Schmidt, H.A., von Haeseler, A., Minh, B.Q. (2014) IQ-TREE: A fast and effective stochastic algorithm for estimating maximum likelihood phylogenies. Mol Biol Evol 32:268–274. doi: 10.1093/molbev/msu300

Table S1. Genomic sequences used in the phylogenetic analyses carried out to identify the *Trichoderma* isolate analyzed in the present work.

**Species**	**GenBank accession number**	**Reference**
Trichoderma asperellum CBS 433.97	GCF_003025105.1	Druzhinina IS, Chenthamara K, Zhang J, Atanasova L, Yang D, Miao Y, Rahimi MJ, Grujic M, Cai F, Pourmehdi S, Salim KA, Pretzer C, Kopchinskiy AG, Henrissat B, Kuo A, Hundley H, Wang M, Aerts A, Salamov A, Lipzen A, LaButti K, Barry K, Grigoriev IV, Shen Q, Kubicek CP<br>Massive lateral transfer of genes encoding plant cell wall-degrading enzymes to the mycoparasitic fungus *Trichoderma* from its plant-associated hosts.<br>*PLoS Genet*. 2018 Apr 9;14(4):e1007322. doi: 10.1371/journal.pgen.1007322.
Trichoderma atroviride IMI 206040	GCF_000171015.1	Kubicek CP, Herrera-Estrella A, Seidl-Seiboth V, et al. Comparative genome sequence analysis underscores mycoparasitism as the ancestral life style of *Trichoderma*.<br>*Genome Biol*. 2011;12(4):R40. doi:10.1186/gb-2011-12-4-r40
Trichoderma gamsii T6085	GCF_001481775.2	Baroncelli R,Zapparata A,Piaggeschi G,Sarrocco S,Vannacci G,null<br>Draft Whole-Genome Sequence of *Trichoderma gamsii* T6085, a Promising Biocontrol Agent of Fusarium Head Blight on Wheat.<br>*Genome Announc*. 2016 Feb 18;4(1):. doi: 10.1128/genomeA.01747-15
Trichoderma hamatum GD12	GCA_000331835.2	Studholme DJ, Harris B, Le Cocq K, Winsbury R, Perera V, Ryder L, Ward JL, Beale MH, Thornton CR, Grant M<br>Investigating the beneficial traits of *Trichoderma hamatum* GD12 for sustainable agriculture-insights from genomics.<br>*Front Plant Sci.* 2013 Jul 30;4:258. doi: 10.3389/fpls.2013.00258.
Trichoderma arundinaceum IBT 40837	GCA_003012105.1	Proctor RH,McCormick SP,Kim HS,Cardoza RE,Stanley AM,Lindo L,Kelly A,Brown DW,Lee T,Vaughan MM,Alexander NJ,Busman M,Gutiérrez S,<br>Evolution of structural diversity of trichothecenes, a family of toxins produced by plant pathogenic and entomopathogenic fungi.<br>*PLoS Pathog*. 2018 Apr;14(4):e1006946. doi: 10.1371/journal.ppat.1006946
Trichoderma virens Gv29-8	GCF_000170995.1	Kubicek CP, Herrera-Estrella A, Seidl-Seiboth V, et al. Comparative genome sequence analysis underscores mycoparasitism as the ancestral life style of *Trichoderma*.<br>*Genome Biol*. 2011;12(4):R40. doi:10.1186/gb-2011-12-4-r40
Trichoderma guizhouense NJAU 4742	GCA_002022785.1	Druzhinina IS, Chenthamara K, Zhang J, Atanasova L, Yang D, Miao Y, Rahimi MJ, Grujic M, Cai F, Pourmehdi S, Salim KA, Pretzer C, Kopchinskiy AG, Henrissat B, Kuo A, Hundley H, Wang M, Aerts A, Salamov A, Lipzen A, LaButti K, Barry K, Grigoriev IV, Shen Q, Kubicek CP<br>Massive lateral transfer of genes encoding plant cell wall-degrading enzymes to the mycoparasitic fungus *Trichoderma* from its plant-associated hosts.<br>*PLoS Genet.* 2018 Apr 9;14(4):e1007322. doi: 10.1371/journal.pgen.1007322.
Trichoderma atrobrunneum ITEM 908	GCA_003439915.1	Fanelli F, Liuzzi VC, Logrieco AF, Altomare C<br>Genomic characterization of *Trichoderma atrobrunneum* (*T. harzianum* species complex) ITEM

		908: insight into the genetic endowment of a multi-target biocontrol strain. *BMC Genomics*. 2018 Sep 11;19(1):662. doi: 10.1186/s12864-018-5049-3.
Trichoderma harzianum CBS 226.95	GCF_003025095.1	Druzhinina IS, Chenthamara K, Zhang J, Atanasova L, Yang D, Miao Y, Rahimi MJ, Grujic M, Cai F, Pourmehdi S, Salim KA, Pretzer C, Kopchinskiy AG, Henrissat B, Kuo A, Hundley H, Wang M, Aerts A, Salamov A, Lipzen A, LaButti K, Barry K, Grigoriev IV, Shen Q, Kubicek CP Massive lateral transfer of genes encoding plant cell wall-degrading enzymes to the mycoparasitic fungus *Trichoderma* from its plant-associated hosts. *PLoS Genet.* 2018 Apr 9;14(4):e1007322. doi: 10.1371/journal.pgen.1007322.
Trichoderma reesei QM6a	GCF_000167675.1	Li WC, Huang CH, Chen CL, Chuang YC, Tung SY, Wang TF Trichoderma reesei complete genome sequence, repeat-induced point mutation, and partitioning of CAZyme gene clusters. *Biotechnol Biofuels.* 2017 Jul 3;10:170. doi: 10.1186/s13068-017-0825-x.
Trichoderma parareesei CBS125925	GCA_001050175.1	Yang D,Pomraning K,Kopchinskiy A,Karimi Aghcheh R,Atanasova L,Chenthamara K,Baker SE,Zhang R,Shen Q,Freitag M,Kubicek CP,Druzhinina IS,null Genome sequence and annotation of *Trichoderma parareese*i, the ancestor of the cellulase producer *Trichoderma reesei*. *Genome Announc.* 2015 Aug 13;3(4):. doi: 10.1128/genomeA.00885-15
Trichoderma citrinoviride TUCIM 6016	GCF_003025115.1	Druzhinina IS, Chenthamara K, Zhang J, Atanasova L, Yang D, Miao Y, Rahimi MJ, Grujic M, Cai F, Pourmehdi S, Salim KA, Pretzer C, Kopchinskiy AG, Henrissat B, Kuo A, Hundley H, Wang M, Aerts A, Salamov A, Lipzen A, LaButti K, Barry K, Grigoriev IV, Shen Q, Kubicek CP Massive lateral transfer of genes encoding plant cell wall-degrading enzymes to the mycoparasitic fungus *Trichoderma* from its plant-associated hosts. *PLoS Genet.* 2018 Apr 9;14(4):e1007322. doi: 10.1371/journal.pgen.1007322.

Table S2. Pairwise distances determined as implemented by MEGA X software (Kumar et al., 2018) from the alignment of the concatenated sequences of the six housekeeping genes used in the present work (see Methods section, and legend to **Figure S1** for more details about these sequences).

[1] *Trichoderma arundinaceum*
[2] *Trichoderma asperellum*
[3] *Trichoderma hamatum*
[4] *Trichoderma atroviride*
[5] *Trichoderma gamsii*
[6] *Trichoderma atrobrunneum*
[7] ***Trichoderma sp.* T154**
[8] *Trichoderma guizhouense*
[9] *Trichoderma harzianum*
[10] *Trichoderma virens*
[11] *Trichoderma citrinoviride*
[12] *Trichoderma parareesei*
[13] *Trichoderma reesei*

[	1	2	3	4	5	6	7	8	9	10	11	12	13]
[1]													
[2]	0.2203												
[3]	0.2238	0.0715											
[4]	0.2237	0.0863	0.0899										
[5]	0.2260	0.0886	0.0921	0.0483									
[6]	0.2083	0.2684	0.2720	0.2719	0.2741								
[7]	0.2105	0.2706	0.2741	0.2740	0.2763	0.0316							
[8]	0.2084	0.2685	0.2720	0.2719	0.2742	0.0339	0.0361						
[9]	0.2071	0.2673	0.2708	0.2707	0.2730	0.0327	0.0348	0.0277					
[10]	0.1923	0.2524	0.2560	0.2559	0.2581	0.0998	0.1020	0.0999	0.0986				
[11]	0.2486	0.3087	0.3122	0.3121	0.3144	0.2165	0.2187	0.2166	0.2153	0.2005			
[12]	0.2569	0.3170	0.3205	0.3204	0.3227	0.2248	0.2270	0.2249	0.2236	0.2088	0.0780		
[13]	0.2570	0.3172	0.3207	0.3206	0.3229	0.2250	0.2271	0.2250	0.2238	0.2090	0.0782	0.0203	

* Species belonging to the clade Green Spored are squared in red in the upper panel, and pairwise distances of *Trichoderma* sp. T154 with the other species of that clade are also squared in red in the lower panel.

Kumar, S., Stecher, G., Li, M., Knyaz, C., Tamura, K (2018). MEGA X: Molecular evolutionary genetics analysis across computing platforms. Mol Biol Evol 35: 1547-1549. doi: 10.1093/molbev/msy096.
