# Supplementary material for: Colonization of Vitis vinifera L. by the Endophyte Trichoderma sp. Strain T154: Biocontrol Activity Against Phaeoacremonium minimum
Source: Front Plant Sci. 2020 Aug 4;11:1170. doi: 10.3389/fpls.2020.01170 (PMC7417607; doi:10.3389/fpls.2020.01170)
Supplement: Supplementary file 2 [file DataSheet_2.pdf]

Niches of colonization of *Vitis vinifera* L. by an endophyte *Trichoderma* sp. T154 strain and its biocontrol activity against *Phaeoacremonium minimum*.

G. Carro-Huerga[1], S. Compant [2], M. Gorfer [2], R.E. Cardoza Silva[1,3], M. Schmoll[2], S. Gutiérrez[1,3], P.A. Casquero[1*]

[1] Research Group of Engineering and Sustainable Agriculture, Natural Resources Institute, Universidad de León, 24071 Portugal Avenue 41, León, Spain.

[2] AIT Austrian Institute of Technology GmbH, Center for Health & Environment, Bioresources Unit, 3430 Tulln, Austria.

[3] Area of Microbiology, University School of Agricultural Engineers, Universidad de León, Ponferrada Campus, 24401 Astorga Avenue s/n, Ponferrada, Spain.

[*] **Correspondence:** Pedro Antonio Casquero: pacasl@unileon.es

---

Data S2

This file contains:

Figures S3-S8

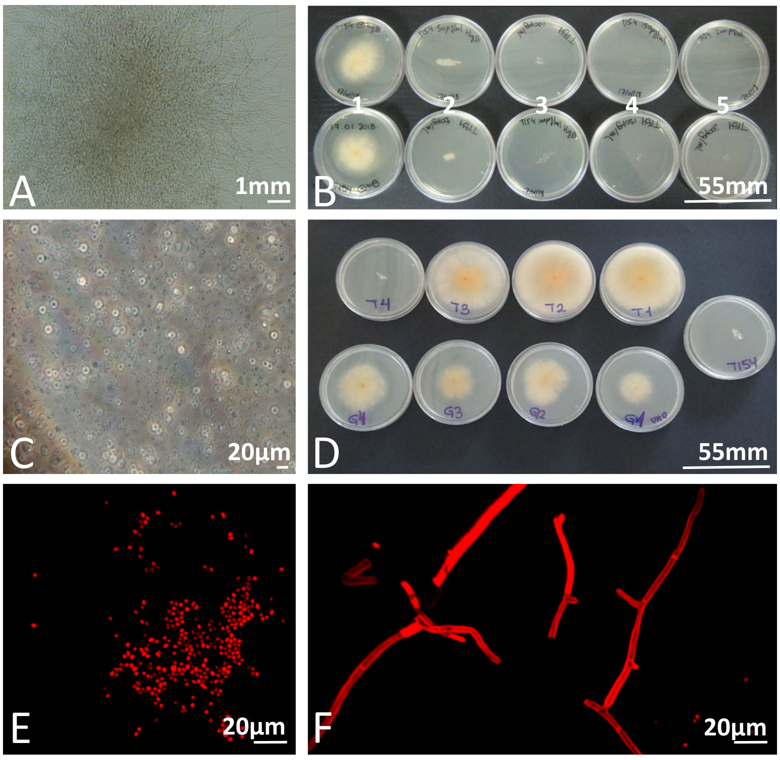

Figure S3 Fungal transformation: (A) Hyphae of *Trichoderma* sp. *T154* strain after 24 hours (10x magnification). **(B)** Assay of hygromycin resistance by duplicate from (1) control to (2) 50 µg mL^{-1}, (3) 100 µg mL^{-1}, (4) 150 µg mL^{-1} and (5) 200 µg mL^{-1}. **(C)** Protoplasts (20X magnification) of *Trichoderma* sp. T154. **(D)** Transformants after two selection rounds on selection medium. Transformed strains with *tdTom* gene T1, T2, T3, T4; strains G1, G2, G3, G4 and T154. **(E)** Spores of *Trichoderma* sp. T154::*tdTom3.* **(F)** Hyphae of *Trichoderma* sp. T154::*tdTom3.*

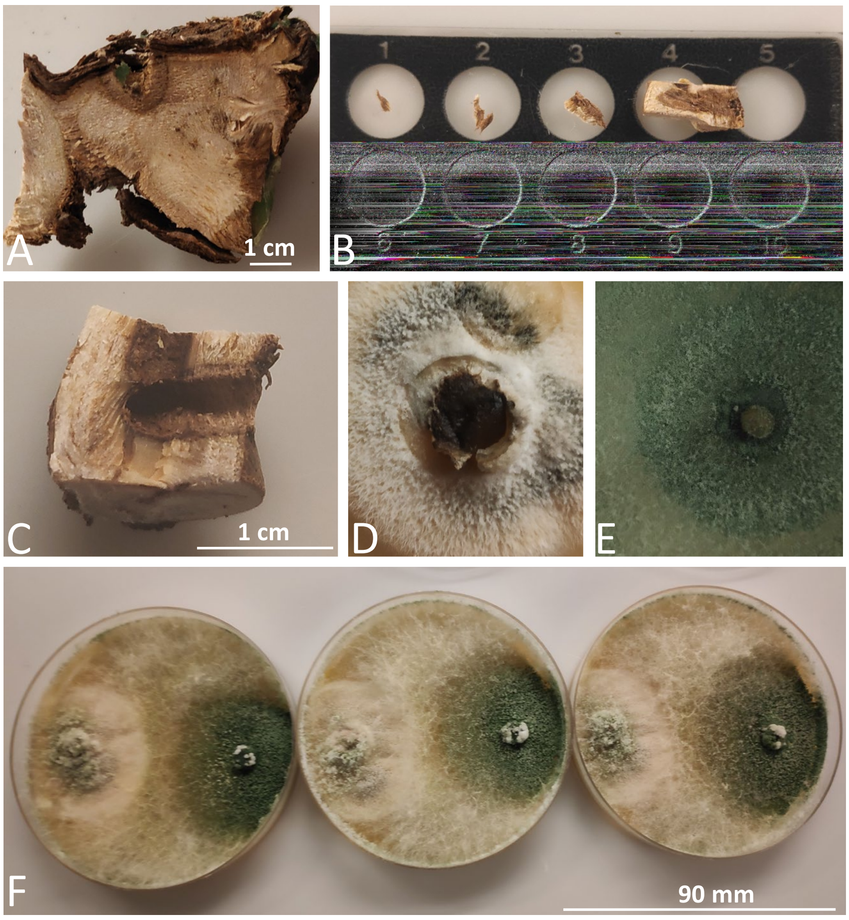

Figure S4 Sampling and evaluation of transformed fungi: **(A)** Overview, transversal section of vine plant. **(B)** Different repetitions of a sample for evaluation. **(C)** Transversal section for evaluation. **(D)** *Phaeoacremonium minimum*::*gfp7* after 14 days in pure culture. **(E)** *Trichoderma* sp. T154::*tdTom3* after 7 days of growth. **(F)** Confrontation assays of *Trichoderma* sp. T154::*tdTom3* (right) vs *P. minimum*::*gfp7* (left) after 10 days of growth.

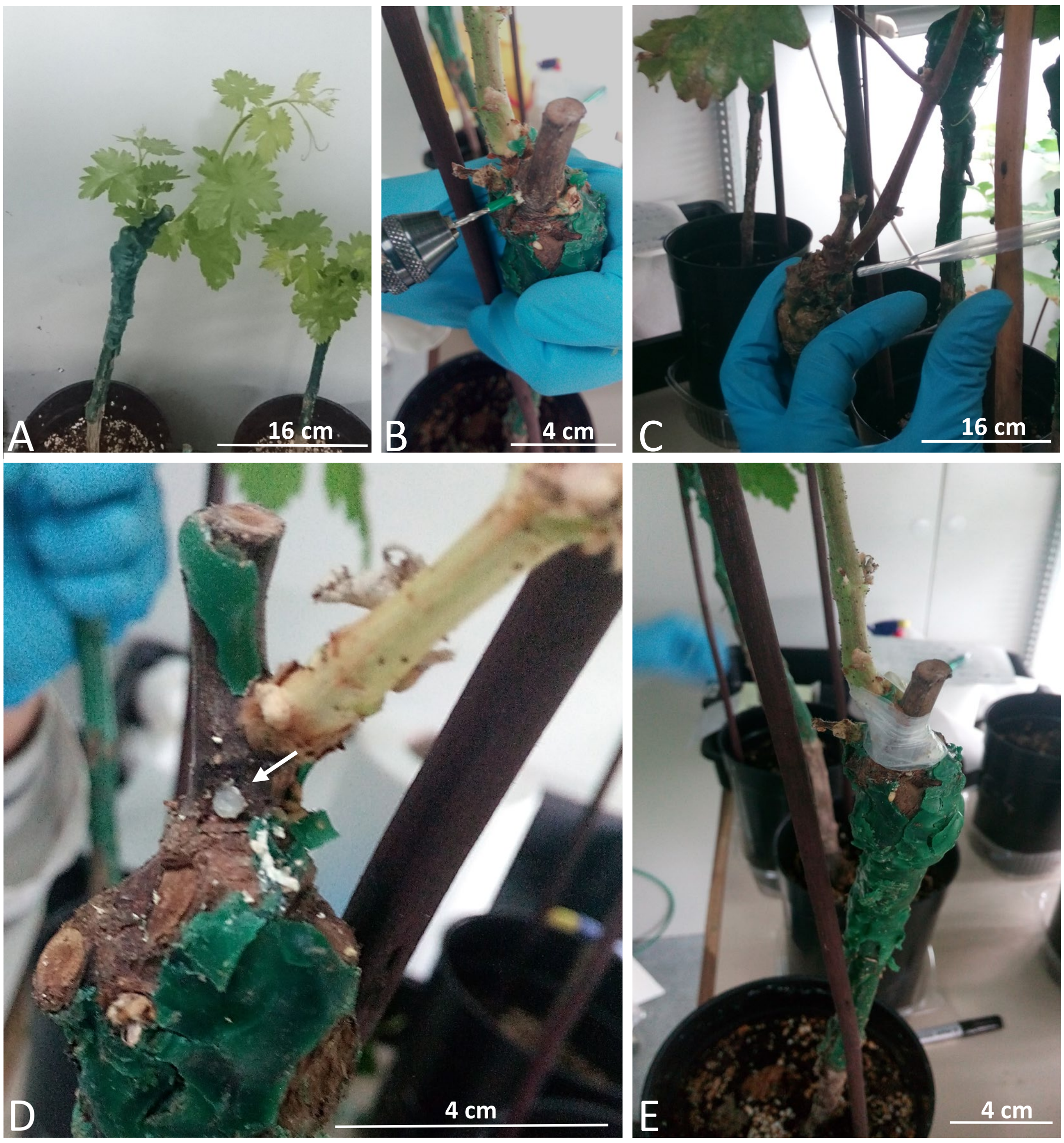

Figure S5 Inoculation: **(A)** One year old grapevine grafted plants of Tempranillo/110 Ritcher were inoculated when at least six leaves were fully developed. **(B)** An internodal damage was performed with a driller over the woody scion Tempranillo cv. **(C)** A plug of fresh mycelium and spores of fungi was inoculated in the pruning injury. **(D)** Point of inoculum after putting plugs. **(E)** The inoculation point was covered with cellophane.

Pure culture after 7 days (CSLM)

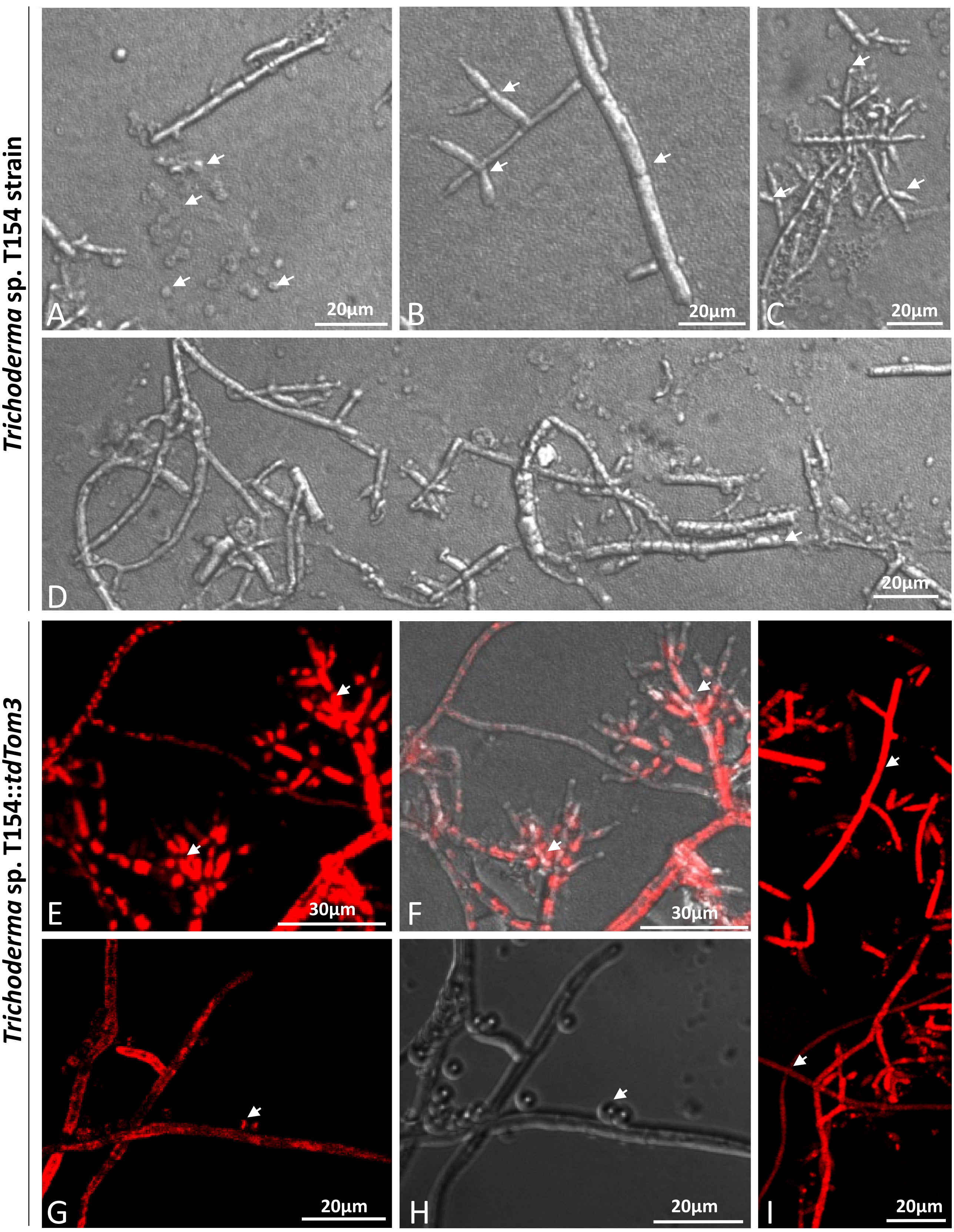

Figure S6 CSLM observation of *Trichoderma* sp. T154 and *Trichoderma* sp. *T154*::*tdTom3* (arrowhead) from a pure culture: (A) Detail of spores of *Trichoderma* sp. T154. **(B)** Detail of *Trichoderma* sp. T154 strain T154's conidiophores. **(C)** General overview of characterisitic *Trichoderma* structures in *Trichoderma* sp. T154. **(D)** General overview of *Trichoderma* sp. T154 strain with mycelium and spores. **(E-F)** Red fluorescent conidiophores and hyphae of *Trichoderma* sp. *T154*::*tdTom3* and also showing conidiophores and hyphae under normal light. **(G-H)** Red fluorescent spores and hyphae of *Trichoderma* sp. T154::*tdTom3* and also showing spores and hyphae of *Trichoderma* sp. T154::*tdTom3* under normal light. **(I)** Red fluorescent hyphae and spores.

Pure culture after 14 days (CSLM)

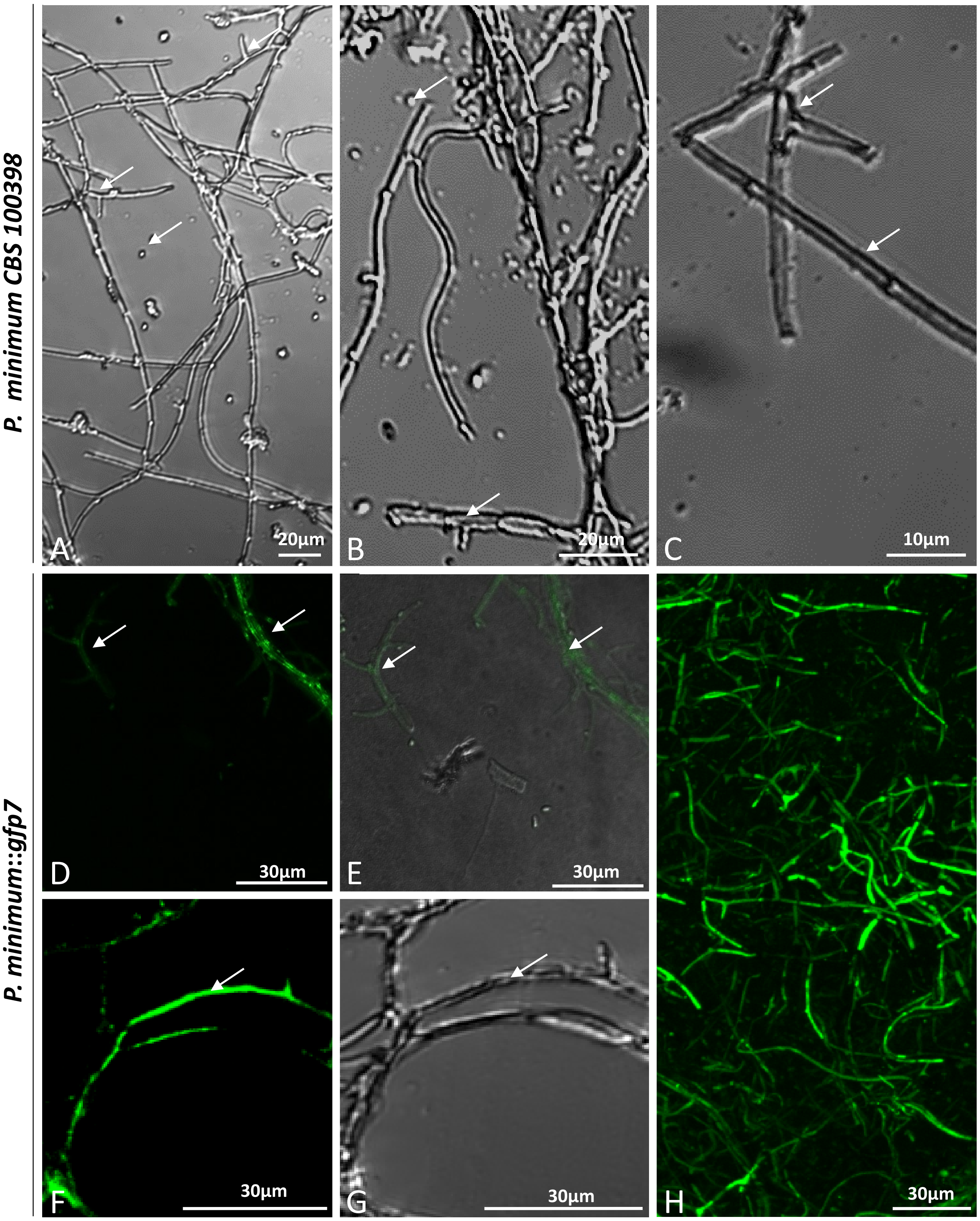

Figure S7 CSLM observation of *P. minimum CBS 100398* and *P. minimum*::*gfp7 from* (arrows) pure cultures: **(A)** General overview of mycelium and spores of *P. minimum* CBS 100398. **(B)** Magnification of hyphae and spores of *P. minimum* CBS 100398. **(C)** Detail of conidiophores of *P. minimum* CBS 100398. **(D-E)** Green fluorescent spores and hyphae of *P. minimum*::*gfp7* and also under normal light, evidencing some lost of signal with the green wavelength. **(F-G)** Green fluorescent Hyphae and conidiophore of *P. minimum*::*gfp7* showing some punctuated fluorescence and also under normal light. **(H)** Mycelium of *P. minimum*::*gfp7* showing a strong green fluorescence.

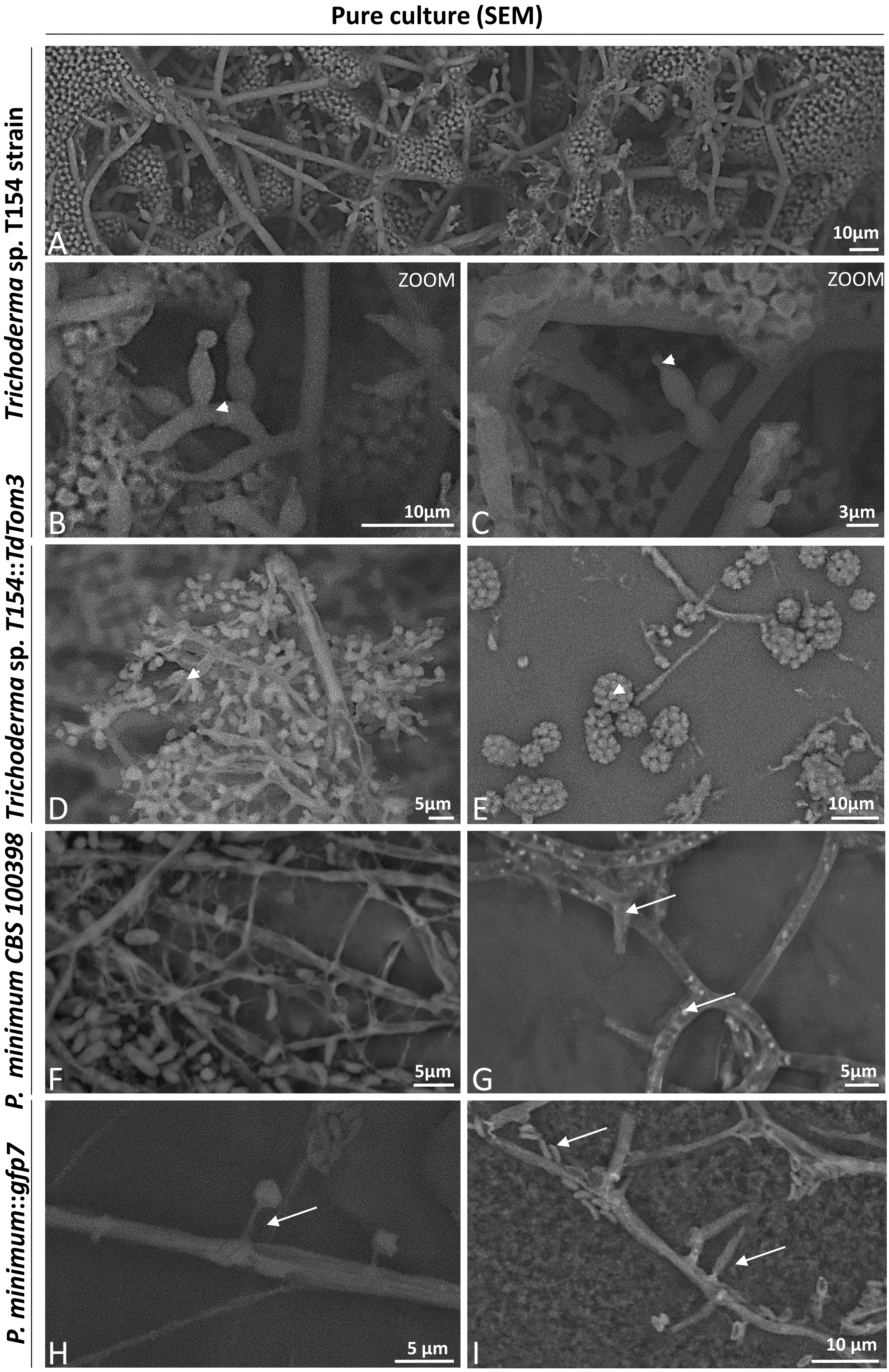

Figure S8 SEM observation of *Trichoderma* sp. T154 (arrowhead), *Trichoderma* sp. *T154*::*tdTom3* (arrowhead)*, P. minimum CBS 100398* (arrows) and *P. minimum*::*gfp7* (arrows), from pure culture: (A) General overview of spores and mycelium of *Trichoderma* sp. T154 after 7 days of growth in pure culture. **(B-C)** Details of conidiophores and phialides of *Trichoderma* sp. T154. **(D)** View of conidiophores and typical structures of *Trichoderma* with spores of *Trichoderma* sp. *T154*::*tdTom3.* **(E)** Phialides full of spores of *Trichoderma* sp. *T154*::*tdTom3.* **(F)** Mycelium and spores of *P. minimum CBS 100398.* **(G)** Phialides of *P. minimum* CBS 100398. **(H)** Hyphae with a phialide full of spores of *P. minimum*::*gfp7.* **(I)** Spores and mycelium of *P. minimum*::*gfp7* .
