# Supplementary material for: Colonization of Vitis vinifera L. by the Endophyte Trichoderma sp. Strain T154: Biocontrol Activity Against Phaeoacremonium minimum
Source: Front Plant Sci. 2020 Aug 4;11:1170. doi: 10.3389/fpls.2020.01170 (PMC7417607; doi:10.3389/fpls.2020.01170)
Supplement: Supplementary file 3 [file DataSheet_3.pdf]

Niches of colonization of *Vitis vinifera* L. by an endophyte *Trichoderma* sp. T154 strain and its biocontrol activity against *Phaeoacremonium minimum*.

G. Carro-Huerga[1], S. Compant [2], M. Gorfer [2], R.E. Cardoza Silva[1,3], M. Schmoll[2], S. Gutiérrez[1,3], P.A. Casquero[1*]

[1] Research Group of Engineering and Sustainable Agriculture, Natural Resources Institute, Universidad de León, 24071 Portugal Avenue 41, León, Spain.

[2] AIT Austrian Institute of Technology GmbH, Center for Health & Environment, Bioresources Unit, 3430 Tulln, Austria.

[3] Area of Microbiology, University School of Agricultural Engineers, Universidad de León, Ponferrada Campus, 24401 Astorga Avenue s/n, Ponferrada, Spain.

[*] **Correspondence:** Pedro Antonio Casquero: pacasl@unileon.es

---

Data S3
This file contains:

Appendix S1-S6

Supplementary Data S3. Sequences retrieved from the genomes analyzed in this work.

Appendix S1. *act1* cds retrieved from the genomes of the 13 species analyzed in the present work

>Trichoderma arundinaceum
ATGGCTCAGCAACCTTTGCCCACGTCGGCTCAGCCAACCGACATCTACGGCGGAGATGAGGTCTCCG
CCCTCGTTCTAGATCCCGGCTACTGCAATACGCGAGCAGGTTTCGCCGGCGAGGATGTCCCCAAATC
AATCCTGCCGTCGTTCTACGGCCACATCACCAGCGACCCCCCTCGAGATCTGTTTGGCGATGAGTGC
ATCATCCCGCGCGGCGACTTCGAGGTCCGCAATTACATGAACAAGGATAGCGTCGTGGAGGATTGG
GATGTGGCAGCTAAGATGTGGGAATTCATGCTGGTTAAGCGCCTGCAGCCCGAGCGACAGACACCT
CCTTCCAAGAACGGGTTGAACGATGACGTGAAAGAGCAGGATGGCGAGGGAGACGTCGCGATGGA
AGAAATTGAGGCACAGGAGAAACCTCTAGAGGAATTCCCTTTGCTGATGACAGAAGCTCCCTGGAA
CTCGCCCAAGGCGCGTGAAAAGGCCATCGAGCTAAGCATGGAGAGCTGGGGAACCCCCGCCTTCTG
GTTGAGTCGGACGCCCGTTCTATCTGCTTTCGCTGCTGGCAAGGCTACCGCCCTTGTCATCGATGTTG
GTGGTGCAAACACCTCCGTTACAGCTATCCACGACGGCATGGTTCTCAAGAGATCTATCCAGCGATC
GCCCGCGGGTGGTCTATGGCTGTCCTCGCAAATCCGCAACATGTGGGAAACCTCCGACCCCAAAGTT
GACCTGATCCCGACGTTTATGGTCGAGAACAAGACTCCTGTGGATGCCCTTGCTCCCGCTCAAGCGC
GACTACGCGAGTTCCCATTCAAGATCCATGACTCTTTCCGAGCTTTTGAAGAAGAGCGGTTGTTGAC
TGAGTATAAAGAGTCCGTTGTTGAGGTTTGGCGTGGCCCTGGAAGATACGGTGCTGCCGGAAACGA
GGAGTACATTAAATCACAGCCTGGCCGTGTATTCGAGATGCCAGATGGCTACAACCAGATGTGGCG
TGAGCAGCGATTCAAGGTGGCTGAGGGAATGTGGGATGAGAATGCTGGATACCCTGTTCCCGAGTC
GGAGCGTCTCACCAAGGCTCAGACCATTCCTGAGCTTATCCGCTCTGCTCTCAATGCTATTGATGTTG
ATCTTCGGGGCAACCTTCTCGCCAACGTTGTCGTCACTGGTAGCACAAGTTTGATCAACGGCTTCAAC
GACCGACTGAACAACGAGCTGACAGCGATGTATCCCGGATTGAAGATCAAGATTCACGCGGCGGGC
TTGTCGAGCGAGCGTAGATTCGGTGCTTGGATCGGTGGCAGCATTCTGGCCAGTCTTGGCACTTTCC
ATCAAATGTGGATATCCAAGAAGGAGTATGAGGAGAACGGAGCCGGTATCGTTGAGAAGCGATGC
AAATAG
>Trichoderma asperellum
ATGGCCCAGCAGCCTTTGCCCACGTCGGCTCAGCCAACCGACATCTACGGTGGAGATGAGGTCTCTG
CACTCGTTCTTGATCCCGGCTACTGCAACACGCGAGCAGGCTTCGCTGGCGAAGATGTCCCCAAGTC
AATCCTGCCATCGTTCTACGGCCATGTCACCAGCGACCCTCCCCGAGATCTATTCGGCGATGAATGC
ATCATCCCACGCAGCGACTTCGAGGTCCGCAACTACATGAATAAGGACAGCGTTGTAGAGGACTGG
GATGTGGCAGCCAAGATGTGGGAATTCATGCTGGTTAAGCGCCTGCAGCCCGAGCGACAGACACCG
CCATCGAAGAATAAGCTGAACGACGACGTGAAGGAGCAGGACGGCGAAGGAGATGTCGCGATGG
AAGAAGTCGAGGCGATGGAGAAGGCTCTAGAGGAGTATCCTTTGTTGATGACAGAAGCTCCGTGG
AACTCGCCCAAGGCACGTGAAAAGGCCATCGAGCTCAGCATGGAGAGCTGGGGAACCCCCGCCTTC
TGGCTAAGCCGGACTCCCGTTCTGTCGTCATTCGCTGCTGGAAAGGCCACTGCTCTTGTCATCGACGT
TGGCGGCGCCAATACCTCCGTTACAGCTATCCATGATGGCATGGTTTTGAAGAGATCTATCCAGCGA
TCACCCGCGGGTGGTTTATGGCTGTCTTCGCAGATCCGCAACATGTGGGAAACTTCCGAGCCGAAA
GTTGACTTGATTCCGACATTCATGGTCGAGAACAAGACTCCTGTGGATGCTCTCGCCCCTGCCCAAG
CGCGACTACGCGAATTTCCCTACAAGATCCACGACTCTTATCGAGCATTTGAAGAAGAGCGGCTCTT
GACCGAGTTCAAGGAGTCTGTCGTTGAGGTTTGGCGTGGGCCTGGAAGATACGGTGCCGCCGGAA
ACGAGGAGTACATCAAATCTCAGCCTGGCCGTGTGTTTGAGATGCCTGATGGCTACAACCAGATGT
GGCGCGAGCAGCGATTCAAGGTAGCTGAGGGCATGTGGGACGAAAACGCTGGATACCCCGTCCCG
GAGTCGGAGCGCCTCACCAAGGCCCAGACCATCCCCGAGCTTATTCGCTCCGCCCTCAACGCTATTG
ACGTTGACCTTCGAGGCAACCTTCTCGCCAACGTTGTTGTTACGGGCAGCACAAGTTTGATCAATGG

CTTCAACGACCGTCTAAATAACGAGTTGACGGCGATGTATCCCGGGCTGAAGATCAAGATCCACGCT
GCAGGTCTGTCAAGCGAGCGTAGATTCGGTGCCTGGATTGGTGGAAGCATTCTTGCTAGCTTGGGA
ACTTTCCACCAAATGTGGATATCCAAAAAGGAGTATGAAGAAAACGGAGTTGGCATTGTCGAGAAG
CGATGCAAATAG
>Trichoderma atrobrunneum
ATGGCCCAGCAGCCTTTGCCCACGTCGGCGCAGCCAACCGACATTTACGGCGGAGATGAGGTCTCT
GCCCTCGTTCTCGACTCCGGCTACTGCAACACGCGAGCAGGTTTTGCCGGCGAAGATGTTCCCAAAT
CAATCCTGCCGTCCTTCTACGGTCACATCACCAGCGATCCCCCGCGAGACCTGTTTGGCGATGAGTG
CATCATCCCGCGCGGCGACTTCGAGGTCCGCAACTACATGAACAAGGACAGCGTCGTGGAGGACTG
GGATGTCGCCGCCAAGATGTGGGAGTTTATGCTGGTTAAGCGCCTGCAGCCCGAGAGACAGACACC
TCCGTCCAAGAATGGATTGAACGACGACGTGAAAGACCAGGACGGCGAGGGCGATGTCGCGATGG
AAGAGATCGAGGGTCAGGAAAAGCCTCTGGAGGAGTTCCCATTGCTGATGACAGAAGCTCCCTGGA
ACTCACCCAAGGCACGTGAAAAGGCCATTGAGCTTAGCATGGAGAGCTGGGGGACGCCGGCCTTTT
GGTTGAGCCGGACACCTGTTCTGTCTGCCTTTGCTGCTGGCAAAGCGACTGCTCTTGTCATCGATATC
GGCGGCGCAAACACCTCCGTCACAGCTATCCACGACGGCATGGTTCTCAAGAGATCTATCCAGCGAT
CACCCGCAGGTGGCCTATGGCTGTCTTCGCAGATCCGCAACATGTGGGAAACCTCTGAGCCCAAGG
TCGACCTGATCCCAACATTCATGGTTGAGAACAAGACTCCTGTGGATGCCCTCGCTCCAGCTCAGGC
ACGGCTACGCGAATTCCCATACAAGATTCACGACTCTTTCCGAGCATTCGAAGAGGAGCGGCTGCTG
ACCGAATTCAAAGAGTCCGTCGTCGAGGTTTGGCGTGGTCCCGGACGGTACGGTGCCGCTGGGAAC
GAGGAGTACATCAAGTCACAGCCTGGCCGTGTATTTGAGATGCCCGATGGCTACAACCAGATGTGG
CGCGAGCAGCGATTCAAGGTGGCCGAGGGAATGTGGGATGAGAACGCCGGATACCCCGTTCCCGA
GGCGGAGCGTCTCACCAAGGCTCAGACCATCCCTGAGCTTATCCGCTCTGCTCTCAACGCCATTGAT
GTTGATCTTCGGGGCAACCTTCTCGCCAATGTTGTCGTTACCGGTAGCACAAGCTTGATCAACGGCTT
CAACGACCGACTCAACAACGAGCTGACAGCCATGTATCCCGGATTGAAGATCAAGATCCACGCGGC
CGGCTTGTCGAGCGAGCGTAGATTCGGTGCCTGGATCGGCGGCAGCATTCTGGCCAGTCTGGGTAC
TTTCCACCAAATGTGGATATCCAAGAAGGAGTATGAGGAAAACGGACCGGGCATTGTTGAGAAGCG
ATGCAAATAG
>Trichoderma atroviride
ATGGCCCAGCAGCCCTTGCCCACGTCGGCTCAGCCAACCGACATCTACGGCGGAGATGAGGTCTCT
GCCCTCGTTCTTGACCCCGGCTACTGCAATACGCGAGCAGGCTTCGCTGGCGAAGATGTCCCCAAGT
CAATTCTGCCGTCATTCTACGGCCATGTCACCAGCGACCCTCCCCGAGATCTATTTGGCGATGAGTGC
ATCATCCCACGAAGCGACTTTGAGGTCCGCAACTACATGAACAAGGACAGCGTCGTAGAGGACTGG
GATGTGGCGGCCAAGATGTGGGAATTCATGCTGGTTAAGCGCCTACAGCCCGAGCGACAGACGCC
GCCATCGAAGAACAAGCTGAACGACGACGTGAAGGAGCAAGACGGCGAGGGAGATGTCGCGATG
GAAGAAGTAGAGACGATGGAGAAGGCCCTAGAAGAGTTCCCTTTGCTGATGACAGAAGCTCCGTG
GAACTCGCCCAAGGCGCGTGAAAAGGCCATCGAGCTCAGCATGGAGAGCTGGGGAACTCCAGCCTT
CTGGCTAAGCCGGACTCCCGTTCTGTCGTCATTCGCCGCTGGAAAGGCCACTGCCCTTGTCATCGAC
GTTGGTGGCGCCAACACCTCCGTGACAGCTATCCATGATGGCATGGTTTTGAAGCGATCTATCCAGC
GATCACCCGCGGGCGGTCTATGGCTGTCTTCGCAGATTCGCAACATGTGGGAGACTTCCGAGCCAA
AAGTTGACTTGGTTCCGACGTTTATGGTTGAGAACAAGTCTCCCGTGGATGCTCTTGCTCCTGCCCAA
GCGCGACTACGCGAGTTCCCTTACAAGATCCACGACTCTTTCCGAGCATTTGAAGAAGAGCGGCTGT
TGACCGAGTTCAAGGAGTCTGTTGTTGAGGTCTGGCGTGGACCTGGAAGATACGGCGCCGCCGGAA
ACGAAGAGTACATCAAATCTCAGCCGGGTCGTGTGTTTGAGATGCCTGATGGCTACAACCAGATGT
GGCGCGAGCAGCGATTCAAGGTCGCTGAGGGCATGTGGGACGAAAACGCTGGATACCCCGTCCCT
GAGTCGGAGCGCCTCACCAAGGCCCAGACCATCCCTGAGCTTATTCGTTCCGCACTCAATGCCATTG
ACGTTGACCTGCGGGGCAACCTCCTCGCCAACGTCGTTGTTACGGGTAGCACAAGTTTGATCAACGG
CTTCAACGATCGTCTAAATAACGAGCTGACAGCGATGTATCCCGGGCTGAAGATCAAGATTCACGCT

GCAGGCCTGTCGAGCGAGCGCAGATTCGGTGCCTGGATTGGTGGAAGTATTCTTGCTAGCTTGGGA
ACTTTCCACCAAATGTGGATATCCAAAAAGGAGTATGAAGAAAACGGAGTTGGCATTGTCGAGAAG
CGATGCAAATAG
>Trichoderma sp. T154
ATGGCCCAGCAGCCTTTGCCCACGTCGGCGCAGCCAACCGACATTTATGGCGGAGATGAGGTCTCT
GCCCTCGTTCTCGACCCCGGCTACTGCAACACGCGAGCAGGCTTTGCTGGCGAAGATGTCCCCAAAT
CAATCCTGCCGTCCTTCTACGGCCACATCACCAGCGATCCCCCGCGAGACCTGTTTGGCGATGAGTG
CATCATCCCGCGCGGCGACTTCGAGGTCCGGAACTACATGAACAAGGACAGCGTCGTGGAGGATTG
GGATGTCGCCGCCAAGATGTGGGAGTTTATGCTGGTTAAGCGCCTGCAGCCTGAGAGACAGACTCC
TCCGTCCAAAAATGGATTGAACGACGATGTGAAAGACCAGGATGGCGAGGGCGATGTCGCGATGG
AGGAAATCGAGGGTCAGGAAAAGCCTCTGGAGGAGTTTCCATTGCTGATGACAGAAGCTCCGTGGA
ACTCGCCCAAAGCGCGTGAAAAGGCCATTGAGCTCAGCATGGAGAGCTGGGGAACCCCGGCCTTTT
GGTTGAGCCGGACACCTGTTCTGTCCGCCTTTGCTGCTGGCAAAGCGACTGCTCTTGTCATCGATATC
GGCGGCGCAAACACCTCCGTCACAGCTATCCACGATGGCATGGTTCTCAAGAGATCTATCCAGCGAT
CACCTGCAGGTGGCCTGTGGCTGTCTTCGCAGATCCGCAACATGTGGGAGACTTCCGAGCCCAAGG
TCGACTTGATCCCGACATTCATGGTTGAGAACAAGACTCCTGTGGATGCCCTCGCCCCCGCTCAGGC
ACGACTACGCGAATTCCCATTCAAGATTCATGACTCCTTCCGAGCATTCGAAGAGGAGCGGCTGCTG
ACCGAGTTCAAAGAGTCTGTTGTCGAGGTTTGGCGTGGTCCCGGACGGTATGGCGCCGCTGGAAAC
GAGGAATACATCAAGTCGCAGCCCGGCCGCGTATTTGAGATGCCCGATGGCTACAACCAGATGTGG
CGCGAGCAGCGATTCAAGGTGGCCGAGGGAATGTGGGACGAGAATGCCGGATACCCCGTTCCCGA
GGCGGAGCGTCTCACCAAGGCTCAGACCATTCCTGAGCTTATCCGCTCTGCTCTCAACGCCATTGAT
GTCGATCTTCGGGGTAACCTTCTCGCCAATGTTGTCGTTACCGGCAGCACAAGCTTGATCAACGGCT
TCAACGACCGACTCAACAACGAGCTGACAGCCATGTATCCCGGACTGAAGATCAAGATTCACGCGG
CCGGCTTGTCGAGCGAGCGTAGATTTGGTGCCTGGATCGGTGGCAGCATCCTGGCCAGTCTGGGCA
CTTTCCATCAAATGTGGATATCCAAGAAGGAGTACGAAGAAAACGGACCGGGCATTGTTGAGAAGC
GATGCAAATAG
>Trichoderma citrinoviride
ATGGCCCAGCAGCCTTTGCCCACGTCGGCGCAGCCAACCGACATTTACGGCGGAGATGAAGTCTCC
GCCCTCGTTCTCGATCCCGGCTACTGCAACACGCGCGCGGGCTACGCCGGCGAAGACGTCCCCAAAT
CCATCCTGCCGTCCTTCTACGGCCACATCACCAGCGACCCTCCGCGAGACCTGTTTGGCGATGAGTG
CATCATCCCGCGCGGCGACTTCGAGGTCCGCAACTACATGAACAAGGACAGCGTCGTGGAGGACTG
GGACGTTGCGGCCAAGCTCTGGGAGTTTATGCTGGTTAAGCGCCTGCAGCCCGAGCGACAGACGCA
TCCGTCCAAGAATGGACTCAACGACGACATCAAGGAGCAGGATGGCGAGGGAGACGTCGCCATGG
AGGAGGTGGAGGGTCTGGAAAAGCCCCTGGAGGAATTCCCCTTGTTGATGTCAGAAGCTCCCTGGA
ACTCGCCCAAGGCGCGTGAAAAGGCCATTGAGCTGAGCATGGAGAGCTGGGGAACACCCGCCTTCT
GGTTGAGCCGGACGCCCGTTCTGGCCTCTTTCGCGGCCGGCAAGGCCACTGCTCTCGTCATTGATGT
CGGCGGCGCAAACACCTCCGTCACAGCCATTCACGACGGCATGGTTCTCAAGCGATCTATCCAGCGA
TCACCCGCCGGCGGCCTGTGGCTGTCGTCGCAGATCCGCAAGATGTGGGAGACGTCCGAGCCCAAG
GTCGACCTGGTGCCGACGTTCATGGTGGAGAACAAGACGCCTGTGGATGCCCTCGCCCCTCCACAA
GCACGACTACGCGAATTTCCCTACAAGATCCACGACTCCTTCCGGGCCTTTGAAGAGGAGCGGGTGC
TGACCGAGTTCAAAGAGTCCGTCGTCGAGGTTTGGCGGGGCCCTGGACGATACGGCGCCGCTGGA
AACGAGGAGTACATCAAGTCACAGCCCGGGCGCGTCTTCGAGATGCCCGACGGCTACAACCAGATG
TGGCGCGAGCAGCGGTTCAAGGTAGCAGAGGGCATGTGGGACGAAAACGCTGGTTACCCTCTTCCC
GAGGAGGAGCGTCTCACAAAGGCGCAGACCATTCCTGAGCTGATCCGTTCTGCGCTCAACGCCGTC
GACGTTGATCTTCGGGGCAACCTTTTGGGCAATGTCGTCGTCACCGGCAGCACGAGCTTGATCAACG
GCTTCAACGACCGATTAAACAACGAGCTGACGGCCATGTATCCCGGATTGAAGATCAAGATCCACGC
CGCGGGCTTGTCAAGCGAGCGCAGATTCGGCGCCTGGATCGGCGGCAGCATCTTGGCCAGCCTGG

GTACCTTCCACCAGATGTGGATATCCAAGAAGGAGTACGAGGAAAACGGAGCTGGCATTGTGGAG
AAGCGATGCAAATAG
>Trichoderma gamsii
ATGGCCCAGCAGCCTTTGCCCACGTCGGCTCAGCCAACCGACATCTACGGCGGAGATGAGGTCTCT
GCCCTCGTTCTTGACCCCGGCTACTGCAATACGCGAGCAGGCTTCGCTGGCGAAGATGTCCCCAAGT
CAATTCTACCATCGTTCTACGGCCATGTCACCAGCGACCCTCCCCGAGATTTATTTGGCGACGAGTGC
ATCATCCCACGAAGCGACTTTGAGGTCCGCAACTACATGAACAAGGACAGCGTCGTAGAGGACTGG
GATGTGGCGGCTAAGATGTGGGAATTCATGCTGGTTAAGCGCCTACAGCCCGAGCGACAGACGCCG
CCATCAAAGAATAAGCTAAACGACGACGTGAAGGAGCAGGATGGCGAAGGAGATGTCGCGATGGA
AGAAGTCGAGACGATGGAGAAGGCTCTAGAAGAGTTCCCCTTGCTGATGACAGAAGCTCCGTGGAA
CTCGCCCAAGGCACGTGAAAAGGCCATCGAGCTCAGCATGGAGAGCTGGGGGACTCCCGCCTTCTG
GCTAAGCCGGACTCCCGTTCTGTCGTCATTCGCCGCTGGAAAAGCTACTGCCCTTGTCATTGACGTTG
GTGGCGCCAATACCTCCGTTACAGCTATCCATGATGGCATGGTTTTGAAGCGATCTATCCAGCGATC
ACCCGCGGGCGGTCTATGGCTGTCTTCGCAGATCCGCAACATGTGGGAAACTTCCGAGCCGAAAGT
GGACTTGGTTCCGACGTTTATGGTTGAGAACAAGTCTCCCGTGGATGCTCTTTCTCCTGCCCAGGCG
CGACTACGCGAGTTCCCTTACAAGATCCACGACTCTTTTCGAACATTTGAAGAAGAGCGGCTGTTGA
CCGAGTTCAAGGAGTCCGTTGTTGAGGTTTGGCGTGGACCTGGAAGATACGGCGCCGCTGGAAACG
AAGAGTACATCAAATCTCAGCCTGGCCGTGTGTTTGAGATGCCTGATGGCTACAACCAGATGTGGC
GCGAGCAGCGATTCAAGGTCGCTGAGGGCATGTGGGACGAAAACGCCGGGTACCCCGTCCCCGAG
TCGGAGCGCCTCACCAAGGCCCAGACCATCCCCGAACTTATCCGTTCCGCCCTCAATGCTATTGACGT
TGACCTGCGGGGCAACCTCCTCGCCAACGTCGTTGTTACGGGAAGCACAAGTTTGATCAACGGCTTC
AACGACCGTCTAAATAACGAGCTGACAGCGATGTACCCTGGGCTGAAGATCAAGATTCACGCTGCA
GGTCTGTCGAGCGAGCGCAGATTCGGTGCCTGGATTGGTGGAAGCATTCTTGCTAGCTTGGGAACT
TTCCACCAAATGTGGATATCCAAAAAGGAGTATGAGGAAAACGGAGTTGGCATTGTCGAGAAGCGA
TGCAAATAG
>Trichoderma guizhouense
ATGGCCCAGCAGCCTTTGCCCACGTCGGCGCAGCCAACCGACATTTACGGCGGAGATGAGGTCTCT
GCCCTCGTTCTCGACCCTGGTTACTGCAACACGCGAGCAGGTTTTGCCGGCGAAGATGTCCCCAAAT
CAATCCTGCCGTCCTTCTACGGTCACATCACCAGCGATCCCCCGCGAGACCTGTTTGGCGATGAGTG
CATCATCCCGCGCGGCGACTTCGAGGTCCGCAACTACATGAACAAGGACAGCGTCGTGGAGGATTG
GGATGTCGCCGCTAAGATGTGGGAGTTTATGCTGGTTAAGCGCCTGCAGCCCGAGAGACAGACACC
CCCGTCCAAGAACGGATTGAATGACGATGTGAAAGACCAGGACGGCGAGGGTGATGTCGCCATGG
AGGAAATCGAGGGTCAGGAAAAGCCTCTGGAGGAGTTCCCATTGCTGATGACGGAAGCTCCCTGGA
ACTCCCCCAAGGCGCGTGAAAAGGCCATTGAGCTCAGCATGGAGAGCTGGGGGACCCCGGCCTTTT
GGTTGAGCCGGACACCTGTTCTGTCTGCTTTTGCTGCTGGCAAAGCGACTGCTCTTGTCATCGATATC
GGTGGCGCAAACACCTCCGTCACAGCTATCCACGACGGCATGGTTCTCAAGAGATCTATCCAGCGAT
CACCCGCAGGTGGCCTGTGGCTGTCCTCGCAGATCCGCAACATGTGGGAAACTTCCGAGCCCAAGG
TCGACTTGATCCCGACATTCATGGTTGAGAACAAGACTCCCGTGGATGCTCTTGCCCCCGCCCAGGC
ACGACTACGCGAATTCCCATACAAGATTCATGACTCCTTCCGAGCATTCGAAGAGGAGCGACTGCTG
ACCGAGTTCAAAGAGTCCGTCGTCGAGGTTTGGCGTGGTCCCGGACGGTATGGTGCCGCAGGAAAC
GAGGAGTACATCAAGTCACAGCCCGGCCGCGTATTTGAGATGCCCGATGGCTACAACCAGATGTGG
CGCGAGCAGCGATTCAAGGTGGCCGAGGGAATGTGGGATGAGAACGCCGGATACCCCGTTCCCGA
GGCAGAGCGTCTCACCAAGGCTCAGACCATTCCTGAGCTTATCCGCTCTGCTCTCAACGCCATCGAT
GTTGATCTTCGGGGCAACCTTCTCGCCAATGTTGTCGTTACTGGCAGCACAAGCTTGATCAACGGCTT
CAACGACCGACTCAACAACGAGCTGACAGCCATGTATCCCGGATTGAAGATCAAGATTCACGCGGC
TGGCTTGTCGAGCGAGCGTAGATTCGGCGCCTGGATTGGTGGCAGCATTCTGGCCAGTCTGGGTAC

TTTTCACCAAATGTGGATATCTAAGAAGGAGTATGAGGAAAACGGACCGGGCATTGTTGAGAAGCG
ATGCAAATAG
>Trichoderma hamatum
ATGGCCCAGCAGCCTTTGCCCACGTCGGCTCAGCCAACTGACATCTACGGTGGAGATGAGGTCTCTG
CCCTCGTTCTTGACCCCGGCTACTGCAATACACGAGCAGGCTTCGCTGGCGAAGATGTCCCCAAGTC
GATCCTGCCCTCGTTCTACGGTCATGTCACCAGCAACCCTCCCCGAGATCTATTTGGCGATGAGTGCA
TCATCCCACGCAGCGACTTCGAGGTCCGCAACTACATGAACAAGGACAGCGTCGTAGAGGATTGGG
ATGTGGCAGCCAAGATGTGGGAATTTATGCTGGTTAAGCGTCTGCAGCCCGAGCGACAGACACCGC
CATCAAAGAATAAGCTGAACGACGACGTGAAGGAGCAGGACGGTGAAGGAGATGTCGCGATGGA
GGAAGTCGAGACGATGGAGAAGGCTCTAGAAGAGTATCCTTTGCTGATGACGGAAGCTCCGTGGA
ACTCGCCCAAGGCACGTGAAAAGGCCATCGAGCTCAGCATGGAGAGCTGGGGAACTCCCGCCTTCT
GGCTGAGCCGGACTCCTGTTCTGTCATCATTCGCCGCTGGAAAGGCTACTGCCCTTGTCATCGACGTT
GGTGGCGCCAATACCTCCGTTACAGCTATCCATGATGGCATGGTTCTGAAGAGATCTATCCAGCGAT
CACCCGCGGGCGGTCTATGGCTGTCTTCGCAGATCCGCAACATGTGGGAAACTTCCGAGCCAAAGG
TTGACTTGATTCCGACGTTTATGGTTGAGAACAAGACTCCTGTGGATGCTCTCTCTCCCGCCCAAGCG
CGACTACGCGAATTCCCCTACAAGATCCACGACTCTTATCGAGCATTTGAAGAAGAGCGGCTGTTGA
CCGAATTCAAGGAGTCTGTCGTTGAGGTTTGGCGTGGACCTGGAAGATACGGCGCCGCCGGAAACG
AAGAGTACATTAAATCTCAACCCGGCCGTGTATTCGAGATGCCTGATGGCTACAACCAGATGTGGCG
CGAGCAGCGATTCAAGGTAGCTGAGGGCATGTGGGACGAAAACGCTGGATACCCCGTCCCCGAGTC
GGAGCGCCTCACCAAGGCCCAGACCATCCCCGAGCTTATTCGCTCCGCACTCAACGCTATTGACGTT
GACCTTCGGGGCAATCTCCTCGCTAACGTTGTTGTTACGGGTAGCACAAGTTTGATCAACGGCTTCA
ACGACCGTCTAAACAACGAGTTGACAGCGATGTATCCCGGACTGAAGATCAAGATTCACGCTGCGG
GTCTGTCGAGCGAGCGCAGATTCGGTGCCTGGATTGGTGGAAGCATTCTCGCTAGCTTGGGAACTTT
CCACCAAATGTGGATATCCAAAAAGGAGTATGAGGAAAACGGAGTTGGCATTGTCGAGAAGCGAT
GCAAATAG
>Trichoderma harzianum
ATGGCCCAGCAGCCTTTGCCCACGTCGGCGCAGCCAACCGACATTTACGGCGGAGATGAGGTCTCT
GCCCTCGTTCTCGACCCCGGCTACTGTAACACGCGAGCAGGTTTTGCCGGCGAAGATGTTCCCAAAT
CAATCCTGCCGTCCTTCTACGGCCACATCACCAGCGATCCCCCGCGAGACCTGTTTGGCGATGAGTG
CATCATCCCGCGCGGCGACTTCGAGGTCCGCAACTACATGAACAAGGACAGCGTTGTGGAGGATTG
GGATGTCGCCGCCAAGATGTGGGAGTTTATGCTGGTTAAGCGCCTGCAGCCCGAGAGACAGACGCC
TCCGTCCAAGAATGGATTGAATGACGACGTGAAAGACCAGGATGGCGAGGGCGATGTCGCGATGG
AGGAGATCGAGGGTCAGGAAAAGCCTCTGGAGGAGTTTCCATTGCTGATGACAGAAGCTCCCTGGA
ACTCGCCCAAGGCGCGTGAAAAGGCTATTGAGCTTAGCATGGAGAGCTGGGGGACCCCGGCCTTTT
GGTTGAGCCGGACACCTGTTCTATCTGCCTTCGCTGCTGGCAAAGCGACGGCTCTTGTCATCGATAT
CGGTGGCGCAAACACCTCCGTCACAGCTATCCACGACGGCATGGTTCTCAAGAGATCTATCCAGCGA
TCACCTGCAGGTGGCCTGTGGCTGTCTTCGCAGATCCGCAACATGTGGGAAACCTCTGAGCCCAAG
GTCGACTTGATCCCGACATTCATGGTTGAGAACAAGACTCCTGTCGATGCCCTCGCCCCCGCCCAGG
CACGACTACGCGAATTCCCATACAAGATTCATGACTCCTTCCGAGCATTCGAAGAGGAGCGACTGCT
CACCGAGTTCAAAGAGTCCGTCGTCGAGGTTTGGCGTGGCCCCGGACGGTATGGTGCCGCTGGGAA
CGAGGAGTACATCAAGTCACAGCCTGGCCGCGTATTTGAGATGCCCGATGGCTACAACCAGATGTG
GCGCGAGCAGCGATTCAAGGTGGCCGAGGGAATGTGGGATGAGAACGCCGGATACCCCGTTCCCG
AGGCGGAGCGTCTCACCAAGGCTCAGACTATCCCTGAGCTTATCCGCTCTGCTCTCAACGCCATTGA
TGTTGATCTTCGGGGCAACCTTCTCGCTAATGTTGTCGTTACTGGTAGCACAAGCTTGATCAACGGCT
TCAACGACCGACTCAACAATGAGCTGACAGCCATGTATCCGGGATTGAAGATCAAGATTCACGCGG
CCGGCTTGTCGAGCGAGCGTAGATTCGGTGCTTGGATCGGCGGCAGCATTCTGGCCAGTCTGGGTA

CTTTCCATCAGATGTGGATATCCAAGAAGGAGTATGAGGAAAACGGACCGGGCATTGTCGAGAAGC
GATGCAAATAG
>Trichoderma parareesei
ATGGCCCAGCAGCCTTTGCCCACGTCGGCGCAGCCAACCGACATTTACGGCGGAGATGAAGTTTCT
GCCCTCGTCCTCGATCCCGGCTACTGCAATACGCGCGCGGGATACGCCGGCGAAGACGTGCCCAAG
TCCATCCTGCCGTCCTTCTACGGCCACATCACCAGCGACCCTCCCCGAGATCTCTTTGGCGATGAGTG
CATCATCCCGCGCGGCGACTTCGAAGTCCGCAACTACATGAACAAGGACAGCGTCGTGGAGGACTG
GGATGTTGCGGCCAAGCTCTGGGAGTTTATGCTGGTTAAGCGCCTGCAGCCCGAGCGGCAGACACA
TCCGTCCAAGAACGGACTGAACGACGACATCAAGGAGCAGGATGGCGAGGGAGACGTCGCCATGG
AGGAGGTTGAGGGTCTGGAGAAGCCCCTGGAAGAGTTTCCCTTGTTGATGTCAGAAGCTCCCTGGA
ACTCGCCCAAGGCGCGTGAAAAGGCCATTGAGCTGAGCATGGAGAGCTGGGGAACGCCCGCCTTCT
GGCTGAGCCGGACACCCGTTCTGGCCTCTTTCGCGGCCGGCAAGGCCACAGCCCTGGTCATCGATGT
CGGCGGCGCAAACACCTCCGTCACGGCCATTCACGACGGCATGGTTCTCAAGCGATCCATCCAGCGA
TCACCCGCTGGCGGTTTGTGGCTGTCGTCGCAAATCCGCAAGATGTGGGAGACGTCCGAGCCCAAG
GTCGACCTGGTGCCCACATTCATGGTTGAAAACAAGACGCCTGTGGATGCGCTCGCCCCTCCACAAG
CACGACTACGCGAATTCCCCTACAAGATCCACGACTCTTTCCGAGCATTTGAAGAGGAGCGGGTGCT
GACTGAGTTCAAAGAGTCCGTCGTCGAAGTTTGGCGTGGCCCTGGACGATACGGCGCCGCTGGAAA
CGAGGAGTACATCAAGTCACAGCCCGGGCGCGTCTTTGAGATGCCCGACGGGTACAACCAGATGTG
GCGCGAGCAGCGGTTCAAGGTGGCGGAGGGAATGTGGGATGAAAACGCTGGGTACCCTCTTCCCG
AGGAGGAGCGTCTCACAAAGGCGCAGACCATTCCCGAGCTTATCCGCTCCGCCCTCAACTCTGTCGA
TGTCGATCTTCGGGGCACTCTTCTCGGCAACGTCGTCGTCACGGGCAGCACGAGCTTGATCAACGGC
TTCAACGACCGATTAAACAACGAGCTGACGGCCATGTATCCCGGCCTGAAGATCAAGATCCACGCCG
CGGGCTTGTCGAGCGAGCGTAGATTCGGCGCCTGGATCGGCGGCAGCATCTTGGCGAGTCTGGGTA
CCTTCCACCAGATGTGGATATCCAAGAAGGAGTACGAGGAGAACGGAGCTGGCATTGTGGAGAAG
CGATGCAAATAG
>Trichoderma reesei
ATGGCCCAGCAGCCTTTGCCCACGTCGGCGCAGCCAACCGACATTTACGGCGGAGATGAAGTCTCT
GCCCTCGTCCTCGATCCCGGCTACTGCAATACGCGCGCGGGCTACGCCGGCGAAGACGTCCCCAAG
TCCATCCTGCCGTCCTTCTACGGCCACATCACCAGCGACCCTCCCCGAGATCTCTTTGGCGATGAGTG
CATCATCCCGCGCGGCGACTTCGAAGTCCGCAACTACATGAACAAGGACAGCGTCGTGGAGGACTG
GGATGTTGCGGCCAAGCTCTGGGAGTTTATGCTGGTTAAGCGCCTGCAGCCCGAGCGGCAGACACA
TCCGTCCAAGAACGGACTGAACGACGACATCAAGGAGCAGGATGGCGAGGGAGACGTCGCCATGG
AGGAGGTTGAGGGTCTGGAGAAGCCGCTGGAAGAGTTTCCCTTGTTGATGTCAGAAGCTCCCTGGA
ACTCGCCCAAGGCGCGTGAAAAGGCCATTGAGCTGAGCATGGAGAGCTGGGGAACGCCCGCCTTCT
GGCTGAGCCGGACGCCCGTTCTGGCCTCTTTCGCGGCCGGCAAGGCCACTGCCCTGGTCATCGATGT
CGGCGGCGCAAACACCTCCGTCACGGCCATTCACGACGGCATGGTTCTCAAGCGATCCATCCAGCGA
TCACCCGCCGGCGGCCTGTGGCTGTCGTCGCAAATCCGCAAGATGTGGGAGACGTCCGAGCCCAAG
GTCGACCTGGTGCCCACATTCATGGTTGAGAACAAGACGCCTGTGGATGCGCTCGCCCCTCCACAAG
CACGACTACGCGAATTTCCCTACAAGATCCACGACTCTTTCCGAGCATTCGAAGAGGAGCGGGTGCT
GACGGAGTTCAAAGAGTCCGTCGTCGAAGTTTGGCGTGGCCCTGGACGATACGGCGCCGCTGGAA
ACGAGGAGTACATCAAGTCACAGCCCGGGCGCGTCTTTGAGATGCCCGACGGGTACAACCAGATGT
GGCGCGAGCAGCGGTTCAAGGTGGCAGAGGGAATGTGGGATGAAAACGCTGGGTACCCTCTTCCC
GAGGAGGAGCGTCTCACAAAGGCGCAGACCATTCCCGAGCTTATCCGCTCCGCCCTCAACTCTGTCG
ACGTCGACCTTCGGGGCACTCTCTTGGGCAACGTCGTCGTCACGGGCAGCACGAGCTTGATCAACG
GCTTCAACGACCGATTAAACAACGAGCTGACGGCCATGTATCCCGGCCTGAAGATCAAGATCCACGC
CGCGGGCTTGTCGAGCGAGCGTAGATTCGGCGCCTGGATCGGCGGCAGCATCCTGGCGAGTCTGG

GTACCTTCCACCAGATGTGGATATCCAAGAAGGAGTACGAGGAAAACGGAGCTGGCATTGTGGAG
AAGCGATGCAAATAG
>Trichoderma virens
ATGGCCCAGCAGCCTTTGCCCACGTCGGCGCAGCCAACCGACATTTACGGCGGAGATGAGGTCTCT
GCCCTCGTTCTCGACCCCGGCTACTGCAACACGCGAGCAGGCTTTGCCGGCGAAGATGTTCCCAAAT
CAATCCTGCCGTCCTTCTATGGCCACATCACCAGCGACCCTCCCCGAGACCTGTTTGGCGATGAATGC
ATCATCCCGCGCGGCGACTTCGAGGTCCGCAACTACATGAACAAGGACAGCGTCGTCGAGGATTGG
GATGTCGCCGCAAAGATGTGGGAGTTTATGCTGGTCAAGCGTCTGCAGCCCGAGAGACAGACACAT
CCGTCCAAGAATGGATTGAACGATGATGTGAAAGATCAGGACGGCGAAGGAGATGTCGCGATGGA
GGAAGTCGAGGGCCAGGAAAAGCCTCTGGAGGAATTCCCTTTGTTGATGACAGAAGCTCCCTGGAA
CTCACCCAAGGCGCGTGAAAAGGCGATTGAGCTTAGCATGGAGAGCTGGGGAACGCCGGCTTTCTG
GTTGAGCCGGACACCTGTGCTGTCCGCCTTTGCTGCTGGCAAGGCTACTGCCCTTGTCATCGATATC
GGCGGCGCAAATACCTCCGTCACAGCTATCCACGACGGCATGGTTCTCAAGAGATCTATTCAGCGAT
CACCCGCGGGTGGCCTTTGGCTGTCTTCTCAAATCCGCAACATGTGGGAAACTTCCGAGCCCAAGGT
CGACTTGATCCCGACTTTCATGGTTGAGAACAAGACTCCCGTGGATGCCCTCGCCCCCGCCCAGGCA
CGACTACGCGAATTCCCGTTCAAGATCCATGACTCCTTCCGAGCGTTCGAGGAGGAGCGGCTGCTGA
CCGAATTCAAAGAGTCAGTCGTCGAGGTTTGGCGTGGTCCTGGACGATATGGTGCCGCTGGAAACG
AGGAATACATCAAATCACAGCCTGGCCGCGTGTTTGAGATGCCCGATGGCTACAACCAGATGTGGC
GTGAGCAGCGATTCAAGGTAGCTGAGGGAATGTGGGATGAGAACGCTGGATACCCCGTTCCAGAG
GCAGAGCGTCTCACCAAAGCTCAGACCATTCCAGAGCTTATCCGTTCTGCTCTCAACGCCATTGATGT
TGATCTTCGGGGCAATCTTCTTGCCAACGTTGTGGTCACAGGCAGCACAAGCTTGATCAACGGCTTC
AACGACCGACTAAACAACGAGCTGACGGCCATGTATCCCGGACTGAAGATCAAGATCCATGCCGCC
GGTTTGTCGAGCGAGCGTAGGTTCGGTGCCTGGATCGGTGGCAGCATTTTGGCCAGTCTGGGTACT
TTCCATCAAATGTGGATATCCAAGAAGGAGTATGAGGAAAACGGACCGGGCATTGTTGAGAAACGA
TGCAAATAG

Appendix S2. *cal1* cds retrieved from the genomes of the 13 species analyzed in the present work

>Trichoderma arundinaceum
ATGGCTGATTCTCTTACCGAAGAGCAGGTCTCCGAGTTCAAGGAGGCCTTCTCTCTCTTTGACAAGG
ACGGTGATGGCCAGATCACCACCAAGGAGCTGGGCACCGTGATGCGCTCTTTGGGCCAGAATCCTT
CCGAGTCAGAGTTGCAGGATATGATCAACGAGGTTGATGCCGACAACAACGGATCTATCGATTTCCC
CGAGTTCCTTACCATGATGGCACGAAAAATGAAGGACACTGATTCCGAGGAGGAGATCCGGGAGG
CGTTCAAGGTCTTCGATCGCGATAACAACGGCTTCATCTCAGCCGCCGAGTTGCGCCATGTCATGAC
GTCTATTGGTGAGAAGCTTACCGACGACGAGGTCGATGAGATGATCCGCGAGGCCGATCAGGACG
GTGATGGCCGAATCGATTACAATGAGTTCGTTCAGCTTATGATGCAAAAATAA
>Trichoderma asperellum
ATGGCTGATTCTCTTACCGAAGAGCAGGTCTCTGAGTTCAAGGAGGCCTTCTCTCTCTTTGACAAGG
ACGGCGATGGCCAGATCACCACCAAGGAGCTGGGCACTGTCATGCGCTCTTTGGGCCAGAACCCCT
CCGAGTCAGAGCTGCAGGACATGATCAACGAGGTTGATGCCGACAACAATGGATCCATCGATTTCC
CTGAGTTCCTTACCATGATGGCACGAAAAATGAAGGACACTGATTCCGAGGAGGAGATCCGAGAGG
CTTTCAAGGTCTTCGATCGCGATAACAACGGCTTCATCTCAGCTGCCGAGCTGCGCCATGTCATGAC
ATCTATTGGCGAGAAGCTCACCGACGACGAGGTCGATGAGATGATTCGTGAGGCTGATCAGGACG
GTGATGGCCGAATTGATTACAACGAGTTTGTACAGCTTATGATGCAAAAATAA
>Trichoderma atrobrunneum
ATGGCTGATTCTCTTACCGAAGAGCAGGTCTCTGAGTTCAAGGAGGCCTTCTCCCTCTTTGACAAGG
ACGGTGATGGCCAGATCACCACCAAGGAGCTGGGCACCGTGATGCGCTCTCTCGGCCAGAACCCCT
CCGAGTCAGAGCTTCAGGACATGATCAACGAGGTCGATGCCGACAACAACGGATCTATCGATTTCCC
TGAATTCCTGACCATGATGGCACGAAAGATGAAGGACACTGATTCCGAGGAGGAGATCCGAGAGG
CATTCAAGGTCTTTGACCGCGACAACAACGGCTTCATCTCAGCCGCTGAGCTGCGCCATGTCATGAC
ATCCATCGGCGAGAAGCTCACCGACGACGAGGTCGATGAGATGATTCGCGAGGCCGATCAGGACG
GTGATGGCCGAATTGATTACAATGAGTTTGTCCAGCTTATGATGCAAAAATAA
>Trichoderma atroviride
ATGGCTGATTCTCTTACCGAAGAGCAGGTCTCTGAGTTCAAGGAGGCCTTCTCTCTCTTTGACAAGG
ACGGCGATGGCCAGATCACCACCAAGGAGCTGGGCACTGTCATGCGCTCTTTGGGACAGAACCCCT
CCGAGTCAGAGCTGCAGGACATGATCAACGAGGTCGATGCCGACAACAACGGATCCATCGATTTCC
CTGAGTTCCTTACCATGATGGCACGAAAAATGAAGGACACTGATTCCGAGGAGGAGATCCGAGAGG
CTTTCAAGGTCTTTGACCGTGATAACAACGGCTTCATCTCAGCTGCTGAGCTGCGCCATGTCATGACA
TCTATTGGCGAGAAGCTCACCGACGACGAGGTCGATGAGATGATTCGCGAGGCTGATCAGGATGGT
GATGGCCGAATTGATTACAACGAGTTTGTTCAGCTCATGATGCAAAAATAA
>Trichoderma sp. T154
ATGGCTGATTCTCTTACCGAAGAGCAGGTCTCTGAGTTCAAGGAGGCCTTCTCCCTCTTTGACAAGG
ACGGTGATGGCCAGATCACCACCAAGGAGCTGGGCACCGTGATGCGCTCCCTCGGCCAGAACCCCT
CCGAGTCAGAGCTGCAGGACATGATTAACGAGGTCGATGCCGACAACAACGGATCTATCGATTTCC
CCGAATTCCTGACCATGATGGCACGAAAGATGAAGGACACTGATTCCGAGGAGGAGATCCGAGAG
GCATTCAAGGTCTTTGACCGCGACAACAACGGCTTCATCTCAGCCGCTGAGCTGCGCCATGTCATGA
CATCCATCGGCGAGAAGCTCACCGACGACGAGGTCGATGAGATGATTCGCGAGGCCGATCAGGAC
GGTGATGGCCGAATTGATTACAATGAGTTTGTCCAGCTTATGATGCAAAAATAA
>Trichoderma citrinoviride
ATGGCTGATTCTCTCACCGAAGAGCAGGTCTCCGAGTTTAAGGAGGCCTTCTCTCTCTTTGACAAGG
ACGGCGATGGCCAGATCACCACCAAGGAGCTGGGCACCGTGATGCGCTCTCTGGGCCAGAACCCTT
CCGAGTCGGAGCTGCAGGACATGATCAACGAGGTCGATGCCGACAACAACGGCTCCATCGACTTCC

CTGAATTTCTCACCATGATGGCACGGAAAATGAAGGACACCGATTCCGAGGAGGAGATCCGGGAG
GCATTCAAGGTCTTTGACCGTGACAACAACGGCTTCATCTCCGCTGCCGAGCTGCGCCATGTCATGA
CTTCCATCGGCGAGAAGCTGACCGACGACGAGGTCGATGAGATGATTCGCGAGGCCGACCAGGAC
GGCGACGGCCGAATCGACTACAACGAGTTTGTCCAGCTCATGATGCAGAAATAA
>Trichoderma gamsii
ATGGCTGATTCACTTACCGAAGAGCAGGTCTCTGAGTTCAAGGAGGCCTTCTCTCTCTTTGACAAGG
ACGGTGATGGCCAGATCACCACCAAGGAGCTCGGCACTGTCATGCGCTCTTTGGGACAGAACCCCT
CCGAGTCAGAGCTGCAGGACATGATTAACGAGGTCGATGCCGACAACAACGGATCCATCGATTTCC
CTGAGTTCCTTACCATGATGGCACGAAAAATGAAGGACACCGATTCCGAGGAGGAGATCCGAGAGG
CTTTCAAGGTCTTCGACCGCGATAACAACGGCTTCATCTCAGCTGCCGAGCTGCGCCATGTCATGAC
ATCTATTGGCGAGAAGCTCACCGACGACGAGGTCGATGAGATGATTCGTGAGGCCGATCAGGATG
GTGATGGCCGAATTGATTACAACGAGTTTGTTCAGCTCATGATGCAAAAATAA
>Trichoderma guizhouense
ATGGCTGATTCTCTTACCGAAGAGCAGGTCTCTGAGTTCAAGGAGGCCTTCTCCCTCTTTGACAAGG
ACGGTGATGGCCAGATCACCACCAAGGAGCTGGGCACCGTGATGCGCTCCCTCGGCCAGAACCCCT
CCGAGTCAGAGCTGCAGGACATGATCAACGAGGTCGATGCCGACAACAACGGATCTATCGATTTCC
CCGAATTCCTGACCATGATGGCACGAAAGATGAAGGACACTGATTCCGAGGAGGAGATCCGAGAG
GCATTCAAGGTCTTTGACCGCGACAACAACGGCTTCATCTCAGCCGCTGAGCTGCGCCATGTCATGA
CATCCATCGGCGAGAAGCTCACCGACGACGAGGTCGATGAGATGATTCGCGAGGCCGATCAGGAC
GGTGATGGCCGAATTGATTACAATGAGTTTGTCCAGCTTATGATGCAAAAATAA
>Trichoderma hamatum
ATGGCTGATTCTCTTACCGAAGAGCAGGTCTCTGAGTTCAAGGAGGCCTTCTCTCTCTTTGACAAGG
ACGGCGATGGCCAGATCACCACCAAGGAGCTGGGCACTGTCATGCGCTCTTTGGGCCAGAACCCCT
CCGAGTCAGAGCTGCAGGACATGATCAACGAGGTTGATGCCGACAACAACGGATCCATCGATTTCC
CTGAGTTCCTTACCATGATGGCACGAAAAATGAAGGACACTGATTCCGAGGAGGAGATCCGAGAGG
CTTTCAAGGTCTTCGACCGTGACAACAACGGTTTCATCTCAGCTGCCGAGCTGCGCCATGTCATGACT
TCTATTGGCGAGAAGCTCACCGACGACGAGGTCGACGAGATGATTCGTGAGGCTGATCAGGACGGT
GATGGCCGAATTGATTACAACGAGTTTGTTCAGCTGATGATGCAAAAATAA
>Trichoderma harzianum
ATGGCTGATTCTCTTACCGAAGAGCAGGTCTCTGAGTTCAAGGAGGCCTTCTCCCTCTTTGACAAGG
ACGGTGATGGCCAGATCACCACCAAGGAGCTGGGCACCGTGATGCGCTCCCTCGGCCAGAACCCCT
CCGAGTCAGAGCTCCAGGACATGATCAACGAGGTCGATGCCGACAACAACGGATCTATCGATTTCCC
CGAATTCCTGACCATGATGGCACGAAAGATGAAGGACACTGATTCCGAGGAGGAGATCCGAGAGG
CATTCAAGGTCTTTGACCGCGACAACAACGGCTTCATCTCAGCCGCTGAGCTGCGCCATGTCATGAC
ATCCATCGGCGAGAAGCTCACCGACGACGAGGTCGATGAGATGATTCGCGAGGCCGATCAGGATG
GTGATGGCCGAATTGATTACAATGAGTTTGTCCAGCTTATGATGCAAAAATAA
>Trichoderma parareesei
ATGGCTGATTCTCTTACCGAAGAGCAGGTCTCTGAGTTCAAGGAGGCCTTCTCCCTCTTTGACAAGG
ACGGCGATGGCCAGATCACCACCAAGGAGCTGGGCACCGTGATGCGCTCTCTCGGCCAGAACCCTT
CCGAGTCGGAGCTGCAGGACATGATCAACGAGGTTGACGCCGACAACAACGGTTCCATCGACTTCC
CTGAATTTCTCACCATGATGGCACGAAAAATGAAGGACACCGACTCCGAGGAGGAGATCCGGGAG
GCATTCAAGGTCTTTGACCGCGACAACAACGGCTTCATCTCTGCTGCTGAGCTGCGCCACGTCATGA
CCTCCATCGGCGAGAAGCTGACCGACGACGAGGTCGATGAGATGATTCGCGAGGCCGACCAGGAC
GGCGATGGCCGAATCGACTACAACGAGTTTGTCCAGCTCATGATGCAGAAATAA
>Trichoderma reesei
ATGGCTGATTCTCTTACCGAAGAGCAGGTCTCTGAGTTCAAGGAGGCCTTCTCCCTCTTTGACAAGG
ACGGCGATGGCCAGATCACCACCAAGGAGTTGGGCACCGTGATGCGCTCTCTCGGCCAGAACCCTT

CCGAGTCGGAGCTGCAGGACATGATCAACGAGGTTGACGCCGACAACAACGGCTCCATCGACTTCC
CTGAATTTCTCACCATGATGGCACGAAAAATGAAGGACACCGATTCCGAGGAGGAGATCCGGGAGG
CATTCAAGGTCTTTGACCGCGACAACAACGGCTTCATCTCTGCTGCTGAGCTGCGCCACGTCATGAC
CTCCATCGGCGAGAAGCTGACCGACGACGAGGTCGACGAGATGATCCGCGAGGCCGACCAGGACG
GCGATGGCCGAATCGACTACAACGAGTTTGTCCAGCTCATGATGCAGAAATAA
>Trichoderma virens
ATGGCTGATTCTCTTACCGAAGAGCAGGTCTCTGAGTTCAAGGAGGCCTTCTCTCTCTTTGACAAGG
ACGGTGATGGCCAGATCACCACCAAGGAGCTGGGCACCGTGATGCGCTCTCTGGGCCAGAACCCCT
CCGAGTCAGAGCTGCAGGACATGATCAACGAGGTCGATGCCGACAACAACGGATCTATCGATTTCC
CTGAATTCCTGACCATGATGGCACGAAAGATGAAGGACACTGATTCCGAGGAGGAGATCCGAGAG
GCATTCAAGGTCTTTGACCGCGACAACAACGGCTTCATCTCAGCCGCCGAGCTGCGCCATGTCATGA
CATCCATCGGCGAGAAGCTCACCGACGATGAGGTCGATGAGATGATTCGTGAGGCCGATCAGGACG
GTGATGGCCGAATTGATTACAATGAGTTTGTCCAGCTTATGATGCAAAAATAA

Appendix S3. *fas1* cds retrieved from the genomes of the 13 species analyzed in the present work

>Trichoderma arundinaceum
ATGCGTCCCGAAGTCGAGCAAGAGCTCGCCCACACGCTCCTCGTTGAGCTTCTGGCATATCAATTCG
CCTCTCCCGTGAGGTGGATTGAGACCCAGGATGTGTTCCTGGCAGAGAAGACGGCCGAGCGCATTG
TTGAAGTCGGCCCCGCAGATACCCTTGGAGTCATGGCCAAGCGCACATTGGCGTCCAAGTATGAGG
CCTACGATGCCGCCAAGTCGGTTCAGCGACAGATCCTTTGCTACAACAAAGATGCCAAGGAGATTTA
TTACGATGTCGACCCTGTTGAAGAAGAGCCCGAGCCCGCCGCCGCCAGCTCATCCAGTGCCCCCTCC
AGTCAGCCCGCTGCTGCTGCAGCTGCCCCGCCAGTTGCTGCCGCTCCGGCTCCCAGCGCAGGGCCTG
CGGCTCAGGTTCCCGATCAGCCTGTGCAAGCTATCGACATTGTGCACACATTGGTTGCGCAAAAGCT
CAAGAAGTCTCTTGCTGATGTGCCACTAAGCAAGGCCATCAAGGATCTTGTTGGAGGCAAGTCCACT
CTCCAAAATGAGATCCTCGGTGATCTTGGAAAGGAATTCGGCTCAACGCCTGAGAAGCCCGAGGAT
ACACCGCTCGACGAGCTCAGTGCTGCCATGGGTGCTACTTTCGATGGTAACCTGGGCAAGCACTCAA
CATCACTCATCGCCAGATTGATCTCTTCAAAGATGCCTGGTGGTTTCAACATCACAGTGGCAAGAAA
ATACCTGGAATCAAGATGGGGCCTTGGTCCCGGCAGACAAGATGGAGCATTGCTGCTGGCCTTGAC
CATGGAGCCTCCTGCTCGTCTAGGAAATGAAGGTGAAGCCAAGGGATACCTTGACGGGATCGTCCA
GAAATACGCTGCAAACGCTGGTATCAGCCTGACAACCGCTGCTTCCTCTGGTGGCTCTGAAGGTGGT
GCGGGAGGCATGATGATGGACCCCGCCGCCATTGACGCTCTCACCAAGGACCAACGAGCCCTGTTC
AAGCAGCAGTTGGAGCTCTTTGCCAGATATCTCAAGATGGACCTTCGGGCAGGTGACAGGGCGCAC
ATTGAATCCCAAAAATCAGAAAAGGTCCTGCAGGCCCAGCTTGATTTGTGGACTGCAGAACATGGT
GACTTCTACGCCTCCGGCATTGAGCCTGTCTTCAGCCCATTGAAGGCTAGGTCATACGACTCATCTTG
GAACTGGGCTCGTCAAGATGCACTCACCATGTTCTATGACATCATCTTCGGCAGACTCAAGACCGTC
GACCGCGAAATTGTCAGCCAGTGCATACGCATTATGAACAAGTCAAACCCCAAGCTCCTCGAGTTTA
TGCAGTACCACATCGATAACTGCCCAACCGAGCGTGGCGAGACCTACCAGCTCGCCAAGGAACTTG
GCCAGCAGCTCATCGAGAACTGCAAGGAGGTGCTGGATCTGTCTCCTGTCTACAAGGACGTTGCCG
TGCCCACCGGTCCTCGCACCACCGTGGACGCCCGCGGCAACCTCAACTACGAGGAAGTTCCTCGTGC
CAGCTGCAGGAAGCTTGAGCATTATGTTCAGCAGATGGCCGAGGGCGGAAAGATTTCCGAGTATGG
CAACCGCACCAAGGTACAGAACGATCTCCAGCGCATCTATAAGTTGATCAAGCAGCAGCACAAGAT
GTCCAAGACTTCTCAGCTCGAGATCAAGAGCCTGTACGGTGATGTTCTGCGCTCTCTGGCCATGAAC
GAGAGCCAGATCATCCCCAAGGAGAACGGCAAGGGCAGAAAGCCTGGCCTGAAGGGCACAAGCCT
TAACAAGGGCAGGGTCGAGACGATCCCATTCCTTCACCTCAAGAGAAAAACTCTACACGGCTGGGA
TTACAGCAAGAAGCTCACTGCTGTCTACCTCAACTCTCTCGAAGAGGCTGCCAAGGATGGTGTCACC
TTCCAGGACAAGTATGTCTTGATGACTGGCGCTGGTGCCGGCTCCATTGGTGCCGAGGTTCTGCAGG
GCCTCGTCAGCGGAGGTGCCAAGGTGGTGGTTACTACTAGCCGATTCTCTCGAGAAGTCACAGAGT
ATTACCAGTCCGTGTACACCCGCTTCGGCTCTCGTGGATCTCAAATCGTTGTCGTCCCCTTTAATCAG
GGAAGCAAGCAAGACGTTGAGGCACTTGTCAACTACATCTATGACACCAAGAACGGCCTTGGCTGG
GATCTCGACTACATTGTTCCTTTTGCCGCCATCCCCGAGAATGGTCGACAGATTGATAGCATCGACTC
CAAGTCTGAGTTGGCTCACCGTATCATGCTCACCAACTTGGTCCGCATGCTTGGCTTTGTCAAGGCTC
AGAAGGCTGAGCGCGGATTTGAGACTCGTCCTGCCCAAGTTGTCCTCCCTCTGTCCCCCAACCACGG
TACCTTCGGTAACGATGGTCTCTACTCCGAGTCCAAACTGGCTCTCGAGACCCTCTTCAACCGATGGC
ACTCTGAGAGCTGGGGCCACTATCTCACGATTTGTGGTGCTGTTATTGGATGGACTCGCGGTACTGG
TCTCATGTCTGGCAACAATATTGTCGCCGAGGGCGTTGAGGCGTTCGGCGTGCGAACATTCTCTCAG
CAAGAGATGGCATTTAACCTCTTGGGTTTGATGTCCGCTACCATTGTTGACCTTTGCCAGTCAGAGCC
AGTCTTTGCCGACTTGAACGGAGGTCTGCAATTCATCCCCAACTTGAACGAATCCATGACCAAGCTC
CGCAAGGACATCATGGAGACTAGTGAAATCCGCCGAGCCGTTAGCAAGGAGAGCGCTATCGAGAA
TACCATTGTCAACGGTGCTGACTCAGAGGTTCTCTATAAGAAGAAGACCATCGAGCCCCGCGCCAAC

ATCAAGTTTGACTTCCCTCCCTTGCCCGACTGGAAGACGGAGGTGGCTCCACTCAACGACCAGCTGA
AGGGCATGGTCGATTTGGAGAAGGTCGTTGTCGTGACTGGTTTTGCGGAAGTTGGTCCTTGGGGCA
ACTCTCGAACTCGATGGGAAATGGAGGCCTATGGCGAGTTCTCGCTCGAGGGTTGCATTGAGATGG
CCTGGATCATGGGTCTTATCAAGAATCACAACGGCCCTCTCAAGGGCAAGCCTTATGCCGGCTGGGT
TGATGCCAAGTCTGGCGAGCCGGTTGATGACAAGGACGTTAAGCAAAAGTACGAGAAGTACATTCT
GGAACACTCCGGTATTCGCTTGATTGAGCCCGAGTTGTTCGATGGATATGACCCCAACAAGAAGCA
GCTGCTTCACGAGGTCGTCATTGAGCAGGACCTTGAGCCGTTCGAGGCCTCTAAGGAGACTGCGGA
AGAATTCCGACGTGAACATGGCGACAAGGTGGAGATTTTTGAGATCCCCGAGTCAGGAGAGTATAT
TGTCCGGGTAAAGAAGGGCGCTTCGCTCTGGATCCCCAAGGCGCTGCGCTTCGACAGACTCGTCGC
TGGTCAAATCCCAACTGGCTGGGACCCCAAGCGATATGGTGTTCCCGAGGATATCATCAGCCAGGT
GGACCCTGTCACATTGTTCCTCCTCGTGTCCACTGCTGAGGCCCTCCTGTCTTCTGGTATTACTGATCC
ATACGAGTTCTACAAGTATGTCCACGTCTCCGAGGTTGGTAACATCGTCGGCTCTGGTATGGGTGGC
GCTGCAGCCCTGCGTGGCATGCACAAGGCCCGATTCCTCGACAAGCCTCTCCAGAACGATATCTTGC
AGGAGTCTTTCATCAACACCATGGCTGCATGGGTTAACATGTTGCTTCTCTCTTCTTCTGGTCCTATCA
AGACACCTGTCGGCGCTTGTGCTACTGCCATCGAGTCTGTCGACATTGGTGTTGAGACGATCATGGA
AGGCAAGGCCCGTATCTGCCTGGTCGGCGGCTTCGACGACTTTGGTGAGGAAGGTTCTTACGAGTT
TGCCAACATGAAGGCTACTAGCAACACAATGGATGAGTTTGCCCACGGTCGTACGCCTGGTGAAAT
GTCGCGTCCTATGACCACTACCCGAAATGGATTTATGGAGTCTCAGGGATGTGGTGTCCAGGTCATC
ATGACCGCCAAGCTCGCTCTGGAGATGGGAGTCCCAATTCACGGTATCCTTGCTTTCACCACTACTGC
CTCTGACAAGATTGGACGATCCGTCCCCGCCCCTGGCCAAGGTGTTCTCACATCCGCCCGAGAGCAT
GTCGGCAAGTTCCCATCGCCGCTGCTTGATATCAACTACCGTCGCCGACAGATTGAGCGCCGCAAGA
AGCAGATCAAGCAATGGGAAGAGTCTGAGCTGGAGTTCCTGCACGATGAGATTGATGCTATGAAGG
CCCAAGGCGGTGCATTCGACGAGAAGGAGTACGCTCAGGATCGCGTGGCACACATCCAGAAGGAG
GCCGAGAGACAAGAAAAGGAGCTTCTCCGAAGCATGGGTAACAACTTCTGGAAGAGCGACCCAAC
CATTGCTCCGCTCCGAGGCGCCCTTGCAACCTGGGGTCTTACTATCGATGACCTGAAGGTTGCCTCG
TTCCACGGCACATCCACTGGTGCCAACGACAAGAACGAGTCCTCGGCCATTTGCCAGCAGCTGCGCC
ACCTTGGCCGAAGCAAGGGCAACGCAGTCCTTGGTGTCTTCCAAAAGTATCTCACTGGTCACCCCAA
GGGTGCTGCAGGTGCTTGGATGATGAACGGCGGTCTGCAAATTCTAAACTCTGGTCTGGTTCCCGG
CAACAGAAATGCTGACAATATTGACCCCATCATGGAGAACTATGACCTGATCGTCTACCCCAGCCGT
AGCATCCAGACTGACGGAGTCAAGGCTTTCTCCCTCACCTCGTTCGGTTTCGGGCAGAAGGGAGCCC
AGGCAGTCGGCGTCCACCCCAAGTATCTGTACGCCACCCTGGACCAGAAGACTTACCAGGAGTACT
GTGCCAAGGTTGAGGCCCGACAGAAGAAGGCCTACCGTTTCTTCCACAGCGGCATGATCAGCAACG
CCCTGTTCGTCTCCAAGTCTCACGCTCCTTACAATGAGGAGCAGCTCAGCGCCGTGCTGCTGAACCCC
GATGCTCGTGTAACCGAAGACAAGAAGACTGCAGAACTCAAGTATCCGGCCAATTTCATGAAGGCG
TCTGAAAAGACTCCCTCTGCAACTGCCGTCAAGGAGACACAGCAGGTTATCGAGGCGCTTGCACACA
AGGTGACGAGCAAGAACAGCAACGTGGGCGTCGATGTCGAGGATATTACATCCTTCAACATTGAGA
ACGACACATTCATTGAGCGCAACTTTACCAGCCAGGAAGTGGCTTATTGCAAGAGTGCGCCCAGCCC
ACAGAGCTCATTCGCCGGTCGATGGAGCGCCAAAGAGGCGGTATTTAAAGCTCTTGGTGTCAGCAG
CAAGGGAGCCGGCGCTGCCTTGAAGGACATTGAAATCCTCAAGGACGATACTGGCGCGCCTGTTGT
TTCTCTTCATGGCGATGCGGCCGCCGCTGCCAAGCAGGCTGGCGTGAAGGAGGTTTCGGTCTCCATC
TCGCATGCTGACAAGCAGGCTGTTGCCGTGGCAGTTGCTCACTTTTAA
>Trichoderma asperellum
ATGCGTCCTGAAGTTGAGCAAGAGCTCGCTCACACGCTCCTCGTTGAGCTTCTAGCATACCAGTTCG
CCTCTCCCGTCAGGTGGATTGAGACTCAGGATGTGTTCCTGGCAGAGAAGACGGCCGAGCGCATCG
TCGAAGTCGGCCCCGCAGATACCCTCGGAGTGATGGCCAAGCGCACATTGGCGTCCAAATACGAAG
CGTACGACGCGGCCAAGTCTGTTCAGCGACAGATCCTCTGCTACAACAAGGATGCCAAGGAGATTT
ACTACGATGTTGACCCCGTTGAAGAAGAGCCCGAGCCTGCTGCGGCCAGCTCATCAGATGCCCCCA

GCCAGCCTGCTGCGGGCGCTCCCGCGGCTGCAGCTGCGCCGGTTGCTGCTGCTCCGGCTCCCTCCTC
AGGGCCTGCAGCTCAGGTTCCTGATCAGCCAGTGCAGGCTGTCGACATCGTACATACCATCGTTGCG
CAAAAACTCAAGAAGTCTCTTAGTGATGTGCCGTTGAGCAAGGCCATCAAGGATCTGGTTGGAGGC
AAATCTACTCTTCAAAATGAGATTCTTGGTGATCTTGGAAAGGAATTCGGCTCAACGCCTGAGAAGC
CTGAAGATACGCCTCTCGACGAGCTCAGCGCTTCCATGCAGGCCACCTTCGACGGCAACCTGGGCAA
GCACACACAGTCACTCATCGCCAGATTGATCTCCTCGAAGATGCCTGGCGGCTTCAATATCACAGTA
GCAAGAAAATACCTGGAAACAAGATGGGGCCTTGGTTCTGGTAGACAAGACGGAGCACTGCTGCTG
GCTTTGACCATGGAGCCTGCTGCTCGTCTAGGAAATGAAGGCGATGCCAAGGGATTCCTTGATGGG
GTTGTCCAGAAGTATGCCGCAAACGCCGGTATCAGCCTGACGTCTGCGGCTGCCTCTGGTGGCTCCG
AAGGTGGCTCTGGAGGCATGATGATGGATCCCGCTGCCATTGACGCCCTCACCAAGGACCAGCGAG
CTCTGTTCAAGCAACAACTGGAGCTCTTTGCCCGATACCTCAAGATTGATCTCAGGTCTGGCGACAA
GGCGCATATTGAGTCTCAAAAATCAGAAAAGATCCTGCAGGCCCAGCTCGACCTGTGGACCGCAGA
GCACGGCGACTTCTATGCCTCTGGTATTGAGCCCGTCTTCAGCCCATTGAAGGCGAGATCTTACGAC
TCATCCTGGAACTGGGCTCGTCAAGATGCACTCACTATGTACTATGACATTATTTTTGGTAGACTCAA
GACTGTCGACCGTGAAATTGTCAGCCAGTGCATCCGTATCATGAACAAATCTAACCCCAAGCTTCTC
GAGTTCATGCAGTATCACATCGATAACTGCCCTACTGAGCGCGGTGAGACCTACCAGCTCGCCAAAG
AACTTGGTCAGCAGCTCATTGAGAACTGCAAAGAAGTTTTGGAGCTGTCTCCTGTCTATAAGGACGT
TGCTGTCCCCACTGGTCCTCGCACCACCGTGGATGCGCGTGGCAACCTCAACTACGAGGAAGTTCCT
CGCGCCAGCTGCAGAAAACTCGAGCACTACGTCCAACAGATGGCCGAGGGTGGAAAGATTTCCGAG
TATGGCAACCGCACCAAGGTGCAAAACGACCTGCAGCGTATCTACAAGTTGATTAAGCAGCAGCAC
AAGATGTCCAAGACTTCTCAGCTCGAAATCAAGAGCTTGTACGGTGATGTTTTGCGCTCTCTGGCCA
TGAACGAGAGCCAGATCATTCCCAAGGAGAACGGCAAGGGCCGAAAGTCCGGCCTGAAGGGCACC
AGCCTGAACAAGGGCAGGGTTGAGACCATCCCATTCCTTCATCTCAAGAGAAAGACCCTGCACGGCT
GGGACTACAGCAAGAAGCTCACTGCTGTCTACCTCAACTCTCTTGAGCAGGCTGCCAAGGATGGTGT
CACTTTCCAGGACAAGTATGTCTTGATGACTGGTGCTGGTGCCGGATCCATTGGTGCTGAGGTTCTG
CAGGGCCTCGTCAGTGGAGGTGCCAAGGTTGTGGTCACTACCAGTCGATTCTCCCGAGAGGTCACT
GAGTACTACCAGGCCATGTACACCCGTTTTGGCTCTCGCGGATCTCAGATCGTTGTCGTCCCCTTCAA
CCAGGGAAGCAAGCAAGACGTCGAGGCCCTCGTTGACTACATCTACGACACTAAGGCGGGTCTTGG
CTGGGATCTCGACTACATTGTTCCTTTCGCTGCCATTCCTGAGAATGGCCGACAGATTGATAACATCG
ACTCCAAGTCGGAGCTGGCCCACCGTATTATGCTGACCAACTTGATCCGCATGCTCGGTTACGTCAA
GTCTCAAAAGGCTGAGCGAGGCTTCGAAACTCGTCCCGCTCAAGTCGTCCTTCCCCTGTCTCCTAACC
ACGGTACCTTTGGTAACGACGGTCTCTACTCCGAGTCTAAGCTCGCTCTCGAGACTCTCTTCAACCGA
TGGCACTCTGAGGACTGGGGCCACTACCTCACAATTTGCGGTGCTGTTATTGGATGGACTCGCGGTA
CTGGTCTCATGTCTGGCAACAACATTGTTGCCGAGGGTGTTGAGGCTTTTGGCGTCCGAACATTCTCT
CAGCAAGAGATGGCTTTCAACCTCTTGGGTCTGATGTCCGCTACCATTGTCGACCTCTGCCAGTCAG
AGCCCGTCTTTGCCGACTTGAACGGTGGCTTGCAATTCATCCCCAACCTGAACGAGGCCATGACCAA
GCTTCGCAAGGATATTATGGAGACTAGTGAAATCCGCAGAATCGTTTCTAAGGAGAGCGCTATCGA
GAACACTATTGTCAACGGAGCCGACTCCGAGGTCCTTTACAAGAAGAAGACCATCGAGCCCCGTGC
CAACATCAAGTTTGACTTCCCTCATCTGCCCGACTGGAAGACCGAGGTCGCTCCTCTTAACGACCAGC
TCAAGGGCATGGTCGACCTGGAGAAGGTAGTTGTCGTCACTGGTTTCGCGGAAGTCGGCCCTTGGG
GTAACTCTCGAACTCGATGGGAGATGGAGGCCCATGGCGAATTCTCGCTCGAAGGCTGCATTGAAA
TGGCCTGGATCATGGGTCTCATCAAGAACCACAATGGTCCTCTCAAGGGCAAGCCTTACGCTGGCTG
GGTTGATGCTAAGACCGGCGAACCTGTTGATGACAAGGACGTCAAGCAGAAGTACGAGAAGTTCAT
TCTGGAACACGCCGGTATTCGTTTGATTGAGCCTGAGCTGTTCGATGGATACGACCCCAACAAGAAG
CAGCTGCTTCACGAGGTTGTCATTGAAGAGGACCTTGAGCCATTCGAGGCTTCCAAGGAGACTGCC
GAAGAGTTCCGACGAGAACATGGTGATAAGGTGGAGATTTTCGAGATCCCCGAGTCTGGAGAGTAC
ACTGTCCGAGTGAAGAAGGGCGCCTCGCTCTGGATCCCCAAGGCTCTGCGCTTCGACAGACTCGTC

GCTGGCCAGATTCCAACTGGCTGGGACCCCAAGCGATATGGTATCCCTGACGATATTGTCAGTCAAG
TAGATCCTGTCACCCTGTTCCTTCTCGTGTCGGTTGCTGAGGCCCTCCTGTCCTCTGGTATTACCGACC
CCTACGAGTTTTACAAGTATGTCCACGTCTCTGAAGTTGGTAACATTGTTGGCTCTGGTATGGGTGGT
GCTTCAGCCCTGCGTGGCATGCACAAGGCCAGATTCCTCGACAAGCCCCTTCAGAACGATATCTTGC
AGGAATCCTTCATCAACACTATGCCTGCTTGGGTTAATATGTTGCTGCTCTCTTCGTCTGGACCTATCA
AGACCCCTGTTGGTGCTTGTGCCACTGCTATTGAGTCTGTTGACATTGGTGTGGAGACCATTTTGGA
AGGCAAGGCTCGCATCTGTCTTGTCGGTGGTCTCGACGACTTTGGTGAGGAAGGCTCTTACGAGTTC
GCCAACATGAAGGCCACCAGCAACTCATTGGATGAGTTTGCCCATGGCCGTACTCCCGGTGAAATGT
CACGTCCTACGACTACTACCCGAAACGGCTTCATGGAGTCTCAGGGATGTGGTGTCCAGGTCATCAT
GACAGCCAAGCTTGCTCTGGATATGGGAGTCCCTATCCACGGTATCCTTGCTTACACCACCACTGCCT
CTGACAAGATCGGACGTTCCGTCCCTGCTCCTGGCAAGGGTGTTCTTACGTCTGCGCGTGAGCACGC
TGGCAAGTTCCCCTCGCCACTGCTTGACATCAACTACCGTCGCCGACAGATTGAGCGCCGCAAGAAA
CAGATTAAGCAGTGGGAAGAGTCTGAGCTGGAGTTCTTGCACGATGAAATCGATGCTATGAAGGCT
CAGGGCGGTGCCTTTGACGAGAAGGAGTACGCCCAGGATCGTGTCGCACACATCCAGAGGGAGGC
TGAGAGACAAGAAAAGGAGCTCCTCCGAAGCATGGGCAACAACTTCTGGAAGAGCGATCCATCCAT
CGCTCCCCTCCGTGGTGCTCTTGCAACATGGGGCCTTACCATTGACGACCTAAAGGTTGCCTCTTTCC
ACGGTACATCTACCGGTGCCAACGACAAGAACGAGTCCTCTGCCGTTTGCCAGCAGCTGCGTCACCT
TGGCAGAAGCAAGGGTAACGCCGTCCTGGGTGTCTTCCAGAAGTTCCTTACTGGTCACCCCAAGGG
TGCTGCCGGTGCTTGGATGTTGAACGGTGGTCTACAGATTCTGAACACTGGTCTGGTCCCTGGTAAC
AGAAATGCCGACAACATCGACCCCATCATGGAAGACTACGACCTGATTGTTTACCCTAGCCGTAGCA
TTCAGACTGATGGCGTCAAGGCTTTCTCCCTCACTTCGTTCGGTTTCGGACAGAAGGGAGCCCAGGC
AGTCGGTGTCCACCCCAGATACCTGTTTGCAACTCTCGACGAAAAGACTTACCAGGAGTACTGCGCC
AAGGTCGAGGCCCGACAGAAGAAGGCTTACCGTTTCTTCCACAACGGCATGATTAGCAACACTCTGT
TCGTCCCCAAGGCTCACGCTCCTTACACTGATGAGCAGCTCAGCGAGGTTCTGATGAACCCTGATGC
TCGTGTCACCGAAGACAAGAAGACAAAGGAGCTCAAGTATGCGGAAAATTTCATGAAGGCGTCTGA
AAAGGTTGTCCCTGCAGTCACTGTCAAGGAGACACAGCAGGTCATTGAGGCGCTCGCTCACAAGGT
TACAAGCAAGAACAGCAATGTGGGTGTAGATGTCGAGGATATCTCTGCCATCAACATTGAGAATGA
TACTTTCATCGAGCGTAACTTTACTAGCCAGGAGATTGCTTACTGCAAGAGCTCTGCTAGCCCCCAGA
GCTCATTCGCTGGCCGATGGAGCGCCAAGGAGGCAGTTTTTAAGTCTCTTGGCGTCGCCAGCAAGG
GTGCCGGCGCTGCTCTGAAGGATATTGAGATCCTCAGGGATGATTCTGGCGCGCCTACTGTTACTCT
TCATGGCGATGCGGCTGCTGCTGCTAAACAGGCTGGTGTGAAGGAGGTTTCCGTCTCCATCTCTCAT
GCTGATAAGCAAGCTGTTGCCGTGGCAGTTGCTCACTTCTAA
>Trichoderma atrobrunneum
ATGCGTCCCGAAGTCGAGCAAGAGCTCGCCCACACGCTCCTCGTCGAGCTTCTGGCATACCAGTTCG
CCTCTCCCGTGAGGTGGATTGAGACCCAGGATGTCTTCCTGGCAGAGAGAACAGCCGAGCGCATCG
TCGAAGTCGGCCCCGCAGATACCCTTGGAGTCATGGCAAAGCGCACATTGGCATCCAAGTATGAGG
CCTACGACGCCGCCAAGTCGGTTCAGCGACAGATCCTCTGCTACAACAAAGACGCCAAGGAGATCT
ACTATGATGTAGACCCCGTTGAGGAAGAGCCCGAGCCTGTTGCCAGCTCATCCGCTGCTCCCTCAAG
TCAACCCGCTGCTGCCAGCGCTCCTGCGGCTGCTGCGCCAGTTGCTGCCGCTCCCGCTGCCGGCGCT
GGACCTGCAGCTCAGGTTCCCGATGTGCCTGTCCAGGCTGTCGAGATTGTGCACACCTTGGTTGCAC
AGAAGCTCAAGAAGTCTCTAGCTGATGTGCCTCTTAGCAAGGCCATCAAGGATCTGGTTGGAGGCA
AATCCACTCTCCAAAATGAGATTCTCGGTGATCTTGGAAAAGAATTCGGCTCAACGCCTGAGAAGCC
CGAAGATACGCCTCTCGACGAGCTCGCCGCCTCTATGCAGGCTACCTTCGATGGCAACCTTGGCAAG
CACACAGTATCCCTCATCGCCAGGCTCATCTCCTCCAAGATGCCTGGTGGCTTCAACATCACAGTGGC
CAGGAAATATCTGGAAACAAGATGGGGTCTCGGCCCCGGCAGGCAAGACGGAGCATTGCTGCTGG
CCTTGACCATGGAGCCTGCTGCCCGTCTAGGAAACGACGGTGAGGCCAAGGGATTCCTCGACGGAA
TCGTCCAGAAGTACGCCGCGAGCGCCGGTATCAGCTTGACATCCGCAGCTGCCTCTGGCGGCTCTGA

AGGAGGTGCGGGAGGCATGATGATGGATCCCGCCGCCATTGATGCTCTCACCAAGGACCAGCGCG
CCCTGTTCAAGCAGCAGCTGGAGCTCTTTGCCAGATACCTCAAGATGGACCTCCGGGCCGGCGACA
AGGCACACATTGACTCCCAGAAATCAGAAAAGGTTCTCCAAGCTCAGCTGGATCTGTGGACCGCCG
AGCACGGTGATTTCTACGCTTCTGGTATCGAGCCTGTCTTTAGCCCGCTCAAGGCTAGATCCTACGAC
TCCTCCTGGAACTGGGCTCGTCAGGATGCGCTCACCATGTTCTACGACATTATCTTTGGCAGACTCAA
GACCGTCGACCGTGAAATTGTCAGCCAGTGCATTCGCATCATGAACAAGTCAAATCCCAAGCTCCTC
GAGTTTATGCAGTACCACATCGACAACTGCCCTACTGAGCGCGGTGAGACCTACCAGCTGGCTAAG
GAACTTGGTCAGCAGCTCATTGAGAACTGCAAGGACGTTCTGAACCTTTCTCCCGTCTACAAGGACG
TTGCTGTTCCCACTGGTCCTCGCACCACTGTGGACGCTCGTGGCAACCTCAACTACGAAGAAGTTCCC
CGCGCCAGCTGCAGGAAGCTCGAACACTACGTTCAGCAGATGGCTGAGGGTGGAAAGATTTCCGAG
TATGGCAACCGCACCAAGGTGCAGAACGACCTGCAGCGTATCTACAAGTTGATCAAGCAGCAGCAC
AAGATTTCCAAGACTTCTCAGCTCGAGATCAAGAGCCTTTACGGCGATGTTCTGCGCTCCCTGGCCAT
GAACGAGAGCCAGATCCTCCCCAAGGAGAATGGCAAGGGCCGAAAGCCGGGTCTCAAGGGCACAA
ACCCCAACAAGGGCCGAGTCGAGACCATCCCCTTCCTCCACCTCAAGAGAAAGACCCTGCACGGCTG
GGACTACAGCAAGAAGCTCACTGCTGTCTACCTCAACTGTCTTGAGGAAGCCGCCAAGGATGGTGT
CACCTTCCAGGACAAGTATGTCCTGATGACTGGTGCTGGTGCCGGTTCCATTGGTGCCGAGGTCCTG
CAGGGCCTCATCAGCGGAGGAGCCAAGGTTGTGGTCACCACTAGCCGATTCTCTCGACAGGTGACC
GAGTACTACCAGTCCATGTACAGCCGCTTTGGCTCTCGCGGATCGCAAATCGTTGTCGTTCCCTTCAA
CCAGGGAAGCAAGCAGGATGTGGAGGCCCTTGTCGACTACATCTACGACGCCAAGAACGGTCTCGG
CTGGGATCTCGACTTCATTGTTCCCTTTGCTGCCATCCCTGAGAACGGCCGACAGATTGATAGCATCG
ATTCCAAGTCGGAGCTGGCTCACCGTATTATGCTCACCAACTTGATCCGCATGCTTGGCTGCGTCAA
GACTCAAAAGGCCGAGCGTGGCTTTGAGACCCGTCCCGCCCAGGTCGTCCTCCCTCTGTCCCCCAAC
CACGGTACCTTCGGTAACGATGGTCTCTACTCCGAGTCCAAGCTCGCTCTCGAGACCCTCTTCAACCG
ATGGCACTCAGAGGACTGGGGCCACTACCTCACCATTTGCGGTGCTGTTATTGGATGGACTCGTGGT
ACTGGTCTCATGTCTGGCAACAACGTTGTTGCTGAGGGTGTGGAGGCGTTCGGCGTGCGAACCTTCT
CCCAGCAAGAGATGGCCTTCAACCTGTTGGGCCTGATGTCAGCTACTATTGTTGATCTCTGCCAGTCA
GAGCCCGTCTTTGCCGACTTGAACGGTGGCCTGCAATTCATCCCCAACCTGAACGAGTCCATGACCA
AGCTCCGCAAGGATATCATGGAGAGTAGCGAAATCCGCAGAGCCGTTACCAAGGAGAGCGCTATCG
AGAACACCATTGTTAACGGAGCCGACTCCGAGGTTCTTTACAAGAAGAAGACCATCGAGCCCCGTG
CCAACATCAAGTGTGACTTCCCTCACCTGCCTGACTGGAAGACGGAGGTGGCCCCTCTCAACGACCA
GCTGAAGGGCATGGTTGACTTGGAGAAGGTCATTGTCGTGACCGGTTTTGCGGAAGTTGGTCCCTG
GGGTAACTCCCGAACTCGATGGGAGATGGAGGCTTACGGCGAGTTTTCGCTCGAAGGTTGCATCGA
AATGGCCTGGATCATGGGCCTTATCAAGAACCACAACGGCCCCCTCAAGGGCAAGCCCTACGCTGG
CTGGGTTGACGCCAAGACTAGCGAGCCCGTCGATGACAAGGATGTCAAGCAGAAGTACGAGAAAC
ACATCCTGGAGCACTCTGGTATTCGTTTGATCGAGCCCGAGCTGTTCGACGGATATGACCCCAACAA
GAAGCAGCTGCTCCACGAGGTCGTCATTCAGGAGGACCTGGAGCCTTTCGAGGCTTCCAAGGAGAC
TGCAGAGGAATTCCGACGCGAGCACGGTGACAAGGTGGAGATTTTCGAGATCCCCGAGAGTGGAG
AGTACATTGTCCGGGTGAAGAAGGGCGCCTCGCTCTGGATCCCCAAGGCTCTGCGCTTTGACAGACT
CGTCGCCGGTCAGATCCCGACTGGCTGGGATCCCAAGCGATATGGTGTCCCCGAGGACATCATCAG
CCAGGTGGACCCTGTCACCCTGTTCCTTCTCGTGTCAACTGCCGAAGCTCTCCTGTCGTCTGGTATCA
CCGACCCCTATGAGTTCTACAAGTACGTTCACGTCTCTGAGGTTGGTAACATTGTCGGCTCTGGTATG
GGCGGTGCTGCGGCCCTTCGTGGCATGCACAAGGCCCGATTCCTCGACAAGCCTCTTCAGAACGAC
ATCTTGCAGGAGTCTTTCATCAACACCATGGCTGCATGGGTCAACATGTTGCTGCTCTCTTCGTCTGG
ACCTATCAAGACGCCTGTCGGCGCTTGTGCTACTGCTATTGAGTCTGTCGACATTGGTGTGGAGACT
ATTCTCGAAGGCAAGGCACGTATTTGTCTTGTCGGTGGTTTCGACGACTTTGGTGAGGAAGGCTCTT
ATGAGTTCGCCAACATGAAGGCCACCAGCAACAGTGTGGATGAGTTTGCCCATGGCCGTACACCCG
GTGAAATGTCACGACCTACTACCACTACCCGAAACGGCTTCATGGAGTCTCAGGGATGTGGTGTCCA

GGTCATCATGACTGCCCAGCTCGCTCTGGACATGGGTGTCCCGATCCACGGTATCCTTGCCTTTACCA
CCACCGCTTCCGACAAGATTGGACGATCCGTCCCTGCGCCTGGCAAGGGTGTCCTCACTTCTGCCCG
AGAGCATGCTGGCAAGTTCCCATCGCCGCTGCTTGATATCAACTACCGCCGCCGACAGATTGAGCGC
CGCAAGAAGCTGATCAAGCAGTGGGAGGAGTCTGAGTTGGAGTTCCTGCACGATGAGGTTGATGCT
ATGAAGGCCCAGGGCGGTAAATTCGACGAGAAGGAGTACGCTCAGGACCGCATGATGCACATCCA
GAAGGAGGCCGCAAGACAAACAAAGGAGCTCCTCCGAAGCATGGGTAACAACTTCTGGAAGAGTG
ACCCAAGCATCGCTCCTCTCCGAGGTGCCCTTGCAACCTGGGGTCTTACCATTGATGATGTCAAAGTT
GCTTCGTTCCACGGCACATCCACTGGCGCCAACGACAAGAACGAGTCTGCGGCCATCTGCCAGCAGC
TGCGTCACCTTGGCCGAAGCGAGGGCAATGCCATTATGGGTGTCTTCCAAAAGTTCCTTACTGGTCA
CCCCAAGGGTGCTGCCGGTGCTTGGATGTTGAACGGCGGCCTGCAGATCCTCAACTCTGGATTGGTC
CCTGGTAACAGAAACGCCGACAACATTGACCCCATCATGGAGCAATACGACCTGATTGTCTACCCCA
GCCGTAGCATCCAGACCGACGGAGTCAAGGCTTTCTCCCTGACCTCGTTTGGTTTCGGACAGAAGGG
AGCCCAGGCAGTCGGCGTTCACCCCAAGTACCTGTTCGCTACTCTCGACGAGAAGACTTACGATGCG
TACCGCGTCAAGGTCGAGGCCCGACAGAAGAAGGCCTACCGCTACTTCCACAACGGAATGATTAGC
AACACCCTGTTTGTCTCCAAGGCCAACGCGCCTTACAGCGATGACCAGCTCAGCGCTGTGCTGCTGA
ACCCCGATGCTCGTGTGAGCGAGGATAAGAAGACGGCGGAGCTTAAGTATGCCGCCAACTTCATGA
AGCAGTCTGAGAAGATCACTCCCGCGACCACCGTCAAGGAGACTCAGCAGGTCATCGAAGCGCTCG
CGCACAAGGTGACGAGCAAGAACAGCAACGTCGGCGTTGATGTCGAGGATATTCCATCCTTCAACG
TTGATAATGACACGTTCATTGAGCGCAACTTCACCACCCAAGAAGTGGCCTACTGCAAGACTGCCCC
TAGCCCGCAGAGCTCATTTGCTGGCCGATGGAGCGCCAAGGAAGCCGTTTTCAAGGCCCTTGGTGTT
GCCAGCAAGGGAGCTGGCGCTGCCCTGAAGGACATTGAGATTCTCAAGGATGACACTGGCGCGCCT
GTTGTTACTCTCCATGGTGAAGCCGCCGCCGCTGCCAAGCAGGCTGGTGTTAAGGAGGTCTCGGTCT
CCATTTCACACGCCGACAAGCAGGCCGTTGCCGTAGCAGTTGCCCATTTCTAA
>Trichoderma atroviride
ATGCGTCCTGAAGTTGAGCAAGAGCTCGCCCACACGCTCCTCGTTGAGCTTCTAGCATACCAGTTCG
CCTCTCCCGTCAGGTGGATTGAGACTCAGGATGTGTTCCTGGCAGAGAAGACGGCCGAGCGCATCG
TCGAAGTCGGTCCCGCAGATACCCTCGGAGTGATGGCTAAGCGCACATTGGCGTCCAAATACGAAG
CCTACGACGCTGCCAAGTCTGTTCAGCGACAGATTCTCTGCTATAACAAAGACGCCAAGGAGATTTA
CTACGATGTCGACCCCATTGAAGAAGAGCCCGAGCCCGCTGCGGCCAGCTCATCAGATGCCCCCAG
CCAGCCTGCTGCGGGCGCCCCCGCGGCTGCTGCTCCGGCTGCTGCTGCTGCTCCGGCTCCCTCCTCA
GGACCTGCAGCCCAGGTTCCTGATCAGCCAGTGCAGGCTGTCGAAATTGTACACACCATTGTTGCAC
AAAAGCTCAAGAAGTCTCTTAGTGATGTGCCTTTGAGCAAGGCCATCAAGGATCTGGTTGGAGGCA
AATCTACTCTTCAGAATGAGATTCTCGGTGATCTTGGAAAAGAATTCGGCTCAACGCCTGAGAAGCC
CGAAGATACCCCTCTCGACGAGCTCAGCGTTTCCATGCAGGCCACTTTCGACGGCAACCTGGGCAAG
CACACACAGTCACTCATCGCCAGATTAATCTCCTCGAAGATGCCTGGTGGTTTCAACATCACAGTAGC
AAGAAAATACCTGGAAACAAGATGGGGCCTTGGTTCTGGTAGACAAGACGGAGCACTGCTGCTGGC
TTTGACCATGGAGCCTGCTGCCCGTCTAGGCAACGAAGGCGAGGCTAAGGGCTTCCTTGACGGAGT
TGTCCAGAAATATGCCGCCAGTGCCGGTATTAGCCTGACGTCTGCGGCTGCTTCTGGTGGCTCCGAA
GGCGGCTCGGGAGGCATGATGATGGATCCCGCTGCCATTGACGCCCTCACCAAGGACCAGCGAGCC
CTGTTCAAGCAACAGCTTGAGCTCTTTGCCCGTTACCTCAAGATCGATCTCAGGTCGGGCGACAAGG
CGCACATTGAGTCTCAAAAATCAGAAAAGGTCCTGCAGGCCCAACTCGATCTGTGGACCGCAGAGC
ACGGCGACTTCTATGCCTCTGGTATTGAGCCCGTCTTTAGCCCATTGAAGGCGAGATCTTACGACTC
GTCCTGGAACTGGGCCCGTCAAGATGCGCTCACCATGTACTATGACATTATTTTCGGTAGACTGCAG
ACTGTCGACCGTGAAATTGTCAGCCAGTGCATCCGCATCATGAACAAGTCAAACCCCAAGCTTCTCG
AGTTCATGCAGTACCACATCGATAACTGCCCTACTGAGCGCGGTGAAACCTACCAGCTCGCCAAAGA
ACTTGGCCAGCAGCTCATTGAGAACTGCAAGGAAGTCCTGGAGCTGTCTCCTGTCTACAAGGACGTT
GCCGTTCCCACTGGTCCTCGCACCACCGTCGATGCCCGTGGCAACCTCAACTACGAGGAAGTTCCTC

GCACCAGCTGCAGAAAACTCGAGCACTACGTTCAGCAGATGGCCGAGGGCGGAAAGATCTCCGAGT
ATGGCAACCGCACCAAGGTTCAGAACGACCTGCAGCGTATCTACAAGTTGATCAAGCAGCAGCACA
AGATGTCCAAGACCTCTCAGCTCGAAATCAAGAGCTTGTACGGTGATGTTTTGCGCTCCCTGGCCAT
GAACGAGAGCCAAATTCTCAAGGAGAACGGCAAGGGCCGAAAGTCGGGCCTGAAGGGCAGCAGC
CTGAACAAGGGCAGGGTCGAGACCATTCCCTTCCTCCATCTCAAGAGAAAGACACTTCACGGCTGG
GACTACAGCAAGAAGCTCACTGCTGTCTACCTCAACTCTCTCGAGCAGGCTGCCAAGGACGGCGTCA
CGTTCCAGGACAAGTATGTTTTGATGACTGGTGCTGGTGCCGGATCCATTGGTGCCGAGGTCCTGCA
GGGCCTCGTCAGCGGAGGTGCCAAGGTCGTGGTCACTACCAGTCGATTCTCCCGAGAGGTGACTGA
GTACTATCAGGCCATGTACACCCGTTTTGGCTCTCGTGGATCTCAAATCGTTGTTGTCCCCTTCAACC
AGGGAAGCAAGCAGGACGTCGAAGCTCTCGTCAATTACATCTACGACGCTAAGACTGGTCTTGGCT
GGGATCTCGACTACATCGTTCCTTTCGCTGCCATCCCTGAGAATGGCCGACAGATTGATAGCATCGA
CTCCAAGTCTGAGCTGGCCCACCGTATTATGCTGACCAACTTGATCCGCATGCTCGGTTACGTCAAG
GCTCAAAAGGCTGAGCGTGGCTTCGAAACTCGTCCCGCTCAGGTTGTCCTTCCCCTGTCTCCCAACCA
CGGTACCTTTGGTAATGATGGTCTCTACTCCGAATCCAAGCTGGCTCTTGAGACTCTCTTCAACCGAT
GGCACTCTGAGGACTGGGGCCACTACCTCACAATTTGCGGTGCTGTTATCGGATGGACTCGCGGTAC
TGGTCTCATGTCTGGCAACAACATTGTCGCTGAGGGTGTTGAGGCGTTTGGTGTCCGAACTTTCTCT
CAGCAAGAGATGGCTTTCAACCTGCTGGGTCTGATGTCCGCTACCATTGTCGATCTCTGCCAGTCAG
AGCCCGTCTTTGCCGACTTGAACGGTGGTTTGCAATTCATCCCCAACCTGAACGAGGCCATGACCAA
GCTTCGCAAGGATATCATGGAGACCAGTGAAATCCGTAGAATCGTTTCTAAGGAGAGCGCTATCGA
GAACACTATTGTCAACGGAGCCGACTCTGAGGTTCTTTACAAGAAGAAGACCATCGAGCCTCGTGCC
AACATCAAGTTTGACTTCCCTCATCTGCCCGACTGGAAGACCGAGGTCGCCCCTCTTAATGACCAGCT
CAAGGGCATGGTCGACCTGGAGAAGGTAGTCGTCGTCACTGGTTTCGCAGAAGTCGGTCCCTGGGG
TAACTCTCGAACCCGATGGGAGATGGAGGCTCATGGCGAATTCTCTCTCGAAGGTTGCATTGAAATG
GCCTGGATCATGGGTCTCATCAAGAACCACAATGGTCCTCTCAAGGGCAAGCCTTACGCTGGCTGG
GTTGACGCTAAGACTGGTGAACCCGTCGACGACAAGGACGTCAAGCAGAAGTACGAGAAGTTCATT
CTGGACCACGCCGGTATCCGTCTGATTGAGCCTGAGCTGTTCAATGGATATGACCCCAACAAGAAGC
AGCTGCTTCACGAGGTTGTCATTGAAGAGGACCTTGAGCCATTCGAGGCCTCCAAGGAGACTGCTG
AAGAGTTCCGACGAGAACACGGTGACAAGGTGGAGATTTTCGAGATCCCCGAGTCTGGAGAGTTCA
CTGTGCGAGTGAAGAAGGGCGCCTCGCTCTGGATCCCCAAGGCTCTGCGCTTCGACAGACTCGTCG
CTGGCCAGATCCCAACTGGCTGGGACCCCAAGCGATATGGTATCCCCGACGATATTGTCAGCCAAGT
GGACCCTGTCACCTTGTTCCTTCTCGTGTCAACTGCTGAGGCTCTCTTGTCCTCTGGTATTACCGATCC
ATACGAGTTCTACAAGTACGTCCACGTCTCTGAAGTTGGTAACATTGTCGGCTCTGGTATGGGTGGT
GCTACGGCCCTGCGTGGCATGCACAAGGCCAGATTCCAAGACAAGCCTCTTCAGAACGATATCCTGC
AGGAATCCTTCATCAACACTATGCCTGCTTGGGTGAACATGTTGCTGCTCTCTTCGTCTGGACCTATC
AAGACCCCGGTCGGAGCTTGTGCCACCGCTATTGAGTCTGTCGACATAGGTGTGGAGACCATTCTG
GAAGGCAAGGCTCGCATCTGTCTTGTTGGTGGTCTCGACGACTTTGGTGAGGAGGGCTCTTACGAG
TTCGCCAACATGAAGGCCACCAGCAACTCAGTAGATGAGTTTGCCCATGGCCGTACTCCTGGCGAAA
TGTCACGTCCTACGACCACGACCCGAAACGGCTTTATGGAGTCTCAGGGATGTGGTGTCCAGGTCAT
CATGACAGCCAGGCTTGCTTTGGATATGGGAGTCCCTATCCACGGTATCCTTGCTTACACCACCACTG
CCTCTGACAAGATCGGACGTTCCGTCCCTGCTCCTGGCAAGGGTGTTCTCACGTCTGCGCGTGAGCA
CGCTGGCAAGTATCCTTCACCACTGCTTGACATCAACTACCGTCGCCGACAGATTGAGCGCCGCAAG
AAGACGATTAAGCAGTGGGAAGAGTCTGAGCTGGAGTTCTTGCACGATGAAATCGATGCTATGAAG
GCTCAGGGTGGTGTCTTTGACGAGAAGGAGTATGCTCAGGATCGCGTCGCACACATCCGGAGGGA
GGCCGAGAGACAAGAAAAGGAGCTCCTCCGAAGCATGGGCAACAACTTCTGGAAGAGCGATCCCT
CCATTGCTCCCCTCCGTGGTGCTCTTGCAACATGGGGCCTTACCATTGACGACCTGAGGGTTGCCTCT
TTCCACGGTACATCCACCGGTGCCAACGACAAGAACGAGTCCTCTGCCGTTTGCCAGCAGCTGCGTC
ACCTTGGCCGAAGCAAGGGTAACGCCGTCCTGGGTGTCTTCCAGAAGTTCCTGACAGGTCACCCCAA

GGGTGCTGCCGGTGCTTGGATGTTGAACGGTGGTCTGCAAATCCTGAACACGGGTCTGGTCCCTGG
TAACAGAAATGCCGACAACATCGACCCCATCATGGAGGACTACGATCTGATTGTTTACCCCAGCCGT
AGCATCCAGACTGATGGAGTCAAGGCTTTCTCTCTCACCTCGTTCGGTTTCGGACAGAAGGGAGCCC
AGGCAGTCGGTGTCCACCCCAGATACTTGTTTGCCGTTCTCGACGAAAAGACATACCAGCAGTACTG
CGCCAAGGTTGAGGCCCGACAGAAGAAGGCTTACCGTTTCTTCCACAACGGCATGATCAGCAACACT
CTGTTCGTTCCCAAGGCTCACGCTCCTTACACCGATGAGCAGCTCAGCGAGGTTCTGATGAACCCTG
ATGCTCGTGTCACCGAAGACAAGAAGACCAAGGAGCTCAAGTATGCCGACAACTTCATGAAGGCGT
CCGAAAAGGTTGTCCCTGCTGTCACTGTCAAGGAGACACAGCAGATCATTGAGGCGCTCGCTCACAA
GGTGACAAGCAAGAGCAGCAACGTTGGCGTAGATGTCGAGGATATCTCTGCCATCAACATTGAGAA
TGACACTTTTATCGAGCGCAACTTCACTAGCCAGGAGATTGCTTACTGCAAGAGTTCTGCTAGCCCCC
AGAGCTCTTTCGCTGGCCGATGGAGCGCCAAGGAGGCAGTCTTCAAGTCTCTTGGCGTCGCCAGCA
AGGGAGCCGGCGCTGCCCTGAAGGATATTGAGATCCTCAAGGATGACTCTGGCGCCCCTACTGTTA
CTCTTCATGGCGATGCAGCTGCTGCTGCCAAACAGGCTGGCGTGAAGGAGGTTTCCGTCTCCATCTC
ACATGCTGACAAGCAGGCTGTTGCCGTGGCTGTGGCTCACTTCTAA
>Trichoderma sp. T154
ATGCGTCCCGAAGTCGAGCAAGAGCTCGCCCACACGCTCCTCGTCGAGCTTCTGGCATACCAGTTCG
CCTCTCCCGTGAGGTGGATTGAGACCCAGGATGTCTTCCTGGCAGAGAGAACAGCCGAGCGCATCG
TCGAAGTCGGCCCCGCAGATACCCTTGGAGTCATGGCAAAGCGCACACTGGCATCCAAGTATGAAG
CCTACGACGCCGCCAAGTCGGTTCAGCGACAGATCCTCTGCTACAACAAAGACGCCAAGGAGATCT
ACTATGATGTAGACCCCGTTGAGGAAGAGCCCGAGCCTGTTGCCAGCTCATCCGCTGCTCCTTCAAG
TCAACCCGCTGCTGCCAGCGCTCCTGCGGCTGCTGCGCCAGTTGCTGCCGCTCCCGCTGCCGGCGCT
GGACCTGCAGCTCAGGTTCCCGATGTGCCTGTCCAGGCTGTCGAGATTGTGCATACCTTGGTTGCAC
AGAAGCTCAAGAAGTCTCTAGCTGATGTGCCTCTTAGCAAGGCCATCAAGGATCTGGTTGGAGGCA
AATCCACTCTCCAAAATGAGATTCTCGGTGATCTTGGAAAAGAATTCGGCTCAACGCCTGAGAAGCC
CGAAGATACGCCTCTCGACGAGCTCGCCGCCTCTATGCAGGCTACCTTCGATGGCAACCTTGGCAAG
CACACAGTATCCCTCATCGCCAGGCTCATCTCCTCCAAGATGCCTGGTGGCTTCAACATCACAGTGGC
CAGGAAATATCTGGAAACAAGATGGGGTCTCGGCCCCGGCAGGCAAGACGGAGCATTGCTGCTGG
CCTTGACCATGGAGCCTGCTGCCCGTCTAGGAAACGACGGTGAGGCCAAGGGATTCCTCGACGGAA
TCGTCCAGAAATACGCCGCGAGCGCCGGTATCAGCTTGACATCTGCAGCTGCTTCTGGTGGCTCTGA
AGGAGGTGCGGGAGGCATGATGATGGATCCCGCCGCCATTGATGCTCTCACCAAGGACCAGCGCG
CCCTGTTCAAGCAGCAGTTGGAGCTCTTTGCCCGATACCTCAAGATGGACCTCCGGGCCGGCGACAA
GGCACACATTGACTCTCAGAAATCAGAAAAGGTCCTCCAAGCTCAGCTGGATCTGTGGACTGCCGA
GCACGGCGACTTCTACGCTTCTGGCATCGAGCCTGTCTTTAGCCCGCTGAAGGCTAGATCTTACGAC
TCTTCTTGGAACTGGGCTCGTCAGGATGCGCTCACCATGTTCTACGACATTATCTTTGGCAGGCTCAA
GACCGTCGACCGTGAAATTGTCAGCCAGTGCATTCGCATCATGAACAAGTCAAATCCCAAGCTCCTC
GAGTTTATGCAGTACCACATCGACAACTGCCCTACTGAGCGCGGTGAGACTTACCAGCTGGCCAAG
GAGCTTGGTCAGCAGCTCATTGAGAATTGCAAGGACGTTCTGAACCTTTCTCCCGTCTACAAGGACG
TTGCTGTCCCCACTGGTCCTCGCACCACTGTGGACGCTCGTGGCAACCTCAACTACGAAGAGGTTCC
CCGCGCCAGCTGCAGGAAGCTCGAGCACTACGTTCAGCAGATGGCTGAGGGTGGAAAGATTTCCGA
GTATGGCAACCGCACCAAGGTGCAAAACGACCTGCAGCGTATCTACAAGTTGATCAAGCAGCAGCA
CAAGATTTCCAAGACTTCTCAGCTCGAGATCAAGAGCCTTTACGGAGATGTCCTGCGCTCTCTGGCC
ATGAATGAGAGCCAGATCCTCCCCAAGGAGAATGGCAAGGGCCGAAAGCCGGGCCTCAAGGGCAC
AAACCCCAACAAGGGCCGAGTCGAGACCATCCCCTTCCTCCACCTCAAGAGAAAGACCCTGCACGGC
TGGGACTACAGCAAGAAGCTCACTGCTGTCTACCTCAACTGTCTTGAGGAGGCCGCCAAGGATGGT
GTCACCTTCCAGGACAAGTATGTCCTGATGACTGGTGCTGGTGCCGGTTCCATTGGTGCCGAGGTCC
TGCAGGGCCTCATCAGCGGAGGTGCCAAGGTTGTGGTTACCACTAGCCGATTCTCTCGACAGGTAA
CCGAATACTATCAGTCCATGTACAGCCGCTTTGGCTCTCGCGGATCGCAAATCGTCGTCGTTCCCTTC

AACCAGGGAAGCAAGCAGGATGTGGAGGCCCTCGTCAACTACATCTACGACGCCAAGACCGGTCTC
GGCTGGGATCTCGACTTCATCGTTCCCTTCGCTGCCATCCCTGAGAACGGCCGACAGATTGATAGCA
TCGATTCCAAGTCGGAGCTGGCTCACCGTATTATGCTCACCAACTTGATCCGCATGCTTGGCTGCGTC
AAGACTCAAAAGGCTGAGCGTGGCTTCGAGACCCGTCCCGCCCAGGTCGTCCTCCCTCTGTCCCCCA
ACCATGGTACCTTCGGTAACGATGGTCTCTACTCCGAGTCCAAGCTGGCTCTTGAGACCCTCTTCAAC
CGATGGCACTCCGAGGACTGGGGCCACTACCTCACCATTTGCGGTGCTGTTATCGGATGGACTCGTG
GTACTGGTCTCATGTCTGGCAACAACGTTGTTGCTGAGGGTGTGGAGGCGTTCGGCGTGCGAACCT
TCTCCCAGCAAGAGATGGCCTTCAACCTGTTGGGCCTGATGTCAGCTACTATTGTTGATCTCTGCCAG
TCAGAGCCCGTCTTTGCCGACTTGAACGGTGGCCTCCAATTCATCCCCAACCTGAACGAGTCCATGA
CCAAGCTCCGCAAGGATATCATGGAGACTAGCGAAATCCGCAGAGCCGTTTCCAAGGAGAGCGCTA
TCGAGAACACCATTGTCAACGGAGCCGACTCCGAGGTTCTTTACAAGAAGAAGACCATCGAGCCCC
GTGCCAACATCAAGTGTGACTTCCCTCACCTGCCTGACTGGAAGACGGAGGTGGCCCCTCTCAACGA
CCAGCTGAAGGGCATGGTCGACTTGGAGAAGGTCATTGTCGTGACCGGTTTTGCTGAAGTTGGTCC
CTGGGGTAACTCTCGAACTCGATGGGAGATGGAGGCCTACGGCGAGTTTTCGCTCGAAGGCTGCAT
CGAAATGGCCTGGATCATGGGCCTCATCAAGAACCACAACGGTCCCCTCAAGGGCAAGCCCTACGC
TGGCTGGGTTGACGCCAAAACTGGCGAGCCCGTCGATGACAAGGATGTCAAGCAGAAGTACGAGA
AACACATTCTGGAGCACTCTGGTATTCGTTTGATCGAGCCCGAGCTGTTCGACGGATATGACCCCAA
CAAGAAGCAGCTGCTCCACGAGGTCGTCATTCAAGAGGATCTGGAGCCTTTTGAGGCTTCCAAGGA
GACTGCAGAGGAATTCCGACGCGAGCACGGTGACAAGGTGGAGATTTTCGAGATCCCCGAGAGTG
GAGAGTACATTGTCCGGGTGAAGAAGGGCGCCTCTCTCTGGATCCCCAAGGCTCTGCGCTTTGACA
GACTCGTCGCCGGTCAGATCCCGACTGGCTGGGATCCCAAGCGATATGGTGTCCCCGAGGACATCA
TCAGCCAGGTGGACCCTGTCACCCTGTTCCTTCTCGTGTCGACTGCCGAGGCTCTCCTGTCGTCCGGT
ATCACCGACCCCTATGAGTTCTACAAGTACGTTCACGTCTCTGAGGTTGGTAACATTGTCGGCTCTGG
TATGGGTGGTGCTGCGGCCCTTCGTGGCATGCACAAGGCCCGATTCCTCGACAAGCCTCTTCAGAAC
GACATCTTGCAGGAGTCTTTCATCAACACCATGGCTGCGTGGGTCAACATGTTGCTGCTCTCTTCGTC
TGGACCTATCAAGACACCTGTCGGTGCTTGTGCTACTGCTATTGAGTCTGTCGACATTGGTGTGGAG
ACTATTCTCGAAGGCAAGGCACGTATCTGTCTTGTCGGTGGTTTCGACGACTTTGGTGAGGAAGGCT
CTTATGAGTTCGCCAACATGAAGGCCACCAGCAACAGTGTGGATGAGTTTGCCCATGGCCGTACGCC
CGGTGAAATGTCACGACCTACTACCACTACCCGAAACGGTTTCATGGAGTCTCAGGGATGTGGTGTC
CAGGTCATCATGACTGCCCAGCTCGCTCTGGACATGGGTGTCCCGATCCACGGTATCCTTGCCTTTAC
CACCACCGCTTCCGACAAGATTGGACGATCCGTCCCTGCGCCTGGCAAGGGTGTCCTCACTTCTGCC
CGAGAGCATGCTGGCAAGTTCCCATCGCCGCTGCTTGATATCAACTACCGCCGCCGACAGATTGAGC
GCCGCAAGAAGCTGATCAAGCAGTGGGAGGAGTCTGAGTTGGAGTTCCTGCACGATGAGGTTGAT
GCCATGAAGGCCCAGGGCGGTAAATTCGACGAGAAGGAGTACGCCCAGGACCGCATGTTGCACAT
CCAGAAGGAGGCCGCAAGACAAACAAAGGAGCTCCTCCGAAGCATGGGTAACAACTTCTGGAAGA
GTGACCCAAGCATCGCTCCTCTCCGAGGTGCCCTTGCAACCTGGGGTCTTACCATTGATGATGTCAA
GGTGGCCTCGTTCCACGGCACATCCACTGGCGCCAACGACAAGAACGAGTCTGCGGCCATCTGCCA
GCAGCTGCGTCACCTTGGCCGAAGCGAGGGCAATGCCATCATGGGTGTCTTCCAAAAGTTCCTTACT
GGTCACCCCAAGGGTGCTGCCGGTGCTTGGATGTTGAACGGTGGCCTGCAAATCCTCAACTCCGGA
TTGGTCCCCGGTAACAGAAATGCCGACAACATTGACCCCATCATGGAGCAATACGACCTGATTGTCT
ACCCCAGCCGTAGCATCCAGACTGACGGAGTCAAGGCCTTCTCCCTGACCTCCTTCGGTTTCGGACA
GAAGGGAGCCCAGGCAGTCGGCGTTCACCCCAAGTACCTGTTTGCTACCCTCGACGAGAAGACTTA
CGATGCGTACCGCGTCAAGGTCGAGGCCCGACAGAAGAAGGCCTACCGCTACTTCCACAATGGAAT
GATCAGCAACACCCTGTTTGTCTCCAAGGCCAACGCGCCTTACAGCGATGACCAGCTCAGCGCTGTG
CTGCTGAACCCTGATGCTCGTGTCAGCGAGGATAAGAAGACGTCGGAGCTTAAGTATGCCGCCAAC
TTCATGAAGCAGTCTGAGAAGATCACTCCCGCGGTCACCGTCAAGGAGACGCAGCAGGTCATCGAG
GCGCTCGCCCACAAGGTCACGAGCAAGAACAGCAACGTCGGTGTTGATGTCGAGGATATTCCATCC

TTCAACATTGACAATGACACGTTCATTGAGCGCAACTTCACCACCCAAGAGGTGGCCTACTGCAAGA
CTGCCCCTAGCCCGCAGAGCTCATTTGCTGGCCGATGGAGTGCCAAGGAGGCCGTGTTCAAGGCTC
TTGGTGTTGCCAGTAAGGGAGCTGGCGCTGCCTTGAAGGACATTGAGATTCTCAAGGACGACACTG
GCGCGCCTATTGTTACTCTCCATGGTGAAGCCGCTGCTGCTGCCAAGCAAGCTGGTGTCAAGGAGGT
CTCTGTCTCCATTTCACACGCCGACAAGCAGGCGGTTGCCGTAGCTGTTGCCCATTTCTAA
>Trichoderma citrinoviride
ATGCGTCCCGAAGTCGAGCAGGAGCTCGCCCACACGCTCCTCGTCGAGCTGCTGGCATACCAATTTG
CCTCTCCCGTGAGGTGGATTGAGACCCAGGATGTCTTCCTGGCAGAGAGAAACGCCGAGCGCATCG
TCGAAATCGGCCCCGCAGACACCCTTGGGGTCATGGCCAAGCGCACACTGGCCTCCAAGTACGAGG
CCTACGATGCCGCAAAGTCGGTCCAGCGACAGATCCTCTGCTACAACAAGGATGCCAAGGAGATTT
ACTACGATGTCGACCCTGTGGAAGAAGAACCCGAACCCGCGGCGAGCTCATCGGCTGCCCCCGCTG
CACCAGCCGCTGCCGGTGCTCCTGCGGCTGCTGCCCCAGTTGCTGCTGCTCCGGCTCCCAGCGCCGG
GCCTGCAGCCCAGGTTCCCGATCAGCCTGTGCAGGCCGTCGACATTGTGCACACCCTGGTTGCGCAA
AAGCTCAAGAAGTCTCTGACTGAGGTGCCTCTGAGCAAGGCCATCAAGGATCTGGTTGGAGGCAAA
TCCACCCTTCAGAATGAAATCCTCGGTGATCTTGGCAAGGAATTCGGTACAACGCCTGAGAAACCCG
AAGATACGCCTCTCGACGAGCTCAGCGCTTCCATGCAGGCTACCTTCGATGGCAACCTTGGCAAGCA
CACACAGTCCCTCATCGCCAGGTTCATCTCCTCTAAGATGCCTGGTGGCTTCAACATCACCGTGGCAA
GGAAATACCTGGAATCAAGATGGGGCCTCGGTCCCGGCAGACAAGACGGAGTACTGCTGCTGGCCT
TGACCATGGAGCCTCCTGCTCGTCTTGGAAACGATGGTGACGCCAAGGCATACCTCGACGGAGTTG
CCCAGAAATACGCCGCCAATGCTGGCATCAGCTTGACGACTGCGGCCGCTTCTGGTGGCTCCGACG
GGGGTGCGGGAGGCATGATGATGGATCCCGCTGCCATTGACGCTCTCACCAAGGACCAGCGCGCTC
TGTTCAAGCAGCAGCTGGAGCTCTTTGCTAGATACCTCAAGATGGACCTCAGAGCCGGCGACAAGG
CGCACATCGAGTCTCAAAAGTCAGAAAAGGTCCTTCAGGCCCAGCTGGATCTGTGGACTGCAGAGC
ACGGTGATTTCTACGCTTCCGGCATTGAGCCTGTCTTCAGCGCATTGAAGGCTAGATCTTACGACTCC
TCCTGGAACTGGGCTCGTCAAGATGCGCTCTCCATGTACTACGACATCATCTTTGGCCGACTCAAGA
CGGTCGACCGTGAGATTGTCAGCCAGTGCATTCGCATCATGAACAAGTCCAACCCCAAGCTCCTCGA
GTTCATGCAGTACCACATCGACAACTGCCCTACCGAGCGCGGCGAGACCTACCAGCTTGCCAAGGA
GCTCGGACAGCAGCTCATTGAGAACTGCAAGGAGGTTCTGAACCTGCCTCCTGCTTACAAGGACGTC
GCTGTCCCCACTGGTCCTCGCACCACCGTGGACGCCCGCGGCAACCTCAACTACGAGGAAGTTCCCC
GCGCCAGCTGCCGGAAGCTCGAGCACTACGTTCAGCAGATGGCCGAGGGTGGCAAGATCTCCGAG
TATGGCAACCGCACCAAGGTGCAGAACGACCTGCAGCGTATCTACAAGTTGATCAAGCAGCAGCAC
AAGATGTCCAAGACTTCTCAGCTCGAGATCAAGAGCCTGTACGGCGATGTTCTGCGCTCCCTGGCCA
TGAACGAGAGCCAGATCATCCCCAAGGAGAACGGCAAGGGCAGGAAGCCAGGCTTCAAGGGCTCA
AGCCCGAACAAGGGCAGAGTCGAGACGATCCCCTTCCTCCACCTCAAGCGCAAGTCCCTGCATGGCT
GGGACTACAGCAAGAAGCTCACGGCCGTCTACCTCAACTGCCTTGAGGAGGCTGCCAAGGACGGTG
TCACGTTCCAGGACAAGTATGTCCTGATGACGGGTGCCGGTGCCGGCTCCATTGGTGCCGAGGTCCT
GCAGGGCCTCATCAGCGGAGGTGCAAAGGTTGTGGTCACCACGAGTCGGTTCTCTCGACAGGTCAC
CGAGTACTACCAGTCCATGTACGCACGCTTTGGTTCCCGCGGCTCTCAGATTGTCGTGGTGCCCTTCA
ACCAGGGAAGCAAGCAGGATGTGGAGGCCCTCGTCAACTACATCTACGACACCAAGAACGGTCTTG
GCTGGGATCTCGACTACATCGTCCCGTTCGCCGCCATTCCCGAGAACGGCCGACAGATTGACAACAT
CGACTCCAAGTCGGAGCTTGCTCACCGCATCATGCTGACCAACCTGATCCGCATGCTCGGCTGCGTC
AAGACGCAAAAGGCTGAGCGCGGCTTCGAGACCCGTCCCGCCCAGGTCGTCCTCCCTCTGTCTCCCA
ACCACGGCACCTTTGGTAACGATGGTCTCTACTCCGAGTCCAAGCTGGCTCTCGAGACCCTCTTCAAC
CGATGGCACTCCGAGGACTGGGGCCACTACCTCACCATCTGCGGTGCTGTCATCGGCTGGACTCGTG
GCACTGGTCTCATGTCTGGCAACAACGTGGTCGCAGAGGGCGTCGAGGCCTTTGGCGTGCGAACCT
TTTCCCAGCAAGAGATGGCCTTCAACCTTCTGGGCCTGATGTCAGCTACCATCGTTGATCTCTGCCAG
TCCGAGCCCGTCTTTGCCGACTTGAACGGCGGTCTGCAGTTCATCCCCAACTTGAACGAATCCATGA

CCAAGCTTCGCAAGGACATCATGGAGACGAGCGAGATCCGCAGAGCCGTCTCCAAGGAGAGCGCC
ATCGAGAACACCATTGTCAACGGAGCCGACTCCGAGGTTCTTTACAAGAAGAAGACCATCGAGCCC
CGTGCCAACATCAAGTGCGACTTCCCCCGCCTTCCCGACTGGAAGACGGAGGTGGCTCCTCTCAATG
AGCAGCTCAAGGGCATGGTCGACTTGGACAAGGTCGTCGTCGTGACCGGTTTTGCCGAAGTCGGCC
CCTGGGGTAACTCCCGAACCCGATGGGAGATGGAGGCCTATGGCGAGTTTTCGCTCGAGGGCTGCA
TCGAGATGGCCTGGATCATGGGCCTCATCAAGAACCACAACGGTCCCCTCAAGGGCAAGCCCTACG
CCGGCTGGGTGGATGCCAAGACTGGCGAGCCCGTCGATGACAAGGATGTCAAGCAAAAGTACGAG
AAGCACATCTTGGAGCACTCCGGTATCCGCCTGATCGAGCCCGAGCTCTTCGACGGATACGACCCCA
ACAAGAAGCAGCTGCTCCACGAGGTCGTCATTCAGGAGGACCTTGAGCCGTTTGAGGCTTCCAAGG
AGACGGCCGAGGAGTTCCAGCGCGAGCACGGTGACAAGGTCGAGATCTTTGAGATCCCCGAGAGT
GGAGAGTACATTATCCGGGTGAAAAAGGGCGCCTCGCTCTGGATCCCCAAGGCTCTGCGCTTTGAC
AGACTGGTCGCTGGTCAGATCCCGACCGGCTGGGACCCCAAGCGATATGGTGTTCCCGAAGACATC
ATCAGCCAGGTGGACCCCGTCACCCTGTTCCTCCTCGTGTCGGTTGCCGAAGCTCTGTTGTCGTCTGG
TATCACCGACCCGTACGAGTTCTACAAGTACGTTCACGTCTCCGAGGTCGGTAACATTGTCGGCTCC
GGTATGGGTGGTGCCAAGGCTCTGCGCGGCATGCACAAGGCGCGATTCCTCGACAAGCCCCTCCAG
AACGACATCCTGCAGGAGTCGTTCATCAACACCATGTCTGCCTGGGTCAACATGCTGCTGCTCTCCTC
GTCCGGTCCCATCAAGACTCCTGTCGGTGCTTGCGCTACCGCTATCGAGTCTGTTGACATTGGTGTG
GAGACGATTCTGGAAGGCAAGGCCCGCATCTGTCTGGTCGGTGGCTTTGACGACTTTGGTGAGGAG
GGTTCATATGAGTTTGCCAACATGAAGGCCACCAGCAACAGCTTGGATGAGTTTGCCCACGGCCGTA
CCCCCGCCGAGATGTCACGTCCCACGACCACCACGCGAAACGGCTTCATGGAGTCTCAGGGATGCG
GTGTCCAGGTCATCATGACTGCTCAGCTGGCCCTGGATATGGGAGTCCCCATCCACGGTATCCTTGC
CTTTACCACCACCGCCTCCGACAAGATCGGACGATCCGTCCCTGCTCCTGGTCAGGGTGTTCTCACCT
CCGCGCGAGAGCATCCCGGCAAGTTCCCGTCGCCCCTGCTTGACATCAACTACCGTCGCAGGCAGAT
TGAGCGTCGCAAGAAGCAGATCAAGCAGTGGGAAGAGTCCGAGTTGGAGTTCCTTCATGATGAGAT
TGAGGCCATGAAGGCCCAGGGCGGTGTCTTTGACGAGAAGGAGTACGCCCAGGAGCGCATCCTGC
ACATCCAGAAGGAGGCCGTCCGACAAGAAAAGGAGCTCCTCCGAAGCATGGGCAACAACTTCTGGA
AGAACGACCCCAGCATCGCTCCTCTCCGCGGAGCCCTTGCCACATGGGGCCTCACCATCGACGACCT
GAAGGTGGCCTCGTTCCACGGCACGTCCACGGGCGCCAACGACAAGAACGAGTCGTCGGCCATCTG
CCAGCAGCTGCGCCACCTTGGCCGAAGCAAGGGCAACGCCATCCTCGGTGTCTTCCAGAAGTACCTT
ACCGGCCACCCCAAGGGTGCTGCTGGTGCTTGGATGATGAACGGTGGTCTGCAGATCCTCAACACC
GGCCTGGTCCCTGGCAACCGGAATGCCGACAACATTGACCCCATCATGGAGAACTACGACCTGATTG
TCTACCCCAGCCGCAGCATCCAGACCGACGGTGTCAAGGCCTTCTCGCTCACCTCGTTCGGTTTCGGC
CAGAAGGGAGCCCAGGCCGTCGGCGTTCACCCCAAGTACCTGTTCGCCACCCTGGACGAGAAGACG
TACGACGCGTACCGTGCCAAGGTTGAGTCCCGACAGAAGAAGGCTTACCGCTTCTTCCACAACGGCA
TGATCAACAACGCCCTGTTTGTCTCCAAGGCCAACCCGCCTTACACCGACGACCAGCTCAGCGCGGT
TCTGATGAACCCCGATGCTCGTGTGGCCGAGGACAAGAAGACGGCAGAGCTCAAGTTCCCGCCCAA
CTTCATGAAGGCGTCCGAGAAGACTGTCTCTCCGACCGTCGCCAAGGAGACGCAGCAGGTGATTGA
GGCGCTCGCGCACAAGGTGGCGGGCAAGAACAGCAACGTGGGCGTGGATGTCGAGGACATTGCCG
CCATCAACATCGACAACGACACGTTCATTGAGCGCAACTTTACCCCCCAGGAGGTTGCCTACTGCAA
GAGCGCTCCTAGCCCGCAGAGCTCGTTTGCCGGCCGGTGGAGCGCAAAGGAGGCCGTGTTCAAGG
CACTTGGTGTTGCCAGCAAGGGAGCTGGCGCTGCCCTCAAGGACATTGAGATCCTCAAGGACGACA
CTGGCGCACCCGTTGTTTCTCTTCATGGTGACGCTGCTGCTGCTGCTAAGCAAGCCGGCGTGAAGGA
GGTCTCCGTCTCCATCTCGCATGCTGACAAGCAGGCCGTTGCCGTGGCCGTTGCCCACTTCTAA
>Trichoderma gamsii
ATGCGTCCTGAAGTTGAGCAAGAGCTCGCCCACACGCTCCTCGTTGAGCTTCTAGCATACCAGTTCG
CCTCTCCCGTCAGGTGGATTGAGACTCAGGATGTGTTCCTGGCAGAGAAAACGGCCGAGCGCATCG
TCGAAGTTGGTCCCGCAGATACCCTCGGAGTGATGGCCAAGCGCACATTGGCGTCCAAATACGAAG

CCTACGACGCTGCCAAGTCTGTTCAGCGACAGATCCTCTGCTACAACAAAGACGCCAAAGAGATTTA
CTACGATGTCGACCCCATTGAAGAAGAGCCCGAGCCCGCTGCGGCCAGCTCATCAGATGCCCCCAG
CCAGCCTGCTGCGGGCGCCCCCGCGGCTGCTGCTCCGGCTGCTGCTGCCCCAGCTCCCTCCTCAGGG
CCTGCAGCTCAGGTTCCTGATCAGCCAGTGCAGGCTGTCGACATTGTACACACCATCGTTGCGCAAA
AGCTCAAAAAGTCTCTTAGTGATGTGCCTCTGAGCAAGGCCATCAAGGATCTGGTTGGAGGCAAAT
CCACTATGCAGAATGAGATTCTCGGTGATCTTGGCAAGGAATTCGGCTCAACGCCTGAGAAGCCCG
AAGATACCCCTCTCGACGAGCTCAGCGCTTCCATGCAGGCCACCTTCGACGGCAACCTGGGCAAGCA
CACACAGTCACTCATCGCCAGATTGATCTCCTCGAAGATGCCTGGTGGCTTCAATATCACAGTAGCA
AGAAAATACCTGGAAACAAGATGGGGCCTTGGTTCTGGTAGACAAGACGGAGCACTGCTGTTGGCT
TTGACCATGGAGCCTGCTGCTCGTCTAGGCAACGAAGGCGAGGCTAAGGGTTTCCTTGACGGAGTT
GTCCAGAAATATGCCGCCAGTGCCGGTATCAGCCTGACATCCACGGCAGCTTCTGGTGGTTCCGAAG
GCGGCTCGGGAGGCATGATGATGGATCCCGCTGCCATTGACGCCCTCACCAAGGACCAGCGAGCGC
TGTTCAAGCAGCAGCTCGAGCTCTTTGCCCGATACCTCAAGATCGACCTCAGGTCGGGCGACAAGGC
GCACATTGAGTCTCAAAAATCAGAAAAGGTCCTTCAGGCCCAGCTCGACCTGTGGACCGCAGAGCA
CGGCGACTTTTATGCCTCTGGTATTGAGCCCGTCTTCAGCCCATTGAAGGCGAGATCTTACGACTCGT
CCTGGAATTGGGCTCGTCAAGATGCGCTCACCATGTACTATGACATTATTTTCGGTAGACTGCAGAC
TGTCGACCGTGAAATTGTCAGCCAGTGCATCCGCATCATGAACAAGTCAAACCCCAAGCTCCTCGAG
TTCATGCAGTACCACATCGATAACTGCCCCACTGAGCGCGGTGAAACCTACCAGCTCGCCAAGGAAC
TTGGCCAGCAGCTCATTGAGAACTGCAAGGAAGTATTGGAGCTGTCTCCTGTCTACAAGGACGTTGC
CGTTCCCACAGGTCCTCGCACCACCGTGGATGCGCGTGGTAACCTCAACTATGAGGAAGTTCCTCGC
GCCAGCTGCAGAAAACTCGAGCACTACGTTCAGCAGATGGCCGAGGGCGGAAAGATCTCCGAGTAT
GGCAACCGCACCAAGGTTCAGAACGACCTGCAGCGTATCTACAAGTTGATCAAGCAGCAACACAAG
ATGTCCAAGACCTCTCAGCTCGAAATCAAGAGCTTGTACGGTGATGTTTTGCGCTCCCTGGCCATGA
ACGAGAGCCAGATTCTCAAGGAGAACGGCAAGGGCCGAAAGCCGACCCTGAAGGGCACCAACCTG
AACAAGGGCAGGGTCGAGACCATTCCCTTCCTCCATCTCAAGAGAAAGACACTTCACGGCTGGGACT
ACAGCAAGAAGCTCACTGCTGTCTACCTCAACTCTCTCGAGCAGGCTGCCAAGGACGGCGTCACGTT
CCAAGACAAGTATGTTTTGATGACTGGTGCTGGTGCCGGATCCATTGGTGCCGAGGTTCTGCAGGG
CCTCGTCAGCGGAGGTGCCAAGGTTGTGGTCACTACCAGTCGATTCTCCCGAGAGGTCACTGAGTAC
TATCAGTCCATGTACACCCGTTTTGGCTCTCGCGGATCTCAAATCGTTGTTGTCCCCTTCAACCAGGG
AAGCAAGCAGGACGTGGAAGCCCTCGTCAATTACATCTACGACGCTAAGACTGGTCTTGGCTGGGA
TCTCGACTACATCGTTCCTTTCGCTGCCATCCCTGAGAATGGCCGACAGATTGATAGCATCGACTCCA
AGTCTGAGCTGGCCCACCGTATTATGCTGACCAACTTGATCCGCATGCTTGGTTACGTCAAGGCTCA
GAAGGCTGAGCGAGGCTTCGAAACTCGTCCCGCTCAAGTTGTCCTTCCCCTTTCTCCCAACCACGGTA
CCTTTGGCAACGACGGTCTCTACTCCGAATCTAAGCTGGCTCTTGAGACTCTCTTCAACCGATGGCAC
TCCGAAGACTGGGGCCACTACCTCACAATTTGCGGTGCTGTTATTGGATGGACTCGCGGTACTGGTC
TCATGTCTGGCAACAACATTGTTGCCGAGGGTGTTGAGGCGTTTGGTGTCCGAACTTTCTCTCAGCA
AGAGATGGCTTTCAACCTCCTGGGTCTGATGTCCGCTACCATTGTCGACCTCTGCCAGTCAGAGCCT
GTCTTTGCCGACTTGAACGGTGGTTTGCAATTCATCCCCAACCTGAACGAGGCCATGACCAAGCTTC
GCAAGGATATCATGGAAACCAGTGAGATCCGTAGAATCGTTTCTAAGGAGAGCGCTATCGAGAACA
CTATTGTCAACGGAGCCGACTCTGAGGTTCTTTACAAGAAGAAGACCATCGAGCCTCGTGCCAACAT
CAAGTTTGACTTCCCTCATCTTCCCGACTGGAAGACCGAGGTCGCCCCTCTTAATGACCAGCTCAAGG
GCATGGTCGACCTGGAGAAGGTAGTCGTTGTCACCGGTTTCGCAGAAGTCGGTCCTTGGGGTAACT
CTCGAACCCGATGGGAGATGGAGGCTCATGGCGAATTCTCTCTCGAAGGCTGCATTGAAATGGCCT
GGATTATGGGTCTCATCAAGAACCACAATGGTCCTCTCAAGGGCAAGCCTTACGCTGGCTGGGTTGA
CGCTAAGACTGGTGAGCCCGTCGACGACAAGGACGTCAAGCAAAAGTACGAGAAGTTCATTCTGGA
CCACGCCGGTATCCGTCTGATTGAGCCTGAGCTGTTTGATGGATATGACCCCAACAAGAAGCAGCTG
CTTCACGAGGTTGTCATTGAAGAGGACCTTGAGCCATTCGAGGCCTCCAAGGAGACTGCCGAAGAG

TTCCGACGAGAACACGGTGACAAGGTGGAGATTTTCGAGATCCCCGAGTCTGGAGAGTTCACTGTC
CGAGTCAAGAAGGGCGCCTCGCTCTGGATCCCCAAGGCTCTGCGCTTCGACAGACTCGTCGCTGGC
CAGATCCCAACTGGCTGGGACCCCAAGCGATATGGTATCCCCGACGACATTGTCAGCCAAGTGGAC
CCTGTCACCTTGTTCCTTCTCGTGTCGACCGCTGAGGCTCTCCTGTCCTCTGGTATTACCGACCCATAC
GAGTTTTACAAGTATGTCCACGTCTCTGAAGTTGGTAACATTGTCGGCTCTGGTATGGGTGGTGCTA
CGGCCCTGCGTGGCATGCACAAGGCCAGATTCCAAGACAAGCCTCTTCAGAACGATATCTTGCAGG
AGTCCTTCATCAACACTATGCCTGCTTGGGTGAACATGTTGCTGCTTTCTTCGTCTGGACCTATTAAG
ACCCCTGTCGGAGCTTGTGCCACCGCTATTGAGTCTGTCGACATAGGTGTGGAGACCATTTTGGAAG
GCAAGGCTCGCATCTGTCTTGTCGGTGGTCTCGACGACTTTGGTGAGGAAGGCTCTTACGAGTTCGC
CAACATGAAGGCCACCAGCAACTCTGTGGATGAGTTTGCTCATGGCCGTACTCCTGGCGAAATGTCA
CGTCCTACGACCACTACCCGAAACGGCTTTATGGAGTCTCAGGGATGTGGTGTCCAGGTCATCATGA
CAGCCAAGCTTGCCCTGGATATGGGAGTCCCTATTCACGGTATCCTTGCTTACACCACCACTGCCTCT
GACAAGATTGGACGTTCCGTCCCTGCTCCTGGCAAGGGTGTTCTCACGTCTGCGCGTGAGCACGCTG
GCAAGTTCCCTTCGCCACTGCTTGACATCAACTACCGCCGCCGACAGATTGAGCGCCGCAAGAAGAC
GATTAAGCAATGGGAAGAATCTGAGCTGGAGTTCTTGCACGATGAAATCGATGCTATGAAGTCTCA
GGGCGGTGTCTTTGACGAGAAGGAGTATGCTCAGGATCGCGTTGCACACATCCAGAGGGAGGCTG
CGAGACAAGAAAAGGAGCTCCTCCGAAGCATGGGCAACAATTTCTGGAAGAGCGATCCCTCCATTG
CTCCCCTCCGTGGTGCTCTTGCAACATGGGGTCTTACCATTGACGACCTGAAGGTTGCCTCTTTCCAC
GGTACATCCACCGGTGCCAACGACAAGAACGAGTCCTCTGCCGTTTGCCAGCAGCTGCGTCACCTTG
GCCGAAGCAAGGGTAACGCCGTCCTGGGTGTCTTCCAGAAGTTCCTGACAGGTCATCCCAAGGGTG
CTGCCGGTGCTTGGATGTTGAACGGTGGTCTGCAAATCCTGAACACTGGTCTGGTTCCTGGTAACAG
GAATGCCGACAACATTGACCCCATCATGGAGGACTACGATCTAATTGTTTACCCCAGCCGTAGCATC
CAGACTGATGGAGTCAAGGCTTTCTCTCTCACCTCGTTCGGTTTCGGACAGAAGGGAGCCCAGGCA
GTCGGTGTCCACCCCAGATACCTGTTTGCCGTTCTCGACGAAAAGACATACCAGGAGTACTGTGCCA
AGGTTGAGGCCCGACAGAAGAAGGCTTACCGATTCTTCCACAACGGCATGATCAGCAACACTCTGTT
CGTCCCCAAGGCTCACGCTCCTTACACCGATGAGCAGCTCAGCGAGGTTCTGATGAACCCTGATGCT
CGTGTCACCGAAGACAAGAAGACCAAGGAGCTCAAGTATGCCGATAACTTCATGAAGGCGTCCGAA
AAGATTGTCCCTGCTGTCACTGTCAAGGAGACACAGCAGATCATTGAGGCGCTCGCCCACAAGGTG
ACAAGCAAGAGCAGCAACGTTGGCGTAGATGTTGAGGATATCTCTGCCATCAACATTGAGAATGAC
ACTTTTATCGAGCGCAACTTCACTAGCCAGGAGATTGCTTACTGCAAGAGCTCTGCTAGCCCCCAGA
GCTCATTCGCTGGCCGATGGAGCGCCAAGGAGGCAGTCTTTAAGTCTCTTGGTGTCGCCAGCAAGG
GAGCCGGCGCTGCCCTGAAGGATATTGAGATCCTCAAGGATGACTCTGGCGCCCCTACTGTTACTCT
TCATGGCGATGCGGCTGCTGCTGCTAAACAGGCTGGCGTGAAGGAGGTTTCCGTCTCCATCTCGCAT
GCTGATAAGCAGGCTGTTGCCGTTGCTGTTGCTCACTTTTAA
>Trichoderma guizhouense
ATGCGTCCCGAAGTCGAGCAAGAGCTCGCCCACACGCTCCTCGTCGAGCTTCTGGCATACCAGTTCG
CCTCTCCCGTGAGGTGGATTGAGACCCAGGATGTCTTCCTGGCAGAGAGAACAGCCGAGCGCATCG
TCGAAGTCGGCCCCGCAGATACCCTTGGAGTCATGGCAAAGCGCACACTGGCATCCAAGTATGAGG
CCTACGACGCCGCCAAGTCCGTTCAGCGACAGATCCTCTGCTACAACAAAGACGCCAAGGAGATCTA
CTATGATGTAGACCCCGTTGAGGAAGAGCCCGAGCCCGTTGCCAGCTCATCCGCTGCTCCCTCAAGT
CAACCCGCTGCTGCCAGCGCTCCTGCGGCTGCTGCGCCAGTTGCTGCCGCTCCCGCTGCCGGCGCTG
GGCCTGCAGCTCAGGTTCCCGATGTGCCTGTCCAGGCTGTCGAGATTGTGCACACCTTGGTTGCACA
GAAGCTCAAGAAATCTCTAGCTGATGTGCCCCTTAGCAAGGCCATCAAGGATCTGGTTGGAGGCAA
ATCCACTCTCCAAAATGAGATTCTCGGTGATCTTGGAAAAGAATTCGGCTCAACGCCTGAGAAGCCC
GAAGATACGCCTCTCGACGAGCTCGCCGCCTCTATGCAGGCCACCTTCGATGGCAACCTTGGCAAGC
ACACAGTATCCCTCATCGCCAGGCTAATCTCTTCCAAGATGCCTGGTGGCTTCAACATCACAGTGGCC
AGGAAATATCTGGAAACAAGATGGGGTCTCGGCCCCGGCAGGCAAGACGGAGCATTGCTGCTGGC

TTTGACCATGGAGCCTGCTGCTCGTCTAGGAAACGACGGTGAGGCCAAGGGATTCCTCGACGGAAT
CGTCCAGAAATACGCCGCGAGCGCCGGTATCAGCTTGACATCTGCAGCTGCTTCTGGTGGCTCTGAA
GGCGGATCGGGAGGCATGATGATGGATCCCGCCGCCATTGATGCTCTCACCAAGGACCAGCGCGCC
CTGTTCAAGCAGCAGTTGGAGCTCTTTGCCCGATACCTCAAGATGGACCTCCGGGCCGGCGACAAG
GCACACATTGACTCTCAGAAATCAGAAAAGGTCCTCCAAGCCCAGCTGGATCTGTGGACCGCCGAG
CACGGCGACTTCTACGCTTCTGGCATCGAGCCTGTCTTTAGCCCGCTCAAGGCTAGATCCTACGACTC
CTCCTGGAACTGGGCTCGTCAGGATGCGCTCACCATGTTCTACGACATTATCTTTGGCAGACTCAAG
ACCGTCGACCGTGAAATTGTCAGCCAGTGCATTCGCATCATGAACAAGTCAAATCCCAAGCTCCTCG
AGTTTATGCAGTACCACATCGACAACTGCCCTACTGAGCGCGGTGAGACTTATCAGCTGGCCAAGGA
GCTTGGTCAGCAGCTCATTGAGAACTGCAAGGACGTCCTGAACCTTTCTCCCGTCTACAAGGACGTT
GCTGTCCCCACTGGTCCTCGCACCACTGTGGATGCTCGCGGCAACCTCAACTACGAAGAAATTCCTC
GCGCCAGCTGCAGGAAGCTCGAACACTACGTTCAGCAGATGGCTGAGGGTGGAAAGATTTCCGAGT
ATGGCAACCGCACCAAGGTGCAAAACGACCTGCAGCGTATCTACAAGTTGATCAAGCAGCAGCACA
AGATTTCCAAGACTTCTCAGCTCGAGATCAAGAGCCTGTATGGCGATGTTCTGCGCTCTCTGGCGAT
GAACGAGAGCCAGATCCTCCCCAAGGAGAATGGCAAGGGCCGAAAGCCGGGTCTCAAGGGCACAA
ACCCCAACAAGGGCCGAGTCGAGACCATCCCCTTCCTCCACCTCAAGAGAAAGACTCTGCACGGCTG
GGACTACAGCAAGAAGCTCACCGCTGTCTACCTCAACTGTCTTGAGGAAGCCGCCAAGGATGGTGT
CACCTTCCAGGACAAGCATGTCCTGATGACTGGTGCCGGTGCCGGTTCCATCGGTGCCGAGGTCCTC
CAGGGCCTCATCAGCGGAGGTGCCAAGGTTGTGGTTACCACTAGCCGATTCTCTCGACAGGTGACC
GAGTACTACCAGTCCATGTACAGCCGCTTTGGCTCTCGCGGATCGCAAATCGTCGTCGTTCCCTTCAA
CCAGGGAAGCAAGCAGGATGTGGAGGCCCTTGTCAACTACATCTACGACACCAAGACCGGTCTTGG
CTGGGATCTCGACTTCATCGTTCCCTTCGCTGCCATTCCTGAGAACGGCCGACAGATTGATAGCATCG
ATTCCAAGTCGGAGCTGGCTCACCGTATTATGCTCACCAACTTGATCCGCATGCTTGGTTGCGTCAAG
GCTCAAAAGGCTGAGCGTGGCTTTGAGACCCGTCCCGCCCAGGTCGTCCTCCCTCTGTCCCCCAACC
ACGGTACCTTCGGTAACGATGGTCTCTACTCCGAGTCCAAGCTGGCTCTTGAGACCCTCTTCAACCGA
TGGCACTCCGAGGACTGGGGCCACTACCTCACCATTTGCGGCGCTGTTATCGGATGGACTCGTGGTA
CTGGTCTCATGTCTGGCAACAACGTTGTTGCTGAGGGTGTGGAGGCGTTCGGCGTGCGAACCTTCTC
CCAGCAAGAGATGGCCTTTAACCTGTTGGGCCTGATGTCGGCTACTATTGTTGATCTCTGCCAGTCA
GAGCCCGTCTTTGCCGACTTGAACGGTGGTCTGCAATTCATTCCCAACCTCAACGAGTCCATGACCA
AGCTCCGCAAGGATATCATGGAGACTAGCGAAATCCGCAGAGCCGTTACCAAGGAGAGCGCTATCG
AGAACACCATTGTTAACGGAGCCGACTCCGAGGTTCTTTACAAGAAAAAGACCATCGAGCCCCGTGC
CAACATCAAGTGTGACTTCCCTCACCTGCCTGACTGGAAGACGGAGGTGGCCCCTCTCAACGACCAG
CTGAAGGGCATGGTTGACTTGGAGAAGGTCATTGTCGTGACCGGTTTTGCGGAAGTTGGTCCCTGG
GGTAACTCTCGAACTCGATGGGAGATGGAGGCCTACGGCGAGTTTTCGCTCGAAGGTTGCATTGAA
ATGGCCTGGATCATGGGCCTCATCAAGAACCACAACGGTCCCCTCAAGGGCAAGCCCTACGCTGGCT
GGGTTGACGCCAAGACTGGCGAGCCTGTCGATGACAAGGATGTCAAGCAGAAGTACGAGAAACAC
ATCCTGGAGCACTCCGGTATTCGTTTGATCGAGCCCGAGCTGTTCGACGGATATGACCCCAACAAGA
AGCAGCTGCTCCACGAGGTCGTCATTCAGGAGGACCTGGAGCCTTTCGAGGCTTCCAAGGAGACTG
CAGAGGAATTCCGACGCGAGCACGGTGACAAGGTAGAGATCTTCGAGATCCCCGAGAGTGGAGAG
TACATTGTCCGGGTGAAGAAGGGCGCCTCGCTCTGGATCCCCAAGGCTCTGCGCTTTGACAGACTCG
TCGCCGGTCAGATTCCCACTGGCTGGGATCCCAAGCGATATGGTGTCCCCGAGGACATCATCAGCCA
GGTGGACCCTGTCACCCTGTTCCTTCTTGTGTCGACTGCCGAAGCTCTCCTGTCGTCTGGTATCACCG
ACCCCTATGAGTTCTACAAGTACGTTCACGTCTCTGAGGTTGGTAACATTGTCGGCTCTGGTATGGGT
GGTGCTGCCGCCCTTCGTGGCATGCACAAGGCCCGATTCCTCGACAAGCCTCTTCAGAACGACATCT
TGCAGGAGTCTTTCATCAACACCATGGCTGCATGGGTCAACATGTTGCTGCTCTCTTCGTCTGGACCT
ATCAAGACGCCTGTCGGCGCTTGTGCTACTGCTATTGAGTCTGTTGACATTGGTGTTGAAACTATTCT
CGAAGGCAAGGCACGCATCTGTCTCGTTGGTGGTTTCGACGACTTTGGTGAGGAAGGCTCTTATGA

GTTCGCCAACATGAAAGCCACCAGCAACAGTGTGGATGAGTTTGCCCATGGCCGTACACCCGGTGA
GATGTCACGACCTACTACCACTACCCGAAACGGCTTCATGGAGTCTCAGGGATGTGGTGTCCAGGTC
ATCATGACTGCCCAGCTCGCTCTGGACATGGGTGTCCCGATTCACGGTATCCTTGCCTTTACCACCAC
CGCTTCCGACAAGATTGGACGATCCGTCCCTGCGCCTGGCAAGGGTGTTCTCACTTCTGCCCGAGAG
CATGCTGGCAAGTTCCCATCGCCGCTGCTTGATATCAACTACCGCCGCCGACAGATTGAGCGCCGCA
AGAAGCTGATCAAGCAGTGGGAAGAGTCTGAGTTGGAGTTCCTGCACGATGAGGTCGATGCTATGA
AGGCCCAGGGCGGTAAATTCGACGAGAAGGAGTACGCTCAGGACCGCATGCTACACATCCAGAAG
GAGGCTGCAAGACAAACAAAGGAGCTCCTCCGAAGCATGGGTAACAACTTCTGGAAGAGTGACCCA
AGCATCGCTCCTCTCCGAGGTGCCCTTGCAACCTGGGGTCTTACCATTGATGATGTCAAGGTGGCCT
CGTTCCACGGCACATCCACTGGCGCCAACGACAAGAACGAGTCTGCGGCCATCTGCCAGCAGCTGC
GTCACCTTGGCCGAAGCGAGGGCAATGCCATTATGGGTGTCTTCCAAAAGTTCCTTACTGGTCACCC
CAAGGGTGCTGCCGGTGCTTGGATGTTGAACGGCGGCCTGCAGATCCTCAACTCTGGATTGGTCCCT
GGTAACAGAAACGCCGACAACATTGACCCCATCATGGAGCAATACGACCTGATTGTCTACCCCAGCC
GTAGCATCCAGACCGACGGAGTCAAGGCTTTCTCCCTGACCTCCTTCGGTTTCGGACAGAAGGGAGC
CCAGGCAGTCGGCGTTCACCCCAAGTACCTGTTCGCTACCCTCGACGAGAAGACTTACGATGCGTAC
CGCGTCAAGGTCGAGGCCCGACAGAAGAAGGCCTACCGCTACTTCCACAACGGAATGATCAGCAAC
ACCCTGTTCGTCTCCAAGGCCAACGCACCTTACAGCGATGACCAGCTCAGCGCTGTGCTGCTGAACC
CCGATGCTCGTGTGAGCGAGGATAAGAAGACGTCGGAGCTTAAGTATGCCGCCAACTTCATGAAGC
AGTCTGAGAAGATCACTCCCGCGACCACCGTCAAGGAGACTCAGCAGGTCATCGAAGCGCTCGCGC
ACAAGGTGACGAGCAAGAACAGCAACGTCGGCGTTGATGTCGAGGATATTCCATCCTTCAACATTG
ACAATGACACGTTCATTGAGCGCAACTTCACCACCCAAGAGGTGGCCTACTGCAAGACTGCCCCTAG
CCCGCAGAGCTCATTTGCTGGCCGATGGAGTGCCAAGGAGGCCGTGTTCAAGGCTCTTGGTGTTGC
CAGCAAGGGAGCTGGCGCTGCCTTGAAGGACATTGAGATTCTCAAGGACGACACTGGCGCACCTGT
TGTTACTCTCCATGGTGAAGCCGCCGCCGCTGCCAAGCAGGCTGGTGTCAAGGAGGTCTCTGTCTCC
ATTTCACATGCCGACAAGCAGGCCGTTGCCGTAGCTGTTGCCCATTTCTAA
>Trichoderma hamatum
ATGCGTCCTGAAGTTGAGCAAGAGCTCGCCCACACGCTCCTCGTTGAGCTTCTAGCATACCAGTTCG
CCTCTCCCGTCAGGTGGATTGAGACCCAGGATGTGTTCCTGGCAGAGAAGACGGCCGAGCGCATCG
TCGAAGTCGGCCCCGCAGATACCCTCGGAGTGATGGCCAAGCGCACATTGGCAGCCAAATACGAAG
CCTACGACGCTGCCAAGTCTGTTCAGCGACAGATCCTCTGCTACAACAAAGACGCCAAGGAGATTTA
CTACGATGTCGACCCCATTGAAGAAGAGCCCGAGCCCGCTGCGGCCAGCTCATCAGAGGCCCCCAG
CCAGCCTGCTGCAGGCGCTCCCGCGGCTGCTGCACCAGTTGCTGCTCCGGCTCCCAGCTCAGGGCCT
GCAGCTCAGGTTCCTGATGCGCCAGTTCAGGCTGTCGAAATTGTGCACACAATCGTTGCGCAAAAAC
TCAAAAAGGGTCTCAGCGATGTGCCTTTAAGCAAGGCCATCAAGGATCTAGTTGGAGGCAAATCCA
CTCTTCAGAATGAGATTCTAGGTGATCTTGGAAAGGAATTCGGCTCAACGCCTGAGAAGCCCGAAG
ACACGCCTCTCGACGAGCTTAGTGCTTCCATGCAGGCCACCTTCGACGGTAACCTGGGCAAGCACAC
ACAGTCACTCATCGCCAGATTGATGTCTTCGAAGATGCCTGGTGGCTTCAATATCACAGTAGCAAGA
AAATACCTGGAAACAAGATGGGGCCTTGGTTCCGGTAGACAAGACGGAGCACTGCTGCTGGCTTTG
ACTATGGAGCCTGCTGCTCGTCTCGGAAACGAAGGCGAGGCCAAGGGATTCCTTGACGGACTTGTC
CAGAAATACGCCGCGAGCGCTGGTATCAGCCTGACGTCTGCGGCTGCTTCTGGTGGCTCTGAAGGT
GGCTCGGGAGGCATGATGATGGATCCCGCTGCCATTGACGCCCTCACCAAGGACCAGCGAGCTCTG
TTCAAGCAGCAGCTGGAGCTCTTTGCCCGATACCTCAAGATCGATCTCAGGTCGGGCGACAAGGCG
CATATTGAGTCTCAGAAATCAGAAACGATTCTGCAGGCCCAGATCGACCTGTGGACCGCAGAGCAC
GGCGACTTCTATGCTTCTGGTATTGAGCCCGTCTTTAGCCCATTGAAGGCGAGATCGTATGACTCGTC
TTGGAATTGGGCTCGCCAAGATGCGCTCACTATGTTCTATGACATTATTTTCGGTAGACTCAAGACTG
TCGACCGTGAAATTGTCAGCCAGTGCATCCGCATCATGAACAAGTCAAACCCCAAGCTTCTCGAGTT
CATGCAGTACCACATCGATAACTGCCCTACCGAGCGCGGTGAAACCTACCAGCTCGCCAAAGAACTT

GGCCAGCAGCTTATTGAGAACTGCAAGGAAGTCCTGGAGCTGTCTCCCGTTTACAAGGACGTTGCT
GTCCCTACTGGTCCTCGCACCACCGTGGATGCGCGTGGCAACCTCAACTATGAGGAAGTTCCTCGCG
CCAGCTGCAGAAAACTCGAGCACTACGTTCAGCAGATGGCCGAGGGCGGAAAGATTTCCGAGTATG
GCAACCGCACCAAGGTCCAGAACGACCTGCAGCGTATCTACAAGTTGATCAAGCAGCAGCACAAGA
TGTCCAAGACTTCTCAGCTCGAAATCAAGAGCCTGTACGGTGATGTTTTGCGCTCTCTGGCCATGAA
CGAGAGCCAGATCATTCCCAAGGAGAACGGCAAGGGCAGAAAGTCGGGCCTGAAGGGCACCAGCC
TGAACAAGGGCCGGGTCGAGACCATCCCATTCCTTCACCTCAAGAGAAAGACTTTGCACGGCTGGG
ACTACAGCAAGAAGCTCACTGCTGTCTACCTCAACTCTCTTGAGCAGGCTGCCAAGGACGGTGTCAC
CTTCCAGGACAAGTATGTCTTGATGACTGGTGCTGGTGCCGGATCCATTGGTGCCGAGGTTCTGCAG
GGCCTTGTCAGCGGAGGTGCCAAGGTTGTGGTTACTACCAGCCGATTCTCTCGAGAAGTCACTGAGT
ACTACCAGTCCGTTTACACCCGTTTTGGCTCTCGCGGATCTCAGATCGTTGTTGTCCCCTTCAACCAG
GGAAGCAAGCAAGACGTAGAGGCTCTCGTCAACTACATCTACGACACCAAGGCTGGTCTTGGCTGG
GATCTCGACTACATCGTTCCTTTCGCTGCCATCCCTGAGAATGGCCGACAGATCGATAGCATCGACTC
CAAGTCGGAACTGGCCCACCGTATTATGCTGACCAACTTGATCCGCATGCTCGGTTACGTCAAGTCT
CAAAAGGCTGAGCGTGGCTTCGAAACTCGTCCTGCTCAAGTTGTCCTTCCCCTGTCTCCTAACCACGG
TACCTTCGGTAACGATGGTCTCTACTCCGAGTCTAAGCTGGCTCTTGAGACTCTCTTCAACCGATGGC
ACTCTGAGGACTGGGGCCACTACCTCACAATTTGCGGTGCTGTCATTGGATGGACTCGCGGTACTGG
CCTCATGTCTGGCAACAACATTGTTGCCGAGGGTGTTGAGGCGTTTGGCGTCCGAACATTCTCTCAG
CAAGAGATGGCTTTCAACCTCTTGGGTCTGATGTCCGCTACCATTGTCGACCTCTGCCAGTCTGAGCC
AGTCTTTGCCGACTTGAACGGTGGTTTACAATTCATCCCCAACTTGAACGAGGCCATGACCAAGCTTC
GCAAGGATATCATGGAGACTAGTGAAATCCGCAGAATCGTTTCTAAGGAGAGCGCTATCGAGAACA
CTATTGTCAACGGAGCCGACTCTGAGGTTCTTTACAAGAAGAAGACCATTGAGCCTCGTGCCAACAT
CAAGTTTGACTTCCCTAATCTGCCTGATTGGAAGACTGAGGTCGCTCCTCTGAACGACCAGCTCAAG
GGCATGGTCGACTTGGAGAAGGTAGTCGTCGTCACTGGTTTCGCTGAAGTCGGCCCTTGGGGTAAC
TCCCGAACTCGATGGGAGATGGAGGCCCATGGTGAATTCTCGCTCGAAGGTTGCATTGAAATGGCC
TGGATCATGGGTCTCATCAAGAACCATAACGGTCCTCTCAAGGGCAAGCCTTACGCTGGCTGGGTTG
ACGCTAAGACTGGCGAACCTGTTGATGACAAGGACGTCAAGCAGAAGTACGAGAAATTCATTCTCG
ATCACGCCGGTATCCGTCTGATTGAGCCTGAGCTGTTTGACGGATATGACCCCAACAAGAAGCAGCT
GCTTCACGAGGTTGTCATTGAAGAGGACCTTGAGCCATTCGAGGCTTCCAAGGAGACTGCCGAGGA
GTTCCGACGAGAACATGGTGACAAGGTGGAGATTTTCGAGATCCCCGAGTCTGGAGAGTTCACTGT
CCGAGTGAAGAAGGGCGCCTCGCTCTGGATCCCCAAGGCTCTGCGCTTCGACAGACTCGTCGCTGG
TCAGATCCCAACTGGCTGGGACCCCAAGCGATATGGTATCCCTGACGATATTGTCAGCCAAGTGGAT
CCTGTCACCTTGTTCCTTCTCGTGTCGGTTGCTGAGGCCCTCCTGTCTTCTGGTATCACCGACCCATAC
GAGTTCTACAAGTATGTCCATGTCTCTGAAGTTGGTAACATTGTCGGCTCTGGTATGGGTGGTGCTT
CGGCCCTGCGTGGCATGCACAAGGCCAGATTCCTCGACAAGCCTCTTCAGAACGATATCTTGCAGGA
GTCCTTCATCAACACCATGCCTGCTTGGGTTAACATGTTACTGCTCTCTTCATCTGGACCTATTAAGAC
CCCTGTCGGTGCTTGTGCCACTGCTATTGAGTCTGTTGACATTGGTGTGGAGACCATTTTGGAAGGC
AAGGCTCGCATCTGTCTTGTCGGTGGTCTCGACGACTTTGGTGAGGAAGGCTCTTACGAGTTCGCCA
ACATGAAGGCCACCAGCAACTCATTGGATGAGTTTGCCCATGGCCGCACTCCTGGCGAAATGTCACG
TCCTACCACTACTACCCGAAACGGCTTTATGGAGTCTCAGGGATGTGGTGTCCAGGTCATCATGACC
GCCAAGCTTGCTCTGGATATGGGAGTCCCTATCCACGGTATCCTCGCTTACACCACCACTGCCTCTGA
CAAGATCGGACGTTCCGTCCCTGCTCCTGGCAAGGGTGTTCTCACGTCTGCGCGTGAGCATGCTGGC
AAGTTCCCTTCGCCACTGCTTGACATCAACTACCGTCGCCGACAGATTGAGCGCCGCAAGAAGCAGA
TTAAGCAGTGGGAAGAGTCTGAGCTGGAGTTCTTGCATGATGAAATCGATGCTATGAAGGCTCAGG
GCGGTGCCTTTGACGAGAAGGAGTACGCCCAGGACCGCGTCGCACACATCCAGAGGGAGGCCGGG
AGACAAGAAAAGGAGCTCCTCCGAAGCATGGGCAACAACTTCTGGAAGAGCGATCCATCCATCGCT
CCCCTCCGTGGTGCTCTTGCAACATGGGGCCTTACCATTGACGACCTGAAGGTTGCCTCTTTCCACGG

TACATCTACCGGTGCCAACGACAAGAACGAATCCTCTGCCGTTTGCCAGCAGCTGCGTCACCTTGGC
AGAAGCAAGGGTAACGCCGTCCTGGGTGTCTTCCAGAAGTTCCTCACTGGTCACCCCAAGGGTGCT
GCTGGTGCTTGGATGTTGAACGGTGGTCTGCAAATTCTGAACACTGGTTTGGTCCCTGGTAACAGAA
ATGCCGACAACATCGACCCCATCATGGAAGAATACGACCTGATCGTTTACCCCAGCCGTAGCATCCA
GACTGATGGAGTCAAGGCTTTCTCTCTCACTTCGTTCGGTTTCGGACAGAAGGGAGCCCAGGCAGTT
GCTGTCCACCCCAGATACCTGTTTGCAACTCTCGACCAAAAGACTTACCAGGAGTACTGCGCCAAGG
TTGAGGCTCGACAGAAGAAGGCTTACCGTTTCTTCCACAACGGCATGATCAGCAACACTCTGTTCGT
CCCCAAGGCCCACGCTCCTTACACTGATGAGCAGCTCAGCGAGGTTCTGATGAACCCTGATGCTCGT
GTCACCGAAGACAAGAAGACAAAGGAGCTCAAGTATGCCGAGAATTTCATGAAAGCGTCCGAAAA
AGTTGTCCCTGCAGTCACCGTCAAGGAGACACAGCAGGTCATTGAGGCACTCGCTCACAAGGTTACA
AGCAAGAACAGCAATGTTGGTGTAGATGTCGAGGATATCTCTGCCATCAACATTGAGAATGACACTT
TCATTGAGCGCAACTTTACTAGCCAGGAGATTGCTTACTGCAAGAGTTCTGCTAGCCCCCAGAGCTC
ATTCGCTGGCCGATGGAGCGCCAAGGAAGCAGTCTTCAAGTCTCTTGGCGTCGCTAGCAAGGGAGC
CGGTGCTTCCTTGAAGGATATTGAGATCCTCAAGGATGATTCCGGTGCACCCACTGTTACTCTTCATG
GCGATGCGGCTGCTGCTGCTAAGCAGGCTGGTGTGAAGGAGGTTTCCGTCTCCATCTCCCATGCTGA
TAAGCAGGCTGTTGCCGTGGCAGTTGCTCACTTCTAA
>Trichoderma harzianum
ATGCGTCCCGAAGTCGAGCAAGAGCTCGCCCACACGCTCCTCGTCGAGCTTCTGGCATACCAGTTCG
CCTCTCCCGTGAGGTGGATTGAGACCCAGGATGTCTTCCTGGCAGAGAGAACAGCCGAGCGCATCG
TCGAAGTCGGCCCCGCAGATACCCTTGGAGTCATGGCAAAGCGCACACTGGCATCCAAGTACGAGG
CCTACGACGCCGCCAAGTCGGTTCAGCGACAGATCCTCTGCTACAACAAAGACGCCAAGGAGATCT
ACTATGATGTAGACCCCGTTGAGGAAGAGCCCGAGCCTGTTGCCAGCTCATCCGCTGCTCCCACAAG
TCAACCCGCTGCTGCCAGCGCTCCTGCGGCTGCTGCGCCAGTTGCTGCTGCTCCCGCTGCCGGCGCT
GGACCTGCAGCTCAGGTTCCCGATGTGCCTGTCCAGGCTGTCGAGATTGTGCACACCTTGGTTGCAC
AGAAGCTCAAGAAGTCTCTAGCTGATGTGCCTCTTAGCAAGGCCATCAAGGATCTGGTTGGAGGCA
AATCCACTCTTCAAAATGAGATTCTCGGTGACCTTGGAAAGGAATTCGGCTCAACACCTGAGAAGCC
CGAAGATACACCTCTCGACGAGCTCGCCGCCTCTATGCAGGCTACCTTCGATGGCAACCTTGGCAAG
CACACAGTATCCCTCATCGCTAGGCTCATCTCCTCCAAGATGCCTGGTGGCTTCAACATCACAGTGGC
CAGGAAATATCTGGAAACAAGATGGGGTCTCGGCCCCGGCAGGCAAGACGGAGCATTGCTGCTGG
CCTTGACCATGGAGCCTACTGCCCGTCTAGGAAACGACGGTGAGGCCAAGGGATTCCTCGACGGAA
TTGTCCAGAAATACGCCGCGAGCGCCGGTATCAGCTTGACATCTGCAGCTGCTTCTGGTGGCTCTGA
AGGAGGTGCGGGAGGCATGATGATGGATCCCGCCGCCATCGATGCTCTCACCAAGGACCAGCGCG
CCCTGTTCAAGCAGCAGTTGGAGCTCTTTGCCAGATACCTCAAGATGGACCTCCGGGCCGGCGACAA
GGCACACATTGACTCCCAGAAATCAGAAAAGGTTCTCCAAGCTCAGCTGGATCTGTGGACCGCCGA
GCACGGCGATTTCTACGCTTCTGGTATCGAGCCTGTCTTTAGCCCGCTCAAGGCTAGATCCTACGACT
CCTCCTGGAACTGGGCTCGTCAGGATGCGCTCACCATGTTCTACGACATTATCTTTGGTAGACTCAAG
ACCGTCGACCGTGAAATTGTCAGCCAGTGCATTCGCATCATGAACAAGTCAAATCCCAAGCTCCTCG
AGTTTATGCAGTACCACATCGACAACTGCCCTACTGAGCGCGGTGAGACCTACCAGCTGGCCAAGG
AGCTTGGTCAGCAGCTCATTGAGAACTGCAAGGACGTTCTGAACCTTTCCCCCGTCTACAAGGACGT
TGCTGTCCCCACTGGTCCTCGCACCACTGTGGACGCTCGTGGCAACCTCAACTACGAAGAGGTTCCC
CGCGCCAGCTGCAGAAAGCTCGAGCACTACGTTCAGCAGATGGCTGAGGGTGGAAAGATTTCCGAG
TATGGCAACCGCACCAAGGTGCAAAACGACCTGCAGCGTATCTACAAGTTGATCAAGCAGCAGCAC
AAGATTTCCAAGACTTCTCAGCTCGAGATCAAGAGCCTGTACGGCGATGTTCTGCGCTCTCTGGCCA
TGAACGAGAGCCAGATCCTCCCCAAGGAGAATGGCAAGGGCCGAAAGCCAGGTCTCAAGGGCACA
AACCCCAACAAGGGCCGAGTCGAGACCATCCCCTTCCTCCACCTCAAGAGAAAGACCTTGCACGGCT
GGGACTACAGCAAGAAGCTCACTGCTGTCTACCTCAACTGTCTTGAGGAAGCGGCCAAGGATGGTG
TCACCTTCCAGGACAAGTATGTCCTGATGACTGGTGCCGGTGCCGGTTCCATTGGTGCCGAGGTCCT

GCAGGGCCTCATCAGCGGAGGTGCCAAGGTTGTGGTTACCACTAGCCGATTCTCTCGACAGGTGAC
CGAGTACTACCAGTCCATGTACAGCCGCTTTGGCTCTCGCGGATCGCAAATCGTCGTCGTTCCCTTCA
ACCAGGGAAGCAAGCAGGATGTGGAGGCTCTTGTCAACTACATCTACGATGCCAAGACCGGTCTCG
GCTGGGATCTCGACTTCATTGTTCCCTTCGCTGCCATTCCTGAGAACGGCCGACAGATTGATAGCATC
GATTCCAAGTCGGAGCTGGCTCACCGTATTATGCTCACCAACTTGATCCGCATGCTTGGCTGCGTCA
AGACTCAAAAGGCTGAGCGTGGCTTCGAGACCCGTCCCGCCCAGGTCGTCCTCCCTCTGTCCCCCAA
CCACGGTACCTTCGGTAACGATGGTCTCTACTCCGAGTCCAAGCTGGCTCTTGAGACCCTCTTCAACC
GATGGCACTCCGAGGACTGGGGCCACTACCTCACCATTTGCGGTGCTGTCATCGGATGGACTCGTG
GTACTGGTCTCATGTCTGGCAACAACGTTGTTGCTGAGGGTGTGGAGGCGTTCGGCGTGCGAACCT
TCTCCCAGCAAGAGATGGCCTTCAACCTGTTGGGCCTGATGTCAGCTACCATTGTTGATCTCTGCCAG
TCAGAGCCCGTCTTTGCCGACTTGAACGGTGGCCTGCAATTCATCCCCAACCTGAACGAGTCCATGA
CCAAGCTCCGCAAGGATATCATGGAGACTAGCGAAATCCGCAGAGCCGTTACCAAGGAGAGCGCTA
TCGAGAACACCATTGTTAACGGAGCCGACTCCGAGGTTCTTTACAAGAAGAAGACTATCGAGCCCCG
TGCCAACATCAAGTGTGACTTCCCTCACCTGCCTGACTGGAAGACGGAGGTGGCCCCTCTCAACGAC
CAGCTGAAGGGCATGGTTGACTTGGAGAAGGTCATTGTTGTGACCGGTTTTGCGGAAGTTGGTCCC
TGGGGTAACTCTCGAACTCGATGGGAGATGGAGGCCTATGGCGAGTTTTCGCTCGAAGGTTGCATC
GAAATGGCCTGGATCATGGGCCTTATCAAGAACCACAACGGTCCCCTCAAGGGCAAGCCCTATGCT
GGCTGGGTTGACGCCAAGACTGGCGAGCCCGTCGATGACAAGGATGTCAAGCAGAAGTACGAGAA
GCACATCCTGGAGCACTCCGGTATTCGTTTGATCGAGCCCGAGCTGTTCGACGGATATGACCCCAAC
AAGAAGCAGCTGCTCCACGAGGTCGTTATCCAAGAGGACCTGGAGCCTTTCGAGGCTTCCAAGGAG
ACTGCAGAGGAATTCCGACGCGAGCATGGTGACAAGGTGGAGATCTTCGAGATCCCCGAGAGTGG
AGAGTACATTGTCCGGGTGAAGAAGGGCGCCTCACTCTGGATCCCCAAGGCTCTGCGCTTTGACAG
ACTCGTCGCCGGTCAGATCCCGACTGGCTGGGACCCCAAGCGATATGGTGTCCCCGAGGACATCAT
CAGCCAGGTGGACCCTGTCACCCTGTTCCTTCTCGTGTCGACTGCCGAGGCTCTGCTGTCGTCTGGTA
TCACCGACCCCTATGAGTTCTACAAGTACGTTCACGTCTCTGAGGTTGGTAACATTGTCGGCTCTGGT
ATGGGTGGTGCTGCAGCCCTTCGTGGCATGCACAAGGCCCGATTCCTCGACAAGCCTCTTCAGAACG
ACATCCTGCAGGAGTCTTTCATCAACACCATGGCTGCATGGGTCAACATGTTGCTGCTCTCTTCGTCT
GGACCTATCAAGACGCCTGTCGGCGCTTGTGCTACTGCTATTGAGTCTGTCGACATTGGTGTGGAGA
CTATTCTCGAAGGCAAGGCACGCATCTGTCTCGTTGGTGGTTTCGACGACTTTGGTGAGGAAGGCTC
TTATGAGTTCGCCAACATGAAGGCCACCAGCAACAGTGTGGATGAGTTTGCCCATGGCCGTACACCC
GGTGAAATGTCACGACCTACTACCACTACCCGAAACGGCTTCATGGAGTCTCAGGGATGTGGTGTCC
AGGTCATCATGACTGCCCAGCTCGCTCTGGACATGGGTGTCCCGATCCACGGTATCCTTGCCTTTACC
ACCACCGCTTCCGACAAGATTGGACGATCCGTCCCTGCGCCTGGCAAGGGTGTCCTCACTTCTGCCC
GAGAGCATGCTGGCAAGTTCCCATCGCCGCTGCTTGATATCAACTACCGCCGCCGACAGATTGAGCG
CCGCAAGAAGCTGATCAAGCAGTGGGAGGAGTCTGAGTTGGAGTTCCTGCACGATGAGGTTGATG
CTATGAAGGCTCAGGGCGGTAAATTCGACGAGAAGGAGTACGCTCAGGACCGCATGCTGCACATCC
AGAAGGAGGCCGCAAGACAAACAAAGGAGCTCCTCCGAAGCATGGGTAACAACTTCTGGAAGAGT
GACCCAAGCATCGCTCCTCTCCGAGGTGCCCTTGCAACCTGGGGTCTTACCATTGATGATGTCAAGG
TGGCCTCGTTCCACGGCACATCCACTGGCGCCAACGACAAGAACGAGTCTGCGGCCATCTGCCAGCA
GCTGCGTCACCTTGGCCGAAGCGAGGGCAATGCCATTATGGGTGTCTTCCAAAAGTTCCTTACTGGT
CACCCCAAGGGTGCTGCCGGTGCTTGGATGTTGAACGGCGGCCTGCAGATCCTCAACTCTGGATTG
GTCCCTGGTAACAGAAACGCCGACAACATTGACCCCATCATGGAGCAATACGACCTGATTGTCTACC
CCAGCCGTAGCATCCAGACTGACGGAGTCAAGGCCTTCTCCCTGACCTCGTTTGGTTTCGGACAAAA
GGGAGCCCAGGCAGTCGGTGTTCACCCCAAGTATCTGTTCGCTACCCTCGACGAGAAGACTTACGAT
GCGTACCGCGTCAAGGTCGAGGCCCGACAGAAGAAGGCCTACCGCTACTTCCACAACGGAATGATT
AGCAACACCCTGTTTGTCTCCAAGGCCAACGCGCCTTACAGCGATGACCAGCTCAGCGCTGTGCTGC
TGAACCCCGATGCTCGTGTGAGCGAGGATAAGAAGACGGCGGAGCTTAAGTATGCCGCCAACTTCA

TGAAGCAGTCTGAGAAGATCACTCCCGCGACCACCGTCAAGGAGACTCAGCAGGTCATCGAAGCGC
TCGCGCACAAAGTGACGAGCAAGAACAGCAACGTCGGCGTTGATGTCGAGGATATTCCATCCTTCA
ACATTGACAATGACACGTTCATTGAGCGTAACTTCACCACCCAAGAAGTGGCCTACTGCAAGACTGC
CCCTAGCCCGCAGAGCTCATTTGCTGGCCGATGGAGTGCCAAGGAGGCCGTGTTCAAGGCCCTTGG
TGTTGCCAGCAAGGGAGCTGGCGCTGCCTTGAAGGACATTGAGATTCTCAAGGACGACACCGGCGC
GCCTGTTGTTACTCTCCATGGTGAAGCCGCCGCCGCTGCCAAGCAGGCTGGTGTCAAGGAGGTCTC
GGTCTCCATTTCACACGCCGACAAGCAGGCCGTTGCCGTAGCAGTTGCCCATTTCTAA
>Trichoderma parareesei
ATGCGTCCCGAAGTCGAGCAGGAGCTCGCCCACACGCTCCTCGTCGAGCTTCTGGCATACCAATTCG
CCTCGCCCGTGAGATGGATTGAGACCCAGGATGTCTTCCTGGCTGAGAGAAACGCCGAGCGCATCG
TCGAAATCGGCCCCGCAGACACCCTTGGAGTCATGGCCAAGCGCACACTGGCCTCCAAGTACGAGG
CCTACGATGCCGCAAAGTCGGTTCAGCGACAGATTCTCTGCTACAACAAGGATGCCAAGGAGATTTA
CTACGATGTCGACCCCGTGGAAGAAGAACCCGAACCCGCGGCGAGCTCATCTGCTGCCCCCGCTGC
CCCGGCCGCTGCCGGCGCTCCTGCGGCTGCTGCTGCTCCAGTTGCTGCTGCTCCGGCTCCCAGCGCT
GGACCTGCAGCCCAGGTCCCCGACCAGCCTGTGCAGGCCGTCGAAATTGTGCACACCCTGGTTGCG
CAAAAGCTCAAGAAGTCCCTGGCTGAGGTGCCTCTCAGCAAGGCCATCAAGGATCTGGTTGGAGGC
AAATCCACCCTTCAGAATGAGATTCTCGGTGACCTTGGAAAGGAATTCGGCACAACGCCTGAGAAG
CCCGAAGACACACCCCTCGACGAGCTCAGTGCTTCCATGCAGGCTACCTTCGATGGCAACCTGGGCA
AGCACACACAGTCCCTCATCGCCAGGCTCATCTCCTCCAAGATGCCTGGTGGCTTCAACATAACCGT
GGCAAGGAAATATCTGGAAACAAGATGGGGCCTCGGTCCCGGCAGGCAAGACGGAGTGTTGCTAC
TGGCCTTGACCATGGAGCCTCCTGCTCGTCTTGGAAACGATGGTGATGCCAAGGCTTACCTCGACGG
AGTTACCCAGAAATACGCCGCGACTGCCGGTATCAGCTTGACGTCTGCGGCCGCATCTGGTGGCTCT
GAGGGAGGCGCGGGAGGCATGATGATGGACCCCGCTGCCATTGACGCTCTCACCAAGGACCAGCG
CGCTCTGTTCAAGCAGCAGCTGGAGCTCTTCGCCAGATACCTCAAGATGGACCTCAGAGCCGGCGA
CAAGGCGCACATCGAATCCCAAAAGTCAGAAAAGGTCCTTCAGGCCCAGCTGGATCTGTGGACTGC
AGAGCACGGCGACTTCTACGCTTCGGGCATTGAGCCTGTCTTCAGCGCATTGAAAGCTAGATCTTAC
GACTCATCCTGGAACTGGGCTCGCCAAGATGCGCTCTCCATGTACTACGATATCATCTTTGGCAGACT
CAAGACGGTCGACCGTGAGATTGTCAGCCAGTGCATTCGCATCATGAACAAGTCCAACCCCAAGCTC
CTCGAGTTCATGCAGTACCACATCGACAACTGCCCCACTGAGCGCGGCGAGACCTACCAGCTCGCCA
AGGAGCTTGGACAGCAGCTCATTGAGAACTGCAAGGAGGTTCTCAACCTGCCACCTGCTTACAAGG
ACGTTGCTGTCCCCACTGGTCCTCGCACCACCGTGGACGCCCGCGGCAACCTCAACTACGAGGAAGT
CCCTCGCGCCAGCTGCCGGAAGCTCGAGCACTACGTTCAGCAGATGGCCGAGGGCGGCAAGATCTC
CGAGTATGGCAACCGCACCAAGGTGCAGAACGACCTGCAGCGTATCTACAAGTTGATCAAGCAGCA
GCACAAGATGTCCAAGACTTCTCAGCTCGAGATCAAGAGCCTGTACGGTGATGTTCTGCGCTCCCTG
GCCATGAACGAGAGCCAGATCATCCCCAAGGAGAATGGCAAGGGCCGAAAGCCCGGTTTCAAGGG
CTCAAGCCCGAACAAGGGCAGGGTTGAGACAATCCCCTTCCTCCACCTCAAGAGGAAGTCCCTGCAC
GGCTGGGACTACAGCAAGAAGCTCACGGCCGTCTACCTCAACTGCCTTGAGGAGGCTGCCAAGGAT
GGCGTCACGTTCCAGGACAAGTATGTCCTGATGACGGGTGCCGGTGCCGGCTCCATTGGTGCCGAG
GTTCTGCAGGGTCTCATCAGCGGAGGTGCCAAGGTTGTGGTCACCACCAGTCGATTCTCTCGACAGG
TCACCGAGTACTACCAGTCCATGTACAGCCGCTTTGGTTCCCGCGGATCTCAGATCGTCGTCGTGCCC
TTCAACCAGGGAAGCAAGCAGGACGTGGAGGCCCTCGTCAACTACATCTACGACACCAAGAACGGC
CTTGGCTGGGATCTCGACTACATCGTCCCCTTCGCCGCCATTCCGGAGAATGGCCGACAGATTGACA
ACATCGACTCCAAGTCGGAGCTGGCTCACCGCATCATGCTCACCAACCTGATCCGCATGCTCGGCTG
CGTCAAGACGCAAAAGGCTGAGCGCGGTTTCGAGACCCGTCCTGCCCAGGTCGTCCTCCCCCTGTCC
CCCAACCACGGTACCTTTGGTAACGACGGTCTCTACTCCGAGTCCAAGCTGGCTCTCGAGACCCTCTT
CAACCGATGGCACTCTGAGGACTGGGGCCACTACCTCACTATCTGCGGTGCTGTTATCGGATGGACT
CGTGGCACTGGTCTCATGTCCGGTAACAACGTGGTCGCCGAGGGCGTGGAGGCCTTTGGCGTTCGC

ACCTTCTCTCAGCAAGAGATGGCCTTTAACCTTCTGGGCCTGATGTCCGCCACCATCGTTGACCTCTG
CCAGTCCGAGCCGGTCTTTGCCGACTTGAACGGTGGCCTGCAGTTCATCCCCAACTTGAACGAGTCC
ATGACCAAGCTCCGCAAGGACATCATGGAGACAAGCGAGATCCGCAGGGCCGTCTCCAAGGAGAG
CGCCATCGAGAACACCATTGTCAACGGAGCCGACTCCGAGGTTCTTTACAAGAAGAAGACCATCGA
GCCCCGTGCCAACATCAAGTGCGACTTCCCCCACCTTCCCGACTGGAAGACGGAGGTGGCTCCTCTC
AACGAGCAGCTCAAGGGCATGGTCGACTTGGACAAGGTTGTCGTCGTGACTGGTTTCGCCGAAGTC
GGTCCTTGGGGCAACTCCCGCACCCGATGGGAGATGGAGGCCTATGGCGAGTTTTCGCTCGAGGGC
TGCATTGAGATGGCCTGGATCATGGGCCTCATCAAGAACCACAACGGTCCCCTCAAGGGCAAGCCCT
ACGCCGGCTGGGTTGATGCCAAGACTGGCGAGCCCGTCGACGACAAGGATGTCAAGCAAAAGTAC
GAGAAGCACATCCTGGAGCACTCCGGTATCCGCCTGATCGAGCCTGAGCTCTTTGACGGATACGACC
CCAACAAGAAGCAGCTGCTGCACGAGGTCGTCATCCAGGAGGACCTTGAGCCGTTCGAGGCCTCCA
AGGAGACGGCTGAGGAGTTCCGCCGCGAGCATGGCGACAAGGTGGAGATCTTTGAGATCCCCGAG
AGCGGAGAGTACATTATCCGGGTCAAGAAGGGCGCCTCGCTTTGGATCCCTAAGGCTCTGCGCTTC
GACAGACTGGTCGCTGGTCAGATCCCGACCGGCTGGGACCCCAAGCGATATGGTATTCCTGAAGAC
ATCATCAGCCAGGTGGACCCCGTCACCCTGTTCCTCCTCGTGTCGGTTGCTGAGGCCCTGCTGTCGTC
TGGTATCACCGACCCGTACGAATTCTACAAGTACGTTCACGTCTCCGAGGTCGGTAACATTGTCGGC
TCCGGTATGGGTGGCGCCAAGGCTCTGCGTGGCATGCACAAAGCGCGATACCTCGACAAGCCTCTT
CAGAACGACATCCTGCAGGAGTCGTTCATCAACACCATGTCTGCTTGGGTCAACATGCTGCTGCTCT
CTTCGTCTGGACCTATCAAGACTCCCGTCGGTGCTTGTGCTACTGCTATCGAGTCTGTTGACATTGGT
GTGGAGACGATTCTTGAAGGCAAGGCCCGCATCTGCCTGGTCGGAGGTTTCGACGACTTTGGTGAG
GAGGGTTCATATGAGTTTGCCAACATGAAGGCCACCAGCAACAGCTTGGACGAGTTCGCCCACGGC
CGTACCCCCGCCGAGATGTCACGTCCCACCACTACCACGCGAAACGGCTTCATGGAGTCTCAGGGAT
GCGGTGTCCAGGTCATCATGACTGCTCAGCTGGCCCTGGATATGGGAGTCCCGATCCACGGTATCCT
CGCCTTTACCACCACCGCCTCCGACAAGATTGGCCGATCCGTCCCTGCTCCTGGCCAGGGAGTTCTCA
CGTCCGCGCGTGAGCACCCCGGCAAGTTCCCGTCGCCCCTGCTTGACATCAACTACCGCCGCAGGCA
GATTGAGCGTCGCAAGAAGCAGATCAAGCAGTGGGAGGAGTCCGAGCTGGAATTCCTTCACGACG
AGATTGACGCCATGAAGGCCCAAGGCGGCGTCTTTGACGAGAAGGAGTATGCTCAGGAGCGCATCC
TGCACATCCAGAAGGAGGCTATCCGACAAGAAAAGGAGCTCCTCCGCAGCATGGGCAACAACTTCT
GGAAGAACGACCCCAGCATCGCTCCTCTCCGAGGCGCCCTTGCCACATGGGGCCTCACCATCGACGA
CCTCAAGGTGGCCTCGTTCCACGGCACGTCCACAGGCGCCAACGACAAGAACGAGTCCTCAGCCATC
TGCCAGCAGCTGCGCCATCTTGGCCGCAGCAAGGGCAACGCCATCCTCGGTGTCTTCCAGAAGTACC
TCACCGGTCACCCCAAGGGTGCTGCTGGTGCCTGGATGATGAACGGTGGTCTGCAAATCCTCAACAC
TGGCCTGGTCCCCGGCAACCGCAATGCCGACAACATTGACCCCATCATGGAGAACTACGACCTGATT
GTCTACCCCAGCCGCAGCATCCAGACCGACGGTGTCAAGGCCTTCTCCCTCACCTCGTTCGGTTTCGG
ACAGAAGGGAGCCCAGGCGGTCGCCGTCCACCCCAAGTACCTGTTCGCCACCCTAGACGAGAAGAC
GTACGACGCGTACCGCGCCAAGGTCGAGTCCCGACAGAAGAAGGCTTACCGCTTCTTCCACAACGG
CATGATTAACAACGCCCTGTTTGTCTCCAAGGCCAACCCGCCCTACACTGACGACCAGCTCAGCGCG
GTTCTGATGAACCCCGATGCTCGTGTGGTCGAGGACAAAAAGACGGCGGAGCTCAAGTTCCCCCCC
AACTTCATGAAGGCATCCGAGAAGACTGTCTCTCCGACCGTCGCCAAGGAGACGCAGCAGGTGATT
GAGGCGCTCGCGCACAAGGTGGCGGGCAAGAACAGCAATGTGGGCGTGGACGTCGAGGACATTG
CATCCTTCAACATTGACAACGACACGTTCATTGAGCGCAACTTTACCAGCCAGGAGGTGGCCTACTG
CAAGAGCGCTCCCAGCCCGCAGAGCTCGTTTGCCGGCCGATGGAGCGCCAAGGAGGCCGTGTTCAA
GGCTCTTGGCGTCGCCAGCAAGGGAGCTGGCGCTGCCCTGAAGGACATTGAGATCCTCAAGGACGA
CACCGGCGCACCTGTTGTTTCTCTTCATGGTGATGCTGCTGCTGCTGCCAAGCAAGCTGGCGTGAAG
GAGGTCTCCGTCTCCATCTCGCATGCCGACAAGCAGGCCGTTGCCGTGGCGGTTGCCCACTTCTAA
>Trichoderma reesei
ATGCGTCCCGAAGTCGAGCAGGAGCTCGCCCACACGCTCCTCGTCGAGCTTCTGGCATACCAATTCG

CCTCGCCCGTGAGATGGATTGAGACCCAGGATGTCTTCCTGGCTGAGAGAAACGCCGAGCGCATCG
TCGAAATCGGCCCCGCAGACACCCTTGGAGTCATGGCCAAGCGCACACTGGCCTCCAAGTACGAGG
CCTACGATGCCGCAAAGTCGGTTCAGCGACAGATTCTCTGCTACAACAAGGATGCCAAGGAGATTTA
CTACGATGTCGACCCCGTGGAAGAAGAACCCGAACCCGCGGCGAGCTCATCTGCTGCCCCCGCTGC
TCCGGCCGCTGCCGGCGCTCCTGCGGCTGCTGCTCCAGTTGCTGCTGCTCCGGCTCCCAGCGCTGGA
CCTGCAGCCCAGGTCCCCGACCAGCCTGTGCAGGCCGTCGACATTGTGCACACCCTGGTTGCGCAAA
AGCTCAAGAAGTCCCTGGCTGAGGTGCCTCTCAGCAAGGCCATCAAGGATCTGGTTGGAGGCAAAT
CCACCCTTCAGAATGAGATTCTCGGTGACCTCGGAAAGGAATTCGGCACAACGCCTGAGAAGCCCG
AAGACACACCCCTCGACGAGCTCAGTGCTTCCATGCAGGCTACCTTCGATGGCAACCTGGGCAAGCA
CACACAGTCCCTCATCGCCAGGCTCATCTCCTCCAAGATGCCTGGTGGCTTCAACATAACCGTGGCA
AGGAAATATTTGGAAACAAGATGGGGCCTCGGTCCCGGCAGGCAAGACGGAGTGTTGCTGCTGGC
CTTGACCATGGAGCCTCCTGCTCGTCTTGGAAACGATGGTGATGCCAAGGCTTACCTCGACGGAGTT
ACCCAGAAATACGCCGCGACTGCCGGTATCAGCTTGACGTCTGCGGCCGCATCTGGTGGCTCTGAG
GGAGGCGCGGGAGGCATGATGATGGACCCCGCTGCCATTGACGCTCTCACCAAGGACCAGCGCGC
TCTGTTCAAGCAGCAGCTGGAGCTCTTCGCCAGATACCTCAAGATGGACCTCAGAGCCGGCGACAA
GGCGCACATCGAATCCCAAAAGTCGGAAAAGGTCCTTCAGGCCCAGCTGGATCTGTGGACTGCAGA
GCACGGCGACTTCTACGCTTCGGGCATTGAGCCTGTCTTCAGCGCATTGAAAGCTAGATCTTACGAC
TCGTCCTGGAACTGGGCTCGCCAAGATGCGCTCTCCATGTACTACGACATCATCTTTGGCAGACTCA
AGACGGTCGATCGTGAGATTGTCAGCCAGTGCATTCGCATCATGAACAAGTCCAACCCCAAGCTCCT
CGAGTTCATGCAGTACCACATCGACAACTGCCCTACTGAGCGCGGCGAGACCTACCAGCTCGCCAAG
GAGCTTGGACAGCAGCTCATTGAGAACTGCAAGGAGGTTCTCAACCTGCCACCTGCTTACAAGGAC
GTTGCTGTCCCCACTGGTCCTCGCACCACCGTGGACGCCCGCGGCAACCTCAACTACGAGGAAGTCC
CCCGCGCCAGCTGCCGGAAGCTCGAGCACTACGTTCAGCAGATGGCCGAGGGCGGCAAGATCTCCG
AGTATGGCAACCGCACCAAGGTGCAGAACGACCTGCAGCGTATCTACAAGTTGATCAAGCAGCAGC
ACAAGATGTCTAAGACTTCTCAGCTCGAGATCAAGAGCCTGTACGGTGATGTTCTGCGCTCCCTGGC
CATGAACGAGAGCCAGATCATCCCCAAGGAGAATGGCAAGGGCCGAAAGCCCGGTTTCAAGGGCT
CAAGCCCGAACAAGGGCAGGGTTGAGACAATCCCCTTCCTCCACCTCAAGAGGAAGTCCCTGCACG
GCTGGGACTACAGCAAGAAGCTCACGGCCGTCTACCTCAACTGCCTTGAGGAGGCTGCCAAGGACG
GCGTCACGTTCCAGGACAAGTATGTCCTGATGACGGGTGCCGGTGCCGGCTCCATTGGTGCTGAGG
TTCTGCAGGGTCTCATCAGCGGAGGTGCCAAGGTTGTGGTCACCACCAGTCGATTCTCTCGACAGGT
CACCGAGTACTACCAGTCCATGTACAGCCGCTTTGGATCCCGCGGATCTCAGATCGTCGTCGTGCCC
TTCAACCAGGGAAGCAAGCAGGACGTGGAGGCCCTCGTCAACTACATCTACGACACCAAGAACGGC
CTTGGCTGGGATCTCGACTACATCGTCCCCTTCGCCGCCATTCCGGAGAATGGCCGACAAATTGACA
ACATCGACTCCAAGTCGGAGCTGGCTCACCGCATCATGCTCACCAACCTGATCCGCATGCTCGGCTG
CGTCAAGACGCAAAAGGCTGAGCGCGGTTTCGAGACCCGTCCCGCCCAGGTCGTCCTCCCCCTGTCC
CCCAACCACGGTACCTTTGGTAACGACGGTCTCTACTCCGAGTCCAAGCTGGCTCTCGAGACCCTCTT
CAACCGATGGCACTCCGAGGACTGGGGCCACTACCTCACCATCTGCGGTGCTGTCATTGGATGGACT
CGTGGCACTGGTCTCATGTCCGGTAACAACGTGGTCGCCGAGGGCGTGGAGGCCTTTGGCGTTCGC
ACCTTCTCTCAGCAAGAGATGGCCTTTAACCTTCTGGGCCTGATGTCCGCCACCATCGTTGACCTCTG
CCAGTCCGAGCCGGTCTTTGCCGACCTGAACGGTGGCCTGCAGTTCATCCCCAACTTGAACGAGTCC
ATGACCAAGCTCCGCAAGGACATCATGGAGACGAGCGAGATCCGCAGGGCCGTCTCCAAGGAGAG
CGCCATCGAGAACACCATTGTCAACGGAGCCGACTCCGAGGTTCTTTACAAGAAGAAGACCATCGA
GCCCCGTGCCAACATCAAGTGCGACTTCCCCCACCTTCCCGACTGGAAGACGGAGGTGGCTCCTCTC
AACGAGCAGCTCAAGGGCATGGTCGACTTGGACAAGGTTGTCGTCGTGACTGGTTTCGCCGAAGTC
GGTCCTTGGGGCAACTCCCGCACCCGATGGGAGATGGAGGCCTATGGCGAGTTTTCGCTCGAGGGC
TGCATTGAGATGGCCTGGATCATGGGCCTCATCAAGAACCACAACGGTCCCCTCAAGGGCAAGCCCT
ATGCCGGCTGGGTTGATGCCAAGACTGGCGAGCCCGTCGATGACAAGGATGTCAAGCAAAAGTAC

GAGAAGCACATCCTGGAGCACTCCGGTATCCGCCTGATCGAGCCTGAGCTCTTTGACGGATACGACC
CCAACAAGAAGCAGCTGCTGCACGAGGTCGTCATCCAGGAGGACCTTGAGCCGTTCGAGGCCTCCA
AGGAGACGGCTGAGGAGTTCCGCCGCGAGCATGGCGACAAGGTGGAGATCTTCGAGATCCCCGAG
AGCGGAGAGTACATTATCCGGGTCAAGAAGGGCGCCTCGCTTTGGATTCCCAAGGCTTTGCGCTTC
GACAGACTGGTCGCTGGTCAGATCCCGACCGGCTGGGACCCCAAGCGATATGGTATTCCTGAAGAC
ATCATCAGCCAGGTGGACCCCGTCACCCTGTTCCTCCTCGTGTCGGTTGCTGAGGCTCTGCTGTCGTC
TGGTATCACCGACCCGTACGAGTTCTACAAGTACGTTCACGTCTCCGAGGTTGGTAACATTGTCGGC
TCCGGTATGGGTGGTGCCAAGGCTCTGCGTGGCATGCACAAGGCGCGATACCTCGACAAGCCTCTT
CAGAACGACATCCTGCAGGAGTCCTTCATCAACACCATGTCTGCTTGGGTCAACATGCTGCTGCTCTC
TTCGTCTGGACCTATCAAGACTCCCGTCGGTGCTTGTGCTACTGCTATTGAGTCTGTTGACATTGGTG
TGGAGACAATTCTTGAAGGCAAGGCCCGCATCTGCCTGGTCGGAGGTTTCGACGACTTTGGTGAGG
AGGGTTCCTATGAGTTTGCCAACATGAAGGCCACCAGCAACAGCTTGGACGAGTTCGCCCACGGCC
GTACCCCCGCCGAGATGTCACGTCCCACCACTACCACGCGAAACGGCTTCATGGAGTCTCAGGGATG
CGGTGTCCAGGTCATCATGACTGCTCAGCTGGCCCTGGATATGGGAGTCCCGATCCACGGTATCCTC
GCCTTTACCACCACCGCCTCCGACAAGATTGGCCGATCCGTCCCTGCTCCTGGCCAGGGCGTTCTCAC
CTCCGCGCGCGAGCACCCCGGCAAGTTCCCGTCGCCCCTGCTTGACATCAACTACCGCCGCAGGCAG
ATTGAGCGTCGCAAGAAGCAGATCAAGCAGTGGGAGGAGTCCGAGCTGGAATTCCTTCATGACGA
GATTGACGCCATGAAGGCCCAGGGCGGCGTCTTTGACGAGAAGGAGTACGCTCAGGAGCGTATCCT
GCACATCCAGAAGGAGGCTATCCGACAAGAAAAGGAGCTCCTCCGCAGCATGGGCAATAACTTCTG
GAAGAACGACCCCAGCATCGCTCCTCTCCGAGGCGCCCTTGCCACATGGGGCCTCACCATCGACGAC
CTCAAGGTGGCCTCGTTCCACGGCACGTCCACGGGCGCCAACGACAAGAACGAGTCCTCGGCCATC
TGCCAGCAGCTGCGCCATCTTGGCCGCAGCAAGGGCAACGCCATCCTCGGTGTCTTCCAGAAGTACC
TCACCGGTCACCCCAAGGGTGCTGCTGGTGCCTGGATGATGAACGGTGGCCTGCAGATCCTCAACA
CTGGCCTGGTCCCCGGCAACCGCAATGCCGACAACATTGACCCCATCATGGAGAACTACGACCTGAT
TGTCTACCCCAGCCGCAGCATCCAGACCGACGGTGTCAAGGCCTTCTCCCTCACCTCGTTCGGTTTCG
GACAGAAGGGAGCCCAGGCGGTCGCCGTCCACCCCAAGTACCTGTTCGCCACCCTGGACGAGAAGA
CGTACGACGCGTACCGCGCCAAGGTCGAGTCCCGACAGAAGAAGGCTTACCGCTTCTTCCACAACG
GCATGATCAACAACGCCTTGTTTGTCTCCAAGGCCAACCCGCCCTACACTGACGACCAGCTCAGCGC
GGTTCTGATGAACCCCGATGCTCGTGTGGTCGAGGACAAGAAGACGGCGGAGCTCAAGTTCCCCCC
CAACTTCATGAAGGCGTCCGAGAAGACTGTCTCTCCGACCGTCGCCAAGGAGACGCAGCAGGTGAT
TGAGGCGCTCGCGCACAAGGTGGCGGGCAAGAACAGCAACGTGGGCGTGGATGTCGAGGACATTG
CATCCTTCAACATTGACAACGACACGTTCATTGAGCGCAACTTTACCAGCCAGGAGGTGGCCTACTG
CAAGAGCGCTCCCAGCCCGCAGAGCTCGTTTGCCGGCCGATGGAGCGCCAAGGAGGCCGTGTTCAA
GGCTCTTGGCGTTGCCAGCAAGGGAGCTGGCGCTGCCCTGAAGGACATTGAGATCCTCAAGGACGA
CACCGGCGCACCTGTTGTTTCTCTTCATGGTGATGCTGCTGCTGCTGCCAAGCAAGCTGGCGTGAAG
GAGGTCTCCGTCTCCATCTCGCATGCCGACAAGCAGGCCGTTGCCGTGGCGGTTGCCCACTTCTAA
>Trichoderma virens
ATGCGTCCCGAAGTCGAGCAAGAGCTCGCCCACACGCTCCTCGTCGAGCTTCTGGCATACCAGTTCG
CCTCTCCCGTGAGGTGGATTGAGACCCAGGATGTGTTCCTGGCTGAGAGAACGGCCGAGCGCATCG
TCGAAGTCGGCCCCGCAGATACCCTCGGAGTCATGGCAAAGCGCACACTGGCATCTAAGTATGAGG
CCTACGATGCCGCCAAGTCGGTTCAGCGACAGATCCTCTGCTACAACAAGGACGCCAAGGAGATCT
ACTATGATGTAGACCCTGTTGAGGAAGAGCCTGAGCCCGTTGCCAGCTCATCCGCTGCCCCCTCCAG
TCAACCCGCCACTGCCAGCGCTCCTGCGGCTGCTGCGCCAGTTGCTGCTGCTCCCGCCGCCAGTGCT
GGACCTGCAGCTCAGGTTCCCGATGCGCCTGTGCAGGCTGTCGACATTGTGCACACACTGGTTGCGC
AAAAGCTCAAGAAGCCTCTGACTGAGGTGCCTCTCAGCAAGGCCATCAAGGATCTGGTTGGAGGCA
AATCCACCCTCCAAAATGAGATTCTCGGTGATCTTGGAAAAGAATTCGGCTCAACGCCTGAGAAGCC
CGAAGATACGCCTCTCGACGAGCTCTCTGCTTCTATGCAGGCTACCTTCGATGGCAACCTTGGCAAG

CACACACAATCCCTCATCGCCAGGCTCATCTCCTCCAAGATGCCTGGTGGCTTCAACATCACAGTGGC
AAGGAAATATCTGGAAACAAGATGGGGTCTTGGCCCCGGCAGACAAGATGGAGCATTGCTGCTGG
CTTTGACCATGGAGCCTGCCGCTCGTCTAGGAAACGACGGTGAAGCCAAGGGATTCCTCGATGGAA
TCGTTCAGAAATACGCCGCGAATGCCGGTATCAGCCTGTCATCTGCGGCCGCTACTGGTGGCTCTGA
AGGAGGTGCGGGAGGCATGATGATGGATCCCGCCGCCATTGACGCTCTCACCAAGGACCAGCGCG
CGCTGTTCAAGCAGCAGTTGGAGCTCTTTGCCAGATACCTCAAGATGGACCTCCGGGCCGGCGACA
AGGCGCACATTGACTCCCAGAAATCAGAAAAGGTCCTCCAGGCCCAGCTGGATCTGTGGACCGCAG
AGCATGGCGATTTCTACGCTTCCGGTATCGAGCCTGTCTTTAGCCCCTTGAAGGCTAGATCCTATGAC
TCGTCCTGGAACTGGGCTCGTCAGGATGCGCTCACCATGTTCTATGACATTATCTTCGGCAGACTCAA
GACCGTCGACCGTGAAATCGTCAGCCAGTGCATTCGCATCATGAACAAGTCAAATCCCAAGCTCCTC
GAGTTTATGCAGTACCACATCGACAACTGCCCCACTGAGCGAGGCGAGACCTACCAGCTGGCCAAG
GAACTTGGTCAGCAGCTCATTGAGAACTGCAAGGAGGTTCTGAACGCATCCCCTGTTTACAAGGAC
GTTGCTGTCCCTACTGGCCCTCGCACCACGGTTGACGCTCGCGGCAACCTCAACTACGAAGAGGTTC
CCCGCGCCAGCTGCAGGAAGCTCGAGCACTACGTTCAGCAGATGGCTGAGGGTGGAAAGATTTCTG
AGTATGGCAACCGCACCAAGGTGCAGAATGACCTGCAGCGTATCTACAAGTTGATCAAGCAGCAGC
ACAAGATTTCCAAGACTTCTCAGCTCGAGATCAAGAGCTTGTACGGCGATGTTCTGCGCTCCCTGGC
CATGAACGAGAGCCAGATCCTCCCCAAGGAGAATGGCAAGGGCCGAAAGCCAGGTCTCAAGGGCA
CAAACCCCAACAAGGGCAGAGTCGAGACCATCCCCTTCCTCCACCTCAAGAGAAAGACTCTGCATGG
CTGGGACTACAGCAAGAAGCTCACTGCTGTCTACCTCAACTGTCTTGAGGAGGCGGCCAAGGATGG
TGTCACTTTCCAGGACAAGTATGTCCTGATGACTGGCGCTGGTGCCGGTTCCATTGGTGCCGAGGTC
CTGCAGGGCCTCATCAGCGGAGGTGCCAAGGTTGTCGTTACCACCAGCCGATTCTCTCGACAGGTCA
CCGAGTATTACCAGTCCATGTACAGCCGCTTTGGCTCTCGCGGATCGCAGATCGTCGTCGTTCCCTTC
AACCAGGGAAGCAAGCAAGATGTGGAGGCTCTTGTCGACTACATCTATGACACCAAGAACGGTCTT
GGCTGGGATCTCGACTTCATCGTTCCCTTCGCCGCCATCCCCGAGAACGGCCGACAGATTGATAGCA
TCGACTCCAAGTCAGAGCTGGCTCACCGTATTATGCTCACCAACTTGATTCGCATGCTCGGCTGTGTC
AAGACTCAAAAGGCTGAGCGTGGCTTTGAGACTCGTCCCGCCCAAGTTGTCCTCCCTCTGTCCCCCA
ACCACGGTACCTTCGGTAACGATGGTCTCTACTCCGAGTCCAAGCTGGCTCTTGAGACCCTCTTCAAC
CGATGGCACTCCGAGGACTGGGGCCACTACCTCACCATTTGCGGTGCCGTTATCGGATGGACTCGTG
GTACTGGTCTCATGTCTGGCAACAACGTTGTCGCTGAGGGTGTGGAAGCGTTCGGCGTGCGAACCT
TCTCCCAACAAGAGATGGCCTTTAACTTGTTGGGCCTGATGTCAGCTACTATTGTTGATCTCTGCCAG
TCAGAGCCTGTCTTTGCCGACTTGAACGGTGGCCTGCAATTCATTCCCAACTTGAACGAGTCCATGAC
TAAGCTTCGCAAGGACATCATGGAGACCAGCGAAATCCGCAGAGCCGTCAGCAAGGAGAGTGCCAT
CGAGAACACCATTGTTAACGGAGCCGACTCTGAGGTTCTTTACAAGAAGAAGACCATCGAGCCCCGT
GCCAACATCAAGTGTGACTTCCCAACCCTGCCCGATTGGAAGACGGAGGTGGCCCCTCTCAACGATC
AGCTCAAGGGTATGGTCGACTTGGAGAAGGTCGTTGTCGTGACTGGTTTTGCCGAGGTTGGTCCTT
GGGGCAACTCCCGAACTCGATGGGAAATGGAGGCCTACGGCGAGTTTTCGCTCGAAGGCTGTATCG
AAATGGCCTGGATCATGGGCCTCATCAAGAACCACAACGGTCCCCTCAAGGGCAAGCCCTACGCTG
GCTGGGTTGATGCCAAGACCGGCGAGCCCGTTGATGACAAGGACGTCAAGCAGAAGTACGAGAAG
CACATTCTGGAGCACTCTGGTATCCGTTTGATTGAGCCCGAGCTGTTTGACGGATATGACCCCAACC
AGAAGCAGCTGCTCCACGAAGTCGTCATTCAAGAGGACTTGGAGCCTTTCGAGGCTTCCAAGGAGA
CTGCGGAGGAATTCCGACGCGAGCACGGTGACAAGGTGGAGATTTTCGAGATCCCCGAGAGCGGA
GAGTACATCGTCCGCGTCAAGAAGGGCGCTTCGCTCTGGATCCCCAAGGCTCTGCGCTTCGACAGAC
TCGTCGCTGGTCAGATCCCAACTGGTTGGGATCCCAAGCGATATGGTGTCCCCGAAGACATCATCAG
CCAAGTGGACCCTGTCACCCTGTTCCTACTCGTGTCAGTTGCCGAGGCCCTCTTGTCGTCTGGTATTA
CCGACCCATATGAGTTCTACAAGTACGTTCACGTCTCTGAGGTTGGTAACATTGTCGGCTCTGGTATG
GGTGGTGCCAAGGCCCTGCGCAGCATGCACAAGGCCCGATACCTCGACAAGCCTGTTCAGAACGAT
ATCTTGCAGGAATCTTTCATCAACACCATGGCTGCATGGGTCAACATGTTGCTGCTCTCTTCCTCTGG

ACCTATCAAGACCCCTGTCGGCGCTTGTGCTACCGCCATTGAATCTGTCGACATTGGCGTGGAGACG
ATTCTCGAAGGCAAGGCACGCATCTGCCTTGTTGGTGGCTTCGACGACCTTGGCGAGGAGGGTTCTT
ATGAGTTTGCCAACATGAAGGCTACCAGCAACACTGTGGACGAGTTCGCCCATGGCCGTACACCCG
GCGAAATGTCGCGTCCTACCACCACTACCCGAAACGGCTTTATGGAGTCTCAGGGATGCGGTGTCCA
GGTCATCATGACTGCTCAGCTCGCTCTGGACATGGGAGTCCCTATTCACGGTATCCTTGCCTTCACCA
CCACCGCCTCTGACAAGATTGGACGATCCGTCCCTGCACCTGGCAAGGGTGTCCTCACATCTGCACG
AGAGCACGCCGGCAAGTTCCCATCGCCGCTGCTTGATATCAACTACCGCCGCCGCCAGATTGAGCGT
CGCAAGAAGCTGATCAAGCAGTGGGAAGAGTCTGAGTTGGAATTCCTGCACGATGAGGTTGATGCT
ATGAAGGCCCAGGGTGGTGCCTTTGACGAGAAGGAGTACGCTCAGGATCGCATGCTGCACATCCAG
AAGGAGGCCGCAAGACAAGAAAAGGAGCTCCTCCGAAGCATGGGCAACAACTTCTGGAAGAGCGA
CCCAAGCATCGCTCCACTCCGAGGTGCCCTTGCAACCTGGGGCCTTACCATTGACGACCTCAAGGTG
GCCTCGTTCCACGGCACATCCACTGGCGCCAACGACAAGAACGAGTCCGCGGCCATCTGTCAGCAG
CTGCGTCACCTTGGCCGAAGCGAGGGCAATGCTATCATGGGTGTCTTCCAAAAGTTCCTTACTGGTC
ACCCCAAGGGTGCTGCTGGTGCTTGGATGTTGAACGGCGGCCTGCAGATCCTCAACACTGGAGTGG
TCCCTGGTAACAGAAATGCCGACAACATTGACCCCATCATGGAGCAATACGACCTGATTGTCTACCC
GAGCCGTAGCATCCAGACCGACGGAGTCAAGGCTTTCTCCTTGACCTCATTTGGTTTCGGACAGAAG
GGAGCTCAGGCAGTCGCCGTTCACCCCAAGTATCTGTTCGCTACCCTCGACGAGAAGACTTACGATG
CGTACCGCGTCAAGGTCGAGGCCCGACAGAAGAAGGCCTACCGCTTCTTCCACAATGGAATGATCA
GCAACACCCTGTTTGTCTCCAAGGCCAATGCTCCTTACACTGACGAGCAGCTGAGCGCTGTTCTGCT
GAACCCTGATGCTCGTGTGACCGAGGATAAGAAGACAGCAGAGCTCACATATGCGGCCAACTTCAT
GAAGGCGTCTGAGAAGGTCACTCCTGCGGCCACTGTCAAGGAGACGCAGCAGGTCATCGAGGCGC
TCGCTCACAAGGTGACGAGCAAGAACAGCAACGTGGGCGTTGATGTCGAGGATATTCCCTCATTCA
ACATTGACAATGACACTTTCATTGAGCGCAACTTCACCAGCCAGGAAGTGGCCTACTGCAAGACTGC
TCCTAGCCCGCAGAGCTCGTTCGCCGGCCGATGGAGCGCCAAGGAAGCCGTGTTCAAGGCTCTTGG
TGTTGCCAGCAAGGGAGCTGGCGCTGCCTTGAAGGACATTGAGATTCTCAAGGACGATACTGGCGC
ACCTGTCGTTACTCTTCATGGTGAAGCGGCTGCTGCTGCTAAGCAAGCCGGTGTTAAGGAGGTCTCG
GTCTCCATTTCACACGCCGATAAGCAGGCCGTTGCCGTGGCAGTTGCCCATTTTTAG

Appendix S4. *lcb2* cds retrieved from the genomes of the 13 species analyzed in the present work

>Trichoderma arundinaceum
ATGCCGCGGCGGCTCGCAAACCCCTTCTCCTCTAGTCAGTCTGCCATGTCGGTGCCCGAAAAGACTG
GCGAAAGACGTCTCTCTGCCTCAAAGACCAACCGACTGAGCCAGCTCTTCTCGTCCAGCCCAAAGTC
CAAGGACCAGGCATCCCTAGCCACCATGCATCAAAGCCAGATGAGCACCATCTCGCCGTCGACGGT
GTCGCTGCCCACCATCTCCCTCTCCACCGCGACGGGCGAGTCCAACAACGTCGAGCCAACCACGACG
CTGTTCAAGCCTCAGTCTGCTGAAGAGGTCGAGCTGCGGCGACGCGTGGAGGCTCAATTCGCTCCTC
TGCTCGATCCGGCCCACCTCTACGTCAGCCAGTCTCACGGCCGGCCCTTCCAGCGGCCGATTGAGGA
CGAGCCTCCGTACTACTTTCTTTTGACCACGTACCTGAGTTATCTTGCTCTTATTCTGTTTGGCCACAT
TCGTGATTTCTTTGGCAAGCGGTTCGGCGACCGCAAGCGCTACCTGCCCCTCAAGGTGCACAATGGG
TTTGCACCCCTCAATGATGATTTCGACAGCTTCTACACCAGACGCTTGAAGCTCCGCTTGGACGACTG
CTTTGCCCGAACCACCATTGGTGTCCCCGGCCGCTACATCACCCTCCTGGACCGGAAATCGGAAGAC
TACAATTACACTTACCGCTACACCGGCACCCACACTGAGACGCTAAACATGAGTTCCTACAACTACCT
CGGTTTCGCTCAGTCAGAGGGTCCCTGCGCCGATGCCGTCGACGAGTGCGTCAAGAAGTATGGTGT
CACCTTTGGAAGCCCCCGATCTGAGAGCGGCACTTCCGATCTGGCCGTTGAGGTCGAGCGGGAAAT
TGCGTCCTTTGTTGGAAAGCCCGACGCCATGGTCTTTTCCATGGGCTACGTCACCAACGCCAGCACG
TTCCCTGCGCTTGTGTCCAAGGGCTGCCTCATCATCTCCGACGAACTGAACCACGCCTCTATCCGAAT
TGGTGCTCGTCTCTCCGGTGCCGTCATCCAGTCTTTCAAGCACAACGACATGGACGCCCTGGAGCGC
GTTCTCCGTGAAGCCATCTCTCAAGGCCAACCGAGGACTCACCGCCCCTGGAAGAAGATTCTCGTGG
TTGTCGAAGGTCTCTACTCCATGGAAGGCAGCATGGTCGACCTGCCCGGTATCCTGGCTCTCAAGAA
GAAGTACAAGTTCTACCTGTTCATTGACGAGGCTCACTCCATCGGTGCCCTGGGACCCCGTGGCCGC
GGCGTCTGCGATTACTTTGGCATTGATCCTGCGGAAGTCGACATTCTCATGGGCACCCTGACCAAGT
CTTTCGGCGCCAACGGAGGCTACGTTGCTGCGGACAAGCACATTGTCGATAAGCTCCGTGCCACCAA
TGCCTCTACCATCTTCGGCGAAGCTCCTGCCCCCTGTGTTCTCATGCAGATTCTCACGTCGCTGAGGC
TGATTACTGGAGAAATCTGCCCTGGTCAAGGTGAAGAGCGTCTGCAGCGCATTGCCTTCAACTCTCG
TTATCTTCGTCTCGGACTTAAGCGTCTCGGCTTAATTGTGGCTGGTCACGACGATTCCCCCATCATCC
CCGTCCTGCTTTACAACCCTGGAAAGATGCCTGCCTTCAGCCGCGAGATGCTCAAGCGTAAAATCTC
CGTCGTCGTCGTCGGCTACCCGGCCACTCCGCTCATCAGCTCGCGTGTTCGCTTCTGCATCTCTGCCG
CCCACAACAAGGATGATCTTGACCGGATGCTCATCGCCTGCAACGAGGTGGGCGATCTGCTCCAGCT
CAAGAACTCCACGGGAATTGCCGGTGGCTTGGAGCCGCTGCCCGAGGGTGTGGCGCCAGAAGACG
AGGCCGAGTGGAGGAGGGTCAACAACATCCCAATCAAGGCCCCTCGATGGGACGTCAAGGAGGTC
ATTCGACGAGGTGTCGTCGACTGCAAGCTTCCTCTGCGGTAA
>Trichoderma asperellum
ATGCCGCGACGGCTTGCAAACCCCTTCTCCTCTACCCAGTCTGCTATGTCAGCATCCGAGAAGACTG
GCGAAAGACGTCTCTCGTCCTCCAAGACCAACCGACTGAGCCAGCTGTTCTCATCCAGCCCAAAGTC
CAAGGACCAGTCGCCTCTCGCCGCCATGCATCAAAGCCAGATGAACAACACCATCTCGCCGTCGACG
GTGTCGCTGCCCACCATTTCCCTGTCCACCGCGACTGGGGAGCCCAACAACGTCGAGCCAGCCAAGA
TGCTGTTCAAGCCTCAATCGGCCGAGGAGCAGGAGCATCGACGACGCACCGAGGCCCAGTTTGGAC
CTCTGCTTGACCCATCCCACCGCTATGTTAGCAAGTCGAATGGCGAAGAGTTCAAGGAGCCGATTGA
GGACATGCCCCCCTACTTCTTTCTCTTGACCACCTACATAAGCTATCTCCTCTTGATCATCTTTGGCCAT
TGTCGCGATTACTTTGGCAAACGTTTTGGCGATAAAAAGCGCTACAATGCCCTCAAAGTGCGAAACG
GGCTTGCGCCTCTCACTGATGATTTCGACAGCTTCTACACCCGACGCTTGAAAGGCCGTCTGGATGA
CTGCTTTGCTCGACCTACTTTTGGTGTTCCCGGCCGCTTCATCACTCTCAAGGAACGTACGGCAGACA
GGCTTAACCGCCACTATCACTACACTGGTAACCACATTGAGACGCTCAACGTGAGTTCGTACAACTAT
CTCGGCTTTGCTCAATCCGAGGGTCCCTGTGCCGACGCTGTCGATGAATGTGTCAAGAAGTACGGCG

TTACCGCTGCAAGCCCCCGTGGTGACAGCGGCACTTCCGACTTGGCCCTTGAAGTCGAGCGCGAAG
TTGCGGCCTTTGTTGGAAAGCCAGATGCCATGGTCTTCTCTATGGGTTATGTTACCAACTCCAGTACT
TTCCCTGCTCTCGTGTCCAAAGGCTGCCTGATCATCTCTGACGAACTGAACCACGCCTCCATCCGTGT
CGGTGCTCGTCTCAGTGGCGCCGTCATCCAGTCGTTCAAGCATAACGATATGACCGCTCTGGAGCGT
GTTCTTCGTGAAGCCATCTCTCAGGGCCAGCCAAGGACTCACCGCCCCTGGAAGAAGATTCTTGTCG
TTGTCGAAGGTCTCTACTCTATGGAAGGTACCATGGTTAATCTACCCGCAATTGTGGCCCTCAAGCAC
AAGTACAAGTTCTACTTGTACGTTGACGAAGCCCACTCCATCGGTGCCCTAGGACCCCGTGGCCGTG
GTGTCTGTGATTACTTTGGCATTGACACCTCGGAGGTTGACATCCTTATGGGTACTCTGACCAAGTCG
TTTGGCGCCAATGGAGGCTACATTGCGGCTGAGAAGCACATCATTGACAAGCTCCGTGCCACTAATG
TCTGCTCAATCTACGGCGAAGCTCCTTCTCCTTTTGTCCTCATGCAGATTCTTACTTCGATCAAGCTGA
TTAACGGCGAGATTTGCCCTGGAGAGGGCGAGGAGCGCCTCCAGCGAATCGCCTTCAACTCCCGCT
ATCTTCGTCTTGGACTCAAGCGTCTTGGCTTGATCGTTGCTGGCTCTGATGACTCTCCCATTATCCCCG
TCCTCCTGTACAACCCCGGAAAGATGCCTGCCTTCAGCCGCGAAATGCTCAAGCGCAAAATCTCCGT
CGTCATTGTTGGTTACCCGGCTACCCCGCTCATCAGCTCGCGTGCCCGCTTCTGCATCTCTGCCGCTC
ACAACAAGGATGATCTTGACCGAATGATCAGGGCCTGTGATGAGGTTGGCGACATGCTTCAGCTCA
AGTTCTCATCAGGCATTGCCGGCGGCTTGGAGCCACTGCCTGCTGGTGTGGCACCAGAAAATGAGG
CTGAGTGGAGGAAGGCCAACAACGTCCCCATCAAGCCTCCTCGATGGGATGTTGAGGAGGTCATTC
GACGAGGCGCCGCCGATTGCAAGCTTCCTCTGCGATAA
>Trichoderma atrobrunneum
ATGCCGCGACGGCTTGCAAATCCCTTCTCCTCCAGCCAGTCTGCCATGTCAGCGTCCGAAAAGACTG
GCGAACGACGTCTCTCAACCTCAAAGACCAACCGACTGAGCCAGCTCTTCTCGTCCAGCCCAAAGTC
CAAGGACCAGGCCACCATGCATCAAAGCCAGATGAGCACCATCTCGCCGTCGACGGTGTCGCTGCC
CACCATTTCGCTGTCCACTGCGACTGGTGAGTCCAACACCGTCGAGCCGACCACGACGCTGTTCAAG
CCTCAGTCGGCCGAGGAGGTGGAGCGACGGCGACGCGTCGAGTCTCAATTTGGCCCGCTGCTTTCT
CCTGAGCACTTGTACACCAGCCAGGCCGCAGGCAAGCCCTTTGAGCGGCCGATTGAAGACGAGGCT
CCATACTACTATCTCTTGACCACCTACCTGAGCTACTTGGCGCTCATTCTGTTTGGCCACGTTCGTGAT
TTCTTTGGCAAGCGGTTCGGCGACCGCAAACGCTATCTGCCTCTCAAGATTCACAATGGATTCGCTCC
CCTCAATGATGATTTCGACAGCTTCTACAGCCGACGCCTCAAGACGCGTCTGGACGACTGCTTTGCC
CGCACCACTGTCGGCGTCCCCGGCCGCTTCATCACCCTCAAGGACCGCAAGTCGGACGACTACAACT
ACACCTACCGCTACACTGGCACCTACACTGAGACGCTAAACATGAGTTCCTACAACTATCTCGGTTTT
GCTCAGTCTGAGGGTCCCTGTGCCGATGCCGTCGAGGAATGCGTCAAGAAGTACGGAGCCACCTTT
GCCAGCCCCCGAGCTGACAGCGGCACTTCTGATTTGGCTCTTGAAGTTGAGCGAGAAATTGCGTCCT
TTGTTGGAAAGCCCGACGCCATGGTCTTCTCCATGGGCTACGTCACCAACTCCAGCACGTTCCCTGC
GCTTGTGTCCAAAGGCTGCCTGATCATCTCCGACGAGCTGAACCATGCCTCTATCCGAATCGGTGCT
CGTCTTTCTGGTGCTGTCATTCAGTCGTTCAAGCACAATGACATGGGCCACCTCGAGCGTGTTCTTCG
TGAAGCCATTTCCCAGGGCCAGCCAAGGACTCACCGCCCCTGGAAGAAGATTCTCTTGGTCGTCGAA
GGTCTCTACTCGATGGAAGGTACCATGGTCAACCTGCCCGGAATCCTGGAACTGAAGCGCAAGTAC
AAGTTTTACCTGTTTATCGACGAGGCTCACTCCATCGGTGCCCTGGGACCCCACGGCCGTGGCGTTT
GCGATTACTATGGCGTTGACACCTCGGAAGTCGACATCCTCATGGGCACTCTGACCAAGTCTTTCGG
CGCCAACGGAGGCTACGTTGCTGCGGAGAAGCACATCATTGACAAGCTCCGCAGCACCAACGCCTC
CACCATCTTTGGCGAGGCCCCTGCCCCCTCGGTTCTCATGCAGATTCTTGCCTCCCTCAAGCTGATTA
ACGGAGATATTTGCCCTGGCCAGGGCGAGGAGCGCCTGAAGCGCATTGCCTTCAACTCTCGCTACCT
TCGCCTCGGACTCAAGCGTCTTGGTATGATTGTGACTGGCCACGACGACTCACCCATCATCCCCGTCC
TGCTTTACAACCCCGGAAAGATGCCTGCCTTCAGTCGTCAGATGCTTGCCCGCAAAATCTCCGTCGTC
GTCGTCGGTTATCCCGCTACTCCGCTCATCAGCTCGCGTGCTCGCTTCTGCGTCTCTGCTGCCCACAA
CAAGGATGACCTCGACCGAATGCTCATTGCCTGTGACGAGGTTGCCGACCTTCTCCAGCTCAAGTAC
TCCACAGGAATTGCCGGTGGTTTGGACCCTCTGCCCGAGGGCGTCGCACCAGAAGACGAGGCTGAG

TGGAGGAAGAACACTGGCACTCCCATCAAGGCCCCCGATGGAAGGTTGAGGACGTCATTCGACGA
GGTGTCGTTGACTGCAAGCTCCCTCTGCGGTGA
>Trichoderma atroviride
ATGCCGCGACGGCTCGCAAACCCCTTCTCCTCTACCCAGTCTGCTATGTCAGCATCCGAGAAGACTG
GCGAAAGACGTCTCTCGTCCTCCAAGACCAACCGACTGAGCCAGCTCTTCTCCTCCAGCCCAAAGTCC
AAGGATCAGTCACCACTCGCCGCCATGCCGCAGAGCCAGATGAACACCATCTCGCCGTCGACGGTG
TCGCTGCCCACCATTTCGCTGTCGACGGCGACCGGCGAGCCCAACAGCATCGAGCCAACCAAGATG
CTGTTCAAGCCGCAATCGGCCGAGGAGCAGGAGCAGCGACGGCTCGCCGAGTCTCAATTTGGCCCT
CTGCTTGATCCGTCCCACCGCTATGTCAGCCAGTCCAATGGCGAGACGTTCAAGGAGCCGATTGAGG
ATATGCCTCCGTACTTCTATCTCCTGACCACCTACTTGAGCTATCTCTTCTTGATCATCATTGGCCACT
GTCGCGATTATTTTGGCAAGCGTTTTGGCGATAAAAAGCGCTACAACGCCCTCAAAGTGCAAAACGG
GCTCGCGCCTCTCACTGATGATTTCGACAGCTTCTACAGCCGCCGGCTGAAGGGTCGTTTGGATGAC
TGCTTTGCTCGACCTACGTACGGCGTTCCCGGCCGCTTCATTACCCTCAAGGAACGTACGGCAGACA
GGCTTAACCGCAACTACCACTACACTGGAAACCACGTCGAGACACTCAACGTGAGCTCCTACAACTA
CCTCGGCTTTGCTCAATCGCAGGGCCCCTGCGCCGATGCTGTCGATGAATGTGTCAAGAAATACGGT
GTTACCGCTGCAAGCCCCCGCGGCGATAGCGGCACTTCCGACCTAGCCCTCGAAGTTGAACGCGAA
GTTGCATCCTTTGTCGGAAAGCCAGAGGCCATGGTCTTCTCCATGGGCTATGTGACCAACTCCAGTA
CCTTCCCTGCTCTCGTGTCAAAGGGCTGCCTGATTGTCTCCGACGAACTGAACCATGCCTCCATCCGT
GTCGGTGCTCGCCTCAGTGGCGCCGTTATCCAATCTTTCAAGCACAACGACGTGGTCGCCCTGGAGC
GTGTTCTTCGTGAAGCCATCTCCCAGGGCCAGCCAAGGACTCACCGCCCCTGGAAGAAGATTCTCGT
CGTTGTCGAAGGTCTCTACTCTATGGAAGGTACTATGGTCAATCTGCCGGCAATTGTGGCCCTCAAG
CGCAAGTACAAGTTCTACCTGTACGTTGACGAGGCTCACTCTATTGGTGCCCTGGGACCCCGTGGCC
GTGGTGTCTGTGATTACTTTGGCGTTGACCCCTCCGAGGTTGACATTCTCATGGGCACCCTGACCAA
GTCCTTTGGCGCCAACGGAGGCTACATTGCGGCAGAGAAGCACATCATTGACAAGCTCCGTTCCACC
AACGTCTGCTCAATCTACGGCGAAGCCCCTTCTCCTTTTGTCCTCATGCAGATTCTTACCTCGATCAAG
CTGATCAACGGCGAGATTTGCCCTGGCCAGGGCGAGGAGCGTCTCCAGCGAATTGCCTTCAACTCTC
GCTATCTTCGCCTTGGACTCAAGCGTCTTGGACTCATCGTTGCTGGCTCTGATGATTCTCCCATTATCC
CCGTTCTGCTGTACAACCCCGGAAAGATGCCTGCCTTTAGCCGCGAAATGCTCAAGCGCAACATCTC
TGTTGTCATCGTTGGTTACCCGGCCACCCCGCTCATCAGCTCGCGTGCCCGCTTCTGCATCTCTGCCG
CTCATAACAAAGATGACCTTGACCGAATGATCAGAGCCTGTGATGAAGTTGGCGACATGCTCCAGAT
CAAGTTCTCATCAGGCATTGCTGGCGGCTTGGAGCCGCTGCCTGCTGGTGTAGCACCAGAAAATGA
GGCTGAGTGGAGGAGGGCTAACAATGTCGCCATCAGGCCTCCTCGATGGGATGTCGAAGAGGTCA
TTCGACGAGGTGCCGTTGATTGCAAGCTTCCTCTGCGATGA
>Trichoderma sp. T154
ATGCCGCGACGGCTTGCAAATCCCTTCTCCTCCAGCCAGTCTGCCATGTCAGCGTCCGAAAAGACTG
GCGAACGACGTCTCTCAACCTCAAAGACCAACCGACTGAGCCAGCTCTTCTCGTCCAGCCCAAAGTC
CAAGGACCAGGCCGCCATGCATCAAAGCCAGATGAGCACCATTTCGCCGTCGACGGTGTCGCTGCC
CACCATTTCGCTGTCCACTGCGACTGGTGAGTCCAACACCGTCGAGCCGACCACGACGCTGTTCAAG
CCTCAGTCGGCCGAGGAGGTGGAGCGACGGCGACGCGTCGAGTCTCAATTTGGCCCGCTGCTTTCT
CCCGAGCACTTGTACACCAGCCAGGCCGCGGGCAAGCCCTTTGAGCGGCCGATTGAAGACGAGCCT
CCGTACTACTATCTCTTGACCACCTACCTGAGCTACTTGGCTCTCATTCTGTTTGGCCACGTTCGCGAT
TTCTTTGGCAAGCGATTCGGCGACCGCAAGCGCTATCTGCCTCTCAAGATTCACAATGGATTCGCTCC
CCTCAATGATGATTTCGACAGCTTCTACACCCGACGCCTCAAGAGGCGTCTGGACGACTGCTTTGCG
CGCACCACTGTCGGCGTCCCTGGCCGCTTCATCACCCTCAAGGACCGCAAGTCGGACGACTACAACT
ACACCTACCGCTACACTGGCACCTACACTGAGACGCTAAACATGAGTTCCTACAACTATCTCGGTTTT
GCTCAGTCTGAGGGTCCCTGTGCCGATGCCGTCGAGGAATGCGTCAAGAAGTATGGAGCCACCTTT
GCCAGCCCCCGAGCTGACAGCGGCACTTCTGATTTGGCTCTCGAAGTCGAGCGGGAAATTGCGTCCT

TTGTTGGAAAGCCCGACGCCATGGTCTTCTCCATGGGCTACGTCACCAACTCCAGCACGTTCCCTGCA
CTTGTGTCCAAGGGTTGCCTGATCATCTCCGACGAACTGAACCATGCCTCTATCCGAATCGGTGCTCG
TCTTTCTGGTGCTGTCATTCAGTCGTTCAAGCACAACGACATGGGCGACCTCGAGCGTGTTCTTCGTG
AAGCCATTTCTCAGGGCCAGCCAAGGACTCACCGCCCCTGGAAGAAGATTCTCTTGGTCGTCGAAG
GTCTCTACTCGATGGAAGGTACCATGGTCAACCTGCCCGGAATCCTGGAACTGAAGCGCAAGTACA
AGTTTTACCTGTTCATCGACGAGGCTCACTCCATCGGTGCCCTGGGACCCCACGGCCGTGGCGTTTG
CGATTACTATGGCGTTGACACCTCGGAAGTCGATATCCTCATGGGCACTCTGACCAAGTCTTTCGGC
GCCAACGGAGGCTACGTTGCTGCGGAGAAGCACATCATTGACAAGCTCCGCAGCACCAACGCCTCC
ACCATCTTTGGCGAGGCCCCTGCCCCCTCGGTTCTTATGCAGATTCTTGCCTCCCTCAAGCTGATTAA
CGGAGAGATTTGCCCTGGCCAGGGTGAGGAGCGCCTGAAGCGCATTGCCTTCAACTCTCGCTACCTT
CGCCTCGGACTCAAGCGTCTCGGCATGATTGTGACTGGTCACGACGACTCACCCATCATCCCCGTCCT
GCTTTACAACCCCGGAAAGATGCCTGCCTTCAGTCGCCAGATGCTTGCCCGCAAAATCTCCGTTGTC
GTCGTCGGTTATCCTGCCACTCCGCTCATCAGCTCGCGTGCTCGCTTCTGCGTCTCTGCTGCCCACAA
CAAGGATGACCTCGACCGAATGCTCATTGCCTGTGACGAGGTTGCCGACCTTCTCCAGCTCAAGTAC
TCCACGGGAATTGCCGGTGGTTTGGACCCTCTGCCCGAGGGCGTCGCACCAGAAGACGAGGCTGAG
TGGAGGAAGAACACTGGCACTCCCATCAAGGCCCCCGATGGAAGGTTGAGGACGTCATTCGACGA
GGTGTCGTTGACTGCAAGCTCCCTCTGCGGTGA
>Trichoderma citrinoviride
ATGCCGCGGCGGCTCGCAAACCCCTTCTCCTCCAGCCAGTCTGCCATGTCAGCGTCCGAAAAGACTG
GCGAACGACGTCTCTCGTCCTCCAAGACCAACCGCCTGAGCCAGCTCTTCTCGTCCAGCCCAAAGTC
CAAGGATCAGGCCACCATGCATCAAAGCCAGACGAGCGCCATTTCGGCGTCCACGGTGTCGCTGCC
CACCATTTCGCTGTCCACCGCGACGGGCGAGGCCAACGCGGTCGAGCCCACGACGACGCTGTTCAA
GCCTCAGTCGGCCGAGGAGGTGGAACGGCGGCGACGCACCGAGTCTCAATTCGGTCCCCTGCTCTC
CCCCGAGCACCTGTACGTCAGCCAGGCCCAAGGCAAGCCCTTTACGCGCCCGATTGAGGACGAGCC
GGCCTACTACTATCTCTTGACCACGTACCTGAGCTACCTGGCCCTCATCCTGTTCGGACACGTCCGCG
ACTTCTTCGGCAAGCGGTTCGGTGACCGCAAGCGCTACGCGCCCCTCAAGGTGCACAACGGCTTCGC
TCCTCTCAATGATGACTTCGACAGCTTCTACAGCAGGCGGCTCAAGCTCCGCCTGGACGATTGCTTTG
CCCGCACCACCGTCGGCGTCCCCGGGCGCTACATCACCCTCAAGGACCGCAAGTCGGACGACTACA
ACTACACCTACAAATACACCGGTACCCACACTGAGACCCTAAACATGAGTTCCTACAACTATCTCGGT
TTTGCTCAGTCGGACGGTCCCTGCGCCGATGCCGTCGAGGAATGCGTCAGGAAGTACGGAGCCACC
TTTGCCAGCCCTCGAGCTGACAGCGGCACCTCGGATCTGGCCCTCGAGGTCGAGCGCGAAATTGCCT
CGTTTGTTGGAAAGCCCGACGCCATGGTCTTCTCCATGGGCTACGTCACCAATGCCAGCACGTTTCCC
GCGCTTGTGTCCAAGGGCTGCCTGATCATCTCCGACGAGCTGAACCACGCCTCTATCCGAATTGGTG
CCCGTGTCTCCGGTGCCGTCATCCAGTCTTTCAAGCACAACGACGTCGAGGCCCTGGAGCGCGTCCT
CCGCGAGGCCATCTCGCAGGGCCAGCCAAGGACTCACCGCCCCTGGAAGAAGATCCTCGTCGTTGT
CGAAGGCCTCTACTCCATGGAAGGCAGCATGGTCAACCTGCCCGGCATCCTGGCTCTGAAGCGCAA
GTACAAGTTCTACCTGTTCATTGACGAGGCTCACTCCATCGGCGCCCTGGGACCCCGCGGCCGCGGC
ATCTGTGACTACTATGGCGTTGACCCTTCGGAAGTCGACATCCTCATGGGCACCTTGACCAAGTCTTT
TGGCGCCAACGGAGGCTACATCGCCGCTGAGAAGCACATCATCGACAAGCTCCGTGTGACCAACGC
CTCCACCATCTTTGGCGAGGCGCCCGCCCCTTCGGTTCTCATGCAGATCCTCACGTCTCTGAAGCTCA
TCAACGGCGAGATCAGCCCCGGCCAGGGTGAAGAGCGCCTGCAGCGCATTGCCTTCAACTCCCGCT
ATCTGCGTCTCGGACTCAAGCGTCTCGGCATGATTGTCACTGGCGACGACGATTCACCCATCATTCCC
GTCCTGCTTTACCACCCCGGCAAGATGCCCGCTTTCAGCCGTCTGATGCTTGCCCGCAAGATCTCCGT
TGTCGTCGTCGGTTACCCGGCCACTCCGCTCATCAGCTCGCGTGCCCGCTTCTGCGTCTCTGCCGCTC
ACAACAAGGATGACCTCGACCGGATGCTCATTGCCTGCAACGAGGTTGCCGACCTGCTCCAGCTCAA
GTACTCGACGGGTATTGCCGGAGGCTTGGAGCCGCTGCCCGAGGGCGTGGCGCCTGAAGACGAGG

CCGAGTGGAGGAAGGCCAATGGCATCCCCATCAAGCCTCCCAGGTGGAAGATTGGAGATGTCATTG
CGCGAGGCGTTGCTGACTGCAAGCTTCCTCTGCGATGA
>Trichoderma gamsii
ATGCCGCGACGGCTCGCAAACCCCTTCTCCTCTACCCAGTCTGCTATGTCAGCATCCGAGAAGACTG
GCGAAAGACGTCTCTCGTCCTCCAAGACCAACCGACTGAGCCAGCTCTTCTCCTCCAGCCCAAAGTCC
AAGGACCAGTCACCACTCGCCGCCATGCCGCAGAGCCAGATGAACACCATCTCGCCGTCGACGGTG
TCGCTGCCCACCATTTCGCTGTCGACGGCGACCGGCGAGCCCAACAGCATCGAGCCAACCAAGATG
CTGTTCAAGCCGCAATCGGCCGAGGAGCAAGAGCAGCGACGACTCGCCGAGTCTCAATTTGGCCCC
CTGCTTGATCCGGCCCACCGCTATGTCAGCCAGTCCAATGGCGAGGAGTTCAAGGAGCCGATTGAG
GATATGCCTCCGTACTTCTATCTCCTGACCACCTACCTGAGCTATCTCTTCTTGATCATGATGGGCCAT
TGTCGCGATTATTTTGGCAAGCGTTTTGGAGATAAAAAGCGCTACGACCCCCTCAAAGTGCGAAACG
GGTTCGCCCCTCTCACTGATGATTTCGACAGCTTCTACACTCGTCGGCTGAAGGGTCGTTTGGATGAC
TGCTTTGCTCGCCCTACTTTCGGCGTTCCCGGTCGTTACATTACTCTCAAGGAACGTACGGCAGACAG
GCTTAACCGCAACTACCACTACACTGGAAACCACGTCGAGACACTCAATGTGAGCTCCTACAACTAC
CTCGGCTTTGCTCAATCGCAGGGCCCCTGTGCCGATGCTGTCGATGAATGTGTCAAGAAGTACGGTG
TTACCGCTGCAAGCCCGCGTGGCGATAGCGGCACTTCCGACCTGGCCCTCGAAGTTGAACGCGAAG
TTGCAACCTTTGTCGGAAAGCCAGAGGCCATGGTCTTCTCTATGGGTTACGTTACCAACTCCAGTACC
TTCCCCGCTCTCGTGTCAAAGGGCTGCCTGATTGTCTCTGACGAACTGAACCATGCCTCCATTCGTGT
CGGTGCTCGTCTCAGTGGCGCTGTTATCCAGTCTTTCAAGCACAACGACGTGGCCGCCCTGGAGCGC
GTTCTTCGTGAAGCCATCTCCCAGGGCCAGCCAAGGACTCACCGCCCCTGGAAGAAGATTCTCGTCG
TTGTTGAAGGTCTCTATTCTATGGAAGGCACTATGGTTAATCTGCCGGCAATTGTGGCTCTCAAGCG
CAAGTACAAGTTCTACCTGTACGTTGACGAGGCTCACTCCATTGGTGCCCTGGGACCCCGTGGCCGT
GGTGTCTGTGATTACTTTGGTATTGATCCCTCCGAGGTTGACATCCTCATGGGCACCCTGACCAAGTC
CTTTGGCGCCAACGGAGGCTACATTGCGGCAGAGAAGCACATCATTGACAAGCTCCGTGCCACCAA
CGTCTGCTCAATCTATGGCGAAGCCCCTTCTCCTTTTGTCCTCATGCAGATTCTTACCTCGATCAAGCT
GATTAACGGCGATATTTCCCCTGGCCAGGGCGAGGAGCGTCTCCAGCGAATTGCCTTCAACTCTCGC
TATCTTCGCCTTGGACTTAAGCGTCTTGGCCTCATCGTTGCTGGCTCTGATGATTCTCCCATTATCCCC
GTTCTGCTGTACAACCCTGGAAAGATGCCTGCCTTTAGCCGCGAAATGCTCAAGCGCAACATCTCTG
TTGTCGTCGTTGGTTACCCGGCTACCCCGCTCATCAGCTCGCGTGCCCGTTTCTGCATCTCTGCCGCT
CACAACAAGGATGACCTTGACCGCATGATCAGAGCTTGCGACGAGGTCGGCGAATTGCTTCAGCTC
AAGTTCTCTTCAGGCATTGCTGGCGGCTTGGAGCCGCTGCCTGCTGGTGTAGCACCAGAAAACGAG
GCTGAGTGGAGGAGGGCCAACAATGTCGCCATCAAGCCTCCTCGATGGGATGTTGAAGAGGTCATT
CGACGAGGTGCCATCGATTGCAAGCTTCCTCTGCGATAA
>Trichoderma guizhouense
ATGCCGCGACGGCTTGCAAATCCCTTCTCCTCCAGCCAGTCTGCCATGTCAGCGTCCGAAAAGACTG
GCGAACGACGTCTCTCAAGCTCAAAGACCAACCGACTGAGCCAGCTCTTCTCGTCCAGCCCAAAGTC
CAAGGACCAGGCCACCATGCATCAAAGCCAGATGAGCACCATCTCGCCGTCGACGGTGTCACTGCC
CACCATTTCGCTGTCCACTGCGACTGGTGAGTCCAACACCGTCGAGCCGACCACGACGCTGTTCAAG
CCTCAGTCGGCCGAGGAGGTGGAGCGACGGCAACGCGTCGAGGCTCAATTTGGCCCGCTACTTTCT
CCTGAGCACTTGTACACCAGCCAGGCCGCAGGCAAGCCCTTTGAGCGGCCGATTGAAGACGAGGCT
CCATACTACTATCTCTTGACCACCTACCTGAGCTACTTGGCGCTCATTCTGTTTGGCCACGTTCGTGAT
TTCTTTGGCAAGCGGTTCGGCGACCGCAAACGCTATCAGCCTCTCAAGATTCACAATGGATTCGCTC
CCCTCAATGATGATTTCGACAGCTTCTACACCCGACGCCTCAAGACGCGTCTGGACGACTGCTTTGCC
CGCACCACGGTCGGCGTCCCCGGCCGCTTCATCACCCTCAAGGACCGCAAGTCGGACGACTACAACT
ACACCTACCGCTACACCGGCACCTACACTGAGACGCTAAACATGAGTTCCTACAACTATCTCGGTTTT
GCTCAGTCTGAGGGTCCCTGTGCCGATGCCGTCGAGGAATGCGTCAAGAAGTACGGAGCCACCTTT
GCCAGCCCCCGAGCTGACAGCGGCACTTCTGATTTGGCTCTCGAAGTCGAGCGGGAAATTGCGTCCT

TTGTTGGAAAGCCCGACGCCATGGTCTTCTCCATGGGCTACGTCACCAACTCCAGCACGTTCCCTGCA
CTTGTGTCCAAGGGTTGCCTGATCATCTCCGACGAACTGAACCATGCCTCTATCCGAATCGGTGCTCG
TCTTTCTGGTGCTGTCATTCAGTCGTTCAAGCACAACGACATGGGCCACCTCGAGCGTGTTCTTCGTG
AAGCCATTTCCCAGGGCCAGCCAAGGACTCACCGCCCCTGGAAGAAGATCCTCTTGGTCGTCGAAG
GTCTCTACTCGATGGAAGGTACCATGGTCAACCTGCCCGGAATCCTGGAACTGAAGCGCAAGTACA
AGTTTTACCTGTTTATCGACGAGGCTCACTCCATCGGTGCCCTGGGACCCCACGGCCGTGGCGTTTG
CGATTACTATGGCGTTGACACCTCGGAAGTCGACATCCTCATGGGCACTCTGACCAAGTCTTTTGGC
GCCAACGGAGGCTACATTGCTGCGGAGAAGCACATCATTGACAAGCTCCGCAGCACCAACGCCTCC
ACCATCTTTGGCGAAGCCCCTGCCCCCTCGGTTCTCATGCAGATTCTTGCCTCCCTCAAGCTGATTAA
CGGAGATATTTGCCCCGGTCAGGGCGAGGAGCGTCTGAAGCGCATTGCCTTTAACTCTCGCTACCTC
CGTCTCGGACTCAAGCGTCTTGGTATGATTGTGACTGGTCACGACGACTCACCCATCATCCCCGTCCT
GCTTTACAACCCCGGGAAGATGCCTGCTTTCAGTCGCCAGATGCTTGCCCGCAAAATCTCTGTCGTC
GTCGTGGGTTATCCCGCCACTCCGCTTATCAGCTCACGTGCTCGCTTCTGCGTCTCTGCTGCCCACAA
CAAGGATGACCTCGACCGAATGCTCATTGCCTGTGACGAGGTTGCCGACCTTCTCCAGCTCAAGTAC
TCCACAGGAATTGCCGGTGGTTTGGACCCTCTGCCCGAGGGCGTCGCACCAGAAGACGAGGCTGAG
TGGAGGAAGAACACTGGCACTCCCATCAAGGCCCCCGATGGAAGGTTGAGGACGTCATCCGACGA
GGTGTCGTTGACTGCAAGCTCCCTCTGCGGTGA
>Trichoderma hamatum
ATGCCGCGACGGCTCCAAAACCCCTTCTCCTCCAACCAGTCTGCTATGTCAGCATCCGAGAAGACTG
GCGAAAGACGTCTCTCGTCCTCCAAGACCAACCGACTGAGCCAGCTGTTCTCATCCAGCCCAAAGTC
CAAGGACCAGTCACCCCTCGCCGCCATGCATCAAGGCAACACCATCTCGCCATCGACGGTGTCGCTG
CCTACCATCTCCCTGTCTACGGCGACTGGCGAGTCCAACAATGTCGAGCCAGCCGCCAAGATGCTGT
TCAAGCCTCAATCGGCTGAGGAGCAAGAGCAACGACGACTCGCCGAGTCTCAGTTTGGCCCTCTGCT
TGATTCATCCCACCGCTACGTGAGCAAGCAGAATGGCGAAGAGTTCAAGGAGCCGGTTGAGGATAT
GCCTCCCTACTGGTTCCTCTTGACCACCTACATAAGCTATCTTGTGTTAATAATTTTTGGCCACTGTCG
CGATTACTTTGGCAAGCGCTTTGGCGATAAAAAGCGCTACAACGCCCTCAAAGTGCAAAACGGACTC
GCACCTCTCACTGATGACTTCGACAGCTTCTACACCCGCCGTTTGAAAGGCCGTCTGGATGATTGCTT
TGCTCGACCTACTGTCGGCGTTCCCGGCCGCTTCATTACTCTCAAGGAACGCACGGCTGACAGACTT
AACCGCAACTACCACTACACTGGCAACTACGTTGAGACACTCAACGTGAGCTCCTACAACTACCTCG
GCTTTGCTCAATCGCAGGGCCCCTGTGCCGATGCTGTCGATGAATGTGTCAAGAAGTACGGTGTTAC
CGCCGCAAGCCCCCGCGGTGACAGCGGCACTTCCGACCTGGCCCTTGAAGTTGAGCGCGAAGTTGC
AGCCTTCGTTGGAAAGCCCGAGGCCATGGTCTTCTCTATGGGTTATGTTACCAACTCCAGTACCTTCC
CTGCTCTCGCGTCCAAAGGCTGCCTGATTGTCTCTGACGAACTGAACCATGCCTCCATTCGTGTCGGT
GCTCGTCTGAGTGGCGCCGTTATCCAGTCTTTCAAGCACAACGACATGACCTCCCTGGAAAAGGTTC
TTCGTGAAGCCATCTCCCAGGGTCAGCCAAGGACTCACCGCCCCTGGAAGAAGATTCTTGTCGTTGT
CGAGGGTCTCTACTCTATGGAAGGTACTATGGTTAATCTGCCGGCAATTGTGGCTCTCAAGCACAAG
TACAAGTTCTACCTGTATGTTGATGAAGCTCACTCTATCGGTGCCCTGGGACCCCGTGGCCGTGGTG
TCTGCGATTACTTTGGCGTTGATCCCTCCGAGGTTGACATTCTTATGGGTACCCTGACCAAGTCGTTT
GGCGCCAATGGAGGCTACATTGCGGCTGAGAAGCACATCATTGACAAGCTCCGTGCCACAAACGTC
TGCTCAATATACGGCGAATCTCCTTCCCCCTTTGTCCTTATGCAGATTCTTACCTCGATCAAGCTTATT
AACGGCGACATTTGCCCCGGTCAGGGCGAGGAGCGTCTTCAGCGAATCGCCTTCAACTCTCGCTACC
TTCGTCTTGGACTCAAGCGTCTTGGCTTAATCGTTGCTGGTTCTGATGACTCTCCCATTATCCCCGTCC
TGCTCTACAACCCCGGAAAGATGCCTGCCTTCAGCCGCGAAATGCTCAAGCGTAACATCTCTGTTGTT
ATCGTTGGTTACCCGGCTACCCCGCTCATCAGCTCGCGTGCCCGCTTCTGCATTTCTGCCGCTCACAA
CAAAGATGACCTTGACCGAATGATTAGGGCTTGTGACGAGGTTGGCGAAATGCTTCAGCTCAAGTT
CTCATCAGGCATTGCTGGCGGCTTGGAGCCGCTGCCCGCTGGTGTGGAACCAGAGAATGAGGCTGA

GTGGAGAAGGGTCAACAATGTCCCCATCAAGCCTCCTCGATGGGATGTTGAGGAGGTCATTCGACG
AGGCGCCGCCGATTGCAAGCTTCCTCTGCGATAA
>Trichoderma harzianum
ATGCCGCGACGGCTTGCAAATCCCTTCTCCAGCCAGTCTGCCATGTCAGCGTCCGAAAAGACTGGCG
AACGACGTCTCTCAACCTCAAAGACCAACCGACTGAGCCAGCTCTTCTCGTCCAGCCCAAAGTCCAA
GGACCAGGCCGTCATGCATCAAAGCCAGATGAGCACCATTTCGCCGTCGACGGTGTCGCTGCCCAC
CATTTCGCTGTCCACTGCGACTGGTGAGTCCAACACCGTCGAGCCGACCACGACGCTGTTCAAGCCT
CAGTCGGCCGAGGAGGTGGAGCGACGGCGACGCGTCGAGTCTCAATTTGGCCCGCTGCTTTCTCCC
GAGCACTTGTACACCAGCCAGGCCGCGGGCAAGCCCTTTGAGCGGCCGATTGAAGACGAGCCTCCG
TACTACTATCTCTTGACCACCTACCTGAGCTACTTGGCTCTCATTCTGTTTGGCCACGTTCGTGATTTC
TTTGGCAAGCGATTCGGCGACCGCAAGCGCTATCTGCCTCTCAAGATTCACAATGGATTCGCTCCCCT
CAATGATGATTTCGACAGCTTCTACACCCGACGCCTCAAGACGCGTCTGGACGACTGCTTTGCCCGC
ACCACTGTCGGCGTCCCTGGCCGCTTCATCACCCTCAAGGACCGCAAGTCGGACGACTACAACTACA
CCTACCGCTACACTGGCACCTACACTGAGACGCTAAACATGAGTTCCTACAACTATCTCGGTTTTGCT
CAGTCTGAGGGTCCCTGTGCCGATGCCGTCGAGGAATGCGTCAAGAAGTACGGAGCCACCTTTGCC
AGCCCCCGAGCTGACAGCGGCACTTCTGATTTGGCTCTCGAAGTCGAGCGGGAAATTGCGTCCTTTG
TTGGAAAGCCCGACGCCATGGTCTTCTCCATGGGCTACGTCACCAACTCCAGCACGTTCCCTGCACTT
GTGTCCAAGGGTTGCCTGATCATCTCCGACGAACTGAACCATGCCTCTATCCGAATCGGTGCTCGTCT
TTCTGGTGCTGTCATTCAGTCGTTCAAGCACAACGACATGGGCGACCTCGAGCGTGTTCTTCGTGAA
GCCATTTCTCAGGGCCAGCCAAGGACTCACCGCCCCTGGAAGAAGATTCTCTTGGTCGTCGAAGGTC
TCTACTCGATGGAAGGTACCATGGTCAACCTGCCCGGAATCCTGGAACTGAAGCGCAAGTACAAGTT
TTACCTGTTCATCGACGAGGCTCACTCCATCGGTGCCCTGGGACCCCACGGCCGTGGCGTTTGCGAT
TACTATGGCGTTGACACCTCGGAAGTCGATATCCTCATGGGCACTCTGACCAAGTCTTTCGGCGCCA
ACGGAGGCTACGTTGCTGCGGAGAAGCACATCATTGACAAGCTCCGCAGCACCAACGCCTCCACCA
TCTTTGGCGAGGCCCCTGCCCCCTCGGTTCTTATGCAGATTCTTGCCTCCCTCAAGCTGATTAACGGA
GAGATTTGCCCTGGCCAGGGTGAGGAGCGCCTGAAGCGCATTGCCTTCAACTCTCGCTACCTTCGCC
TCGGACTCAAGCGTCTCGGCATGATTGTGACTGGTCACGACGACTCACCCATCATCCCCGTCCTGCTT
TACAACCCCGGAAAGATGCCTGCCTTCAGTCGCCAGATGCTTGCCCGCAAAATCTCCGTTGTCGTCG
TCGGTTATCCTGCCACTCCGCTCATCAGCTCGCGTGCTCGCTTCTGCGTCTCTGCTGCCCACAACAAG
GATGACCTCGACCGAATGCTCATTGCCTGTGACGAGGTTGCCGACCTTCTCCAGCTCAAGTACTCCA
CGGGAATTGCCGGTGGTTTGGACCCTCTGCCCGAGGGCGTCGCACCAGAAGACGAGGCTGAGTGG
AGGAAGAACACTGGCACTCCCATCAAGGCCCCCGATGGAAGGTTGAGGACGTCATTCGACGAGGT
GTCGTTGACTGCAAGCTCCCTCTGCGGTGA
>Trichoderma parareesei
ATGCCGCGGCGGCTCGCAAACCCCTTCTCCTCCAGCCAGTCTGCCATGTCAGCGTCCGAAAAGACTG
GCGAACGACGTCTCTCGGCCTCCAAGACCAACCGGTTGAGCCAGCTCTTCTCGTCCAGCCCAAAGTC
CAAGGACCAGGCCGCCATGCATCAAAGCCAGATGAGCACCGTCTCGCCGTCCACGGTGTCGCTGCC
CACCATTTCGCTGTCCACCGCGACGGGCGAGCCCAACACGGCCGAGCCCACCACGACGTTGTTCAAG
CCTCAGTCGGCCGAGGAGGTGGAGCGACGACGACGCGCCGAGTCTCAATTCGCTCCGCTGCTCTCC
CCGGAGCACCTGTACGTCAGCCAGGCCCATGGCAAGCCCTTTACGCGGCCGATTGAGGACGAGCCG
GCCTACTACTATCTCTTAACCACGTATCTGAGCTACCTGGCTCTCATCCTGTTTGGACACGTCCGCGAT
TTCTTTGGCAAGCGGTTCGGCGACCGCAAGCGCTACGCGCCCCTCAAGGTGCACAACGGCTTCGCTC
CTCTCAACGATGACTTCGACAGCTTCTACAGCAGGCGGCTCAAGCTCCGCCTGGACGATTGCTTTGC
CCGCACCACTGTCGGCGTCCCCGGTCGCTACATCACCCTCAAGGACCGCAAGTCGGACGACTACAAC
TACACCTACCGTTACACCGGCACCCACACTGAGACCCTAAACATGAGTTCCTACAACTATCTCGGTTT
TGCTCAGTCGGACGGTCCCTGCGCCGATGCCGTCGAGGAATGCGTCAGGAAGTACGGAGCCACCTT
TGCCAGCCCCCGAGCTGACAGCGGCACTTCGGATCTGGCCCTCGAGGTCGAGCGCGAAATCGCCTC

GTTTGTTGGAAAGCCCGACGCCATGGTCTTCTCCATGGGCTACGTCACCAATGCCAGCACGTTCCCT
GCGCTCGTGTCCAAGGGCTGCCTGATCATCTCGGACGAGCTCAACCACGCCTCTATCCGAATCGGTG
CCCGTGTTTCCGGTGCCGTTATCCAGTCTTTCAAGCACAACGACGTCGAGGCCCTGGAGCGCGTCCT
CCGCGAGGCCATCTCCCAGGGCCAGCCAAGGACTCACCGCCCCTGGAAGAAGATCCTCGTCGTTGT
CGAAGGCCTCTACTCCATGGAAGGCAGCATGGTCAACCTGCCCGGCATCCTGGCTCTGAAGCGCAA
GTACAAGTTCTACCTGTTCATCGACGAGGCTCACTCCATTGGCGCCCTGGGACCCCGCGGCCGCGGC
ATCTGTGATTACTACGGCGTCGACCCTTCGGAGATTGACATCCTCATGGGCACCTTGACCAAGTCCTT
CGGCGCCAACGGAGGCTACATCGCCGCTGAAAAGCACATCATCGACAAGCTCCGTGTGACCAATGC
CTCCACCATCTTTGGCGAAGCGCCTGCCCCTTCGGTTCTCATGCAGATCCTCACGTCCCTGAAGCTCA
TCAACGGCGAGATCAGCCCCGGCCAGGGTGAAGAGCGCCTGCAGCGCATTGCCTTCAACTCTCGCT
ATCTCCGCCTCGGACTCAAGCGTCTCGGCATGATTGTCACTGGCGACGACGATTCACCCATCATCCCT
GTCCTGCTTTACCACCCCGGCAAGATGCCCGCCTTCAGCCGTCTGATGCTCGCCCGCAAGATCTCCGT
CGTCGTCGTCGGGTACCCGGCCACTCCGCTCATCAGCTCGCGTGCTCGCTTCTGCGTCTCTGCCGCTC
ACAACAAGGATGACCTCGACCGGTTGCTCATCGCCTGCGACGAGATTGCCGACCTGCTGCAGCTCA
GGTACTCGACAGGTATTGCCGGAGGCTTGGAGCCGCTTCCCGAGGGAGTGGCGCCCGAGGACGAG
GCCGAGTGGAGGAAGGCCAATGGCATTCCCATCAAGCCCCCCAGGTGGAAGGTTGAGGATGTCATT
GCGCGAGGCGTCGCTGACTGCAAGCTTCCCCTGCGATGA
>Trichoderma reesei
ATGCCGCGGCGGCTCGCAAACCCCTTCTCCTCCAGCCAGTCTGCCATGTCAGCGTCCGAAAAGACTG
GCGAACGACGTCTCTCGGCCTCCAAGACCAACCGGTTGAGCCAGCTCTTCTCGTCCAGCCCAAAGTC
CAAGGACCAGGCCGCCATGCATCAAAGCCAGATGAGCACCGTCTCGCCGTCCACGGTGTCGCTGCC
CACCATTTCGCTGTCCACCGCGACGGGCGAGCCCAACACGGCCGAGCCCACCACGACGCTGTTCAA
GCCTCAGTCGGCCGAGGAGGTGGAGCGACGACGACGCGCCGAGTCTCAATTCGCTCCGCTGCTCTC
CCCGGAGCACCTGTACGTCAGCCAGGCCCATGGCAAGCCCTTTACGCGGCCGATTGAGGACGAGCC
GGCCTACTACTATCTCTTGACCACGTACCTGAGCTACCTGGCTCTCATCCTGTTTGGACACGTCCGCG
ATTTCTTTGGCAAGCGGTTTGGCGACCGCAAGCGCTACGCGCCCCTCAAGGTGCACAACGGCTTCGC
TCCTCTCAACGATGACTTCGACAGCTTCTACAGCAGGCGGCTCAAGCTCCGCCTGGACGATTGCTTT
GCCCGCACCACTGTCGGCGTCCCCGGCCGCTACATCACCCTCAAGGACCGCAAGTCGGACGACTACA
ACTACACCTACCGTTACACCGGCACCCACACTGAGACCCTAAACATGAGTTCCTACAACTATCTCGGT
TTTGCTCAGTCGGACGGTCCCTGCGCCGATGCCGTCGAGGAATGCGTCAGGAAGTACGGAGCCACC
TTTGCCAGCCCCCGGGCTGACAGCGGCACTTCGGATCTGGCCCTCGAGGTCGAGCGCGAAATCGCC
TCGTTTGTTGGAAAGCCCGACGCCATGGTCTTCTCCATGGGCTACGTCACCAATGCCAGCACGTTCCC
TGCGCTTGTGTCCAAGGGCTGCCTGATCATCTCCGACGAGCTCAACCACGCCTCCATTCGAATCGGT
GCCCGTGTTTCCGGCGCCGTCATCCAGTCTTTCAAGCACAACGACGTCGAGGCCCTGGAGCGCGTCC
TCCGCGAGGCCATCTCCCAGGGCCAGCCAAGGACTCACCGCCCCTGGAAGAAGATCCTCGTCGTTGT
CGAAGGCCTCTACTCCATGGAAGGCAGCATGGTCAACCTGCCCGGCATCCTGGCTCTGAAGCGCAA
GTACAAGTTCTACCTGTTCATCGACGAGGCTCACTCCATCGGCGCCCTGGGACCCCGCGGCCGCGGC
ATCTGTGATTACTACGGCGTCGACCCTTCGGAGATTGACATCCTCATGGGCACCTTGACCAAGTCCTT
CGGCGCCAACGGAGGCTACATCGCCGCCGAGAAGCACATCATCGACAAGCTCCGTGTGACCAACGC
CTCCACCATCTTCGGCGAGGCGCCTGCCCCTTCGGTTCTCATGCAGATCCTGACGTCCCTGAAGCTCA
TCAACGGCGAGATCAGCCCCGGCCAGGGTGAAGAGCGCCTGCAGCGCATCGCCTTCAACTCTCGCT
ATCTCCGCCTCGGACTCAAGCGTCTCGGCATGATTGTCACTGGCGACGACGATTCACCCATCATCCCT
GTCCTGCTTTACCACCCCGGCAAGATGCCCGCCTTCAGCCGCCTGATGCTCGCCCGCAAGATCTCCGT
CGTCGTCGTCGGTTACCCGGCCACTCCGCTCATCAGCTCGCGTGCTCGCTTCTGCGTCTCTGCCGCCC
ACAACAAGGATGACCTCGACCGGTTGCTCATCGCCTGCGACGAGATTGCCGACCTGCTGCAGCTCA
GGTACTCGACGGGTATTGCCGGAGGCTTGGAGCCGCTTCCCGAGGGCGTGGCGCCCGAGGACGAG

GCCGAGTGGAGGAAGGCCAATGGCATTCCCATCAAGCCCCCCAGGTGGAAGGTTGAGGATGTCATT
GCGCGAGGCGTCGCTGACTGCAAGCTTCCCCTGCGATGA
>Trichoderma virens
ATGCCGCGACGGCTTGCAAATCCCTTCTCCTCCAGCCAGTCTGCCATGTCAGCGTCCGAAAAGACTG
GCGAACGACGTCTCTCAACCTCCAAGACCAATCGACTGAGCCAGCTCTTCTCGTCCAGCCCAAAGTC
CAAGGACCAGGCCGCCATGCATCAAAGCCAGATGAGCACCATCTCGCCGTCCACGGTGTCGCTGCC
CACCATTTCGCTGTCCACTGCGACTGGTGAGTCCAACGCCGTCGAGCCGACCACGACGCTGTTCAAG
CCTCAGTCGGCCGAGGAGGTCGAGCGACGGCGACGCGTCGAGGCTCAATTTGCCCCGCTGCTTTCT
CCTGAGCACCTGTACACCAGCCAGGCTCATGGCAAGCCCTTTACGCGGCCGATTGAGGACGAGCCT
CCGTACTACTATCTCTTGACCACCTACCTGAGCTACTTGGCACTCATCCTGTTTGGCCACGTTCGTGAT
TTCTTTGGCAAGCGATTCGGCGACCGCAAGCGTTACCTGCCTCTCAAGGTGCACGGTGGATTCGCGC
CTCTCAATGATGATTTCGACAGCTTCTACAGCAGACGCCTGAAGCTCCGTCTGGACGACTGCTTTGCC
CGCACCACCGTCGGCGTTCCCGGCCGCTTCATCACCCTCAAGGACCGCAAGTCGGATGACTACAACT
ACACCTACCGCTACACCGGTACCCACACTGAGACGCTAAACATGAGTTCCTACAACTATCTCGGTTTT
GCTCAGTCTGAGGGTCCCTGCGCCGATGCCGTTGAGGAATGCGTCAAAAAGTACGGAGCCACCTTT
GCCAGTCCCCGATCTGACAGCGGCACTTCCGATTTGGCCCTCGAGGTCGAGCGGGAGATTGCGTCCT
TTGTTGGAAAGCCCGACGCCATGGTCTTTTCGATGGGCTACGTCACCAATGCCAGCACGTTCCCTGC
GCTTGTGTCCAAGGGCTGCTTGATCATCTCCGACGAGCTGAACCATGCCTCTATCCGAATCGGCGCT
CGCCTTTCTGGTGCCGTCATCCAGTCCTTCAAGCACAACGACATGGACGATCTGGAGCGTGTCCTCC
GTGAGGCCATTTCCCAGGGCCAGCCAAGGACTCACCGCCCCTGGAAGAAGATCCTCGTGGTCGTGG
AAGGTCTTTACTCTATGGAAGGCACCATGGTCAACCTGCCCGGAATTCTGGAACTGAAGGGCAAATA
CAAGTTTTACCTGTTCATTGATGAGGCTCACTCCATCGGTGCCCTGGGGCCCCGCGGCCGTGGCGTC
TGTGATTACTATGGCGTTGACACTGCTGAGGTCGACATTCTCATGGGTACTCTGACCAAGTCTTTCGG
CGCCAACGGAGGCTACATTGCTGCGGAGAAGCACATCATTGACAAGCTCCGTGCCACCAACGCCTCC
ACCATCTTTGGCGAGGCCCCTGCCCCTTCGGTTCTCATGCAGATTCTTGCCTCTCTCAAGCTGATTAAC
GGCGACATCTGCCCTGGCCAGGGTGAGGAGCGCCTGAGGCGCATTGCCTTCAACTCTCGCTATCTTC
GTCTTGGACTCAAGCGTCTTGGTATGATTGTGACTGGTCACGACGACTCACCCATCATCCCCGTCCTG
CTTTACAACCCCGGAAAGATGCCTGCTTTCAGTCGCCAAATGCTTGCCCGTAAGGTCTCCGTCGTCGT
CGTCGGTTATCCCGCCACCCCGCTCATCAGCTCGCGTGCTCGCTTCTGCGTCTCTGCCGCTCACAACA
AGGATGACCTCGACCGAATGCTCATTGCCTGCGATGAAGTTGCCGACCTGCTCCAGCTCAAGTACTC
TACCGGAATTGCTGGTGGCTTGGAGCCTCTGCCCGACGGCGTCGCACCAGAAGACGAGGCTGAGTG
GAGGAAGAACACCGGCACCCCCATCAAGGCTCCCCGATGGAAGATTGAGGACGTCATTCGACGAG
GCGCCGTTGACTGCAAGCTCCCTCTGCGATGA

Appendix S5. *rpb2* cds retrieved from the genomes of the 13 species analyzed in the present work

>Trichoderma arundinaceum
ATGGCTGACTACGAGGACGACTACGACTACGAGAACTATGGAGACGAGGATGAAGGCATCACCCCC
GAGGATTGCTGGACTGTGATCTCCTCATTCTTTGAGACAAAGGGTCTCGTATCACAACAGACCGACT
CCTTTGACGAGTTCACTCAGACGACGATTCAGGATCTTGTAAACGAATACTCCACCATCACACTCGAT
CAGCCAAATCCTCCTTCGCCACCCGGCCGAACGATAGCCCTCCGTCGATATGAGATCAAGTTTGGAA
GCGTCATGGTGTCACGCCCCACTATCAGTGAGACGGACGGAACTGTGACTTCTCTGCTTCCCTATGA
GTGTCGAGACCGCAACTTGACGTACGCCAGTCCGCTGTACATCAAGATCACCAAAAAGGTATCCGCT
GCCGTCGAGAGGGAGGTGCCCCTCCACGAAATGGACGATGCTCAGCAGGAGGAATATGCAAGGAC
CGGCGACCATCCTACAAAGCTTGAGTGGGAGGAGGAGGAGAATGGCGAAGACGACAATATCGGCA
AGTCTGATGACTGGAAGGACATGGTCTTTGTTGGCAAACTGCCCATCATGGTCAAATCCAAGATTTG
CCATCTGAGCCGTGAACAGGATGATAGTCTGTTCCTTGTCAACGAGTGTCCCTATGATCAAGGTGGC
TATTTCGTTATCAACGGTAGTGAGAAGGTTCTCATCGCTCAAGAGCGATCCGCCGCCAACATTGTCC
AAGTATTCAAGAAGGCCCAGCCCAGTGCCTACACATACACCGCTGAAATTCGAAGTGCGTTGGAAA
AGGGTTCACGGCTTATCTCTAGCATGATGCTCAAGTTGTACGGCAAGGGAGACTCTGCACGAGGTG
GCTTTGGCCAGACGATCCACACCACTCTCCCCTTTGTCAAGTCAGATCTTCCCGTTGCCATTGTTTTCC
GTGCCTTGGGTGTCGTTTCTGATGAGGACATTCTGAACCACATTTGCTACGACCGAAACGACAGCCA
GATGCTAGAAATGCTTCGGCCGTGTATCGAGGAGGCCTTTTGTGTTCAGGACCGAGAGGTTGCTCTT
GACTTCATCGGAAAGCGAGGCAATCGCGACCAAGCTGGTCTCGGTCGTGAGAAGCGTGTCCGTGTG
GCTAAGGACATTCTTCAAAAAGAGACCCTTCCTCACATTTCTCAGACGGAAGGCAGTGAAACCAGAA
AGGCATTCTTCCTGGGATACATGGTCCACAAGCTGTTGCAATGCGCTCTCGGAAGACGTGAGCCCGA
CGATCGTGACCACTTTGGAAAGAAGCGTCTGGATCTGGCGGGTCCCCTGCTAGCTAAGCTGTTCCGT
GGTATCATGCGAAGGATGAACACCGAGTTGGCCAATTATCTGAGACGTTGTGTGGAGGGCAACCGA
CATTTCAACCTTGCTGTGGGTATCAAGCCCGGCACGCTTTCAAACGGTCTGAAGTATTCTCTTGCCAC
TGGAAACTGGGGTGACCAGAAGAAGGCCATGAGCTCGACCGCAGGTGTGTCTCAGGTGCTCAATC
GCTACACTTTTGCTTCGACCCTGTCACATTTGCGTCGTACCAACACACCCATCGGAAGAGATGGTAA
GCTGGCGAAGCCTCGACAGCTTCATAATACGCATTGGGGTCTGGTCTGCCCAGCCGAGACACCTGA
AGGCCAAGCTTGTGGTTTGGTCAAAAACTTGTCTTTGATGTGCTACGTCAGTGTCGGATCTCCTTCCG
AACCCCTGATCGAGTTCATGATCAACAGAGGTATGGAGGTCGTCGAAGAGTATGAGCCGTTGCGGT
ATCCCCACGCTACGAAGATCTTTGTCAACGGTGTATGGGTGGGAGTTCATCAAGATCCCAAGCACCT
GGTGAACCAGGTTCTGGACACTCGTCGCAAATCCTATCTGCAGTATGAAGTCTCCCTGATCAGGGAC
ATTCGTGACCAGGAATTCAAAATCTTCTCCGACGCAGGTCGTGTTATGCGTCCCGTCTTTACTGTTCA
GCAAGAAGACGACCCAGAAACTGGCATTAATAAGGGCCATTTGGTTTTGACAAAGGAGCTGGTCAA
CAGGCTGGCAAAGGAGCAGGCCGAGCCTCCGGAGGACCCGAGCATGAAGATTGGATGGGAGGGG
TTAATTAGGGCTGGTGCGGTTGAATATCTCGATGCCGAGGAAGAAGAGACGGCAATGATTTGCATG
ACCCCCGAGGATCTCGAGCTTTATCGTCTTCAGAAAGCTGGTATATCCACAGAAGAAGACATGGCAG
ATGATCCAAACAAGCGACTCAAGACGAAGACGAATCCAACAACTCACATGTACACGCATTGTGAGA
TTCACCCGAGTATGATCTTAGGCATCTGTGCTAGTATCATTCCTTTCCCTGATCACAACCAGTCCCCCC
GTAACACCTACCAATCTGCCATGGGTAAACAAGCCATGGGCTTCTTCCTAACTAACTATTCTCGGCGT
ATGGATACCATGGCCAACATTCTCTACTATCCCCAGAAGCCGCTGGGCACCACCCGTTCTATGGAGT
TTTTGAAATTCCGTGAACTGCCAGCTGGACAGAATGCCATTGTAGCCATTGCTTGTTACTCTGGCTAC
AATCAAGAAGATTCCGTCATTATGAACCAGAGTAGTATCGACAGAGGTCTCTTCCGAAGTTTGTTCTT
CCGATCATACTCGGATCAGGAGAAGAAGGTTGGGTTGAACTACACTGAAGTGTTTGAGAAGCCATT
CCACCAGAATACCCTTCGTATGAAGCACGGTACATATGACAAGCTAGACGAAGATGGTATCGTTGCT
CCTGGCGTTCGAGTGTCCGGTGAAGACATCATCATCGGCAAGACTGCGCCAATTGATGCAGAGACA

CAGGATCTTGGCACCAGGACGACCATGCACCAGAGGCGTGATATCTCAACGCCTCTGCGCAGTACT
GAGAACGGTATCGTTGATTCCGTCATCGTGACGGTGAACGCGGACAATGTCAAGTATGTCAAGGTC
CGTGTTCGAACGACCAAGATTCCCCAGATTGGAGACAAGTTTGCCTCTCGTCACGGTCAGAAGGGC
ACTATTGGTGTCACCTACCGACAAGAGGATATGCCGTTCACAAGGGAAGGCGTTACCCCCGACATCA
TCATCAACCCTCACGCCATTCCATCTCGTATGACAATTGCTCACTTGATTGAGTGTCTCCTGAGTAAG
GTCTCTACGTTGGAAGGTATGGAGGGCGATGCCACCCCCTTCACGGACGTAACGGTCGACTCGGTC
TCAGAGCTTCTGCGAAAGCACGGCTACCAGTCGCGAGGTTTTGAGATTATGTACAATGGTCATACAG
GTCGCAAGCTGAGAGCACAAGTGTTCTTCGGGCCAACGTACTACCAGCGACTGCGCCACATGGTGG
ATGACAAGATCCATGCCCGAGCTCGTGGTCCCGTGCAGATCATGACCAGACAGCCAGTGGAGGGTC
GTGCCCGAGACGGTGGTCTCCGATTCGGAGAAATGGAACGTGATTGTATGATTGCACACGGTGCAG
CATCTTTCCTCAAGGAGCGATTGTTTGAGGTGTCAGACGCATTCCGAGTCCACATTTGCGAGATTTGT
GGACTCATGACACCTATTGCTAACCTCTCCAAGCAATCGTTCGAGTGCCGACCATGCAAGAACAAGA
CCAAGATTGCGCAGATTCATATCCCTTACGCTGCCAAGCTCTTGTTCCAGGAGCTACAGTCTATGAAC
ATTGCGGCCAGAATGTTCACAAACCGGTCCGGCGCGTCCGTTAGGTAG
>Trichoderma asperellum
ATGGCTGATTACGAAGACGATTACGATTATGAGAACTATGGGGATGAGGATGAGGGCATCACGCCC
GAGGATTGCTGGACCGTGATTTCCTCTTTCTTCGAGACCAAGGGTCTCGTATCGCAGCAGACCGACT
CCTTTGACGAGTTTACTCAGACGACCATCCAGGATCTCGTTAACGAATACTCCACCATCACGCTCGAT
CAGCCCAATCCTCCTTCGCCACCTGGTCGAACGATAGCCCTTCGCCGATATGAAATCAAATTTGGAA
GCGTCATGGTATCACGCCCCACTATCAGCGAAACAGATGGAACTGTGACATCTTTGCTTCCTTACGA
ATGCCGAGACCGTAACTTGACCTACGCCAGTCCGCTCTACATCAAAATCACCAAAAAGGTGTCTGCG
GCTGTCGAAAGAGAGGTTCCGCTGCACGAGATGGACGATGCCCAGCAAGAACAGTATGCAAGGAC
CGGAGAGCACCCTACAAAGTTGGAATGGGAAGAGGAAGAAAATGGCGAAGACGACAATCTCGGCA
AGTCTGACGACTGGAAGGACATGGTTTTCGTTGGCAAGCTGCCCATCATGGTCAAATCCAAGATTTG
TCATCTGAGCCGTGAACAGGATGATAGCCTGTTCCTTGTCAACGAGTGCCCTTACGACCAAGGTGGA
TATTTCGTCATCAACGGTAGTGAAAAGGTCCTCATTGCCCAAGAGCGCTCCGCCGCAAACATCGTCC
AGGTCTTCAAGAAGGCCCAGCCCAGTGCTTATACCTACACGGCCGAAATCCGAAGTGCACTGGAAA
AGGGATCTCGACTCATCTCTAGCATGATGCTCAAGTTGTATGGCAAAGGAGACTCTGCGCGAGGTG
GTTTTGGACAGACCATTCACACAACCCTGCCTTTTGTCAAGTCAGATCTTCCCGTCGCCATTGTTTTCC
GTGCCTTGGGTGTCGTTTCTGATGAAGACATTCTGAATCACATTTGCTACGATCGAAACGACAGCCA
AATGCTTGAAATGCTTCGGCCCTGTATTGAAGAAGCCTTCTGCGTCCAGGATCGAGAAGTTGCTCTC
GATTTCATCGGAAAGCGAGGCAACCGTGATCAAGCTGGCCTCGGTCGTGAGAAGCGTGTTCGTGTA
GCGAAGGACATTCTCCAGAAGGAGACGCTTCCCCACATTTCCCAGACTGAAGGTAGCGAGACGAGA
AAGGCATTCTTCCTCGGATACATGGTGCACAAGCTATTGCAATGCGCACTTGGAAGACGAGAGCCC
GATGACCGAGATCACTTCGGAAAGAAGCGTCTGGATCTGGCTGGTCCACTGCTGGCCAAGCTGTTC
CGTGGTATCATGCGTAGAATGAACACCGAGTTGGCCAACTACCTGAGACGATGTGTTGAGGGTAAC
CGTCACTTCAACCTGGCTGTTGGCATCAAGCCCGGCACACTCTCCAACGGATTGAAGTATTCTCTCGC
CACCGGAAACTGGGGTGACCAGAAGAAGGCAATGAGCTCGACCGCAGGTGTATCACAGGTGCTTA
ACCGTTACACTTTTGCTTCGACACTTTCTCATTTGCGTCGTACCAATACACCCATCGGAAGAGATGGT
AAGCTGGCGAAGCCTCGACAGCTTCACAACACACACTGGGGTTTGGTATGCCCGGCCGAGACCCCT
GAGGGACAGGCTTGTGGTTTGGTCAAAAACTTGTCTCTGATGTGCTACGTCAGTGTCGGATCTCCTT
CCGAGCCTTTGATCGAGTTTATGATCAACAGAGGTATGGAGGTCGTTGAGGAGTACGAACCGCTGA
GGTATCCCCATGCGACAAAGATTTTTGTGAATGGTGTCTGGGTTGGAATCCACCAAGACCCCAAGCA
TCTGGTAAACCAAGTTTTGGACACTCGTCGTAAATCCTATCTGCAATACGAAGTCTCTCTGATCAGAG
ATATTCGTGACCAAGAATTCAAAATCTTCTCTGACGCCGGTCGTGTTATGCGTCCTGTCTTTACTGTA
CAGCAAGAGGATGACCCGGAAACGGGCATCAACAAGGGCCACCTGGTTTTAACCAAGGATCTTGTC
AATAGACTGGCCAAAGAGCAGGCTGAGCCTCCGGAAGACCCAAGCATGAAGCTTGGATGGGAAGG

GTTAATTCGGGCTGGTGCGGTGGAATATCTCGATGCTGAGGAAGAAGAAACGGCTATGATTTGCAT
GACACCGGAGGATCTTGAGCTTTACCGTCTTCAGAAGGCTGGCATTGCCACAGATGAAGACATGGG
AGACGATCCAAACAAGCGTCTCAAGACCAAGACAAATCCGACAACTCACATGTACACGCATTGCGA
GATTCACCCGAGTATGATCTTAGGTATCTGTGCTAGTATCATTCCTTTCCCCGATCACAACCAGTCCCC
CCGTAACACCTACCAATCTGCCATGGGTAAACAAGCCATGGGCTTCTTCTTGACCAACTATTCTCGGC
GTATGGACACCATGGCCAATATCCTCTACTACCCTCAGAAACCGCTGGGCACCACTCGGTCTATGGA
GTTCTTGAAATTCCGTGAGCTGCCAGCCGGACAAAACGCCATTGTAGCAATTGCTTGTTACTCTGGTT
ATAACCAAGAAGATTCCGTCATTATGAACCAGAGTAGTATTGACAGAGGTCTCTTCCGAAGTCTTTTC
TTCCGATCATATTCAGATCAGGAGAAAAAGGTTGGCTTGAACTACACGGAAGTGTTTGAGAAGCCAT
TCCACCAAAACACCCTTCGTATGAAGCACGGCACATATGATAAGCTGGATGAAGATGGTATCGTTGC
TCCTGGTGTCCGAGTGTCAGGTGAGGATATCATCATCGGCAAGACGGCGCCAATCGACCCCGAGAC
CCAGGATCTTGGAACCAGGACGACCATGCACCAGAGACGCGATATCTCAACGCCTCTGCGAAGTAC
CGAGAACGGTATCGTTGACCAGGTCATCGTCACGGTGAACGCGGACAATGTTAAGTATGTCAAGGT
CCGTGTTCGAACGACCAAGATTCCCCAGATTGGAGACAAGTTTGCTTCTCGTCACGGTCAGAAGGGC
ACTATTGGTGTTACTTACCGACAGGAGGATATGCCTTTCACAAGAGAAGGTCTCACTCCAGATATCA
TTATCAACCCCCACGCCATTCCGTCTCGTATGACAATTGCTCACTTGATTGAGTGTCTCCTCAGTAAG
GTCTCTACGTTGGAAGGTATGGAGGGTGATGCTACTCCCTTCACGGATGTCACGGTCGACTCAGTCT
CTGAGCTCTTGCGAAAGCACGGTTACCAGTCTCGAGGTTTCGAGATTATGTACAACGGCCATACTGG
CCGCAAGCTAAGAGCACAAGTGTTCTTTGGACCAACATACTACCAGCGACTCCGTCACATGGTGGAT
GACAAGATCCATGCCAGAGCTCGCGGTCCTGTGCAGATCATGACAAGACAGCCGGTGGAGGGTCGT
GCTCGAGATGGTGGTCTCCGATTCGGAGAAATGGAACGTGATTGTATGATTGCACACGGTGCGGCG
TCCTTCCTCAAGGAGCGATTGTTTGAGGTGTCGGACGCCTTCAGAGTTCACATTTGCGAGATTTGTG
GACTCATGACACCCATTGCTAACTTATCCAAACAATCGTTCGAGTGCCGGCCATGCAAGAACAAGAC
TAAGATTGCACAGATTCACATTCCTTATGCTGCCAAGCTCTTGTTCCAAGAGCTCCAGTCTATGAACA
TTGCAGCTAGAATGTATACAAACCGATCTGGTGCATCTATCCGATAG
>Trichoderma atrobrunneum
ATGGCTGACTACGAGGACGAATACGACTACGAGAACTACGGAGACGAGGATGAAGGCATCACTCC
CGAGGATTGCTGGACCGTCATCTCCTCCTTCTTCGAAACCAAGGGTCTTGTATCGCAGCAGACCGAC
TCCTTTGACGAATTCACCCAGACCACGATCCAGGATCTCGTAAACGAATACTCCACCATCACACTCGA
CCAGCCGAACCCTCCTTCGCCACCCGGTCGAACAATAGCCCTTCGTCGATATGAGATCAAGTTTGGA
AGCGTCATGGTTTCACGTCCCACTATCAGCGAGACGGACGGAACTGTGACTTCTCTGCTCCCTTATG
AGTGCCGAGACCGCAACTTGACCTACGCCAGTCCCCTCTATATCAAGATTACTAAAAAGGTGTCGGC
TGCCGTCGAGAGAGAGGTTCCCCTGCACGAAATGGACGATGCTCAGCAGGAAGAATACGCAAGAA
CCGGCGAACACCCTACAAAGCTTGAGTGGGAAGAGGAGGAGAACGGCGAAGATGACAACATCGGC
AAGTCTGATGATTGGAAGGACATGGTCTTCGTTGGCAAGCTGCCCATCATGGTCAAATCCAAGATTT
GTCATCTGAGCCGTGAACAGGATGACAGCCTGTTCCTTGTCAACGAATGTCCCTACGATCAGGGTGG
CTACTTTGTTATCAACGGCAGTGAAAAGGTTCTCATCGCCCAAGAGAGATCTGCCGCAAACATTGTC
CAAGTCTTCAAAAAGGCCCAGCCCAGTGCCTATACCTATACGGCTGAAATCCGAAGTGCGCTGGAAA
AGGGCTCACGGCTCATCTCCAGCATGATGCTCAAGCTGTATGGCAAGGGAGACTCTGCTCGTGGTG
GCTTTGGCCAAACTATCCACACCACCCTGCCCTTTGTCAAGTCAGATCTTCCCGTCGCCATTGTCTTCC
GTGCCCTAGGTGTCGTTTCTGATGAAGATATCCTCAACCACATTTGCTACGACCGCAACGACAGCCA
GATGCTGGAGATGCTTCGACCTTGTATTGAGGAGGCCTTTTGTGTTCAGGACCGAGAGGTTGCTCTC
GATTTCATCGGAAAGCGTGGAAACCGAGACCAAGCTGGTCTCGGACGTGAGAAGCGTGTCCGCGT
GGCCAAGGATATCCTTCAGAAGGAGACTCTTCCTCACATTTCACAGACAGAGGGAAGTGAAACCAG
AAAGGCATTCTTCCTGGGATACATGGTGCACAAGCTGTTGCAATGTGCGCTCGGAAGAAGAGAGCC
TGACGATCGTGACCACTTTGGAAAGAAGCGTCTGGATCTGGCCGGTCCCCTGCTGGCCAAGCTGTTC
CGTGGTATCATGCGAAGGATGAACACTGAGTTGGCCAACTACCTGAGACGATGCGTTGAGGGCAAC

CGACATTTCAACCTTGCTGTTGGTATCAAGCCCGGCACGCTTTCAAACGGATTGAAGTATTCGCTTGC
CACAGGCAACTGGGGTGATCAGAAGAAGGCCATGAGCTCAACTGCAGGTGTGTCCCAGGTGCTTAA
CCGTTACACGTTTGCTTCGACCTTGTCGCATTTGCGTCGTACCAATACTCCTATCGGAAGAGATGGTA
AGCTGGCAAAGCCTCGACAGCTTCACAACACGCATTGGGGTTTGGTCTGCCCAGCCGAGACACCCG
AAGGACAGGCTTGTGGTCTGGTCAAGAACTTGTCTTTGATGTGTTACGTCAGTGTCGGTTCTCCCTCT
GAACCTCTCATTGAGTTCATGATCAACAGAGGTATGGAAGTCGTCGAAGAGTACGAGCCTCTGCGG
TATCCTCATGCTACAAAGATTTTTGTGAACGGTGTCTGGGTTGGAGTCCACCAAGACCCTAAGCACTT
GGTGAACCAGGTTCTGGACACTCGTCGCAAGTCCTATCTGCAATACGAAGTCTCTCTCGTGAGAGAA
ATTCGAGACCAGGAATTCAAAATCTTTTCCGACGCTGGCCGTGTCATGCGACCAGTCTTTACCGTTCA
ACAGGAAGATGACCCGGAAACGGGCATCAACAAGGGCCACCTGGTATTGACCAAGGAGCTCGTCA
ATAGATTGGCCAAGGAGCAGGCTGAGCCTCCGGAAGACCCCAGCATGAAGATTGGATGGGAGGGA
TTGATCAGGGCTGGTGCGGTTGAATATCTCGACGCCGAGGAAGAGGAGACGTCCATGATCTGCATG
ACGCCAGAGGATCTCGAGCTGTATCGTCTTCAGAAGGCCGGTATTAACACTGAGGAAGACATGGGA
GATGACCCGAACAAGCGACTAAAGACCAAGACCAACCCGACAACTCACATGTACACCCATTGCGAG
ATTCACCCAAGTATGATCTTAGGCATCTGTGCTAGTATCATTCCTTTCCCCGATCACAACCAGTCCCCC
CGTAACACTTACCAATCTGCCATGGGTAAGCAAGCTATGGGTTTCTTCCTCACGAACTATTCCCGGCG
CATGGACACCATGGCCAACATCCTTTACTACCCCCAGAAGCCGCTGGGTACCACTCGATCTATGGAG
TTTTTGAAATTCCGAGAGTTGCCTGCTGGACAGAACGCCATTGTAGCCATTGCCTGTTACTCTGGTTA
CAACCAGGAAGATTCCGTCATTATGAACCAAAGTAGTATTGACAGAGGTCTGTTCCGAAGTCTCTTC
TTCCGATCATACTCGGATCAAGAGAAGAAGGTTGGATTGAACTACACCGAAGTGTTTGAGAAGCCCT
TCCACCAAAACACTCTTCGCATGAAGCACGGTACATACGACAAGCTGGATGAAGACGGTATCGTCGC
TCCTGGTGTCCGAGTGTCTGGTGAAGACATCATTATTGGCAAGACGGCGCCGATTGACGCTGAGAC
ACAAGATCTCGGTACCAGGACGACTATGCACCAGAGGCGAGATATCTCAACTCCTCTGCGTAGCACC
GAGAACGGTATCGTTGACTCCGTCATTGTGACGGTGAACGCGGACAATGTCAAGTATGTCAAGGTC
CGTGTCCGAACGACCAAGATTCCCCAGATTGGAGACAAGTTCGCCTCTCGTCACGGTCAAAAGGGC
ACTATTGGTGTTACTTACCGACAAGAGGATATGCCCTTCACAAGAGAAGGTGTCACTCCAGACATCA
TCATCAACCCTCACGCCATTCCATCCCGTATGACAATCGCTCACTTGATTGAGTGTCTCCTAAGTAAG
GTCTCTACGCTGGAAGGTATGGAGGGTGATGCCACCCCCTTTACGGACGTCACCGTCGACTCAGTCT
CAGAGCTGTTGCGAAAGCACGGCTACCAGTCACGAGGCTTTGAGATTATGTACAATGGCCATACAG
GCCGCAAGCTGAGAGCCCAGGTGTTCTTCGGACCGACATACTACCAGCGACTCCGTCACATGGTGG
ATGATAAGATCCACGCTCGTGCCCGTGGTCCCGTACAGATTATGACTAGACAACCTGTGGAGGGTC
GTGCGAGAGATGGTGGTCTCCGATTCGGAGAAATGGAACGTGATTGCATGATTGCTCACGGTGCTG
CCTCCTTCCTCAAGGAGCGATTGTTCGAGGTGTCAGACGCCTTCCGAGTTCACATTTGCGAGATTTGT
GGACTCATGACGCCTATTGCCAACCTCTCTAAGCAATCGTTTGAGTGTCGACCTTGTAAGAACAAGA
CCAAGATTGCGCAGATTCACATCCCTTATGCTGCCAAGCTCCTGTTCCAGGAACTCCAGTCGATGAAC
ATTGCGGCTCGCATGTTCACAAATCGGTCTGGCGCGTCCGTCAGGTAA
>Trichoderma atroviride
ATGGCTGATTACGAAGACGATTACGACTATGAGAACTATGGGGATGAGGATGAGGGCATCACGCCC
GAGGATTGCTGGACTGTGATTTCCTCCTTCTTCGAGACCAAGGGCCTCGTATCACAGCAGACCGACT
CCTTTGACGAGTTCACCCAGACGACAATCCAGGATCTCGTCAACGAATACTCCACCATCACACTCGAC
CAGCCCAATCCTCCTTCGCCACCTGGTCGAACGATAGCCCTTCGCCGATATGAAATCAAATTTGGAA
GCGTCATGGTATCACGTCCCACTATCAGTGAGACGGATGGAACTGTGACGTCTTTGCTCCCTTACGA
ATGTCGAGACCGCAACCTGACTTACGCCAGTCCGCTGTACATCAAGATCACCAAGAAAGTGTCTGCG
GCCGTCGAGAGGGAGGTTCCGCTGCATGAGATGGATGATGCCCAGCAGGAACAGTATGCAAGGAC
CGGAGAAAACCCCACAAAGCTGGAATGGGAAGAGGAAGAGAATGGCGAAGACGACAATCTCGGC
AAGTCTGACGACTGGAAGGACATGGTTTTCGTTGGCAAGCTGCCCATCATGGTCAAGTCCAAGATTT
GTCATCTGAGCCGTGAACAGGATGACAGCCTGTTCCTCGTCAACGAGTGCCCTTATGACCAAGGAG

GCTACTTCGTTATCAACGGTAGTGAAAAGGTCCTCATCGCCCAGGAGCGTTCCGCCGCAAACATCGT
CCAGGTCTTCAAGAAGGCCCAGCCCAGTGCCTATACCTACACGGCCGAAATCCGAAGTGCGCTGGA
AAAGGGATCTCGACTCATCTCCAGCATGATGCTCAAGTTGTACGGCAAAGGAGACTCTGCGCGAGG
TGGTTTTGGGCAAACGATTCACACTACCCTGCCTTTTGTCAAGTCAGATCTTCCCGTTGCCATTGTTTT
CCGTGCCTTGGGCGTCGTTTCTGATGAGGACATTCTGAACCACATCTGCTACGACCGAAACGACAGC
CAAATGCTCGAAATGCTTCGGCCTTGCATTGAAGAAGCCTTTTGTGTTCAGGATCGAGAAGTTGCCC
TTGATTTCATCGGAAAGCGAGGCAACCGTGATCAAGCCGGCCTCGGTCGTGAGAAGCGTGTTCGTG
TAGCAAAGGACATTCTCCAGAAGGAGACGCTTCCCCACATTTCCCAGACTGAAGGCAGTGAGACCA
GAAAGGCATTCTTCCTTGGATACATGGTGCACAAGCTATTGCAATGCGCACTTGGAAGACGAGAGC
CCGACGACCGAGACCACTTTGGAAAGAAGCGTCTAGATCTGGCGGGTCCACTGCTGGCCAAGCTGT
TCCGTGGTATCATGCGCAGAATGAATACAGAGCTGGCCAACTACCTCAGACGATGTGTTGAGGGTA
ACCGCCACTTCAACCTTGCTGTTGGCATTAAGCCCGGCACACTTTCCAACGGACTAAAGTACTCGCTC
GCCACTGGAAACTGGGGTGACCAGAAGAAGGCAATGAGCTCGACCGCAGGTGTCTCACAGGTGCTT
AACCGTTACACTTTTGCTTCTACACTTTCCCATTTGCGTCGTACCAATACACCCATCGGAAGAGATGG
TAAGCTGGCGAAGCCTCGACAGCTCCACAACACACACTGGGGTTTGGTGTGCCCGGCTGAGACCCC
TGAAGGTCAAGCTTGTGGTCTGGTCAAGAATTTGTCTCTCATGTGCTACGTCAGTGTTGGATCTCCTT
CTGAGCCTTTGATCGAGTTTATGATCAACAGAGGTATGGAAGTCGTTGAGGAGTATGAGCCACTGA
GGTATCCCCATGCTACAAAGATCTTTGTGAATGGTGTCTGGGTTGGAATCCACCAAGACCCCAAGCA
TCTGGTAAACCAAGTTTTGGATACTCGTCGCAAATCCTATCTGCAGTACGAGGTCTCTCTGATCAGAG
AAATTCGAGACCAAGAATTCAAAATCTTCTCTGACGCCGGCCGTGTCATGCGTCCCGTCTTCACTGTA
CAGCAGGAAGATGACCCGGAAACGGGTATCAACAAGGGCCACCTGGTTTTGACCAAGGACCTTGTC
AATAGACTGGCCAAAGAGCAGGCTGAGCCTCCAGAAGACCCAAGCATGAAGCTCGGATGGGAGGG
GCTGATTAGGGCTGGTGCGGTGGAATATCTCGACGCCGAGGAAGAAGAAACGTCCATGATTTGCAT
GACACCGGAAGATCTTGAGCTCTATCGTCTTCAAAAGGCCGGCATTGCCACAGATGAAGACATAGG
AGATGACCCAAATAAGCGTCTCAAGACCAAGACAAATCCGACAACTCACATGTATACACATTGCGAG
ATTCACCCGAGTATGATCTTAGGTATCTGTGCTAGTATCATTCCTTTCCCCGATCACAACCAGTCCCCC
CGTAACACCTACCAGTCTGCCATGGGTAAACAAGCCATGGGCTTCTTCCTAACCAACTATTCTCGGCG
TATGGACACCATGGCCAATATCCTCTACTACCCTCAGAAACCGCTGGGCACCACTCGTTCTATGGAGT
TTTTGAAATTCCGTGAGCTGCCAGCCGGACAAAACGCCATTGTAGCAATTGCTTGTTACTCTGGTTAT
AACCAAGAAGATTCCGTCATTATGAACCAGAGTAGTATTGACAGAGGTCTCTTCCGAAGTCTTTTCTT
CCGATCATATTCAGATCAAGAGAAGAAGGTTGGCTTGAACTACACGGAAGTGTTTGAGAAGCCATT
CCACCAAAACACTCTTCGCATGAAGCACGGCACATATGACAAGCTGGACGAAGATGGTATCGTTGCT
CCTGGTGTTCGTGTGTCAGGTGAGGATATCATCATCGGTAAGACAGCACCAATCGACGCCGAGACA
CAGGATCTTGGAACCAGGACGACCATGCACCAAAGACGCGATATCTCGACGCCTTTGCGAAGTACC
GAGAATGGTATCGTCGACCAGGTTATCGTGACGGTGAACGCGGATAATGTTAAATATGTCAAGGTC
CGTGTTCGAACGACCAAGATTCCCCAGATTGGAGACAAGTTTGCCTCTCGTCACGGTCAGAAGGGC
ACTATTGGTGTTACCTACCGACAGGAGGACATGCCATTCACCAGAGAAGGTCTCACTCCAGATATCA
TTATCAACCCCCACGCCATTCCGTCTCGTATGACAATTGCTCACTTGATTGAGTGTCTCCTGAGTAAG
GTCTCTACGTTGGAAGGTATGGAGGGTGATGCCACTCCCTTTACGGATGTCACCGTCGATTCAGTCT
CTGAGCTCTTGCGAAAGCACGGCTACCAGTCTCGAGGCTTCGAAATCATGTACAACGGCCATACTGG
ACGCAAGTTAAGAGCACAAGTATTCTTTGGACCAACATACTACCAGCGACTCCGCCACATGGTGGAC
GACAAGATCCATGCCAGAGCTCGTGGTCCTGTGCAGATCATGACACGGCAGCCAGTGGAGGGTCGT
GCTCGAGATGGTGGTCTCCGATTCGGAGAAATGGAACGTGATTGTATGATTGCACACGGTGCAGCG
TCCTTCCTCAAGGAGCGATTGTTTGAGGTGTCGGACGCCTTCCGAGTTCACATTTGCGAGATTTGTG
GACTCATGACACCCATTGCTAACTTATCCAAACAATCGTTTGAGTGTCGGCCATGCAAGAACAAGAC
AAAGATTGCACAGATTCACATTCCTTACGCTGCCAAGCTCTTATTCCAGGAGCTCCAGTCAATGAACA
TTGCAGCTAGAATGTACACCAACCGGTCTGGTGCATCTGTTCGGTAG

>Trichoderma sp. T154
ATGGCTGACTACGAGGACGAATACGACTACGAGAACTATGGAGACGAGGATGAAGGCATCACGCC
CGAGGATTGCTGGACCGTCATCTCCTCCTTCTTCGAAACCAAGGGTCTTGTGTCGCAGCAGACCGAC
TCCTTTGACGAATTCACCCAGACGACGATCCAGGATCTCGTAAACGAATATTCCACCATCACACTCGA
CCAGCCAAACCCTCCTTCGCCACCCGGTCGAACAATAGCCCTTCGTCGATATGAGATCAAGTTTGGA
AGCGTCATGGTGTCACGTCCCACTATCAGTGAAACGGACGGAACTGTGACTTCTCTGCTCCCTTACG
AGTGCCGAGACCGCAACTTGACCTACGCCAGTCCCCTCTACATCAAGATTACTAAAAAGGTGTCCGC
TGCCGTCGAGAGGGAGGTTCCCCTGCACGAAATGGACGATGCTCAGCAGGAGGAATACGCAAGAA
CCGGCGAACACCCTACAAAACTTGAGTGGGAGGAGGAGGAGAACGGCGAAGATGACAACATCGGC
AAGTCTGATGACTGGAAAGACATGGTCTTCGTTGGCAAGCTGCCCATCATGGTCAAATCCAAGATTT
GTCATCTGAGCCGTGAACAGGATGATAGCCTGTTCCTTGTCAACGAATGTCCCTACGATCAGGGTGG
CTACTTTGTTATCAACGGCAGTGAAAAGGTTCTCATCGCCCAAGAGAGATCTGCCGCCAACATTGTA
CAAGTCTTCAAAAAGGCCCAGCCCAGTGCCTACACCTATACGGCTGAAATCCGAAGTGCGTTGGAAA
AGGGCTCACGGCTCATCTCCAGCATGATGCTCAAGCTGTATGGCAAGGGAGACTCTGCTCGTGGTG
GCTTTGGCCAGACTATCCACACCACTCTGCCCTTTGTCAAGTCAGATCTTCCCGTCGCCATTGTCTTCC
GTGCCCTAGGTGTCGTTTCTGATGAAGATATCCTCAACCACATTTGCTACGACCGCAACGACAGCCA
GATGCTGGAGATGCTTCGACCTTGTATTGAGGAGGCCTTCTGTGTCCAGGATCGAGAAGTTGCTCTC
GATTTCATCGGAAAGCGAGGAAACCGAGACCAAGCTGGTCTCGGACGCGAGAAACGTGTCCGCGT
GGCCAAGGATATCCTTCAGAAGGAGACTCTTCCCCACATTTCACAGACAGAGGGAAGTGAAACCAG
AAAGGCGTTCTTCCTGGGATACATGGTGCACAAGCTGTTGCAATGTGCGCTCGGAAGAAGAGAGCC
GGACGATCGTGACCACTTTGGAAAGAAGCGTCTGGATCTGGCGGGTCCCCTGCTGGCCAAGCTGTT
CCGTGGTATCATGCGAAGGATGAACACTGAGTTGGCCAACTACCTGAGACGATGCGTTGAGGGCAA
CCGACACTTCAACCTGGCTGTTGGTATCAAGCCCGGCACGCTTTCAAACGGACTGAAGTATTCGCTT
GCCACAGGAAACTGGGGTGATCAGAAGAAGGCCATGAGCTCAACTGCCGGTGTGTCCCAGGTGCTT
AACCGTTACACGTTTGCTTCGACCTTGTCACATTTGCGTCGTACCAACACTCCTATCGGGAGAGATGG
TAAGCTGGCGAAGCCTCGACAGCTTCACAACACGCATTGGGGCTTGGTCTGCCCAGCCGAGACACC
CGAAGGACAGGCCTGTGGTCTGGTCAAGAACTTGTCTTTGATGTGTTACGTCAGTGTCGGTTCTCCC
TCCGAGCCTCTGATTGAGTTCATGATCAACAGAGGTATGGAAGTCGTCGAAGAGTACGAGCCGCTG
CGGTATCCTCATGCTACAAAGATTTTTGTGAACGGTGTCTGGGTTGGAGTTCACCAAGACCCTAAGC
ACTTGGTGAACCAGGTTCTGGATACTCGTCGCAAGTCCTATCTGCAATACGAAGTCTCTCTCGTGAG
AGAAATTCGAGACCAGGAATTCAAAATCTTTTCCGATGCAGGTCGTGTCATGCGACCAGTCTTTACC
GTTCAGCAGGAAGATGATCCGGAAACGGGCATCAACAAGGGCCACCTGGTATTGACCAAGGAGCTC
GTCAATAGATTGGCCAAGGAGCAGGCTGAGCCTCCGGAAGACCCCAGCATGAAGATTGGATGGGA
GGGATTGATCAGGGCTGGTGCGGTTGAATATCTCGACGCCGAGGAAGAGGAGACGGCCATGATCT
GCATGACACCAGAGGATCTCGAGCTGTATCGTCTTCAGAAGGCCGGTATCAACACTGAGGAAGACA
TGGGAGATGATCCGAACAAGCGACTCAAGACCAAGACGAACCCGACAACTCACATGTACACCCATT
GCGAGATTCACCCAAGTATGATCTTAGGTATCTGTGCTAGTATCATTCCTTTCCCCGATCACAACCAG
TCCCCCCGTAACACTTACCAATCTGCCATGGGTAAGCAAGCTATGGGTTTCTTCCTCACGAATTATTC
TCGGCGCATGGACACCATGGCCAATATCCTTTACTACCCCCAGAAGCCGCTGGGTACCACTCGATCC
ATGGAGTTTTTGAAATTCCGAGAGTTGCCTGCTGGTCAGAACGCCATTGTAGCCATTGCCTGTTACTC
TGGTTACAACCAGGAAGATTCCGTCATTATGAACCAGAGTAGTATTGATAGAGGTCTTTTCCGAAGT
CTCTTCTTCCGATCATACTCTGATCAGGAGAAGAAGGTTGGGTTGAACTATACCGAAGTGTTTGAGA
AGCCCTTCCACCAAAACACTCTTCGCATGAAGCACGGCACATACGACAAGCTGGATGAAGACGGTAT
CGTTGCTCCTGGTGTCCGAGTATCTGGTGAGGACATCATTATTGGCAAGACGGCGCCGATTGACGCT
GAGACACAAGATCTCGGTACCAGGACAACTATGCACCAGAGGCGAGATATCTCAACTCCCCTGCGT
AGCACCGAGAACGGTATCGTTGACTCTGTCATTGTGACAGTGAACGCGGACAATGTCAAGTATGTC
AAGGTCCGTGTCCGAACGACCAAGATTCCCCAGATTGGAGACAAGTTCGCCTCTCGTCACGGTCAAA

AGGGCACTATTGGTGTTACTTACCGACAAGAGGACATGCCCTTCACAAGAGAAGGTGTCACTCCGG
ACATCATCATCAACCCTCACGCCATTCCGTCCCGTATGACAATCGCTCACTTGATTGAGTGTCTCCTAA
GTAAGGTCTCTACGCTGGAAGGTATGGAGGGTGATGCCACTCCCTTTACGGACGTCACGGTCGACT
CGGTCTCAGAGCTGCTGCGAAAGCACGGCTACCAGTCACGAGGCTTTGAGATTATGTACAATGGCC
ACACAGGCCGCAAGCTCAGAGCCCAGGTGTTCTTTGGACCTACATACTACCAACGACTCCGTCACAT
GGTGGACGACAAGATCCACGCTCGTGCCCGTGGTCCCGTGCAGATCATGACCAGACAACCTGTGGA
GGGTCGTGCCAGAGATGGTGGTCTCCGATTCGGAGAGATGGAACGTGATTGTATGATTGCTCACGG
TGCCGCGTCCTTCCTCAAGGAGCGATTGTTTGAGGTGTCTGACGCCTTCCGAGTTCACATTTGCGAG
ATTTGTGGACTCATGACGCCTATTGCCAACCTCTCTAAGCAATCGTTCGAATGTCGACCTTGTAAGAA
CAAGACCAAGATTGCGCAGATTCACATCCCCTATGCTGCCAAGCTTCTGTTCCAGGAACTCCAGTCG
ATGAACATTGCGGCTCGCATGTTCACAAACCGGTCTGGCGCGTCTATCAGGTAA
>Trichoderma citrinoviride
ATGGCTGACTACGAGGACGACTACGACTACGAAAATTACGGAGATGAGGATGAGGGCATCACCCCC
GAGGACTGCTGGACCGTCATCTCCTCCTTCTTCGAAACCAAGGGCCTTGTCTCGCAGCAGACAGACT
CCTTTGACGAGTTCACGCAGACGACGATCCAGGATCTCGTAAACGAATACTCCACCATCACCCTCGA
CCAGCCCAACCCGCCTTCGCCGCCCGGCCGGACAATAGCCCTTCGTCGATATGAGATCAAGTTTGGA
AGTGTCATGGTGTCACGTCCGACTATCAGTGAGACTGACGGGACCGTGACCTCTCTGCTTCCGTACG
AATGCCGAGATCGCAACTTGACGTACGCCAGTCCGCTCTATATTAAGATTACCAAGAAGCTGTCGGC
CGCCGTCGAGAGGGAGATTCCGCTGCACGAGATGGACGATGCCCAGCAGGAGGAATACGCGAGGA
CCGGCGAGGCCCCTACAAAGCTCGAGTGGGAGGAGGAGGAGGCTGGCGATGACGACCACAATGTG
GGCAAGTCCGATGACTGGAAGGATATGGTCTTTGTGGGCAAGCTGCCCATCATGGTCAAGTCCAAG
ATCTGCCATTTGAGCCGCGAAACCGACGACAGCCTGTTCCTCGTCAACGAGTGTCCCTACGATCAGG
GCGGCTACTTTGTCATCAACGGCAGTGAAAAGGTCCTCATCGCCCAGGAGAGATCTGCCGCCAACAT
TGTCCAAGTTTTCAAAAAGGCCCAGCCCAGTGCTTACACCTACACGGCCGAGATCCGAAGCGCCCTC
GAGAAGGGCTCGCGACTCATCTCTAGCATGATGCTAAAGCTGTACGGCAAGGGAGATTCTGCCCGC
GGAGGCTTCGGTCAGACTATTCACACGACCCTGCCCTTTGTCAAGTCGGATCTTCCCGTCGCCATTGT
CTTCCGCGCCCTGGGTGTCGTTTCCGATGAAGACATTCTGAACCACATTTGCTACGACCGAAACGAC
AGTCAGATGCTGGAAATGCTTCGGCCGTGCATTGAGGAGGCGTTTTGTGTCCAGGACCGAGAGGTC
GCCCTGGATTTCATCGGAAAGCGTGGTAACCGAGACCAAGCCGGCCTCGGCCGTGAGAAGCGTGTC
CGTGTGGCCAAGGACATCCTCCAAAAGGAGACACTGCCTCACATCTCTCAGACGGAAGGTAGCGAG
ACCAGAAAGGCCTTCTTCTTGGGCTACATGGTACATAAGCTGCTGCAGTGCGCACTCGGAAGAAGA
GAGCCCGACGATCGTGACCACTTTGGAAAGAAGCGTCTGGATCTGGCGGGTCCCTTGCTGGCCAAG
CTGTTCCGTGGCATCATGCGAAGAATGAACACGGAGCTGGCCAACTATCTGAGACGGTGTGTGGAG
GGCAACCGACACTTCAATCTCGCGGTTGGTATCAAGCCCGGCACGCTTTCCAACGGCTTGAAGTACT
CGCTCGCCACTGGAAACTGGGGTGATCAGAAGAAGGCCATGAGCTCGACGGCAGGTGTGTCTCAG
GTGCTCAACCGCTACACGTTTGCCTCGACCCTCTCGCATTTGCGCCGCACCAACACGCCCATCGGAA
GAGACGGCAAGCTGGCGAAGCCTCGACAGCTTCACAACACCCATTGGGGCCTGGTCTGCCCGGCCG
AGACACCCGAAGGACAGGCTTGTGGTCTTGTCAAGAACCTGTCTCTGATGTGTTACGTCAGTGTCGG
CTCTCCGTCGGAGCCGTTGATTGAGTTCATGATCAACAGAGGCATGGAAGTCGTCGAGGAATACGA
GCCATTGCGTTATCCTCACGCTACCAAGATCTTCGTCAACGGTGTCTGGGTGGGCGTTCACCAAGAC
CCCAAGCATCTGGTTCAACAGGTTTTGGACACTCGTCGTAAATCTTACCTGCAGTACGAGGTCTCTCT
TGTCCGAGAAATTCGAGACCAGGAGTTCAAAATCTTCTCCGACGCCGGCCGCGTCATGCGACCCGTC
TTCACCGTCCAGCAAGATGACGAATCCGACAATGGCATTCCAAAGGGCCACTTGGTACTGACCAAAG
AACTGGTTAATAAGTTGGCTCAAGAGCAGGCCGAGCCTCCAGAAGACCCAAGCATGAAGATTGGTT
GGGAGGGGCTCATCAGGGCTGGTGCAGTCGAATATCTCGACGCCGAGGAAGAGGAGACGGCCATG
ATTTGCATGACGCCCGAGGATCTCGAACTGTATCGTGCTCAGAAGGCGGGCGTCCAGATGGAAGAG
GATGTTGGCGACGATCCTAACAAGAGACTCAAGACGAAGACGAACCCAACAACGCACATGTACACG

CATTGTGAGATTCATCCAAGCATGATCTTGGGCATTTGTGCAAGCATCATTCCTTTCCCCGATCACAA
CCAGTCCCCCCGTAACACATACCAGTCTGCCATGGGTAAACAAGCCATGGGTTTCTTCCTCACCAACT
ATTCCCGGCGCATGGATACCATGGCCAACATTCTCTACTACCCCCAGAAGCCGCTGGGCACGACCCG
CTCTATGGAGTTTTTGAAATTCCGTGAGCTGCCGGCCGGACAAAACGCCATTGTAGCCATTGCTTGC
TACTCTGGCTACAACCAGGAAGATTCGGTCATCATGAATCAGAGTAGTATCGACAGAGGCCTCTTCC
GAAGTCTCTTCTTCCGATCCTACTCGGATCAGGAGAAGAAGGTGGGCTTGAACTACACCGAAGTGTT
TGAGAAGCCCTTCCACCAAAACACCCTCCGCATGAAGCACGGCACATACGACAAGCTGGACGAGGA
TGGCATCGTCGCTCCTGGTGTCCGAGTGTCCGGCGAGGACATCATCATCGGCAAGACAGCGCCGAT
TGACGCCGACACACAAGATCTCGGCACCAGAACCACCATGCACCAGAGGCGTGATATCTCCACGCCC
CTGCGCAGCACCGAGAACGGCATCGTGGACTCTGTCATCGTGACGGTCAACGCGGACAATGTCAAG
TATGTCAAGGTCCGCGTCCGCACGACCAAGATTCCGCAGATTGGTGACAAGTTTGCCTCTCGTCACG
GACAGAAGGGCACTATTGGTGTTACTTACCGACAAGAGGACATGCCGTTCACCAGAGAGGGAATCA
CGCCAGACATCATCATCAACCCCCACGCCATTCCATCTCGTATGACAATCGCTCACTTGATTGAGTGT
CTCCTAAGCAAGGTCTCAACGCTGGAAGGCATGGAGGGTGATGCCACCCCCTTTACTGATGTCACG
GTCGACTCGGTCTCGGAGCTGCTGCGAAAGCACGGCTACCAGTCCCGAGGCTTCGAGATCATGTAC
AATGGTCACACAGGGCGCAAGCTGAGGGCCCAGGTCTTCTTTGGACCGACATACTACCAGCGACTC
CGCCACATGGTGGACGACAAGATCCACGCCCGTGCCCGTGGTCCGGTGCAGATCATGACACGACAG
CCGGTGGAGGGTCGTGCCCGAGACGGTGGTCTCCGATTCGGAGAAATGGAGCGTGATTGCATGATT
GCACACGGTGCCGCGTCTTTCCTCAAGGAGCGATTGTTTGAGGTGTCTGACGCCTTCCGAGTCCATA
TTTGCGAGATTTGTGGACTCATGACGCCCATTGCCAACCTTTCTAAACAATCGTTCGAGTGCCGGCCG
TGCAAGAACAAGACCAAGATTGCGCAGATTCACATACCTTACGCTGCCAAGCTGTTGTTCCAGGAGC
TCCAGTCGATGAACATTGCAGCTCGAATGTTCACCGACCGGTCCGGCGCGTCTGTCAGGTAA
>Trichoderma gamsii
ATGGCTGATTACGAAGACGATTACGACTATGAGAACTATGGGGATGAAGATGAGGGCATCACGCCC
GAGGATTGCTGGACTGTGATTTCCTCCTTCTTCGAAACCAAGGGTCTTGTATCGCAGCAGACCGACT
CCTTTGACGAGTTCACGCAGACGACAATCCAGGATCTCGTCAACGAATATTCCACCATCACACTCGAT
CAGCCCAATCCTCCTTCGCCACCTGGTCGAACGATAGCCCTTCGCCGATATGAAATCAAATTTGGAA
GTGTCATGGTATCACGTCCCACTATCAGTGAGACGGATGGAACTGTGACATCTCTGCTCCCTTACGA
ATGCCGAGACCGTAACTTGACTTACGCCAGTCCGCTTTACATCAAGATCACCAAGAAGGTGTCTGCG
GCCGTCGAGAGGGAGGTTCCGTTGCACGAGATGGACGATGCCCAGCAAGAACAGTATGCAAGGAC
CGGAGAAAACCCCACAAAGCTGGAATGGGAGGAGGAAGAAAATGGCGAAGACGACAATCTGGGC
AAGTCTGATGACTGGAAGGACATGGTTTTCGTTGGCAAGCTGCCCATCATGGTCAAATCCAAGATTT
GTCATCTGAGCCGTGAACAGGATGACAGCCTGTTCCTCGTCAACGAGTGCCCTTACGACCAAGGAG
GCTACTTTGTTATCAACGGTAGTGAAAAGGTCCTCATCGCCCAAGAGCGGTCCGCCGCAAACATTGT
TCAGGTCTTCAAGAAGGCCCAGCCCAGTGCCTATACCTACACTGCTGAAATCCGAAGTGCGCTGGAA
AAGGGATCTCGACTCATCTCCAGCATGATGCTCAAGTTGTATGGCAAAGGAGACTCTGCGCGAGGT
GGTTTTGGACAAACTATTCACACAACCCTGCCTTTTGTCAAGTCAGATCTTCCCGTTGCCATTGTTTTC
CGTGCCTTGGGCGTCGTTTCTGATGAGGACATTCTGAATCACATCTGCTATGACCGAAACGACAGCC
AAATGCTCGAAATGCTTCGGCCTTGCATTGAAGAAGCCTTTTGTGTTCAGGATCGAGAAGTTGCTCT
TGATTTCATCGGAAAGCGAGGCAATCGTGATCAGGCCGGCCTCGGTCGTGAGAAGCGTGTTCGTGT
AGCAAAGGACATTCTTCAGAAGGAGACGCTTCCCCACATTTCCCAGACTGAAGGCAGTGAGACCAG
AAAGGCATTCTTCCTCGGATACATGGTGCACAAGCTGTTGCAATGCGCGCTCGGAAGACGAGAGCC
CGACGACCGAGATCACTTTGGAAAGAAGCGTCTGGATTTGGCGGGTCCACTGCTGGCCAAGCTGTT
CCGTGGTATCATGCGCAGAATGAATACTGAGCTGGCCAACTACCTGAGACGATGTGTTGAGGGTAA
CCGCCACTTCAACCTTGCTGTTGGCATCAAGCCCGGTACACTTTCCAACGGACTCAAGTACTCACTCG
CCACTGGAAACTGGGGTGACCAGAAGAAGGCAATGAGCTCGACTGCGGGTGTCTCACAGGTGCTTA
ACCGTTACACTTTTGCTTCTACACTTTCCCATTTGCGTCGTACCAATACACCCATCGGAAGAGATGGT

AAGTTGGCGAAGCCTCGACAGCTCCACAACACACACTGGGGTTTGGTGTGCCCGGCCGAGACCCCT
GAAGGACAGGCTTGTGGTCTGGTCAAGAATTTGTCTCTGATGTGTTACGTCAGTGTTGGATCTCCTT
CTGAGCCTTTGATCGAGTTTATGATCAACAGAGGCATGGAAGTCGTTGAGGAGTATGAGCCACTGA
GGTATCCCCATGCCACAAAGATCTTTGTGAATGGTGTCTGGGTTGGAATTCATCAAGACCCAAAGCA
TCTGGTAAACCAAGTCTTGGATACTCGTCGCAAGTCCTATCTGCAGTACGAGGTCTCTCTGATCAGA
GAAATTCGAGACCAAGAATTCAAAATCTTCTCTGATGCCGGTCGTGTTATGCGACCCGTCTTCACTGT
ACAGCAGGAAGATGACCCGGAAACGGGTATCAACAAGGGCCACCTGGTTTTGACCAAGGACCTCGT
CAATAGACTGGCCAAAGAGCAGGCTGAGCCTCCAGAAGACCCAAGCATGAAGCTCGGATGGGAGG
GGCTGATCAGGGCTGGTGCGGTGGAATATCTCGACGCCGAGGAGGAAGAAACGTCCATGATTTGC
ATGACACCGGAAGATCTTGAGCTTTATCGTCTTCAAAAGGCCGGCATTGCCACGGATGAAGACATAG
GAGATGACCCAAATAAGCGTCTCAAGACCAAGACAAATCCGACAACTCACATGTATACGCATTGCGA
GATTCACCCGAGTATGATTCTAGGTATCTGTGCTAGTATCATTCCTTTCCCCGATCACAACCAGTCCCC
CCGTAACACCTATCAATCCGCCATGGGTAAACAAGCCATGGGCTTCTTCTTAACCAACTATTCTCGGC
GTATGGACACCATGGCAAATATCCTTTACTACCCTCAGAAACCGCTTGGCACCACTCGCTCTATGGA
GTTTTTGAAATTCCGTGAGCTGCCAGCCGGACAAAACGCCATTGTAGCGATTGCTTGTTACTCTGGTT
ATAACCAAGAAGATTCCGTCATTATGAACCAGAGTAGTATTGACAGAGGTCTCTTCCGAAGTCTTTTC
TTCCGATCATATTCAGATCAAGAGAAAAAGGTTGGCTTGAATTACACGGAAGTGTTTGAGAAGCCAT
TCCACCAAAACACTCTTCGTATGAAGCACGGCACATATGACAAACTGGATGAAGACGGTATTGTTGC
TCCTGGTGTTCGTGTGTCAGGTGAGGATATCATCATCGGCAAGACGGCACCAATCGACGCCGAGAC
ACAGGATCTTGGAACCAGGACGACCATGCACCAAAGACGCGATATCTCGACGCCTCTGCGAAGTAC
CGAGAACGGCATTGTCGACCAGGTTATCGTGACGGTGAACGCGGATAATGTCAAATATGTCAAGGT
CCGTGTTCGAACGACCAAGATTCCCCAGATTGGAGACAAGTTTGCTTCTCGTCACGGTCAAAAGGGC
ACTATTGGTGTTACTTACCGACAGGAGGATATGCCTTTCTCAAGAGAAGGTCTCACACCAGATATCA
TTATCAACCCCCACGCCATTCCGTCTCGTATGACAATTGCTCACTTGATCGAGTGTCTCCTAAGTAAG
GTCTCTACGTTGGAAGGTATGGAGGGTGATGCTACTCCCTTTACGGATGTCACCGTCGACTCCGTCT
CAGAGCTCTTGCGAAAGCACGGCTACCAGTCTCGAGGCTTCGAGATTATGTACAACGGCCATACTG
GACGCAAGCTCAGGGCACAAGTATTCTTTGGACCAACATACTACCAGCGACTCCGCCACATGGTGGA
CGACAAGATCCATGCCAGAGCTCGTGGTCCTGTGCAGATCATGACTCGGCAGCCAGTGGAGGGTCG
TGCTCGAGATGGTGGTCTCCGATTCGGAGAGATGGAACGTGATTGTATGATTGCACACGGTGCAGC
GTCCTTCCTCAAGGAGCGACTCTTTGAGGTATCGGACGCCTTCCGAGTTCACATTTGCGAGATTTGTG
GACTCATGACGCCCATTGCTAACTTATCCAAACAATCATTCGAGTGTCGACCATGCAAGAACAAGAC
CAAGATTGCACAGATTCACATTCCTTACGCTGCCAAGCTCTTGTTCCAGGAGCTCCAGTCAATGAACA
TTGCAGCTAGAATGTACACCAATCGGTCTGGTGCATCTGTCCGGTAG
>Trichoderma guizhouense
ATGGCTGACTACGAGGACGAATACGACTACGAGAACTACGGAGACGAGGATGAAGGCATCACTCC
CGAGGATTGCTGGACTGTCATCTCCTCCTTCTTCGAAACCAAGGGTCTCGTATCGCAGCAGACCGAC
TCCTTTGACGAATTTACCCAGACGACGATCCAGGACCTCGTAAACGAATACTCCACCATCACACTCGA
CCAGCCAAACCCTCCTTCGCCACCCGGCCGAACAATAGCCCTTCGTCGATATGAGATCAAGTTTGGA
AGCGTCATGGTTTCACGTCCCACTATCAGTGAGACGGACGGAACTGTGACTTCTCTGCTCCCTTACG
AGTGCCGAGACCGTAACTTGACCTACGCCAGTCCCCTTTACATCAAGATTACTAAAAAGGTATCGGC
TGCTGTTGAGAGGGAGGTTCCCCTGCACGAAATGGACGATGCCCAGCAGGAAGAATACGCAAGAA
CCGGCGAACACCCTACAAAGCTTGAGTGGGAAGAGGAGGAGAACGGCGAAGATGACAACATCGGC
AAGTCTGATGACTGGAAGGACATGGTCTTCGTTGGCAAGCTGCCCATCATGGTCAAATCCAAGATTT
GTCATCTGAGCCGTGAACAGGATGATAGCCTGTTCCTTGTCAACGAATGTCCCTACGATCAGGGTGG
CTACTTTGTTATCAACGGCAGTGAAAAGGTTCTCATCGCCCAAGAGAGATCTGCCGCCAACATTGTT
CAAGTTTTCAAAAAGGCCCAGCCCAGTGCCTACACCTATACGGCTGAAATCCGAAGTGCGTTGGAAA
AGGGTTCACGGCTCATCTCCAGCATGATGCTCAAGCTGTATGGCAAGGGAGACTCTGCTCGTGGTG

GCTTTGGCCAAACTATCCACACCACCCTGCCCTTTGTCAAGTCAGATCTTCCCGTGGCCATTGTCTTCC
GTGCCCTGGGTGTCGTTTCTGATGAAGATATCCTCAACCACATTTGCTACGACCGCAACGACAGCCA
GATGCTGGAGATGCTTCGACCTTGTATTGAGGAGGCCTTCTGTGTCCAGGACCGAGAGGTTGCTCTC
GATTTCATCGGAAAGCGTGGAAACCGAGACCAAGCTGGTCTCGGACGCGAGAAGCGTGTCCGCGT
GGCCAAGGATATCCTTCAGAAGGAGACTCTTCCCCACATTTCACAGACAGAGGGAAGTGAAACCAG
AAAGGCATTCTTCCTGGGATACATGGTGCACAAGCTGTTGCAATGTGCGCTCGGAAGAAGAGAGCC
CGATGATCGTGACCACTTTGGAAAGAAGCGTCTGGATCTGGCGGGTCCCCTGCTAGCCAAGCTGTTC
CGTGGTATCATGCGAAGGATGAACACTGAATTGGCCAACTATTTGAGACGTTGCGTTGAGGGCAAC
CGACACTTCAACCTTGCTGTGGGTATCAAGCCCGGCACGCTCTCAAACGGATTGAAGTATTCGCTTG
CCACAGGAAACTGGGGTGATCAGAAGAAGGCCATGAGCTCAACTGCTGGTGTGTCCCAGGTGCTTA
ACCGATACACGTTTGCTTCGACTCTGTCACATTTGCGTCGTACCAACACTCCCATCGGAAGAGATGGT
AAGCTGGCAAAGCCTCGACAGCTTCACAACACGCATTGGGGTTTGGTCTGCCCAGCCGAGACACCC
GAAGGACAGGCCTGTGGTCTGGTCAAAAACTTATCTTTGATGTGTTACGTCAGTGTCGGTTCTCCCTC
TGAGCCTCTGATTGAGTTCATGATCAACAGAGGTATGGAAGTCGTCGAAGAGTACGAGCCGCTGCG
GTATCCTCATGCTACAAAGATTTTTGTGAACGGTGTCTGGGTTGGAGTTCACCAAGACCCTAAGCAC
TTGGTGAACCAGGTTCTGGATACTCGTCGCAAGTCCTATCTGCAGTACGAAGTTTCTCTGGTGAGAG
AAATTCGAGACCAGGAATTCAAAATCTTTTCCGACGCAGGTCGTGTCATGCGACCAGTCTTTACCGTT
CAGCAGGAAGATGACCCGGAAACGGGCATCAACAAGGGCCACCTGGTATTGACCAAGGAACTCGT
CAATAGATTGGCCAAGGAGCAGGCTGAGCCTCCGGAAGACCCCAGCATGAAGATTGGATGGGAGG
GATTGATTCGGGCTGGTGCGGTTGAATACCTCGACGCCGAGGAAGAGGAGACGTCCATGATCTGCA
TGACGCCAGAGGATCTCGAGCTGTATCGTCTTCAGAAGGCCGGTATTAATACCGAGGAAGACATGG
GAGATGACCCGAACAAGCGACTAAAGACCAAGACGAACCCGACAACTCACATGTACACCCATTGCG
AGATTCACCCAAGTATGATCTTAGGTATCTGTGCTAGTATCATTCCTTTCCCCGATCACAACCAGTCCC
CCCGTAACACTTACCAATCTGCCATGGGTAAGCAAGCTATGGGTTTCTTCCTCACGAACTATTCCCGG
CGCATGGACACCATGGCCAACATTCTCTACTACCCCCAGAAGCCGCTGGGTACCACTCGATCCATGG
AATTTTTGAAATTCCGAGAGTTGCCTGCTGGTCAGAACGCCATTGTAGCCATTGCCTGTTACTCTGGT
TACAACCAGGAAGATTCCGTCATTATGAACCAGAGTAGTATTGACAGAGGTCTTTTCCGAAGTCTCT
TCTTCCGATCATACTCGGATCAGGAGAAGAAGGTTGGATTGAACTACACCGAAGTGTTTGAGAAGC
CCTTCCACCAAAATACTCTTCGCATGAAGCACGGTACATATGACAAGCTGGATGAAGACGGTATCGT
TGCTCCTGGTGTCCGTGTATCTGGTGAAGATATCATTATTGGCAAGACGGCGCCGATTGACGCTGAG
ACACAAGATCTTGGTACCAGGACAACTATGCACCAGAGGCGAGATATCTCAACTCCTCTGCGTAGCA
CTGAGAACGGTATCGTTGACTCTGTCATTGTGACAGTGAACGCGGACAATGTCAAGTATGTCAAAGT
CCGTGTCCGAACGACCAAGATTCCCCAGATTGGAGACAAGTTCGCCTCTCGTCACGGTCAAAAGGGC
ACTATTGGTGTTACTTACCGACAAGAGGACATGCCCTTCACGAGAGAAGGTGTCACTCCAGACATCA
TCATCAACCCTCACGCCATTCCATCCCGTATGACAATCGCTCACTTGATTGAGTGTCTCCTAAGTAAG
GTCTCTACGCTGGAAGGTATGGAGGGTGACGCCACCCCCTTTACGGACGTCACGGTCGACTCGGTCT
CAGAGCTGTTGCGAAAGCACGGCTACCAGTCACGAGGCTTTGAGATTATGTACAATGGCCACACAG
GTCGCAAGCTGAGAGCCCAGGTGTTCTTCGGACCAACATACTACCAGCGACTCCGTCACATGGTGG
ACGACAAGATCCACGCTCGTGCCCGTGGTCCCGTGCAGATCATGACTAGACAACCTGTGGAGGGTC
GTGCCAGAGATGGTGGTCTCCGATTCGGAGAAATGGAACGTGATTGTATGATTGCTCACGGTGCCG
CGTCCTTCCTCAAGGAGCGATTGTTTGAGGTGTCAGACGCCTTCCGAGTTCACATTTGCGAGATTTGT
GGACTCATGACGCCTATTGCCAACCTCTCTAAGCAATCGTTCGAGTGTCGACCTTGTAAGAACAAGA
CCAAGATTGCGCAGATTCACATCCCCTATGCTGCCAAGCTCCTGTTCCAGGAACTCCAGTCGATGAA
CATTGCGGCTCGTATGTTCACAAACCGGTCTGGCGCGTCCATCAGGTAA
>Trichoderma hamatum
ATGGCTGATTACGAAGACGAGTACGATTATGAGAACTATGGGGATGAGGATGAGGGCATCACGCC
CGAGGATTGCTGGACTGTGATTTCCTCTTTCTTCGAGACAAAGGGTCTTGTATCGCAGCAGACCGAC

TCCTTTGACGAGTTCACTCAGACGACAATCCAGGATCTCGTTAACGAATACTCCACCATCACCCTCGA
TCAGCCCAATCCTCCTTCGCCACCTGGCCGAACGATAGCCCTTCGCCGATATGAAATCAAATTTGGAA
GTGTCATGGTATCACGCCCCACTATCAGTGAGACGGATGGAACCGTGACATCTTTGCTGCCTTACGA
ATGTCGAGACCGTAACCTGACCTACGCCAGTCCGCTTTACATCAAGATCACCAAAAAGGTGTCTGCG
GCTGTCGAGAGGGAGGTTCCGCTGCACGAGATGGACGATGCCCAGCAAGAACAGTATGCAAGGAC
CGGAGAGCACCCTACAAAGCTGGAATGGGAGGAGGAAGAGAATGGCGAAGACGACAATCTCGGC
AAGTCTGACGACTGGAAGGACATGGTTTTCGTTGGCAAGCTGCCTATCATGGTCAAATCTAAGATTT
GTCATCTGAGCCGTGAACAGGATGATAGCCTGTTCCTTGTTAACGAGTGCCCTTACGACCAAGGTGG
CTACTTTGTTATCAACGGTAGTGAAAAGGTCCTCATTGCCCAAGAGCGCTCCGCCGCAAACATTGTTC
AGGTCTTCAAGAAGGCCCAACCCAGTGCCTATACCTACACAGCCGAAATCCGAAGTGCGCTGGAAA
AGGGATCTCGACTCATCTCTAGCATGATGCTCAAGTTGTATGGCAAAGGAGACTCTGCGCGAGGTG
GCTTTGGCCAGACTATTCACACAACCCTGCCTTTTGTCAAATCAGATCTTCCCGTTGCCATTGTTTTCC
GTGCCTTGGGTGTCGTTTCTGATGAGGACATTCTGAATCACATTTGCTACGACCGAAACGATAGCCA
AATGCTTGAAATGCTCCGACCATGTATTGAAGAAGCCTTTTGCGTCCAGGATCGAGAGGTTGCTCTC
GATTTCATCGGAAAGCGAGGCAATCGTGATCAAGCTGGTCTCGGTCGTGAGAAGCGTGTTCGTGTA
GCAAAGGATATTCTCCAGAAGGAGACGCTTCCCCACATTTCCCAGACTGAGGGCAGTGAGACCAGA
AAGGCATTCTTCCTCGGATACATGGTGCACAAGCTATTGCAGTGCGCACTCGGAAGACGAGAGCCC
GATGACCGTGATCACTTTGGAAAGAAGCGTCTGGATCTGGCTGGTCCACTGCTGGCTAAGCTGTTCC
GTGGTATCATGCGCAGGATGAATACCGAGTTGGCCAACTACCTGAGACGATGTGTTGAGGGTAACC
GCCACTTCAACCTTGCTGTTGGCATCAAGCCCGGCACACTCTCCAACGGATTGAAGTATTCACTTGCC
ACCGGAAACTGGGGTGACCAGAAGAAGGCAATGAGCTCGACTGCAGGTGTATCACAGGTGCTTAA
CCGTTACACTTTTGCCTCGACACTTTCTCATTTGCGTCGTACCAACACACCCATTGGAAGAGATGGTA
AGCTGGCGAAGCCTCGACAGCTTCACAACACACATTGGGGTTTGGTGTGCCCAGCCGAGACCCCCG
AAGGACAAGCTTGTGGTCTGGTCAAAAACTTGTCTCTGATGTGCTACGTCAGTGTCGGATCTCCTTCT
GAGCCTTTGATCGAGTTTATGATCAATAGGGGTATGGAGGTTGTTGAGGAGTACGAACCACTGAGG
TATCCCCATGCTACAAAGATCTTTGTGAATGGTGTCTGGGTTGGAATCCACCAAGACCCCAAGCATC
TGGTAAACCAAGTTTTGGACACTCGTCGTAAATCCTATCTGCAGTACGAAGTCTCTCTGATCAGAGAT
ATTCGTGACCAAGAATTCAAAATCTTCTCTGACGCCGGTCGTGTTATGCGTCCTGTCTTTACTGTACA
GCAAGAAGATGACCCGGAAACGGGTATCAACAAGGGCCACTTGGTATTGACCAAGGACCTCGTCAA
CAGACTTGCCAAAGAGCAGGCTGAGCCTCCAGAAGACCCAAGCATGAAGCTTGGATGGGAGGGGT
TAATTAGGGCTGGTGCGGTGGAATATCTCGACGCCGAGGAAGAAGAAACGGCTATGATTTGCATGA
CACCGGAGGATCTTGAACTTTATCGTCTTCAGAAGGCTGGTATTTCCACGGATGAAGACATGGGAG
ACGATCCAAACAAGCGTCTTAAGACCAAGACAAATCCGACAACTCACATGTACACGCATTGCGAGAT
TCACCCAAGTATGATCTTAGGTATCTGTGCTAGTATCATTCCTTTCCCCGATCACAACCAGTCCCCCG
TAACACCTACCAATCTGCCATGGGTAAACAAGCCATGGGCTTCTTCTTAACCAACTATTCTCGGCGTA
TGGATACCATGGCCAATATCCTCTACTACCCTCAGAAGCCGCTGGGCACCACTCGGTCTATGGAGTT
CTTGAAATTCCGTGAACTGCCAGCCGGACAAAACGCCATTGTAGCAATTGCTTGTTACTCTGGTTATA
ACCAAGAAGATTCCGTCATTATGAACCAGAGTAGTATTGACAGAGGTCTCTTCCGAAGTCTCTTCTTC
CGATCATATTCAGACCAAGAGAAGAAGGTTGGCTTGAACTACACGGAAGTGTTTGAGAAGCCATTC
CACCAAAACACCCTTCGTATGAAGCACGGCACATATGACAAGTTGGATGAAGATGGTATCGTTGCTC
CTGGTGTCCGTGTGTCTGGTGAGGATATCATTATCGGCAAGACAGCGCCAATCGACGCCGAGACAC
AGGATCTTGGAACCAGGACGACCATGCACCAGAGACGCGATATCTCAACGCCTCTGCGAAGCACTG
AGAACGGTATCGTTGACCAGGTCATCGTAACGGTGAACGCGGACAATGTCAAATATGTCAAGGTCC
GTGTTCGAACGACCAAGATTCCCCAGATTGGAGACAAGTTTGCCTCTCGTCACGGTCAAAAGGGCAC
TATTGGTGTTACTTACCGACAGGAGGATATGCCTTTCACAAGAGAAGGTCTCACTCCAGATATCATT
ATCAACCCCCACGCCATTCCGTCTCGTATGACAATTGCTCACTTGATTGAGTGTCTCCTGAGTAAGGT
CTCTACGTTGGAAGGTATGGAGGGTGATGCCACTCCCTTCACGGATGTCACGGTCGACTCAGTCTCG

GAGCTCTTGCGGAAGCACGGCTACCAGTCTCGAGGCTTCGAGATTATGTACAATGGCCATACAGGT
CGCAAGCTAAGAGCACAAGTGTTCTTTGGACCAACATACTACCAGCGACTCCGTCACATGGTGGATG
ACAAGATCCATGCCAGAGCTCGTGGTCCTGTGCAGATCATGACAAGACAGCCGGTGGAGGGTCGTG
CTCGAGATGGTGGTCTCCGATTCGGAGAAATGGAACGTGATTGTATGATTGCTCACGGTGCAGCGT
CCTTCCTCAAGGAGCGACTATTTGAGGTGTCAGATGCTTTCCGAGTTCACATTTGCGAGATTTGTGG
ACTCATGACACCCATTGCTAACTTGTCCAAACAATCATTTGAGTGCCGACCATGCAAGAACAAGACC
AAGATTGCACAGATTCACATTCCTTATGCTGCCAAGCTCTTGTTCCAAGAGCTCCAGTCAATGAACAT
TGCAGCTAGAATGTACACAAACCGATCTGGTGCATCTGTTCGGTAA
>Trichoderma harzianum
ATGGCTGACTACGAGGACGAATACGACTACGAGAACTACGGAGACGAGGATGAAGGCATCACTCC
CGAGGATTGCTGGACTGTCATCTCCTCCTTCTTCGAAACCAAGGGTCTCGTATCGCAGCAGACCGAC
TCTTTTGACGAATTCACCCAGACGACGATCCAGGACCTCGTAAACGAATACTCCACCATCACACTCGA
CCAGCCAAACCCTCCTTCGCCACCCGGCCGAACAATAGCCCTTCGTCGATATGAGATCAAGTTTGGA
AGCGTCATGGTGTCACGTCCCACTATCAGTGAGACGGACGGCACTGTGACTTCTCTGCTCCCTTACG
AGTGCCGAGACCGTAACTTGACCTACGCCAGTCCCCTCTATATCAAGATCACTAAAAAGGTGTCGGC
TGCTGTTGAGAGGGAGGTTCCCCTGCACGAAATGGACGATGCTCAGCAGGAGGAATACGCAAGAA
CCGGCGAACACCCTACAAAGCTCGAGTGGGAAGAGGAGGAGAACGGCGAAGATGACAACATCGGC
AAGTCTGATGACTGGAAGGACATGGTCTTCGTTGGCAAGCTGCCCATCATGGTCAAATCCAAGATTT
GTCATCTGAGCCGTGAACAGGATGATAGCCTGTTCCTTGTCAACGAATGTCCCTACGATCAGGGTGG
CTACTTTGTTATCAACGGCAGTGAAAAGGTCCTCATCGCCCAAGAGAGATCCGCCGCCAACATTGTC
CAAGTCTTCAAGAAGGCCCAGCCCAGTGCCTATACCTATACGGCTGAAATCCGAAGTGCGCTGGAA
AAGGGCTCACGGCTCATCTCTAGCATGATGCTCAAGCTGTATGGCAAGGGAGACTCTGCTCGTGGT
GGCTTTGGCCAGACTATCCACACCACCCTGCCCTTTGTCAAGTCAGATCTTCCCGTCGCCATTGTCTTC
CGTGCCCTGGGTGTCGTTTCTGATGAAGATATCCTCAACCACATTTGCTACGACCGCAACGACAGCC
AGATGCTGGAGATGCTTCGACCTTGTATTGAGGAGGCCTTCTGTGTCCAGGACCGAGAGGTTGCTCT
GGATTTCATCGGAAAGCGTGGAAACCGAGACCAAGCTGGTCTCGGACGCGAGAAGCGTGTCCGCG
TGGCCAAGGATATCCTTCAGAAGGAGACTCTTCCCCACATTTCACAGACAGAGGGAAGTGAAACCA
GAAAGGCATTTTTCTTGGGATACATGGTGCACAAGCTGTTGCAATGTGCGCTCGGAAGAAGAGAGC
CCGACGATCGTGACCACTTTGGAAAGAAGCGTCTGGATCTGGCGGGTCCCCTGCTGGCAAAGCTGT
TCCGTGGTATCATGCGAAGGATGAACACTGAGTTGGCCAACTATCTGAGACGATGCGTCGAGGGCA
ACCGACACTTCAACCTTGCTGTGGGTATCAAGCCCGGCACGCTTTCAAACGGATTGAAGTATTCGCT
TGCCACAGGAAACTGGGGTGATCAGAAGAAGGCCATGAGCTCAACTGCAGGTGTGTCCCAGGTGCT
TAACCGTTACACGTTTGCTTCGACCCTATCACATTTGCGTCGTACCAATACTCCTATCGGAAGAGATG
GTAAGCTCGCAAAGCCTCGACAGCTTCACAACACGCACTGGGGTTTGGTCTGCCCGGCCGAGACAC
CCGAGGGACAGGCTTGTGGTCTGGTCAAGAACTTGTCTTTGATGTGTTACGTCAGTGTCGGTTCTCC
CTCCGAACCTCTGATTGAGTTCATGATCAACAGAGGTATGGAAGTCGTGGAAGAGTACGAGCCGCT
GCGGTATCCTCATGCTACAAAGATTTTTGTGAACGGTGTCTGGGTTGGAGTCCACCAAGACCCTAAG
CACTTGGTGAACCAGGTCCTGGACACTCGTCGCAAGTCCTATCTGCAATACGAAGTCTCTCTCGTGA
GAGAAATTCGAGACCAGGAATTCAAAATCTTTTCCGACGCAGGCCGTGTAATGCGGCCAGTCTTTAC
CGTTCAGCAGGAAGATGACCCGGAAACGGGCATCAACAAGGGCCACCTGGTATTGACCAAGGAGC
TCGTCAATAGATTGGCCAAGGAGCAGGCTGAACCTCCGGAAGACCCCAGCATGAAGATTGGATGGG
AGGGATTGATTAGGGCTGGTGCGGTTGAATATCTCGACGCCGAGGAAGAGGAGACGTCCATGATCT
GCATGACGCCAGAGGATCTCGAGCTGTATCGTCTTCAGAAGGCTGGTATTAACACTGAGGAAGACA
TGGGAGATGACCCGAACAAGCGACTAAAGACCAAGACAAACCCGACTACTCACATGTACACCCATT
GCGAGATTCACCCAAGTATGATCTTAGGCATCTGTGCTAGTATCATTCCTTTCCCCGATCACAACCAG
TCCCCCCGTAACACTTACCAATCTGCCATGGGTAAGCAAGCTATGGGTTTCTTCCTCACAAACTATTC
CCGGCGCATGGACACCATGGCCAACATTCTCTACTACCCCCAGAAGCCGCTGGGTACCACTCGATCC

ATGGAGTTTTTGAAGTTCCGAGAGTTGCCTGCTGGTCAGAACGCCATTGTAGCCATTGCCTGTTACTC
TGGTTACAACCAGGAAGATTCCGTCATTATGAACCAGAGTAGTATTGACAGAGGTCTTTTCCGAAGT
CTCTTCTTCCGATCATACTCGGATCAGGAGAAGAAGGTTGGATTGAACTACACCGAAGTGTTTGAGA
AGCCATTCCACCAAAACACTCTTCGCATGAAGCACGGCACATACGACAAGCTGGATGAAGACGGTA
TCGTTGCTCCTGGTGTCCGAGTATCTGGTGAAGATATCATTATTGGCAAGACGGCGCCGATTGACGC
TGAGACACAAGATCTCGGTACCAGGACGACTATGCACCAGAGGCGAGATATCTCAACTCCTCTGCGT
AGCACTGAGAACGGTATCGTTGACTCTGTCATTGTGACAGTGAACGCGGACAATGTCAAGTATGTCA
AGGTCCGTGTCCGAACGACCAAGATTCCTCAGATTGGAGACAAGTTCGCCTCTCGTCACGGTCAAAA
GGGCACTATTGGTGTTACTTACCGACAAGAGGACATGCCCTTCACAAGAGAAGGTGTCACTCCAGAC
ATCATCATCAACCCTCACGCCATTCCATCCCGTATGACAATCGCTCACTTGATTGAGTGTCTCCTAAGT
AAGGTCTCTACGCTGGAAGGTATGGAGGGTGATGCCACCCCCTTCACGGATGTCACCGTCGACTCG
GTCTCAGAGCTGTTGCGAAAGCACGGCTACCAGTCACGAGGCTTTGAGATTATGTACAATGGCCAC
ACAGGCCGCAAGCTGAGAGCCCAGGTGTTCTTTGGACCAACATACTACCAGCGACTCCGTCACATGG
TGGACGACAAGATCCACGCTCGTGCCCGTGGTCCCGTGCAGATTATGACAAGACAACCCGTGGAGG
GTCGTGCCAGAGATGGTGGTCTCCGATTCGGAGAAATGGAACGTGATTGTATGATTGCTCACGGTG
CCGCGTCCTTCCTCAAGGAGCGATTGTTTGAGGTGTCAGACGCCTTCCGAGTTCACATTTGCGAGAT
TTGTGGACTCATGACGCCTATTGCCAACCTCTCTAAACAATCGTTCGAGTGTCGACCTTGTAAGAACA
AGACCAAGATTGCACAGATTCACATCCCTTATGCCGCCAAGCTCCTGTTCCAGGAACTCCAGTCGAT
GAACATTGCGGCTCGCATGTTCACAAACCGGTCTGGCGCGTCCATCAGGTAA
>Trichoderma parareesei
ATGGCTGATTACGAGGACGACTACGACTACGAAAACTATGGAGACGAGGACGAGGGCATCACTCCT
GAGGACTGCTGGACCGTCATCTCCTCCTTCTTCGAGACCAAGGGCCTTGTCTCGCAGCAGACAGACT
CCTTTGACGAGTTCACGCAGACGACAATCCAGGACCTCGTAAACGAATACTCCACCATCACCCTCGA
CCAGCCCAACCCGCCTTCGCCGCCCGGCCGGACAATAGCCCTTCGTCGATATGAGATCAAGTTTGGA
AGCGTCATGGTGTCACGCCCCACTATCAGTGAGACGGACGGAACCGTCACCTCCCTGCTTCCGTACG
AGTGCCGAGATCGCAACTTGACATACGCCAGTCCGCTTTACATTAAGATTACCAAGAAGCTGTCGGC
CGCCGTCGAGAAGGAGATTCCGTTGCACGAGATGGACGACGCCCAGCAGGAGGAGTACGCAAGGA
CCGGCGAGGCCCCGACAAAACTTGAGTGGGAGGAGGAGGAGGCTGGCGAAGATGACCACAACATT
GGCAAGTCTGAAGACTGGAAGGATATGGTTTTCGTGGGCAAGCTGCCCATCATGGTCAAGTCCAAG
ATCTGTCATTTGAGCCGCGAGACCGACGACAGCCTGTTTCTCGTCAACGAGTGTCCCTACGACCAGG
GCGGCTACTTTGTCATCAATGGCAGTGAAAAAGTTCTCATCGCCCAGGAGAGATCTGCCGCCAACAT
CGTTCAAGTGTTTAAAAAGGCCCAGCCTAGTGCCTACACCTACACGGCGGAGATTCGAAGTGCGCTC
GAGAAGGGCTCGAGGCTCATCTCTAGCATGATGCTCAAGCTCTACGGCAAGGGAGATTCTGCCCGC
GGAGGCTTTGGTCAGACCATTCACACGACCCTGCCCTTTGTCAAGTCGGATCTTCCGGTAGCCATTGT
CTTCCGCGCCTTGGGTGTCGTTTCTGATGAAGACATCCTGAACCATATTTGCTACGACCGAAACGAC
AGTCAGATGCTGGAAATGCTTCGGCCATGCATTGAGGAGGCGTTCTGTGTCCAGGACCGAGAGGTC
GCTCTGGACTTCATCGGAAAGCGTGGTAACCGAGATCAAGCCGGTCTCGGCCGTGAGAAGCGTGTC
CGTGTGGCCAAGGATATCCTCCAAAAGGAGACGCTGCCTCACATTTCCCAGACAGAAGGTAGCGAG
ACCAGAAAGGCCTTCTTCTTGGGCTACATGGTGCACAAACTGCTGCAGTGCGCACTCGGCAGAAGA
GAACCCGACGATCGTGACCACTTTGGAAAGAAGCGGCTGGACCTGGCGGGTCCTCTGCTGGCCAAG
CTGTTCCGTGGCATCATGCGAAGAATGAACACCGAGCTGGCCAACTATCTGAGACGGTGCGTGGAG
GGCAACCGACACTTCAATCTCGCCGTCGGCATCAAGCCCGGCACGCTTTCAAACGGCCTAAAGTACT
CGCTCGCCACTGGAAACTGGGGTGATCAGAAGAAGGCCATGAGCTCGACCGCAGGTGTGTCTCAG
GTGCTCAACCGCTACACGTTTGCCTCGACCCTCTCGCATTTGCGGCGTACCAACACGCCTATCGGAA
GAGATGGCAAGCTGGCGAAGCCTCGACAGCTTCACAACACCCACTGGGGTCTCGTCTGCCCGGCCG
AGACACCCGAAGGACAGGCCTGTGGTCTTGTCAAGAACCTGTCTCTGATGTGTTATGTCAGTGTCGG
CTCTCCGTCAGAGCCGTTGATTGAGTTTATGATCAATAGGGGCATGGAAGTGGTCGAGGAATACGA

GCCACTGCGTTATCCTCATGCTACCAAGATCTTCGTCAACGGTGTCTGGGTGGGCATTCACCAGGAC
CCCAAGCATCTCGTCCAGCAGGTCGTGGACACTCGTCGTAAATCCTACCTGCAGTACGAGGTCTCTC
TCGTCAGAGAAATTCGAGACCAAGAGTTCAAGATCTTCTCGGATGCTGGCCGTGTCATGCGACCCGT
TTTTACCGTCCAGCAAGATGAAGAGTCGGACACTGGCATTCCAAAGGGCCACTTGGTACTGACCAAA
GACCTCGTTAATAAGTTGGCCCAAGAGCAGGCCGAGCCGCCAGAAGACCCAAGCATGAAGATTGGA
TGGGAGGGGCTCATCAGGGCTGGTGCAGTTGAGTATCTCGATGCCGAGGAAGAGGAGACGGCCAT
GATTTGCATGACTCCCGAGGATCTCGAGCTGTATCGTGCCCAGAAGGCAGGCATTGCCACCGAAGA
GGACGTGGGCGACGATCCGAACAAGCGACTCAAGACGAGGACAAACCCAACAACGCACATGTACA
CGCACTGCGAGATTCATCCAAGCATGATCTTGGGTATCTGCGCGAGCATCATTCCTTTCCCCGATCAC
AACCAGTCCCCCCGTAACACCTACCAATCTGCCATGGGTAAACAAGCCATGGGTTTCTTCCTCACCAA
CTATTCCAGGCGCATGGATACCATGGCCAACATTCTCTACTACCCCCAGAAGCCGCTGGGTACGACC
CGCTCTATGGAGTTTTTGAAGTTCCGCGAGCTGCCGGCTGGACAAAACGCCATTGTAGCCATTGCTT
GTTACTCTGGCTACAACCAGGAAGATTCGGTCATCATGAATCAGAGTAGTATTGACAGAGGCCTCTT
CCGAAGTCTCTTCTTCCGATCCTACTCGGATCAGGAGAAGAAGGTGGGCTTGAACTACACCGAAGTG
TTCGAGAAGCCGTTCCACCAAAACACCCTCCGTATGAAGCACGGCACATACGACAAGCTTGACGAG
GACGGCATCGTCGCTCCTGGCGTCCGAGTTTCCGGGGAGGACATCATCATCGGCAAGACTGCGCCG
ATTGACGCCGACACGCAAGATCTCGGCACCAGAACCACAATGCACCAGAGGCGTGATATCTCGACA
CCCCTGCGCAGCACCGAGAACGGCATCGTGGACTCTGTCATTGTGACGGTCAACGCGGACAATGTC
AAGTATGTCAAGGTCCGCGTCCGCACGACCAAGATTCCGCAGATTGGTGACAAGTTTGCGTCTCGTC
ACGGACAAAAGGGCACTATTGGTGTCACTTACCGGCAGGAGGACATGCCGTTCACCAGAGAGGGA
ATTACGCCCGACATCATCATCAACCCCCACGCCATTCCGTCTCGTATGACAATCGCTCACTTGATTGA
GTGTCTCCTAAGCAAGGTCTCTACGTTGGAGGGTATGGAGGGTGATGCTACCCCCTTTACCGATGTC
ACGGTCGACTCGGTTTCGGAGCTGCTGCGAAAGCACGGCTACCAGTCTCGAGGCTTCGAGATCATG
TACAATGGTCACACGGGCCGCAAGCTGAGGGCCCAGGTCTTCTTTGGACCAACATACTACCAGCGAC
TCCGCCACATGGTGGACGACAAGATCCACGCCCGTGCCCGTGGTCCGGTGCAGATCATGACGCGAC
AGCCGGTGGAGGGTCGTGCCCGAGACGGTGGTCTGCGATTCGGAGAAATGGAGCGTGATTGCATG
ATTGCACACGGAGCCGCGTCCTTCCTCAAGGAGCGTTTGTTTGAGGTGTCTGACGCCTTCCGAGTTC
ACATTTGCGAGATTTGTGGACTCATGACGCCCATTGCCAACCTCTCCAAGCAATCGTTCGAGTGCCG
GCCGTGCAAGAACAAGACCAAGATTGCGCAGATTCACATCCCTTATGCGGCCAAGCTCTTGTTCCAG
GAGCTCCAGTCGATGAACATTGCCGCCCGAATGTTTACTGACCGGTCTGGCGCGTCTGTCAGGTAA
>Trichoderma reesei
ATGGCTGATTACGAGGACGACTACGACTACGAAAACTATGGAGACGAGGACGAGGGCATCACCCCC
GAGGACTGCTGGACCGTCATCTCCTCCTTCTTCGAGACCAAGGGCCTCGTCTCGCAGCAGACAGACT
CCTTTGACGAGTTCACGCAGACGACAATCCAGGACCTCGTAAACGAATACTCCACCATCACCCTCGA
CCAGCCCAACCCGCCTTCGCCGCCCGGCCGGACAATAGCCCTTCGTCGATATGAGATCAAGTTTGGA
AGCGTCATGGTGTCACGCCCCACTATCAGTGAGACGGACGGAACCGTCACCTCCCTGCTTCCGTACG
AGTGCCGAGATCGCAACTTGACATACGCCAGTCCGCTGTACATTAAGATTACCAAGAAACTGTCCGC
CGCCGTCGAGAAGGAGATTCCGTTGCACGAGATGGACGACGCCCAGCAGGAAGAGTACGCAAGGA
CCGGCGAGGCCCCTACAAAGCTCGAGTGGGAGGAGGAAGAGGCTGGCGAAGATGACCACAACATT
GGCAAGTCCGAAGACTGGAAGGATATGGTCTTCGTGGGCAAGCTGCCCATCATGGTCAAGTCCAAG
ATCTGTCATTTGAGTCGCGAGACCGACGACAGCCTGTTTCTCGTCAACGAGTGTCCCTACGACCAGG
GCGGCTACTTTGTTATCAATGGCAGTGAAAAGGTCCTCATCGCCCAGGAGAGATCTGCTGCCAACAT
CGTCCAAGTGTTCAAAAAGGCCCAGCCTAGTGCCTACACCTATACGGCGGAGATTCGAAGTGCGCTC
GAAAAGGGCTCGAGGCTCATCTCAAGCATGATGCTCAAGCTGTACGGCAAGGGAGATTCTGCCCGC
GGAGGCTTTGGTCAGACCATTCACACGACCCTGCCCTTTGTCAAGTCGGATCTTCCTGTCGCCATTGT
CTTCCGCGCCCTGGGTGTCGTTTCCGATGAGGACATCCTGAACCATATTTGCTACGACCGAAACGAC
AGTCAGATGCTGGAAATGCTTCGGCCATGCATTGAGGAGGCGTTCTGTGTCCAGGACCGAGAGGTC

GCTCTGGACTTCATCGGAAAGCGTGGTAACCGAGACCAAGCGGGTCTCGGCCGTGAGAAGCGTGTC
CGTGTGGCCAAGGATATCCTCCAAAAGGAGACGCTGCCTCACATTTCCCAGACAGAAGGTAGCGAG
ACCAGAAAGGCCTTCTTCTTGGGCTACATGGTGCACAAACTGCTGCAGTGCGCACTCGGCAGAAGA
GAACCCGACGATCGTGACCACTTTGGAAAGAAGCGGCTGGACCTGGCGGGTCCTCTGCTGGCCAAG
CTGTTCCGTGGCATCATGCGAAGAATGAACACCGAGCTGGCCAATTATCTGAGACGGTGCGTGGAG
GGCAACCGACACTTCAATCTCGCCGTCGGCATCAAGCCCGGCACGCTTTCAAACGGCCTAAAGTACT
CGCTCGCCACTGGAAACTGGGGTGATCAGAAGAAGGCCATGAGCTCGACCGCAGGTGTGTCTCAG
GTGCTCAACCGCTACACGTTTGCCTCGACCCTCTCGCATTTGCGGCGTACCAACACGCCCATCGGAA
GAGATGGCAAGCTGGCGAAGCCTCGACAGCTTCACAACACCCATTGGGGTCTCGTCTGCCCGGCCG
AGACACCCGAAGGACAGGCCTGTGGCCTTGTCAAGAACCTGTCTCTGATGTGTTATGTCAGTGTCGG
CTCTCCGTCAGAGCCCTTGATTGAGTTTATGATCAATAGGGGCATGGAAGTGGTCGAGGAATACGA
GCCACTGCGTTATCCTCACGCGACCAAGATCTTCGTCAACGGTGTCTGGGTGGGCATTCACCAGGAC
CCTAAGCATCTCGTCCAGCAGGTCGTGGACACTCGTCGTAAATCCTACCTGCAGTACGAGGTCTCCC
TCGTCAGAGAAATTCGAGACCAAGAGTTCAAGATCTTCTCGGATGCTGGCCGTGTCATGCGACCCGT
TTTTACCGTCCAGCAAGATGAAGAGTCGGACACTGGCATTCCAAAGGGCCACTTGGTACTGACCAAA
GACCTCGTTAATAAGTTGGCCCAAGAGCAGGCCGAGCCGCCAGAAGACCCAAGCATGAAGATTGGA
TGGGAGGGGCTCATCAGGGCTGGTGCGGTTGAGTATCTCGACGCCGAGGAAGAGGAGACGGCCAT
GATTTGCATGACTCCCGAGGATCTCGAGCTGTATCGTGCCCAGAAGGCAGGCATTGCCACCGAAGA
GGACGTGGGCGACGATCCGAACAAGCGACTCAAGACGAGGACAAACCCAACAACGCACATGTACA
CGCACTGCGAGATTCATCCAAGCATGATCTTGGGTATCTGCGCGAGCATCATTCCTTTCCCCGATCAC
AACCAGTCCCCCCGTAACACCTACCAATCTGCCATGGGTAAACAAGCCATGGGTTTCTTCCTCACCAA
CTATTCCCGGCGCATGGACACCATGGCCAACATTCTCTACTACCCCCAGAAGCCGCTGGGTACGACC
CGCTCTATGGAGTTTTTAAAGTTCCGCGAGCTGCCGGCTGGACAAAACGCCATTGTAGCCATTGCTT
GTTACTCTGGCTACAACCAGGAAGATTCGGTCATCATGAATCAGAGTAGCATCGACAGAGGCCTCTT
CCGAAGTCTCTTCTTCCGATCCTACTCGGATCAGGAGAAGAAGGTGGGCTTGAACTACACCGAAGTG
TTTGAGAAGCCGTTCCACCAGAACACCCTCCGCATGAAGCACGGCACATACGACAAGCTGGACGAG
GACGGCATCGTCGCTCCTGGCGTCCGTGTTTCAGGAGAGGACATCATCATCGGCAAGACTGCGCCG
ATTGACGCCGACACGCAAGATCTCGGCACCAGAACTACAATGCACCAGAGGCGTGATATCTCGACA
CCCCTGCGCAGCACCGAGAACGGCATCGTGGACTCTGTCATCGTGACGGTCAACGCGGACAATGTC
AAGTATGTCAAGGTCCGGGTCCGCACGACCAAGATTCCGCAGATTGGTGACAAGTTTGCGTCTCGTC
ACGGACAAAAGGGCACTATTGGTGTCACTTACCGGCAGGAGGACATGCCGTTCACCAGAGAGGGA
ATCACGCCCGACATCATCATCAACCCCCACGCCATTCCGTCTCGTATGACAATCGCTCACTTGATTGA
GTGTCTCCTAAGCAAGGTCTCTACGTTGGAGGGCATGGAGGGTGATGCGACACCCTTTACTGATGTT
ACGGTCGACTCGGTCTCGGAGCTGCTGCGAAAGCACGGCTACCAGTCCCGAGGCTTCGAGATCATG
TACAATGGTCACACGGGCCGCAAGCTGAGGGCCCAGGTCTTCTTTGGACCAACATACTACCAGCGAC
TTCGCCACATGGTGGACGACAAGATCCACGCTCGTGCCCGAGGTCCGGTGCAGATCATGACGCGAC
AGCCGGTGGAGGGTCGTGCCCGAGACGGTGGTCTCCGATTCGGAGAAATGGAGCGTGATTGCATG
ATTGCACACGGAGCCGCGTCCTTCCTCAAGGAGCGATTGTTTGAGGTGTCTGACGCCTTCCGAGTTC
ACATTTGCGAGATTTGTGGACTCATGACGCCCATTGCCAACCTCTCCAAGCAATCGTTCGAGTGCCG
GCCGTGCAAGAACAAGACCAAGATTGCGCAGATTCACATCCCTTATGCGGCCAAGCTCTTGTTCCAG
GAGCTCCAGTCGATGAACATTGCCGCCCGAATGTTTACTGACCGGTCCGGCGCGTCTGTCAGGTAA
>>Trichoderma virens
ATGGCCGACTACGAGGACGACTACGACTACGAGAACTACGGGGACGAGGATGAAGGAATCACGCC
CGAGGATTGCTGGACCGTCATCTCCTCCTTCTTCGAAACCAAGGGCCTTGTTTCGCAGCAGACTGACT
CCTTCGACGAGTTCACGCAGACGACGATCCAGGATCTGGTAAACGAATACTCCACCATCACACTCGA
CCAGCCAAACCCTCCTTCGCCACCCGGCCGAACAATAGCCCTTCGTCGATATGAGATCAAGTTTGGA
AGCGTCATGGTGTCACGTCCCACTATCAGTGAAACGGACGGAACTGTGACTTCTCTGCTCCCTTACG

AGTGCCGAGACCGCAACTTGACGTATGCCAGTCCGCTCTACATCAAGATCACCAAAAAAGTGTCGGC
CGCCGTCGAGAGGGAGGTTCCCCTCCATGAGATGGACGATGCTCAACAGGAGGAATATGCAAGGA
CCGGCGAACACCCTACAAAGCTTGAGTGGGAAGAGGAGGAAAACGGCGAGGATGACAATGTTGGC
AAGTCTGATGATTGGAAGGACATGGTCTTTGTTGGCAAGTTGCCCATCATGGTCAAATCCAAGATTT
GTCATCTGAGCCGTGAACAGGACGATAGCCTGTTCCTTGTCAACGAATGTCCCTACGATCAGGGTGG
CTACTTTGTTATCAACGGCAGTGAGAAGGTTCTCATCGCCCAAGAGAGATCCGCTGCCAATATCGTT
CAAGTTTTCAAAAAGGCCCAGCCCAGTGCCTATACGTACACGGCTGAAATCCGAAGTGCTTTGGAAA
AGGGCTCACGGCTCATCTCCAGCATGATGCTCAAGCTGTACGGAAAGGGAGACTCTGCTCGTGGTG
GCTTTGGCCAGACTATCCACACCACCCTCCCCTTTGTCAAGTCAGATCTCCCCGTCGCCATTGTCTTCC
GTGCCCTGGGTGTCGTTTCTGATGAAGATATCCTGAACCACATTTGCTACGACCGCAACGACAGCCA
GATGCTGGAGATGCTTCGACCTTGCATTGAGGAGGCTTTCTGTGTCCAGGACCGAGAGGTTGCTCTC
GATTTCATCGGAAAGCGAGGAAACCGAGACCAGGCTGGTCTCGGTCGTGAGAAGCGTGTTCGCGT
GGCCAAGGATATCCTTCAGAAGGAGACGCTTCCTCACATTTCCCAGACGGAAGGCAGTGAAACCAG
AAAGGCATTCTTCCTGGGATACATGGTACACAAGCTGCTGCAATGCGCGCTCGGAAGAAGAGAGCC
TGACGATCGTGACCACTTTGGAAAGAAGCGTCTGGATCTGGCTGGTCCCCTGTTGGCTAAATTGTTC
CGTGGTATCATGCGAAGGATGAACACCGAATTGGCCAACTATCTGAGACGTTGCGTCGAGGGCAAC
CGACACTTCAACCTTGCCGTTGGTATCAAGCCCGGCACACTTTCAAACGGATTGAAGTATTCGCTTGC
CACTGGAAACTGGGGTGATCAGAAGAAGGCCATGAGCTCAACTGCCGGTGTGTCTCAGGTGCTTAA
CCGTTACACGTTTGCTTCGACCCTATCACATTTGCGTCGTACCAACACCCCCATTGGAAGAGATGGTA
AGCTGGCGAAGCCTCGACAGCTTCACAACACGCATTGGGGTTTGGTGTGTCCAGCCGAGACACCCG
AAGGACAGGCTTGTGGTCTGGTCAAAAACTTGTCTCTGATGTGCTACGTCAGTGTCGGTTCTCCCTCC
GAACCACTGATTGAGTTCATGATCAACAGAGGTATGGAAGTCGTCGAAGAGTACGAGCCGCTGCGA
TATCCTCATGCTACCAAGATTTTCGTGAACGGTGTCTGGGTTGGAGTTCACCAAGACCCTAAGCATCT
GGTGAACCAGGTTCTAGACACTCGTCGCAAATCCTATCTGCAGTATGAAGTCTCTCTCGTGAGAGAG
ATTCGAGACCAGGAATTCAAAATCTTTTCCGATGCAGGCCGTGTCATGCGACCTGTATTTACCGTTCA
GCAGGAGGATGACCCTGAAACAGGCATCAACAAGGGCCACCTGGTATTAACTAAGGAGCTCGTCAA
CAGATTGGCTAAGGAGCAAGCTGAACCTCCGGAAGACCCGAGCATGAAGATTGGATGGGAGGGAC
TAATCAGGGCTGGTGCGGTTGAATATCTCGACGCCGAAGAAGAGGAGACGTCGATGATTTGCATGA
CACCAGAGGATCTCGAGCTGTATCGTCTTCAGAAAGCCGGTATCTCTACCGATGAAGACATGGGAG
ATGATCCGAACAAGCGACTAAAGACGAAGACCAACCCAACAACCCACATGTACACACATTGCGAGA
TTCACCCAAGCATGATCTTGGGCATCTGTGCTAGTATCATTCCTTTCCCCGATCATAACCAGTCCCCCC
GTAACACTTACCAATCTGCCATGGGCAAGCAAGCTATGGGTTTCTTCCTCACGAACTATTCCCGGCGC
ATGGACACCATGGCCAATATCCTGTACTACCCCCAGAAGCCGCTGGGTACCACTCGATCCATGGAGT
TTTTGAAATTCCGCGAGTTGCCCGCTGGACAAAACGCTATTGTAGCCATTGCCTGTTACTCTGGCTAC
AACCAGGAAGATTCCGTCATTATGAACCAGAGCAGTATTGACAGAGGTCTCTTCCGAAGTCTCTTCT
TCCGATCGTACTCGGACCAGGAGAAAAAGGTTGGCTTGAACTACACTGAAGTGTTTGAGAAGCCGT
TCCACCAAAACACCCTCCGCATGAAGCACGGAACATATGACAAGCTGGATGAAGACGGTATCGTCG
CCCCTGGTGTCCGAGTATCTGGTGAGGATATCATTATTGGCAAGACGGCGCCGATTGATGCCGAGA
CACAAGATCTCGGTACCCGGACGACTATGCACCAGAGACGAGATATCTCAACCCCTCTGCGTAGTAC
CGAGAACGGCATCGTCGACTCGGTCATTGTGACAGTGAACGCGGACAATGTCAAATACGTCAAGGT
CCGTGTCCGAACGACCAAGATTCCCCAGATTGGAGACAAGTTCGCCTCTCGTCACGGTCAGAAGGG
CACTATTGGTGTTACTTACCGACAGGAGGACATGCCCTTCACAAGAGAAGGTGTGACTCCAGATATT
ATCATCAACCCTCACGCCATTCCATCCCGTATGACAATCGCTCACTTGATTGAGTGTCTCCTAAGTAA
GGTCTCTACGCTGGAGGGTATGGAGGGTGATGCTACTCCCTTTACGGATGTCACGGTCGACTCGGTC
TCGGAACTGTTGCGAAAGCATGGCTACCAGTCACGAGGCTTTGAGATCATGTACAATGGCCATACG
GGCCGCAAGCTGAGAGCCCAGGTGTTCTTCGGACCGACATACTACCAGCGACTCCGTCACATGGTG
GACGACAAGATTCACGCCCGTGCCCGTGGTCCCGTGCAGATCATGACCAGACAGCCCGTGGAGGGT

CGTGCTAGAGATGGTGGTCTTCGATTCGGAGAAATGGAGCGTGATTGTATGATTGCTCACGGTGCT
GCATCCTTCCTCAAGGAGCGATTGTTCGAGGTGTCTGACGCCTTCCGAGTTCACATTTGCGAGATTTG
TGGACTCATGACGCCCATTGCCAACCTATCTAAACAATCGTTCGAGTGTCGACCATGCAAGAACAAG
ACCAAGATTGCGCAGATTCACATCCCTTATGCTGCTAAGCTCTTGTTCCAGGAGCTCCAATCCATGAA
TATTGCAGCCCGCATGTTCACAAACCGGTCTGGCGCGTCTGTCAGGTAA

Appendix S6. *tef1* cds retrieved from the genomes of the 13 species analyzed in the present work

>Trichoderma arundinaceum
ATGGGTAAGGAGGACAAGACTCACATCAACGTGGTCGTTATCACTGGTCACTTGATCTACCAGTGCG
GTGGTATCGACAAGCGTACCATCGAGAAGTTCGAGAAGGAAGCCGCCGAACTCGGCAAGGGTTCCT
TCAAGTATGCGTGGGTTCTTGACAAGCTCAAGGCCGAGCGTGAGCGTGGTATCACCATCGACATTG
CCCTCTGGAAGTTCGAGACTCCCAAGTACTATGTCACCGTCATTGACGCTCCCGGCCACCGTGATTTC
ATCAAGAACATGATCACTGGTACTTCCCAGGCTGACTGCGCTATCCTCATTATCGCTGCCGGTACTGG
TGAGTTCGAGGCTGGTATCTCCAAGGATGGCCAGACCCGTGAGCACGCTCTGCTCGCCTACACCCTG
GGTGTCAAGCAGCTCATCGTCGCCATCAACAAGATGGACACTCCTTGAGGCTATCGACTCCATCGAG
CCCCCCAAGCGTCCCACGGACAAGCCCCTCCGTCTGCCCCTCCAGGACGTGTACAAGATTGGTGGTA
TCGGAACAGTCCCTGTCGGCCGTATCGAGACTGGTGTCCTCAAGCCCGGTATGGTCGTTACCTTCGC
TCCCTCCAACGTCACCACTGAAGTCAAGTCCGTTGAGATGCACCACGAGCAGCTCAGCGAGGGCCA
GCCCGGTGACAACGTTGGTTTCAACGTGAAGAACGTTTCCGTCAAGGAAATCCGCCGTGGCAACGT
TGCCGGTGACTCCAAGAACGACCCCCCCATGGGCGCCGCTTCTTTCACCGCCCAGGTCATCGTCATG
AACCACCCCGGTCAGGTTGGTGCCGGCTATGCCCCCGTCCTCGACTGCCACACTGCCCACATTGCCT
GCAAGTTCGCCGAGCTCCAGGAGAAGATCGACCGCCGTACCGGTAAGGCTACCGAGTCTGCCCCCA
AGTTCATCAAGTCCGGTGACTCTGCCATCGTCAAGATGGTTCCCTCCAAGCCCATGTGCGTTGAGGC
TTTCACCGACTACCCTCCCCTGGGTCGTTTCGCCGTCCGTGACATGCGTCAGACCGTCGCTGTCGGTG
TCATCAAGGCTGTCGACAAGTCCGCTGCCACCAGTGGCAAGGTCACCAAGTCCGCTGCCAAGGCCA
CCAAGAAATAA
>Trichoderma asperellum
ATGGGTAAGGAGGAGAAGACTCACATCAACGTCGTCGTCATCACTGGTCACTTGATCTACCAGTGC
GGTGGTATTGACAAGCGTACCATCGAGAAGTTCGAGAAGGAAGCCGCCGAACTCGGCAAGGGTTC
CTTCAAGTATGCGTGGGTTCTTGACAAGCTCAAGGCCGAGCGTGAGCGTGGTATCACCATCGACATT
GCCCTCTGGAAGTTCGAGACTCCCAAGTACTATGTCACCGTCATTGACGCTCCCGGTCACCGTGATTT
CATCAAGAACATGATCACTGGTACCTCCCAGGCTGACTGCGCTATCCTGATTATCGCTGCCGGTACT
GGTGAGTTCGAGGCTGGTATCTCCAAGGATGGCCAGACCCGTGAGCACGCTCTGCTCGCCTACACC
CTGGGTGTCAAGCAGCTCATCGTTGCCATCAACAAGATGGACACTCCTCGAGGCCATCGACGCCATT
GAGCCCCCCAAGCGTCCCACAGACAAGCCCCTCCGTCTGCCCCTCCAGGACGTCTACAAGATCGGTG
GTATCGGAACAGTCCCTGTCGGCCGTATCGAGACTGGTGTCCTCAAGCCCGGTATGGTCGTCACCTT
CGCTCCCTCCAACGTCACCACTGAAGTCAAGTCCGTCGAGATGCACCACGAGCAGCTCGCTGAGGGT
GTCCCCGGTGACAACGTTGGATTCAACGTCAAGAACGTCTCTGTCAAGGATATCCGCCGTGGTAACG
TTGCCGGTGACTCCAAGAACGACCCTCCCATGGGTGCCGCTTCTTTCAACGCCCAGGTCATTGTCATG
AACCACCCTGGCCAGGTCGGTGCCGGTTACGCTCCCGTCCTCGATTGCCACACTGCCCACATTGCCT
GCAAGTTCTCTGAGCTCCTCGAGAAGATCGACCGCCGTACCGGTAAGGCTACTGAGGCCTCCCCCAA
GTTCATCAAGTCTGGTGACTCCGCCATCGTCAAGATGGTTCCCTCCAAGCCCATGTGCGTTGAGGCTT
TCACCGACTACCCTCCCCTGGGTCGTTTCGCCGTCCGTGACATGCGTCAGACCGTCGCCGTCGGTGTC
ATCAAGTCCGTCGAGAAGTCCACTGGTGCTGCCGGCAAGGTCACCAAGTCCGCCGCCAAGGCCGCC
AAGAAATAA
>Trichoderma atrobrunneum
ATGGGTAAGGAGGACAAGACTCACATCAACGTGGTCGTTATCACCGGTCACTTGATCTACCAGTGC
GGTGGTATCGACCGTCGTACCATCGAGAAGTTCGAGAAGGAAGCCGCCGAACTCGGCAAGGGTTCC
TTCAAGTACGCTTGGGTTCTTGACAAGCTCAAGGCCGAGCGTGAGCGTGGTATCACCATCGACATTG
CTCTGTGGAAGTTCGAGACTCCCAAGTACTATGTCACCGTCATTGACGCTCCCGGCCACCGTGATTTC
ATCAAGAACATGATCACTGGTACTTCCCAGGCCGATTGCGCTATCCTCATCATTGCCGCCGGTACTG

GTGAGTTCGAGGCTGGTATCTCCAAGGATGGCCAGACCCGTGAGCACGCTCTGCTCGCCTACACCCT
GGGTGTCAAGCAGCTCATCGTTGCCATCAACAAGATGGACACTCCTTGAGGCTATCGACTCCATCGA
GCCCCCCAAGCGTCCCACGGACAAGCCCCTCCGTCTTCCCCTCCAGGATGTCTACAAGATCGGTGGT
ATCGGAACAGTTCCCGTCGGCCGTATCGAGACTGGTATCCTCAAGCCCGGTATGGTCGTCACCTTCG
CTCCCTCCAACGTCACCACTGAAGTCAAGTCCGTCGAGATGCACCACGAGCAGCTCGTCGAGGGTGT
TCCCGGTGACAACGTTGGTTTCAACGTCAAGAACGTTTCCGTTAAGGAAATTCGCCGTGGTAACGTT
GCCGGTGACTCCAAGAACGACCCCCCCATGGGTGCCGCTTCTTTCACCGCTCAGGTCATCGTCATGA
ACCACCCTGGCCAGGTCGGTGCCGGCTACGCCCCCGTTCTTGACTGCCACACTGCCCACATTGCCTG
CAAGTTCGCCGAGCTCCAGGAGAAGATCGACCGCCGTACCGGTAAGGCTACCGAGACTGCCCCCAA
GTTCATCAAGTCCGGTGACTCTGCCATCGTCAAGATGATTCCCTCCAAGCCCATGTGCGTTGAGGCTT
TCACCGACTACCCTCCCCTGGGTCGTTTCGCCGTCCGTGACATGCGACAGACCGTCGCTGTCGGTGT
CATCAAGGCTGTCGACAAGTCCGCTGCCACCGCTGGCAAGGTCACCAAGTCCGCTGCCAAGGCCAC
CAAGAAATAA
>Trichoderma atroviride
ATGGGTAAGGAGGAGAAGACTCACATCAACGTCGTCGTCATCACTGGTCACTTGATCTACCAGTGC
GGTGGTATTGACAAGCGTACCATCGAGAAGTTCGAGAAGGAAGCCGCCGAGCTCGGCAAGGGTTC
TTTCAAGTATGCGTGGGTTCTTGACAAGCTCAAGGCCGAGCGTGAGCGTGGTATCACCATCGACATT
GCCCTCTGGAAGTTCGAGACTCCCAAGTACTATGTCACCGTCATTGACGCTCCCGGTCACCGTGATTT
CATCAAGAACATGATCACTGGTACTTCCCAGGCTGACTGCGCTATCCTGATTATCGCTGCCGGTACTG
GTGAGTTCGAGGCTGGTATCTCCAAGGATGGCCAGACCCGTGAGCACGCTCTGCTCGCCTACACCCT
GGGTGTCAAGCAGCTCATCGTTGCCATCAACAAGATGGACACTTCTCGAGGCCATTGACGCCATTGA
GCCCCCCAAGCGTCCCACAGACAAGCCCCTCCGTCTTCCCCTTCAGGATGTTTACAAGATCGGTGGT
ATTGGAACAGTCCCTGTCGGCCGTATCGAGACTGGTATCCTCAAGCCCGGTATGGTCGTTACCTTCG
CTCCCTCCAACGTCACCACTGAAGTCAAGTCCGTTGAGATGCACCACGAGCAGCTCGTCGAGGGTGT
CCCCGGTGACAACGTTGGATTCAACGTCAAGAACGTCTCCGTCAAGGATATCCGCCGTGGTAACGTT
GCCGGTGACTCCAAGAACGACCCCCCCATGGGTGCCGCTTCTTTCAACGCCCAGGTCATCGTCATGA
ACCACCCTGGCCAGGTCGGTGCCGGATACGCTCCCGTCCTCGATTGCCACACTGCCCACATTGCCTG
CAAGTTCTCTGAGCTCCTCGAGAAGATCGACCGCCGTACCGGTAAGGCTACTGAGGCCTCCCCCAAG
TTCATCAAGTCTGGTGACTCCGCCATCGTCAAGATGGTTCCCTCCAAGCCCATGTGCGTTGAGGCCTT
CACCGACTACCCTCCCCTGGGTCGTTTCGCCGTCCGTGACATGCGCCAGACCGTCGCCGTCGGTGTC
ATCAAGTCCGTCGAGAAGTCCACTGGTGCTACCGGCAAGGTCACCAAGTCCGCCGCCAAGGCCGCC
AAGAAATAA
>Trichoderma sp. T154
ATGGGTAAGGAGGAAAAGACTCACATCAACGTGGTCGTTATCACCGGTCACTTGATCTACCAGTGC
GGTGGTATCGACCGTCGTACCATCGAGAAGGAAGCCGCCGAACTCGGCAAGGGTTCCTTCAAGTAC
GCTTGGGTTCTTGACAAGCTCAAGGCCGAGCGTGAGCGTGGTATCACCATTGACATTGCTCTGTGGA
AGTTCGAGACTCCCAAGTACTATGTCACCGTCATTGACGCTCCCGGCCACCGTGATTTCATCAAGAAC
ATGATCACTGGTACTTCCCAGGCCGATTGCGCTATCCTCATCATTGCCGCCGGTACTGGTGAGTTCGA
GGCTGGTATCTCCAAGGATGGCCAGACCCGTGAGCACGCTCTGCTCGCCTACACCCTGGGTGTTAAG
CAGCTCATCGTTGCCATCAACAAGATGGACACTCCTTGAGGCCATCGACTCCATCGAGCCCCCCAAG
CGTCCCACGGACAAGCCCCTCCGTCTTCCCCTCCAGGATGTCTACAAGATCGGTGGTATTGGAACAG
TTCCCGTCGGCCGTATCGAGACTGGTGTCCTCAAGCCCGGTATGGTTGTCACTTTCGCTCCCTCCAAC
GTCACCACTGAAGTCAAGTCCGTCGAGATGCACCACGAGCAGCTCGTCGAGGGTGTTCCCGGTGAC
AACGTTGGTTTCAACGTCAAGAACGTTTCCGTTAAGGAAATTCGCCGTGGTAACGTTGCCGGTGACT
CCAAGAACGACCCCCCCATGGGTGCCGCTTCTTTCACCGCTCAGGTCATCGTCATGAACCACCCTGG
CCAGGTCGGTGCCGGCTACGCCCCCGTTCTTGACTGCCACACTGCCCACATTGCCTGCAAGTTCGCC
GAGCTCCAGGAGAAGATCGACCGCCGTACCGGTAAGGCTACCGAGACTGCCCCCAAGTTCATCAAG

TCCGGTGACTCTGCCATCGTCAAGATGATTCCCTCCAAGCCCATGTGCGTTGAGGCTTTCACCGACTA
CCCTCCCCTGGGTCGTTTCGCCGTCCGTGACATGCGACAGACCGTCGCTGTCGGTGTCATCAAGGCC
GTCGACAAGTCCGCTGCCACCGCTGGCAAGGTCACCAAGTCCGCTGCCAAGGCCACCAAGAAATAA
>Trichoderma citrinoviride
ATGGGTAAGGAGGACAAGACTCACATCAACGTGGTCGTTATCACTGGTCACTTGATCTACCAGTGCG
GTGGTATCGACAAGCGTACCATTGAGAAGTTCGAGAAGGAAGCCGCCGAACTCGGCAAGGGTTCCT
TCAAGTATGCGTGGGTTCTTGACAAGCTCAAGGCCGAGCGTGAGCGTGGCATCACCATCGACATTG
CCCTCTGGAAGTTCGAGACTCCCAAGTACTATGTCACCGTCATTGACGCTCCCGGCCACCGTGACTTC
ATCAAGAACATGATCACTGGTACTTCCCAGGCCGACTGCGCTATTCTCATCATTGCCGCCGGTACTG
GTGAGTTCGAGGCTGGTATCTCCAAGGATGGCCAGACCCGTGAGCACGCTCTGCTCGCCTACACCCT
GGGTGTCAAGCAGCTCATCGTCGCCATCAACAAGATGGACACTCCTTGAGGCCATCGACTCCATCGA
GCCCCCCAAGCGTCCCACGGACAAGCCCCTCCGTCTGCCCCTTCAGGACGTCTACAAGATCGGTGGT
ATCGGAACAGTTCCCGTCGGCCGTATCGAGACTGGTGTCCTCAAGCCCGGTATGGTCGTTACCTTCG
CTCCCTCCAACGTCACCACTGAAGTCAAGTCCGTCGAGATGCACCACGAGCAGCTCGCTGAGGGCCA
GCCCGGTGACAACGTTGGTTTCAACGTGAAGAACGTTTCCGTCAAGGAAATCCGCCGTGGCAACGT
TGCCGGTGACTCCAAGAACGACCCCCCCATGGGCGCCGCTTCTTTCACCGCTCAGGTCATCGTCATG
AACCACCCCGGCCAGGTCGGTGCCGGCTACGCCCCCGTCCTCGACTGCCACACTGCCCACATTGCCT
GCAAGTTCGCCGAGCTCCTCGAGAAGATCGACCGCCGTACCGGTAAGGCCACCGAGACTGCCCCCA
AGTTCATCAAGTCCGGTGACTCCGCCATCGTCAAGATGATTCCCTCCAAGCCCATGTGCGTTGAGGC
TTTCACCGACTACCCTCCCCTGGGTCGTTTCGCCGTCCGTGACATGCGCCAGACCGTCGCTGTCGGTG
TCATCAAGGCCGTTGAGAAGTCCACCGCCGCCGCCGGCAAGGTCACCAAGTCCGCTGCCAAGGCCG
CCAAGAAATAA
>Trichoderma gamsii
ATGGGTAAGGAGGAGAAGACTCACATCAACGTCGTCGTCATCACTGGTCACTTGATCTACCAGTGC
GGTGGTATTGACAAGCGTACCATCGAGAAGTTCGAGAAGGAAGCCGCCGAACTCGGCAAGGGTTCT
TTCAAGTATGCGTGGGTTCTTGACAAGCTCAAGGCCGAGCGTGAGCGTGGTATCACCATCGACATTG
CCCTCTGGAAGTTCGAGACTCCCAAGTACTATGTCACCGTCATTGACGCTCCCGGCCACCGTGATTTC
ATCAAGAACATGATCACTGGTACCTCCCAGGCTGACTGCGCTATCCTGATTATCGCTGCCGGTACTG
GTGAGTTCGAGGCTGGTATCTCCAAGGATGGCCAGACCCGTGAGCACGCTCTTCTCGCCTACACCCT
GGGTGTCAAGCAGCTCATCGTTGCCATCAACAAGATGGACACTTCTCGAGGCCATTGACGCCATTGA
GCCCCCCAAGCGTCCCACAGACAAGCCCCTTCGTCTGCCCCTTCAGGATGTTTACAAGATCGGTGGT
ATCGGAACAGTCCCTGTCGGCCGTATCGAGACTGGTATCCTCAAGCCCGGTATGGTCGTTACCTTCG
CTCCCTCCAACGTCACCACTGAAGTCAAGTCCGTTGAGATGCACCACGAGCAGCTCGTTGAGGGTGT
CCCCGGTGACAACGTTGGTTTCAACGTCAAGAACGTCTCCGTCAAGGATATCCGCCGTGGTAACGTT
GCCGGTGACTCCAAGAACGACCCCCCCATGGGTGCCGCTTCTTTCAACGCCCAGGTCATCGTCATGA
ACCACCCTGGCCAGGTCGGTGCCGGATACGCTCCCGTCCTCGATTGCCACACCGCCCACATTGCCTG
CAAGTTCTCTGAGCTCCTCGAGAAGATCGACCGCCGTACCGGTAAGGCTACTGAGGCCTCCCCCAAG
TTCATCAAGTCTGGTGACTCCGCCATCGTCAAGATGGTTCCCTCCAAGCCCATGTGCGTTGAGGCTTT
CACCGACTACCCTCCCCTGGGTCGTTTCGCCGTCCGTGACATGCGCCAGACCGTCGCCGTCGGTGTC
ATCAAGTCCGTCGAGAAGTCCACTGGTGCTACCGGCAAGGTCACCAAGTCCGCCGCCAAGGCCGCC
AAGAAATAA
>Trichoderma guizhouense
ATGGGTAAGGAGGACAAGACTCACATCAACGTGGTCGTTATCACCGGTCACTTGATCTACCAGTGC
GGTGGTATCGACCGTCGTACCATCGAGAAGTTCGAGAAGGAAGCCGCCGAACTCGGCAAGGGTTCC
TTCAAGTACGCTTGGGTTCTTGACAAGCTCAAGGCCGAGCGTGAGCGTGGTATCACCATCGACATTG
CTCTGTGGAAGTTCGAGACTCCCAAGTACTATGTCACCGTCATTGACGCTCCCGGCCACCGTGATTTC
ATCAAGAACATGATCACTGGTACTTCCCAGGCCGATTGCGCTATCCTCATCATTGCCGCCGGTACTG

GTGAGTTCGAGGCTGGTATCTCCAAGGATGGCCAGACCCGTGAGCACGCTCTGCTCGCCTACACCCT
GGGTGTCAAGCAGCTCATCGTTGCCATCAACAAGATGGACACTCCTTGAGGCCATCGACTCCATCGA
GCCCCCCAAGCGTCCCACGGACAAGCCCCTCCGTCTTCCCCTCCAGGATGTCTACAAGATCGGTGGT
ATTGGAACAGTTCCTGTCGGCCGTATCGAGACTGGTATCCTCAAGCCCGGTATGGTCGTCACCTTCG
CTCCCTCCAACGTCACCACTGAAGTCAAGTCCGTCGAGATGCACCACGAGCAGCTCGTCGAGGGTGT
TCCCGGTGACAACGTTGGTTTCAACGTCAAGAACGTTTCCGTTAAGGAAATTCGCCGTGGTAACGTT
GCCGGTGACTCCAAGAACGACCCCCCCATGGGTGCCGCTTCTTTCACCGCTCAGGTCATCGTCATGA
ACCACCCTGGCCAGGTCGGTGCCGGCTACGCCCCCGTTCTTGACTGCCACACTGCCCACATTGCCTG
CAAGTTCGCCGAGCTCCAGGAGAAGATCGACCGCCGTACCGGTAAGGCTACCGAGACTGCCCCCAA
GTTCATCAAGTCCGGTGACTCTGCCATCGTCAAGATGATTCCCTCCAAGCCCATGTGCGTTGAGGCTT
TCACCGACTACCCTCCCCTGGGTCGTTTCGCCGTCCGTGACATGCGACAGACCGTCGCTGTCGGTGT
CATCAAGGCTGTCGACAAGTCCGCTGCCACCGCTGGCAAGGTCACCAAGTCCGCTGCCAAGGCCAC
CAAGAAATAA
>Trichoderma hamatum
ATGGGTAAGGAGGAGAAGACTCACATCAACGTCGTCGTTATCACTGGTCACTTGATCTACCAGTGCG
GTGGTATTGACAAGCGTACCATCGAGAAGTTCGAGAAGGAAGCCGCCGAACTCGGCAAGGGTTCCT
TCAAGTATGCGTGGGTTCTTGACAAGCTCAAGGCCGAGCGTGAGCGTGGTATCACCATCGACATTG
CCCTGTGGAAGTTCGAGACTCCCAAGTACTATGTCACCGTCATTGACGCTCCCGGTCACCGTGATTTC
ATCAAGAACATGATCACTGGTACCTCCCAGGCCGATTGCGCTATCCTCATTATCGCTGCCGGTACTG
GTGAGTTCGAGGCTGGTATCTCCAAGGATGGCCAGACCCGTGAGCACGCTCTGCTCGCCTACACCCT
GGGTGTCAAGCAGCTCATTGTTGCCATCAACAAGATGGACACTCCTCGAGGCCATTGACGCCATCGA
GCCCCCCAAGCGTCCCACAGACAAGCCCCTCCGTCTGCCCCTTCAGGATGTCTACAAGATCGGTGGT
ATCGGAACAGTCCCTGTCGGCCGTATCGAGACTGGTATCCTCAAGCCCGGTATGGTCGTTACCTTCG
CTCCTTCCAACGTCACCACTGAAGTCAAGTCCGTCGAGATGCACCACGAGCAGCTCGTTGAGGGTGT
CCCCGGTGACAACGTTGGATTCAACGTCAAGAACGTCTCCGTCAAGGATATCCGCCGTGGTAACGTT
GCCGGTGACTCCAAGAACGACCCCCCCATGGGTGCCGCTTCTTTCAACGCCCAGGTCATCGTCATGA
ACCACCCTGGCCAGGTCGGTGCCGGATACGCTCCCGTCCTCGATTGCCACACTGCCCACATTGCCTG
CAAGTTCTCTGAGCTCCTCGAGAAGATCGACCGCCGTACCGGTAAGGCTACTGAGGCCTCCCCCAAG
TTCATCAAGTCTGGTGACTCCGCCATCGTCAAGATGGTTCCCTCCAAGCCCATGTGCGTTGAGGCTTT
CACCGACTACCCTCCCCTGGGTCGTTTCGCCGTCCGTGACATGCGACAGACCGTCGCCGTCGGTGTC
ATCAAGTCCGTCGAGAAGTCCACTGGTGCTGCCGGTAAGGTCACCAAGTCCGCCGCCAAGGCCGCC
AAGAAATAA
>Trichoderma harzianum
ATGGGTAAGGAGGACAAGACTCACATCAACGTGGTCGTTATCACCGGTCACTTGATCTACCAGTGC
GGTGGTATCGACCGTCGTACCATCGAGAAGTTCGAGAAGGAAGCCGCCGAACTCGGCAAGGGTTCC
TTCAAGTACGCTTGGGTTCTTGACAAGCTCAAGGCCGAGCGTGAGCGTGGTATCACCATCGACATTG
CTCTGTGGAAGTTCGAGACTCCCAAGTACTATGTCACCGTCATTGACGCTCCCGGCCACCGTGATTTC
ATCAAGAACATGATCACTGGTACTTCCCAGGCCGATTGCGCTATCCTCATCATTGCCGCCGGTACTG
GTGAGTTCGAGGCTGGTATCTCCAAGGATGGCCAGACCCGTGAGCACGCTCTGCTCGCCTACACCCT
GGGTGTCAAGCAGCTCATTGTTGCCATCAACAAGATGGACACTCCTTGAGGCTATCGACTCCATCGA
GCCCCCCAAGCGTCCCACGGACAAGCCCCTCCGTCTTCCCCTCCAGGATGTCTACAAGATCGGTGGT
ATTGGAACAGTTCCCGTCGGCCGTATCGAGACTGGTATCCTCAAGCCCGGTATGGTCGTCACCTTCG
CTCCCTCCAACGTCACCACTGAAGTCAAGTCCGTCGAGATGCACCACGAGCAGCTCGTCGAGGGTGT
TCCCGGTGACAACGTTGGTTTCAACGTCAAGAACGTTTCCGTTAAGGAAATTCGCCGTGGTAACGTT
GCCGGTGACTCCAAGAACGACCCCCCCATGGGTGCCGCTTCTTTCACCGCTCAGGTCATCGTCATGA
ACCACCCTGGCCAGGTCGGTGCCGGCTACGCCCCCGTTCTTGACTGCCACACTGCCCACATTGCCTG
CAAGTTCGCCGAGCTCCAGGAGAAGATCGACCGCCGTACCGGTAAGGCTACCGAGACTGCCCCCAA

GTTCATCAAGTCCGGTGACTCTGCCATCGTCAAGATGATTCCCTCCAAGCCCATGTGCGTTGAGGCTT
TCACCGACTACCCTCCCCTGGGTCGTTTCGCCGTCCGTGACATGCGACAGACCGTCGCTGTCGGTGT
CATCAAGGCTGTCGACAAGTCCGCCGCCACCGCTGGCAAGGTCACCAAGTCCGCTGCCAAGGCCAC
CAAGAAATAA
>Trichoderma parareesei
ATGGGTAAGGAGGACAAGACTCACATCAACGTGGTCGTTATCACTGGTCACTTGATCTACCAGTGCG
GTGGTATCGACAAGCGTACCATTGAGAAGTTCGAGAAGGAAGCTGCCGAACTCGGCAAGGGTTCCT
TCAAGTACGCGTGGGTTCTTGACAAGCTCAAGGCCGAGCGTGAGCGTGGTATCACCATCGACATTG
CCCTCTGGAAGTTCGAGACTCCCAAGTACTATGTCACCGTCATTGACGCTCCCGGCCACCGTGACTTC
ATCAAGAACATGATCACTGGTACTTCCCAGGCCGACTGCGCTATCCTCATTATCGCTGCCGGTACTG
GTGAGTTCGAGGCTGGTATCTCCAAGGATGGCCAGACCCGTGAGCACGCTCTGCTCGCCTACACCCT
GGGTGTCAAGCAGCTCATCGTCGCCATCAACAAGATGGACACTCCTTGAGGCCATCGACTCCATCGA
GCCCCCCAAGCGTCCCACGGACAAGCCCCTGCGTCTGCCCCTCCAGGACGTCTACAAGATCGGTGGT
ATCGGAACAGTTCCCGTCGGCCGTATCGAGACTGGTGTCCTCAAGCCCGGCATGGTCGTTACCTTCG
CCCCCTCCAACGTCACCACTGAAGTCAAGTCCGTCGAGATGCACCACGAGCAGCTCGCTGAGGGTCA
GCCCGGTGACAACGTTGGTTTCAACGTGAAGAACGTCTCCGTCAAGGAAATCCGCCGTGGTAACGT
TGCCGGTGACTCCAAGAACGACCCCCCCATGGGCGCCGCTTCTTTCACCGCCCAGGTCATCGTCATG
AACCACCCCGGCCAGGTCGGTGCCGGCTACGCCCCCGTCCTCGACTGCCACACTGCCCACATTGCCT
GCAAGTTCGCCGAGCTCCTCGAGAAGATCGACCGCCGTACCGGTAAGGCTACCGAGACTGCCCCCA
AGTTCATCAAGTCCGGTGACTCCGCCATCGTCAAGATGATCCCCTCCAAGCCCATGTGCGTTGAGGC
TTTCACCGACTACCCTCCCCTGGGTCGTTTCGCCGTCCGTGACATGCGCCAGACCGTCGCTGTCGGTG
TCATCAAGGCCGTTGAGAAGTCCTCTGCCGCCGCCGGCAAGGTCACCAAGTCCGCTGCCAAGGCTG
CCAAGAAATAA
>Trichoderma reesei
ATGGGTAAGGAGGACAAGACTCACATCAACGTGGTCGTCATCACTGGTCACTTGATCTACCAGTGC
GGTGGTATCGACAAGCGTACCATTGAGAAGTTCGAGAAGGAAGCCGCCGAACTCGGCAAGGGTTC
CTTCAAGTACGCGTGGGTTCTTGACAAGCTCAAGGCCGAGCGTGAGCGTGGTATCACCATCGACATT
GCCCTCTGGAAGTTCGAGACTCCCAAGTACTATGTCACCGTCATTGACGCTCCCGGCCACCGTGACTT
CATCAAGAACATGATCACTGGTACTTCCCAGGCCGACTGCGCTATCCTCATCATCGCTGCCGGTACTG
GTGAGTTCGAGGCTGGTATCTCCAAGGATGGCCAGACCCGTGAGCACGCTCTGCTCGCCTACACCCT
GGGTGTCAAGCAGCTCATCGTCGCCATCAACAAGATGGACACTCCTTGAGGCCATCGACTCCATCGA
GCCCCCCAAGCGTCCCACGGACAAGCCCCTGCGTCTTCCCCTCCAGGACGTCTACAAGATCGGTGGT
ATCGGAACAGTTCCCGTCGGCCGTATCGAGACTGGTGTCCTCAAGCCCGGTATGGTCGTTACCTTCG
CTCCCTCCAACGTCACCACTGAAGTCAAGTCCGTCGAGATGCACCACGAGCAGCTCGCTGAGGGCCA
GCCTGGTGACAACGTTGGTTTCAACGTGAAGAACGTTTCCGTCAAGGAAATCCGCCGTGGCAACGTT
GCCGGTGACTCCAAGAACGACCCCCCCATGGGCGCCGCTTCTTTCACCGCCCAGGTCATCGTCATGA
ACCACCCCGGCCAGGTCGGTGCCGGCTACGCCCCCGTCCTCGACTGCCACACTGCCCACATTGCCTG
CAAGTTCGCCGAGCTCCTCGAGAAGATCGACCGCCGTACCGGTAAGGCTACCGAGTCTGCCCCCAA
GTTCATCAAGTCTGGTGACTCCGCCATCGTCAAGATGATCCCCTCCAAGCCCATGTGCGTTGAGGCTT
TCACCGACTACCCTCCCCTGGGTCGTTTCGCCGTCCGTGACATGCGCCAGACCGTCGCTGTCGGTGTC
ATCAAGGCCGTCGAGAAGTCCTCTGCCGCCGCCGGCAAGGTCACCAAGTCCGCTGCCAAGGCCGCC
AAGAAATAA
>Trichoderma virens
ATGGGTAAGGAGGAGAAGACTCACATCAACGTGGTCGTCATCACTGGTCACTTGATCTACCAGTGC
GGTGGTATCGACAAGCGTACCATCGAGAAGTTCGAGAAGGAAGCCGCCGAACTCGGCAAGGGTTC
CTTCAAGTACGCTTGGGTTCTTGACAAGCTCAAGGCCGAGCGTGAGCGTGGTATCACCATCGACATT
GCCCTGTGGAAGTTCGAGACTCCCAAGTACTATGTCACCGTCATTGACGCCCCCGGCCACCGTGATT

TCATCAAGAACATGATCACTGGTACTTCCCAGGCCGATTGCGCCATTCTCATCATTGCCGCCGGTACT
GGTGAGTTCGAGGCTGGTATCTCCAAGGATGGCCAGACCCGTGAGCACGCTCTGCTCGCCTACACC
CTGGGTGTCAAGCAGCTCATCGTCGCCATCAACAAGATGGACACTCCTTGAGGCCATCGACTCCATC
GAGCCCCCCAAGCGTCCCACGGACAAGCCCCTCCGTCTTCCCCTCCAGGATGTGTACAAGATCGGTG
GTATCGGAACGGTTCCCGTCGGCCGTATCGAGACTGGTGTCCTCAAGCCCGGTATGGTCGTTACCTT
CGCTCCTTCCAACGTCACCACTGAAGTCAAGTCCGTCGAGATGCACCACGAGCAGCTCGTTGAGGGT
GTTCCCGGTGACAACGTTGGTTTCAACGTGAAGAACGTTTCCGTCAAGGAAATCCGCCGTGGTAACG
TTGCCGGTGACTCCAAGAACGACCCCCCCATGGGTGCCGCTTCTTTCACCGCCCAGGTCATCGTCAT
GAACCACCCCGGTCAGGTCGGTGCCGGCTACGCCCCCGTCCTTGACTGCCACACTGCCCACATTGCC
TGCAAGTTCGCCGAGCTCCAGGAGAAGATCGACCGCCGTACCGGTAAGGCTACCGAGACTGCCCCC
AAGTTCATCAAGTCCGGTGACTCTGCCATCGTCAAGATGATTCCCTCCAAGCCCATGTGCGTTGAGG
CTTTCACCGACTACCCTCCCCTGGGTCGTTTCGCCGTCCGTGACATGCGACAGACCGTCGCTGTCGGT
GTCATCAAGGCTGTCGACAAGTCCGCTGCCACCGCTGGCAAGGTCACCAAGTCCGCTGCCAAGGCC
ACCAAGAAATAA
